# Supplementary material for: Restoration of defective oxidative phosphorylation to a subset of neurons prevents mitochondrial encephalopathy
Source: EMBO Mol Med. 2024 Aug 21;16(9):13. doi: 10.1038/s44321-024-00111-4 (PMC11392956; doi:10.1038/s44321-024-00111-4)
Supplement: Supplementary file 1 — Appendix [file 44321_2024_111_MOESM1_ESM.pdf]

Appendix Table of Contents

Appendix Figure S1. Gene replacement therapy improves locomotive function in OXPHOS-nKO mice. (p. 2)
Appendix Figure S2. Mitochondrial DNA copy number is unaffected in *Ndufs3*-nKO mice. (p. 3)
Appendix Figure S3. Cre-driven recombination and gene ablation over time. (p.4)
Appendix Figure S4. Dose-response study of AAV.PHP.eB-hSYN-eGFP (p. 5)
Appendix Figure S5. Total Protein Loading and respective blots. (p. 6)
Appendix Table S1. Exact p-values for statistical analyses (p. 12)
Appendix Table S2. Genotyping primers (p. 26)
Appendix Table S3. Digital PCR primers and probes (p. 27)

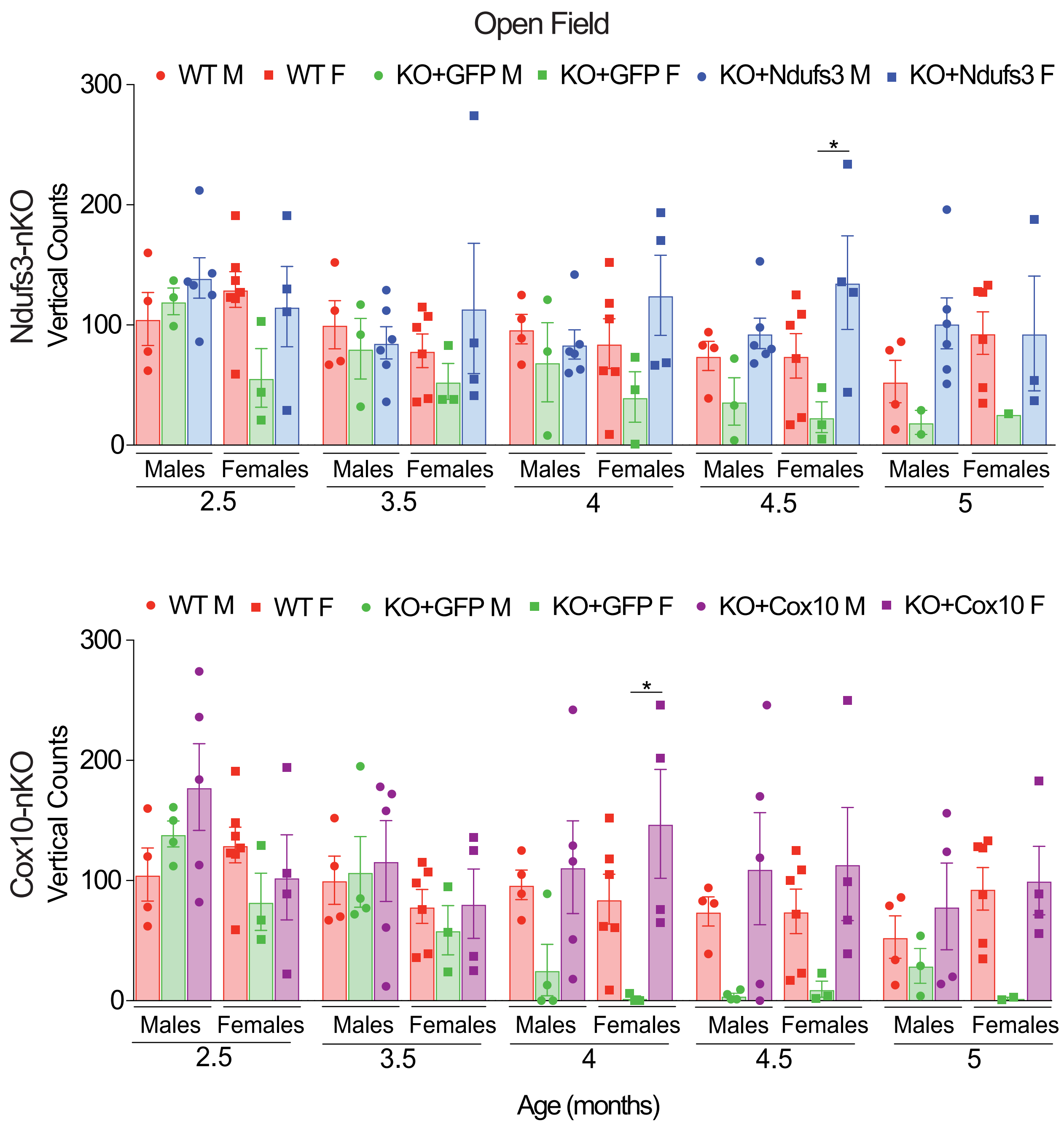

Appendix Figure S1. Gene replacement therapy improves locomotive function A) Vertical counts recorded during open field analysis of Ndufs3-nKO (top) and Cox10-nKO (bottom) mice at 2.5, 3.5, 4, 4.5, and 5 months of age.

Data information: In (A), data are represented as mean ± SD. For Ndufs3-nKO, WT males (red circles, n=4), WT females (red squares, n=6), KO+GFP males (green circles, n=3) KO+GFP females (green squares, n=3), KO+Ndufs3 males (blue circles, n=6), and KO+Ndufs3 females (blue squares, n=4). For Cox10-nKO, WT males (red circles, n=4), WT females (red squares, n=6), KO+GFP males (green circles, n=4) KO+GFP females (green squares, n=3), KO+Cox10 males (purple circles, n=5),and KO+Cox10 females (purple squares, n=4). P values were calculated using two-way ANOVA. P(*)= 0.0332, P(**)=0.0021, P(***)=0.0002, P(****)<0.0001.

mtDNA Levels

A

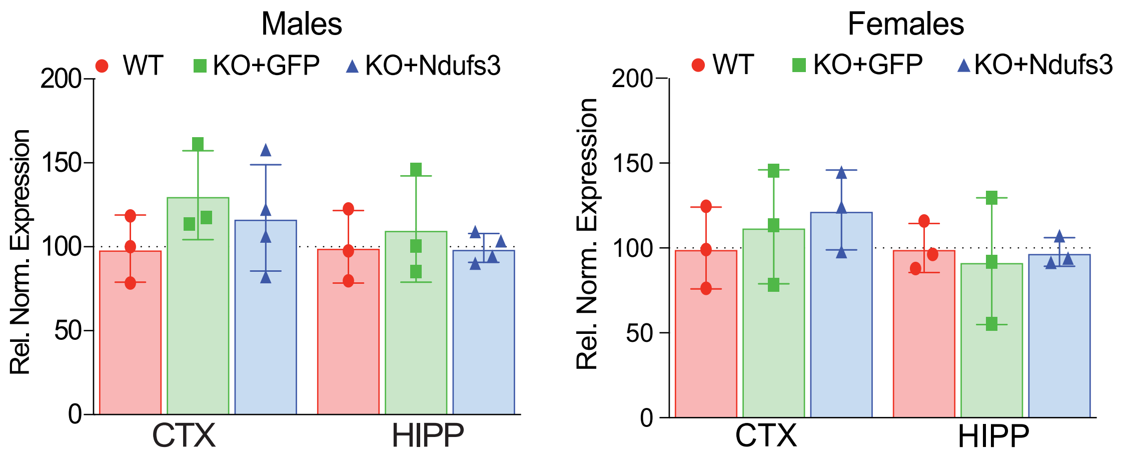

Appendix Figure S2. Mitochondrial DNA copy number is unaffected in Ndufs3-nKO mice.

mtDNA levels measured by digital PCR in DNA extracted from cortex and hippocampus of 5-month-old mice.

Data information: Data are represented as mean ± SD (n = 3-4/group). P values were calculated using One-way ANOVA. No significant differences detected.

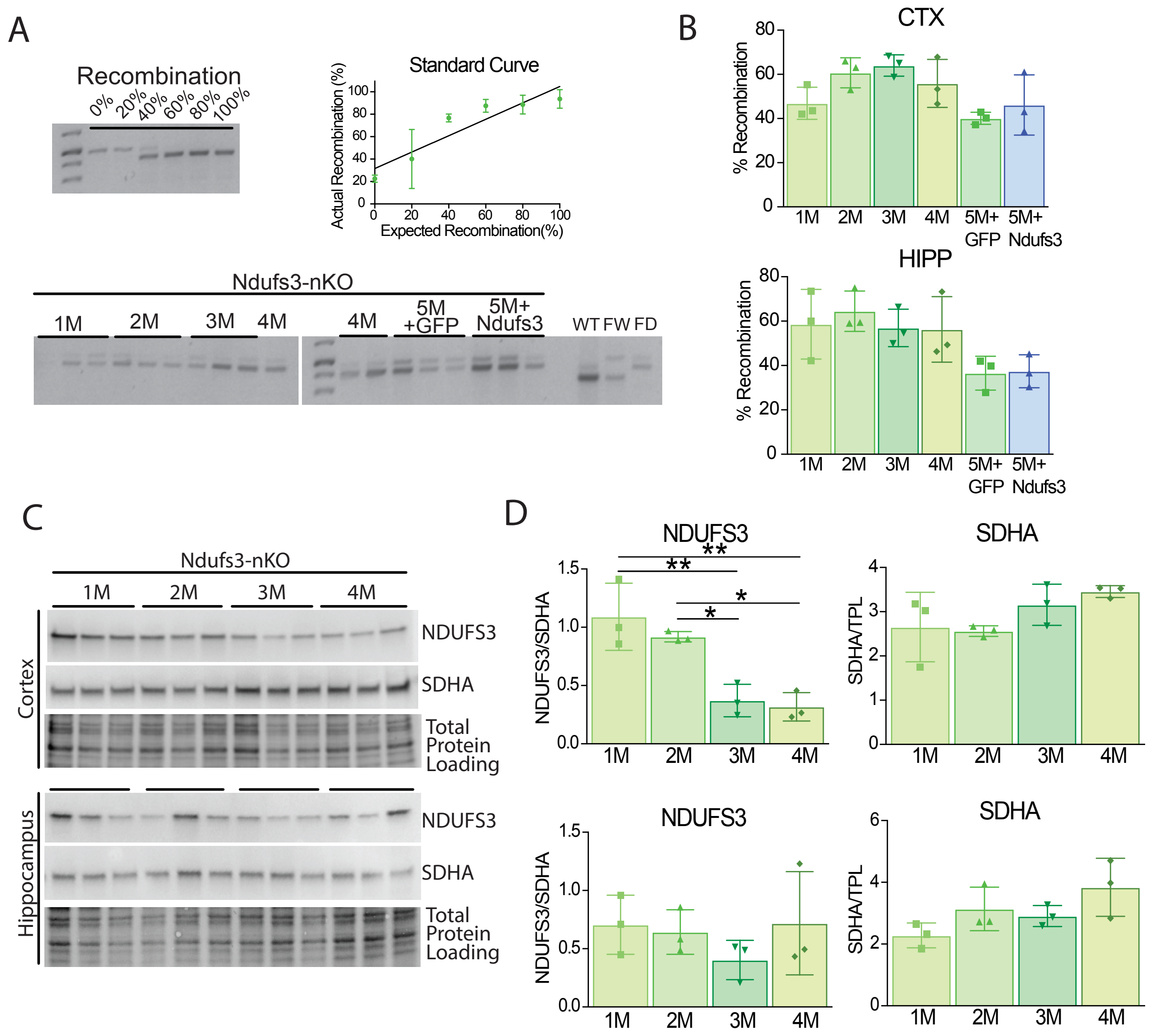

Appendix Figure S3. Cre-driven recombination and gene ablation over time.

A) Representative gel of three primer PCR, showing recombination of Ndufs3-nKO mice at 1-month (1M), 2-month (2M), 3-month (3M), 4-month (4M), and 5-month with AAV-PHP.eB-GFP or AAV-PHP.eB-Ndufs3 (5M+GFP, +Ndufs3). A 6-point standard curve generated from purified floxed and deleted bands.

B) Quantification of the three primer PCR for cortex (top) and hippocampus (bottom).

C, D) Western blots and relative quantifications of protein homogenates from cortical and hippocampal homogenates for Ndufs3-nKO mice at 1M, 2M, 3M, and 4M. Probed for NDUFS3 and SDHA. Total protein loading was used as loading control for SDHA.

Data information: In (B, D), data are represented as mean ± SD (n = 3/group). P values were calculated using One-way ANOVA. P(*)= 0.0332, P(**)=0.0021, P(***)=0.0002, P(****)<0.0001.

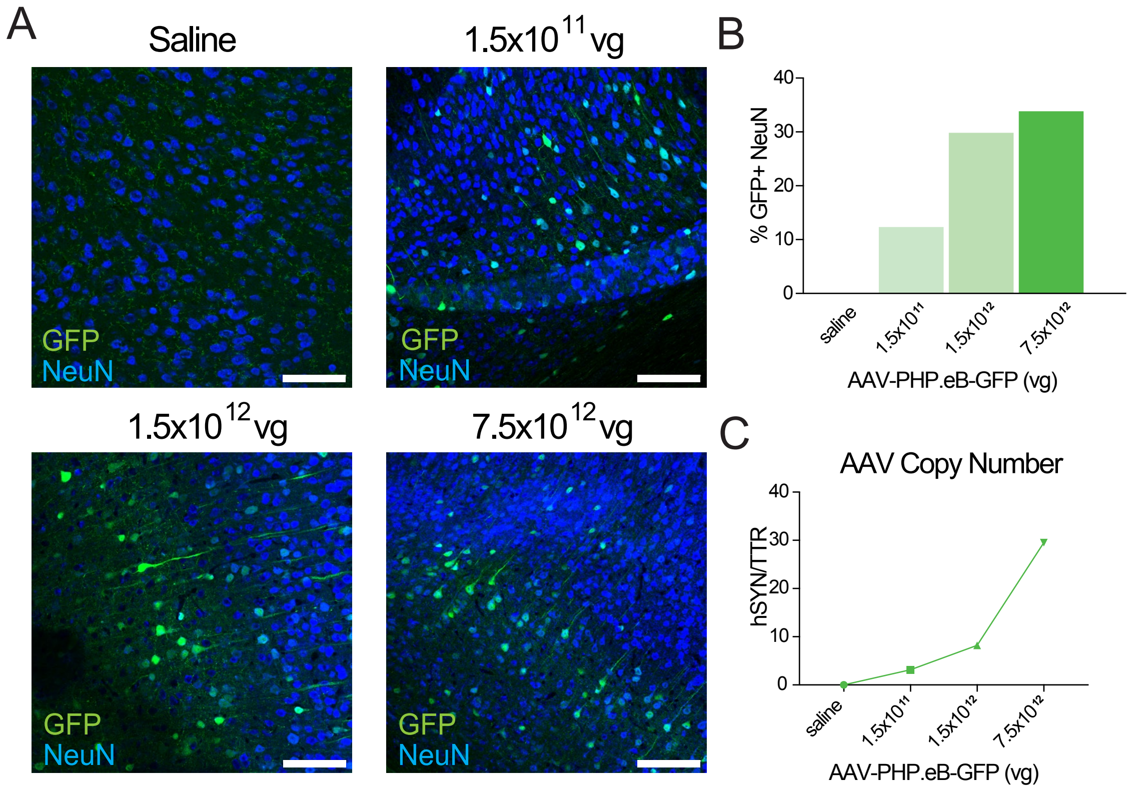

Appendix Figure S4. Dose-response study of AAV.PHP.eB-hSYN-eGFP
A) Representative images of GFP (green) expression in various brain regions of GFP-injected animals, 2 months post injection, 5-months of age. Scale bar is 100um.
B) Quantifications of GFP-positive cells in cortex.
C) AAV copy number as calculated by digital PCR analysis of the hSYN promoter.

Data information: Statistics were not performed as n=1.

Appendix Figure S5: Total protein loading and respective blots

In some instances the same gel was used for different antibodies, but only a representative protein loading was shown in the figure.

This Appendix shows all protein loading staining with their respective blots:

Cortex

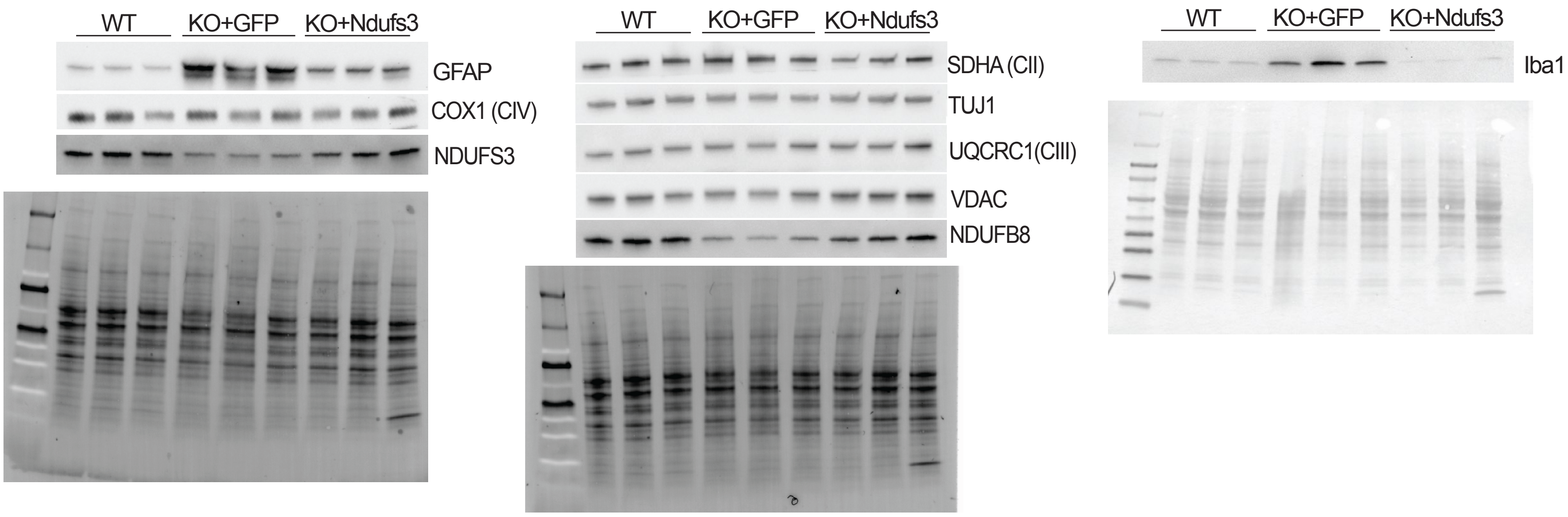

Blots in Figures 3 and 6

Hippocampus

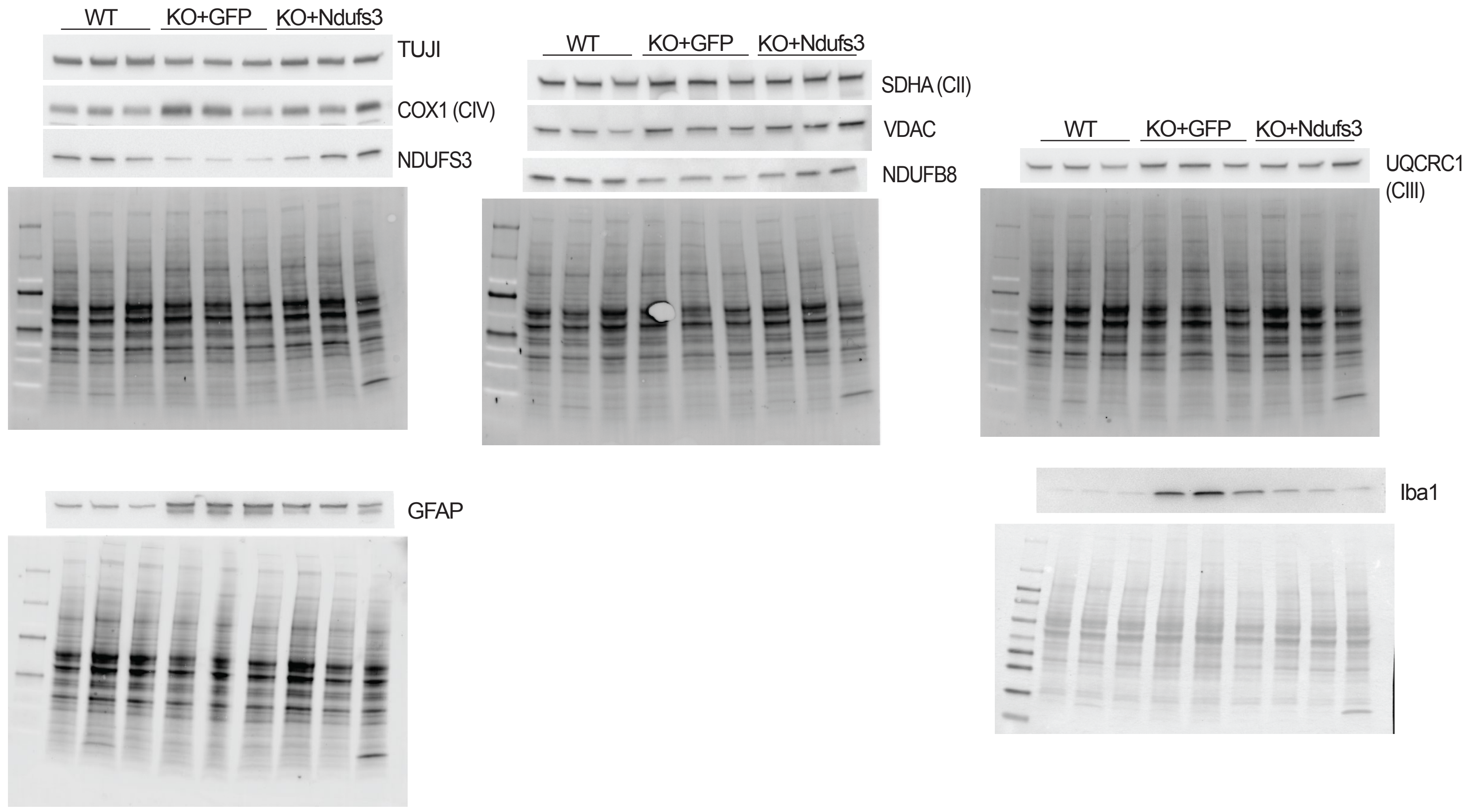

Blots in Figures 3 and 6

Cortex

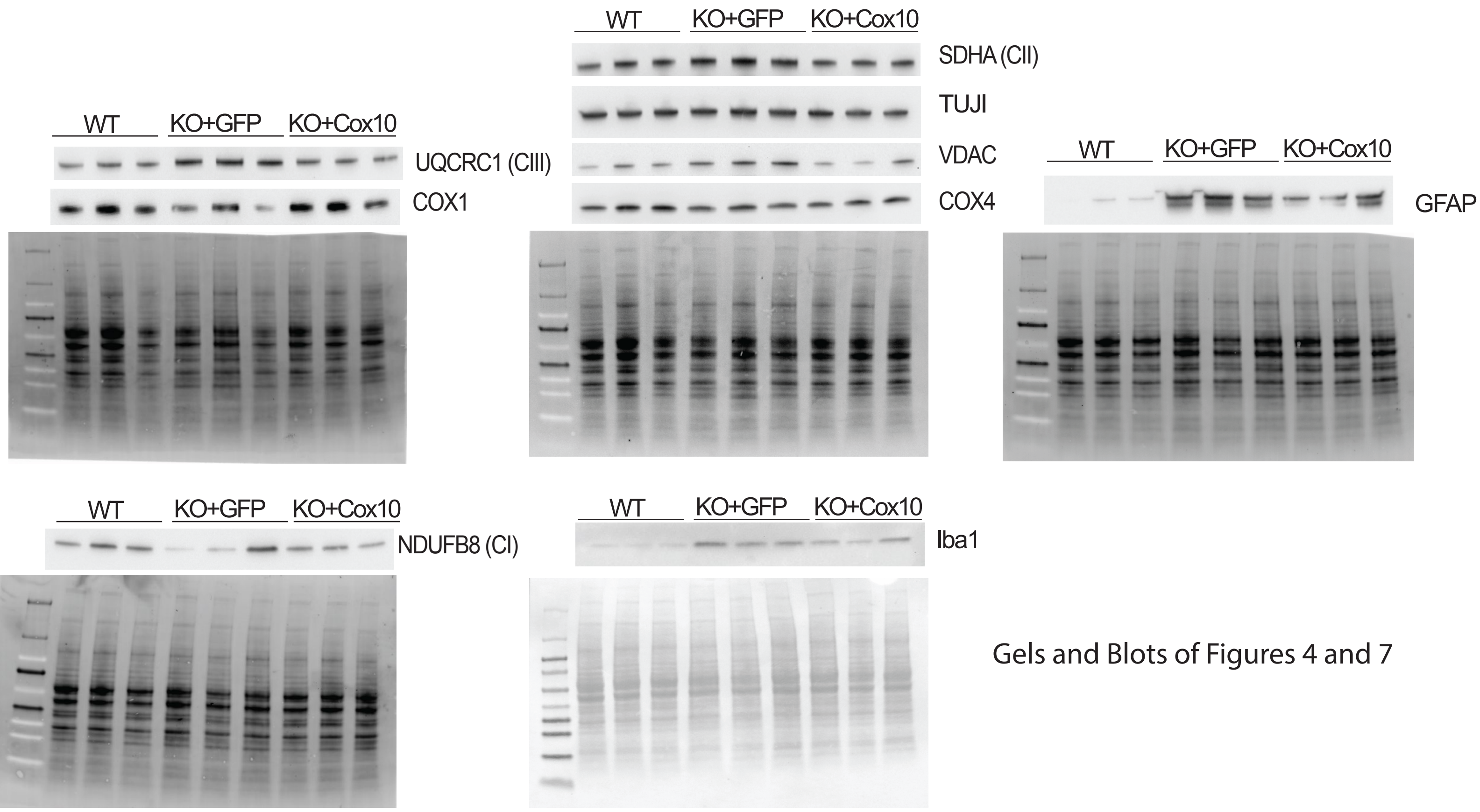

Gels and Blots of Figures 4 and 7

Hippocampus

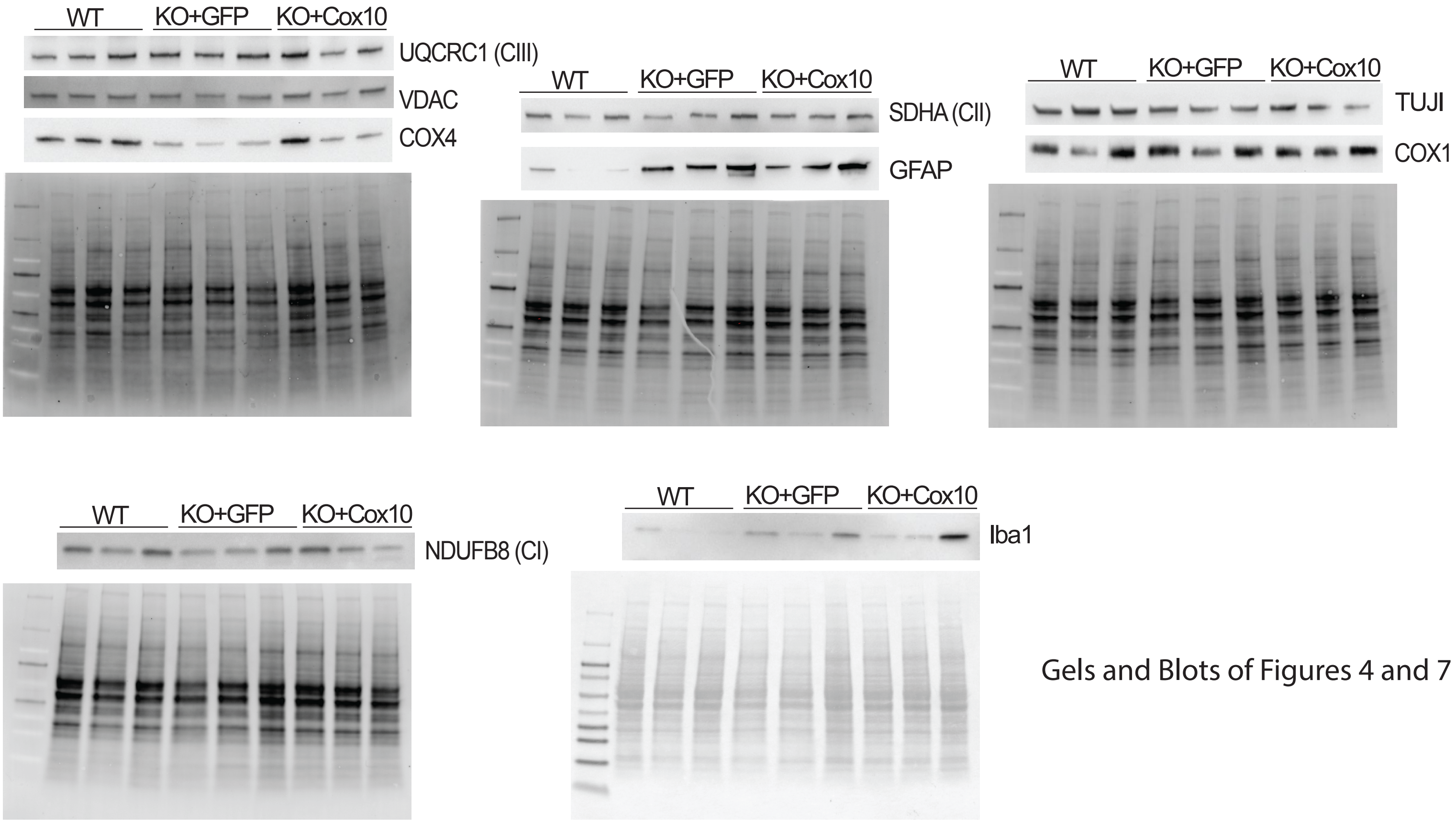

Gels and Blots of Figures 4 and 7

Cortex

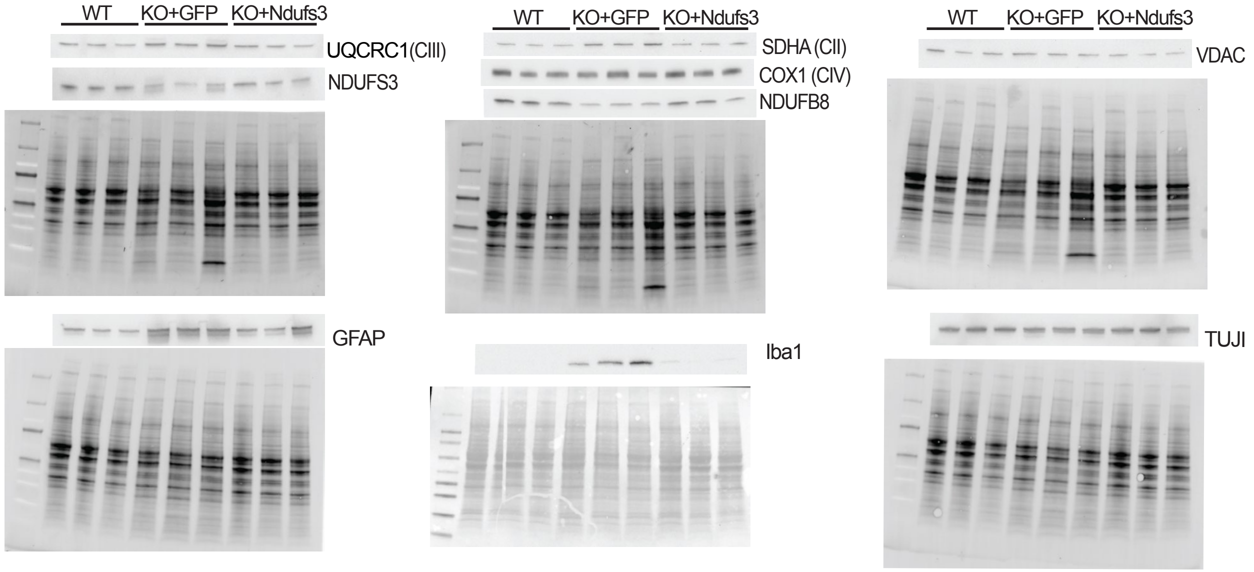

Gels and Blots Figure EV1

Hippocampus

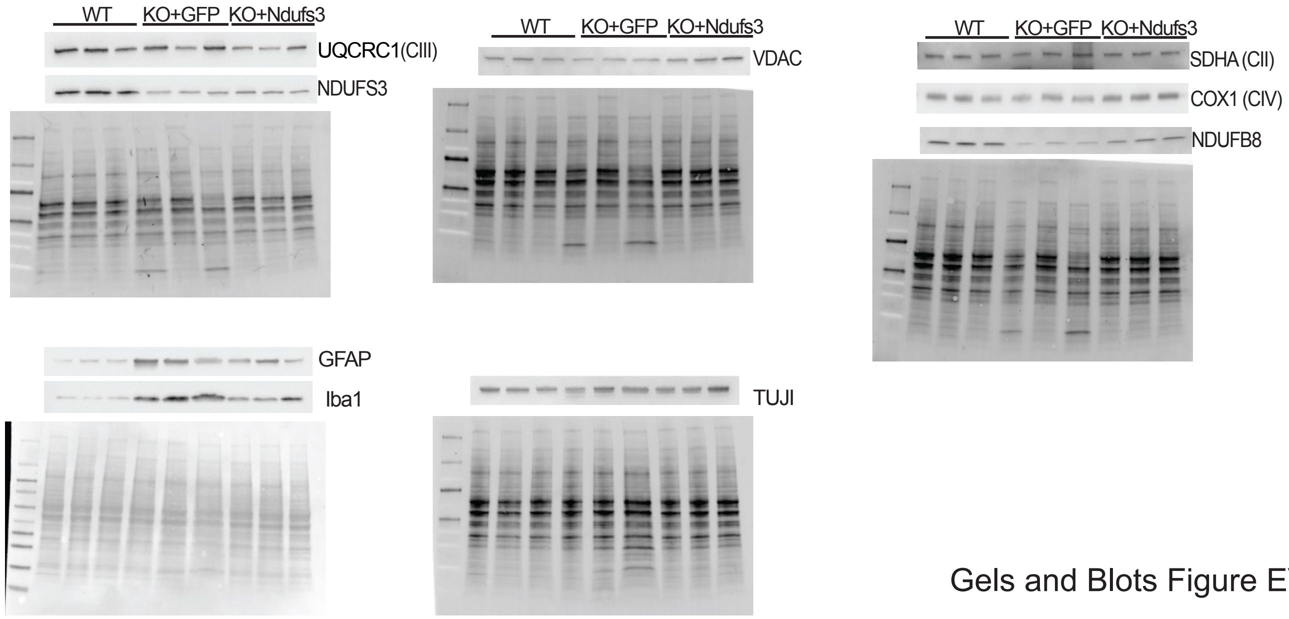

Gels and Blots Figure EV1-2

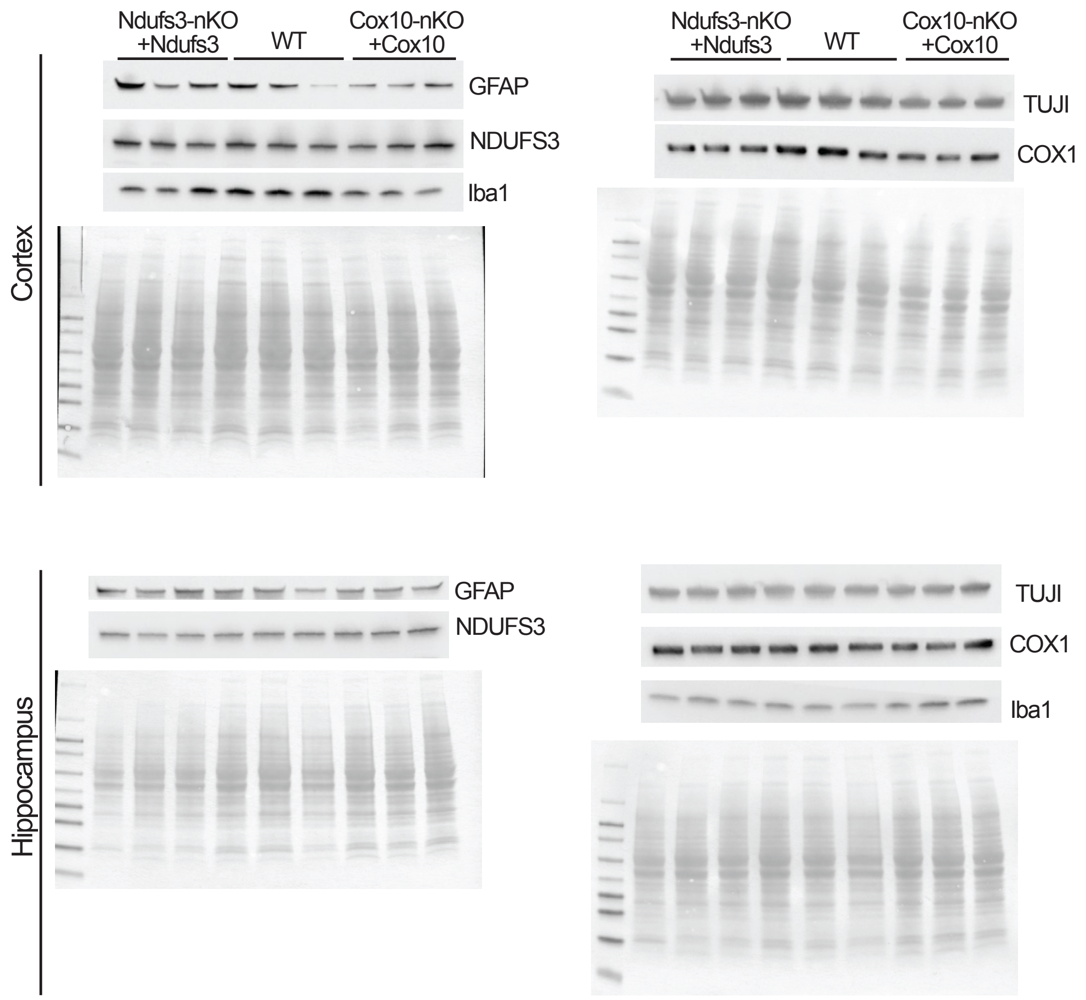

Gels and Blots Figure EV3-4

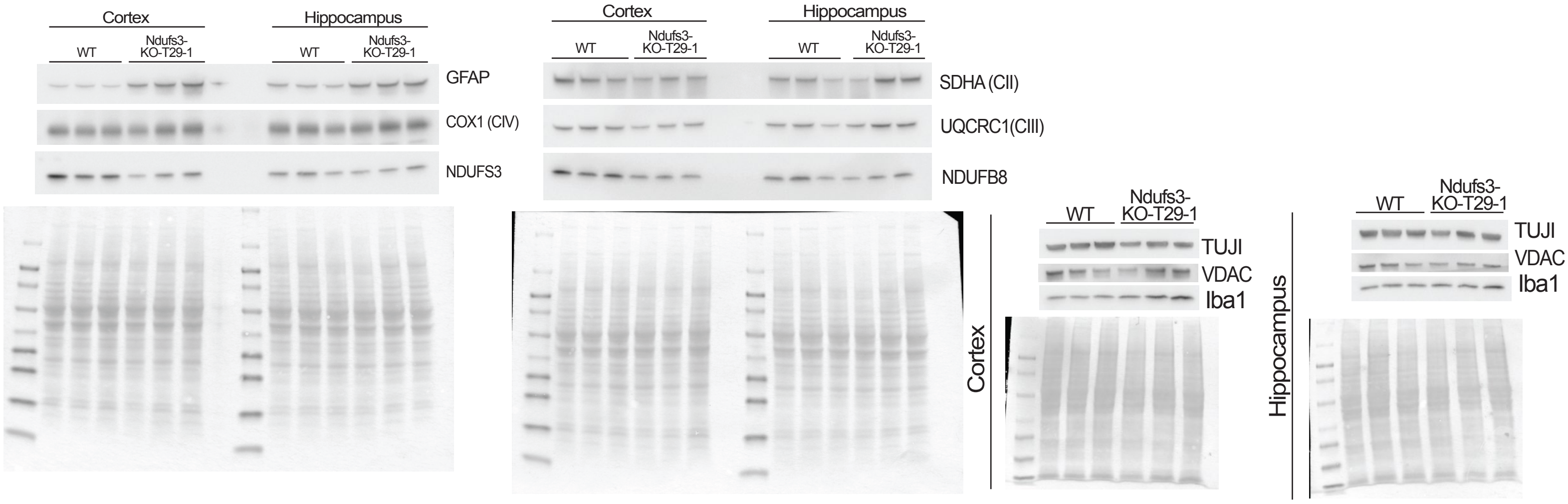

Gels and Blots Figure EV5

Appendix Table S1: Exact P values

C

	1.5M		1.75M		2M		2.25M		2.5M		2.75M	
	p value	symbol	p value	symbol	p value	symbol	p value	symbol	p value	symbol	p value	symbol
WT M vs. WT F	0.3186	ns	0.0918	ns	0.0171	*	0.0494	*	0.0218	*	0.0506	ns
WT M vs. KO+GFP M	0.9976	ns	0.9891	ns	0.9974	ns	0.8894	ns	0.9383	ns	0.8792	ns
WT M vs. KO+GFP F	0.4529	ns	0.2747	ns	0.1203	ns	0.1401	ns	0.6194	ns	0.2187	ns
WT M vs. KO+Ndufs3 M	0.9994	ns	0.9996	ns	0.9991	ns	0.9997	ns	0.9696	ns	0.8864	ns
WT M vs. KO+Ndufs3 F	0.2353	ns	0.2405	ns	0.0164	*	0.0113	*	0.0926	ns	0.0752	ns
WT F vs. KO+GFP M	0.0401	*	0.0024	**	0.0084	**	0.0028	**	0.0017	**	0.0029	**
WT F vs. KO+GFP F	>0.9999	ns	0.9992	ns	0.9995	ns	>0.9999	ns	0.9719	ns	0.9986	ns
WT F vs. KO+Ndufs3 M	0.0111	*	0.0009	***	0.0013	**	0.0072	**	0.0002	***	0.0005	***
WT F vs. KO+Ndufs3 F	0.9997	ns	0.9924	ns	>0.9999	ns	0.9954	ns	>0.9999	ns	>0.9999	ns
KO+GFP M vs. KO+GFP F	0.1151	ns	0.0274	*	0.0616	ns	0.0134	*	0.2112	ns	0.0231	*
KO+GFP M vs. KO+Ndufs3 M	>0.9999	ns	0.9977	ns	>0.9999	ns	0.9467	ns	0.9997	ns	>0.9999	ns
KO+GFP M vs. KO+Ndufs3 F	0.0257	*	0.0138	*	0.008	**	0.0005	***	0.0111	*	0.0047	**
KO+GFP F vs. KO+Ndufs3 M	0.071	ns	0.0253	*	0.0287	*	0.0431	*	0.2032	ns	0.0093	**
KO+GFP F vs. KO+Ndufs3 F	0.9998	ns	>0.9999	ns	0.9994	ns	0.9955	ns	0.9922	ns	0.9998	ns
KO+Ndufs3 M vs. KO+Ndufs3 F	0.0069	**	0.0082	**	0.0012	**	0.001	***	0.0039	**	0.0009	***

	3M		3.25M		3.5M		3.75M		4M		4.25M		4.5M		4.75M		5M	
	p value	symbol	p value	symbol	p value	symbol	p value	symbol	p value	symbol	p value	symbol	p value	symbol	p value	symbol	p value	symbol
WT M vs. WT F	0.0521	ns	0.0348	*	0.0176	*	0.0156	*	0.0148	*	0.0101	*	0.017	*	0.0301	*	0.0099	**
WT M vs. KO+GFP M	0.9893	ns	0.2265	ns	0.6391	ns	0.0839	ns	0.0162	*	0.1232	ns	>0.9999	ns	0.7107	ns	0.8163	ns
WT M vs. KO+GFP F	0.2056	ns	0.9888	ns	0.484	ns	0.4641	ns	0.5074	ns	0.0746	ns	0.0026	**	<0.0001	****	0.1898	ns
WT M vs. KO+Ndufs3 M	0.9041	ns	0.3716	ns	0.9485	ns	0.8815	ns	0.9476	ns	0.8815	ns	0.939	ns	0.9516	ns	0.9832	ns
WT M vs. KO+Ndufs3 F	0.2881	ns	0.0776	ns	0.0316	*	0.1132	ns	0.3542	ns	0.1272	ns	0.0475	*	0.0421	*	0.0308	*
WT F vs. KO+GFP M	0.0439	*	<0.0001	****	<0.0001	****	<0.0001	****	<0.0001	****	<0.0001	****	0.0111	*	0.7294	ns	0.743	ns
WT F vs. KO+GFP F	>0.9999	ns	0.0098	**	0.9084	ns	0.8282	ns	0.8746	ns	0.9982	ns	0.8651	ns	0.004	**	0.9991	ns
WT F vs. KO+Ndufs3 M	0.0003	***	<0.0001	****	<0.0001	****	<0.0001	****	0.0002	***	<0.0001	****	<0.0001	****	0.0005	***	0.0001	***
WT F vs. KO+Ndufs3 F	0.9939	ns	>0.9999	ns	>0.9999	ns	0.9803	ns	0.9575	ns	0.9475	ns	>0.9999	ns	>0.9999	ns	>0.9999	ns
KO+GFP M vs. KO+GFP F	0.1282	ns	0.6048	ns	0.0259	*	0.0005	***	<0.0001	****	<0.0001	****	0.0017	**	<0.0001	****	0.8169	ns
KO+GFP M vs. KO+Ndufs3 M	>0.9999	ns	0.9631	ns	0.9578	ns	0.4132	ns	0.1059	ns	0.5234	ns	0.9681	ns	0.1812	ns	0.4294	ns
KO+GFP M vs. KO+Ndufs3 F	0.1817	ns	0.0002	***	0.0003	***	<0.0001	****	<0.0001	****	<0.0001	****	0.0335	*	0.791	ns	0.8163	ns
KO+GFP F vs. KO+Ndufs3 M	0.0096	**	0.8791	ns	0.0728	ns	0.0398	*	0.0915	ns	0.0014	**	<0.0001	****	<0.0001	****	0.0586	ns
KO+GFP F vs. KO+Ndufs3 F	0.9995	ns	0.0244	*	0.9064	ns	0.9936	ns	>0.9999	ns	0.9986	ns	0.8549	ns	0.0027	**	0.9987	ns
KO+Ndufs3 M vs. KO+Ndufs3 F	0.012	*	<0.0001	****	0.0005	***	0.0017	**	0.0466	*	0.0021	**	0.0002	***	0.0008	***	0.0011	**

E

	1.5M		1.75M		2M		2.25M		2.5M		2.75M	
	p value	symbol	p value	symbol	p value	symbol	p value	symbol	p value	symbol	p value	symbol
WT M vs. WT F	0.2139	ns	0.0442	*	0.0055	**	0.0204	*	0.0074	**	0.021	*
WT M vs. KO+GFP M	0.9634	ns	0.9992	ns	0.9975	ns	0.997	ns	>0.9999	ns	0.9998	ns
WT M vs. KO+GFP F	0.0593	ns	0.0458	*	0.0014	**	0.0219	*	0.1246	ns	0.0319	*
WT M vs. KO+Cox10 M	0.9995	ns	0.9975	ns	0.9901	ns	0.9944	ns	0.9724	ns	0.963	ns
WT M vs. KO+Cox10 F	>0.9999	ns	0.9996	ns	>0.9999	ns	0.9994	ns	>0.9999	ns	0.9651	ns
WT F vs. KO+GFP M	0.4956	ns	0.0206	*	0.0264	*	0.0838	ns	0.0033	**	0.0092	**
WT F vs. KO+GFP F	0.9137	ns	0.9997	ns	0.9597	ns	0.9798	ns	>0.9999	ns	>0.9999	ns
WT F vs. KO+Cox10 M	0.1017	ns	0.0002	***	0.0002	***	0.0014	**	<0.0001	****	0.0006	***
WT F vs. KO+Cox10 F	0.0318	*	0.0031	**	0.017	*	0.0153	*	0.0059	**	0.003	**
KO+GFP M vs. KO+GFP F	0.152	ns	0.0289	*	0.0066	**	0.0664	ns	0.0836	ns	0.0154	*
KO+GFP M vs. KO+Cox10 M	0.9868	ns	0.9069	ns	0.8834	ns	0.9034	ns	0.9936	ns	0.9938	ns
KO+GFP M vs. KO+Cox10 F	0.8556	ns	0.9698	ns	0.999	ns	0.9715	ns	>0.9999	ns	0.9925	ns
KO+GFP F vs. KO+Cox10 M	0.0232	*	0.0006	***	<0.0001	****	0.0035	**	0.018	*	0.0015	**
KO+GFP F vs. KO+Cox10 F	0.0071	**	0.005	**	0.0042	**	0.0151	*	0.1118	ns	0.0051	**
KO+Cox10 M vs. KO+Cox10 F	0.9928	ns	>0.9999	ns	0.9903	ns	>0.9999	ns	0.9807	ns	>0.9999	ns

	3M		3.25M		3.5M		3.75M		4M		4.25M		4.5M		4.75M		5M	
	p value	symbol	p value	symbol	p value	symbol	p value	symbol	p value	symbol	p value	symbol	p value	symbol	p value	symbol	p value	symbol
WT M vs. WT F	0.0218	*	0.0132	*	0.0057	**	0.0049	**	0.0046	**	0.0029	**	0.0054	**	0.011	*	0.0027	**
WT M vs. KO+GFP M	0.9991	ns	0.9824	ns	0.6994	ns	0.8054	ns	0.7956	ns	>0.9999	ns	0.2423	ns	0.3425	ns	0.0235	*
WT M vs. KO+GFP F	0.0205	*	0.0117	*	0.0015	**	0.0557	ns	0.1128	ns	0.0172	*	0.0001	***	<0.0001	****	<0.0001	****
WT M vs. KO+Cox10 M	0.4638	ns	0.8019	ns	0.9375	ns	0.9802	ns	0.6867	ns	0.8704	ns	0.9997	ns	0.9754	ns	0.9975	ns
WT M vs. KO+Cox10 F	0.9999	ns	0.9946	ns	>0.9999	ns	>0.9999	ns	>0.9999	ns	0.9866	ns	0.9871	ns	0.9929	ns	>0.9999	ns
WT F vs. KO+GFP M	0.0058	**	0.0011	**	<0.0001	****	<0.0001	****	<0.0001	****	0.0029	**	0.9053	ns	0.8762	ns	>0.9999	ns
WT F vs. KO+GFP F	0.994	ns	0.9998	ns	0.9217	ns	0.9958	ns	0.994	ns	0.9999	ns	0.5524	ns	0.2272	ns	0.0027	**
WT F vs. KO+Cox10 M	<0.0001	****	<0.0001	****	<0.0001	****	0.0001	***	<0.0001	****	<0.0001	****	0.0001	***	0.0007	***	0.0004	***
WT F vs. KO+Cox10 F	0.0092	**	0.0049	**	0.0057	**	0.0073	**	0.004	**	0.0007	***	0.0222	*	0.0036	**	0.0016	**
KO+GFP M vs. KO+GFP F	0.0067	**	0.0012	**	<0.0001	****	0.0011	**	0.003	**	0.0172	*	0.1797	ns	0.0236	*	0.013	*
KO+GFP M vs. KO+Cox10 M	0.7028	ns	0.9947	ns	0.9917	ns	0.9898	ns	>0.9999	ns	0.8704	ns	0.0672	ns	0.0785	ns	0.0059	**
KO+GFP M vs. KO+Cox10 F	>0.9999	ns	>0.9999	ns	0.6994	ns	0.9124	ns	0.8149	ns	0.9866	ns	0.5335	ns	0.1567	ns	0.0163	*
KO+GFP F vs. KO+Cox10 M	<0.0001	****	<0.0001	****	<0.0001	****	0.0047	**	0.001	**	0.0002	***	<0.0001	****	<0.0001	****	<0.0001	****
KO+GFP F vs. KO+Cox10 F	0.0099	**	0.0044	**	0.0015	**	0.0621	ns	0.104	ns	0.0044	**	0.0005	***	<0.0001	****	<0.0001	****
KO+Cox10 M vs. KO+Cox10 F	0.6254	ns	0.9907	ns	0.9375	ns	0.9963	ns	0.7107	ns	0.9993	ns	0.8937	ns	>0.9999	ns	0.9995	ns

FIGURE 2C and E

D

Age (Months)	2.5		3.5		4		4.5		5	
Comparison	p value	symbol	p value	symbol	p value	symbol	p value	symbol	p value	symbol
WT M vs. WT F	0.9964	ns	>0.9999	ns	>0.9999	ns	>0.9999	ns	>0.9999	ns
WT M vs. KO+GFP M	0.9984	ns	0.9958	ns	<0.0001	****	<0.0001	****	<0.0001	****
WT M vs. KO+GFP F	0.9984	ns	0.3034	ns	<0.0001	****	<0.0001	****	<0.0001	****
WT M vs. KO+NDUFS3 M	0.9964	ns	0.975	ns	>0.9999	ns	0.9994	ns	>0.9999	ns
WT M vs. KO+NDUFS3 F	0.9976	ns	>0.9999	ns	0.7135	ns	0.5047	ns	0.7613	ns
WT F vs. KO+GFP M	>0.9999	ns	0.994	ns	<0.0001	****	<0.0001	****	<0.0001	****
WT F vs. KO+GFP F	>0.9999	ns	0.2238	ns	<0.0001	****	<0.0001	****	<0.0001	****
WT F vs. KO+NDUFS3 M	>0.9999	ns	0.9596	ns	>0.9999	ns	0.9989	ns	>0.9999	ns
WT F vs. KO+NDUFS3 F	>0.9999	ns	>0.9999	ns	0.6278	ns	0.4002	ns	0.6845	ns
KO+GFP M vs. KO+GFP F	>0.9999	ns	0.6796	ns	0.9418	ns	0.9922	ns	0.9998	ns
KO+GFP M vs. KO+NDUFS3 M	>0.9999	ns	>0.9999	ns	<0.0001	****	<0.0001	****	<0.0001	****
KO+GFP M vs. KO+NDUFS3 F	>0.9999	ns	0.9958	ns	<0.0001	****	<0.0001	****	<0.0001	****
KO+GFP F vs. KO+NDUFS3 M	>0.9999	ns	0.6209	ns	<0.0001	****	<0.0001	****	<0.0001	****
KO+GFP F vs. KO+NDUFS3 F	>0.9999	ns	0.3034	ns	<0.0001	****	<0.0001	****	<0.0001	****
KO+NDUFS3 M vs. KO+NDUFS3 F	>0.9999	ns	0.975	ns	0.6278	ns	0.6188	ns	0.6845	ns

F

Age (Months)	2.5		3.5		4		4.5		5	
Comparison	p value	symbol	p value	symbol	p value	symbol	p value	symbol	p value	symbol
WT M vs. WT F	0.9993	ns	>0.9999	ns	>0.9999	ns	>0.9999	ns	>0.9999	ns
WT M vs. KO+GFP M	0.8034	ns	0.4752	ns	0.9724	ns	<0.0001	****	<0.0001	****
WT M vs. KO+GFP F	0.9997	ns	>0.9999	ns	0.0013	**	<0.0001	****	<0.0001	****
WT M vs. KO+Cox10 M	>0.9999	ns	0.9992	ns	0.1706	ns	0.0628	ns	0.397	ns
WT M vs. KO+Cox10 F	>0.9999	ns	0.9998	ns	0.9386	ns	0.9979	ns	>0.9999	ns
WT F vs. KO+GFP M	0.5116	ns	0.3705	ns	0.9592	ns	<0.0001	****	<0.0001	****
WT F vs. KO+GFP F	>0.9999	ns	>0.9999	ns	0.0004	***	<0.0001	****	<0.0001	****
WT F vs. KO+Cox10 M	0.9999	ns	0.9987	ns	0.0961	ns	0.0277	*	0.2949	ns
WT F vs. KO+Cox10 F	0.9996	ns	0.9997	ns	0.9117	ns	0.9968	ns	>0.9999	ns
KO+GFP M vs. KO+GFP F	0.6857	ns	0.5614	ns	0.0137	*	0.9998	ns	0.9865	ns
KO+GFP M vs. KO+Cox10 M	0.6986	ns	0.6456	ns	0.6135	ns	0.0071	**	0.0001	***
KO+GFP M vs. KO+Cox10 F	0.7814	ns	0.6479	ns	>0.9999	ns	<0.0001	****	<0.0001	****
KO+GFP F vs. KO+Cox10 M	>0.9999	ns	0.9995	ns	0.3141	ns	0.0077	**	0.0105	*
KO+GFP F vs. KO+Cox10 F	0.9998	ns	0.9999	ns	0.0213	*	<0.0001	****	<0.0001	****
KO+Cox10 M vs. KO+Cox10 F	>0.9999	ns	>0.9999	ns	0.7202	ns	0.181	ns	0.397	ns

FIGURE 2D and F

Panel	Label	Comparison	p value	symbol
		WT vs. KO+GFP	0.0003	***
	NDUFS3	WT vs. KO+NDUFS3	0.6658	ns
		KO+GFP vs. KO+NDUFS3	0.0013	**
B		WT vs. KO+GFP	0.0001	***
	NDUFSB8	WT vs. KO+NDUFS3	0.0334	*
		KO+GFP vs. KO+NDUFS3	0.0193	*
		WT vs. KO+GFP	0.9183	ns
	SDHA	WT vs. KO+NDUFS3	0.495	ns
		KO+GFP vs. KO+NDUFS3	0.2933	ns
		WT vs. KO+GFP	0.9676	ns
C	UQCRC1	WT vs. KO+NDUFS3	0.4706	ns
		KO+GFP vs. KO+NDUFS3	0.616	ns
		WT vs. KO+GFP	0.1688	ns
	COX1	WT vs. KO+NDUFS3	0.9598	ns
		KO+GFP vs. KO+NDUFS3	0.1042	ns
		WT vs. KO+GFP	0.0003	***
	NDUFS3	WT vs. KO+NDUFS3	0.8479	ns
		KO+GFP vs. KO+NDUFS3	0.0007	***
E		WT vs. KO+GFP	0.0106	*
	NDUFSB8	WT vs. KO+NDUFS3	0.7189	ns
		KO+GFP vs. KO+NDUFS3	0.0431	*
		WT vs. KO+GFP	0.6642	ns
	SDHA	WT vs. KO+NDUFS3	0.9069	ns
		KO+GFP vs. KO+NDUFS3	0.895	ns
		WT vs. KO+GFP	0.3431	ns
F	UQCRC1	WT vs. KO+NDUFS3	0.8261	ns
		KO+GFP vs. KO+NDUFS3	0.6799	ns
		WT vs. KO+GFP	0.0333	*
	COX1	WT vs. KO+NDUFS3	0.2482	ns
		KO+GFP vs. KO+NDUFS3	0.5286	ns
		WT vs. KO+GFP	0.8561	ns
	CTX	WT vs. KO+NDUFS3	0.2311	ns
		KO+GFP vs. KO+NDUFS3	0.0986	ns
G		WT vs. KO+GFP	0.1853	ns
	HIPP	WT vs. KO+NDUFS3	0.0377	*
		KO+GFP vs. KO+NDUFS3	0.6249	ns
		WT vs. KO+GFP	<0.0001	****
H	NDUFS3+NeuN	WT vs. KO+NDUFS3	0.0015	**
		KO+GFP vs. KO+NDUFS3	0.0006	***

FIGURE 3

Panel	Label	Comparison	p value	symbol
B	COX1	WT vs. KO+GFP	0.0305	*
		WT vs. KO+COX10	0.564	ns
		KO+GFP vs. KO+COX10	0.0878	ns
	COX4	WT vs. KO+GFP	0.0404	*
		WT vs. KO+COX10	0.8108	ns
		KO+GFP vs. KO+COX10	0.0627	ns
C	NDUFB8	WT vs. KO+GFP	0.8782	ns
		WT vs. KO+COX10	0.9278	ns
		KO+GFP vs. KO+COX10	0.9924	ns
	SDHA	WT vs. KO+GFP	0.245	ns
		WT vs. KO+COX10	0.9828	ns
		KO+GFP vs. KO+COX10	0.184	ns
	UQCRC1	WT vs. KO+GFP	0.0027	**
		WT vs. KO+COX10	0.9699	ns
		KO+GFP vs. KO+COX10	0.0046	**
D	VDAC	WT vs. KO+GFP	0.0469	*
		WT vs. KO+COX10	0.8246	ns
		KO+GFP vs. KO+COX10	0.0996	ns
F	COX1	WT vs. KO+GFP	0.9341	ns
		WT vs. KO+COX10	0.9856	ns
		KO+GFP vs. KO+COX10	0.8656	ns
	COX4	WT vs. KO+GFP	0.0366	*
		WT vs. KO+COX10	0.3167	ns
		KO+GFP vs. KO+COX10	0.4086	ns
G	NDUFB8	WT vs. KO+GFP	0.8782	ns
		WT vs. KO+COX10	0.9278	ns
		KO+GFP vs. KO+COX10	0.9924	ns
	SDHA	WT vs. KO+GFP	0.245	ns
		WT vs. KO+COX10	0.9828	ns
		KO+GFP vs. KO+COX10	0.184	ns
	UQCRC1	WT vs. KO+GFP	0.0027	**
		WT vs. KO+COX10	0.9699	ns
		KO+GFP vs. KO+COX10	0.0046	**
H	VDAC	WT vs. KO+GFP	0.8806	ns
		WT vs. KO+COX10	0.7312	ns
		KO+GFP vs. KO+COX10	0.9554	ns
I	CTX	WT vs. KO+GFP	0.6573	ns
		WT vs. KO+COX10	0.9952	ns
		KO+GFP vs. KO+COX10	0.6016	ns
	HIPP	WT vs. KO+GFP	0.2237	ns
		WT vs. KO+COX10	0.9934	ns
		KO+GFP vs. KO+COX10	0.1892	ns
J	COX1+NeuN	WT vs. KO+GFP	0.0059	**
		WT vs. KO+COX10	0.6963	ns
		KO+GFP vs. KO+COX10	0.0139	*

FIGURE 4

Panel	Label	Comparison	p value	symbol
B	CI SC	WT vs. KO+GFP	0.004	**
		WT vs. KO+NDUFS3	0.7513	ns
		KO+GFP vs. KO+NDUFS3	0.0083	**
	CI-CIII	WT vs. KO+GFP	0.2584	ns
		WT vs. KO+NDUFS3	0.9952	ns
		KO+GFP vs. KO+NDUFS3	0.2974	ns
	CIII	WT vs. KO+GFP	0.999	ns
		WT vs. KO+NDUFS3	0.9822	ns
		KO+GFP vs. KO+NDUFS3	0.9897	ns
	Total CIII	WT vs. KO+GFP	0.5136	ns
		WT vs. KO+NDUFS3	>0.9999	ns
		KO+GFP vs. KO+NDUFS3	0.519	ns
	CIV	WT vs. KO+GFP	0.3469	ns
		WT vs. KO+NDUFS3	0.042	*
		KO+GFP vs. KO+NDUFS3	0.2838	ns
D	CI Activity	WT vs. KO+GFP	0.0013	**
		WT vs. KO+NDUFS3	0.5406	ns
		KO+GFP vs. KO+NDUFS3	0.0033	**
	CIV Activity	WT vs. KO+GFP	0.9947	ns
		WT vs. KO+NDUFS3	0.8788	ns
		KO+GFP vs. KO+NDUFS3	0.9203	ns
F	CIV	WT vs. KO+GFP	0.0696	ns
		WT vs. KO+COX10	0.2273	ns
		KO+GFP vs. KO+COX10	0.0082	**
	CI SC	WT vs. KO+GFP	0.1037	ns
		WT vs. KO+COX10	0.5905	ns
		KO+GFP vs. KO+COX10	0.0293	*
	CI-CIII	WT vs. KO+GFP	0.0801	ns
		WT vs. KO+COX10	0.9997	ns
		KO+GFP vs. KO+COX10	0.0764	ns
	CIII	WT vs. KO+GFP	0.0006	***
		WT vs. KO+COX10	0.2337	ns
		KO+GFP vs. KO+COX10	0.0253	*
	Total CIII	WT vs. KO+GFP	0.2699	ns
		WT vs. KO+COX10	0.9759	ns
		KO+GFP vs. KO+COX10	0.3654	ns
H	CIV Activity	WT vs. KO+GFP	0.184	ns
		WT vs. KO+COX10	0.7457	ns
		KO+GFP vs. KO+COX10	0.071	ns
	CI Activity	WT vs. KO+GFP	0.1136	ns
		WT vs. KO+COX10	0.4635	ns
		KO+GFP vs. KO+COX10	0.5198	ns

FIGURE 5

Panel	Label	Comparison	p value	symbol
GFAP (D)	CTX	WT vs. KO+GFP	<0.0001	****
		WT vs. KO+NDUFS3	0.0719	ns
		KO+GFP vs. KO+NDUFS3	<0.0001	****
	HIPP	WT vs. KO+GFP	0.0034	**
		WT vs. KO+NDUFS3	0.4471	ns
		KO+GFP vs. KO+NDUFS3	0.0322	*
Iba1 (D)	CTX	WT vs. KO+GFP	0.0557	ns
		WT vs. KO+NDUFS3	0.9775	ns
		KO+GFP vs. KO+NDUFS3	0.0389	*
	HIPP	WT vs. KO+GFP	0.0001	***
		WT vs. KO+NDUFS3	0.7981	ns
		KO+GFP vs. KO+NDUFS3	0.0003	***
TUJI (D)	CTX	WT vs. KO+GFP	0.0324	*
		WT vs. KO+NDUFS3	0.9942	ns
		KO+GFP vs. KO+NDUFS3	0.0389	*
	HIPP	WT vs. KO+GFP	0.0067	**
		WT vs. KO+NDUFS3	0.3656	ns
		KO+GFP vs. KO+NDUFS3	0.0824	ns
E	brain weight	WT vs. KO+GFP	0.1049	ns
		WT vs. KO+NDUFS3	0.6539	ns
		KO+GFP vs. KO+NDUFS3	0.505	ns

FIGURE 6

Panel	Label	Comparison	p value	symbol
B	brain weight	WT vs. KO+GFP	<0.0001	****
		WT vs. KO+COX10	0.047	*
		KO+GFP vs. KO+COX10	0.0233	*
GFAP (F)	CTX	WT vs. KO+GFP	0.0035	**
		WT vs. KO+COX10	0.0697	ns
		KO+GFP vs. KO+COX10	0.0741	ns
	HIPP	WT vs. KO+GFP	0.0717	ns
		WT vs. KO+COX10	0.1105	ns
		KO+GFP vs. KO+COX10	0.9385	ns
Iba1 (F)	CTX	WT vs. KO+GFP	0.0074	**
		WT vs. KO+COX10	0.3019	ns
		KO+GFP vs. KO+COX10	0.0466	*
	HIPP	WT vs. KO+GFP	0.4798	ns
		WT vs. KO+COX10	0.4125	ns
		KO+GFP vs. KO+COX10	0.9894	ns
TUJI (F)	CTX	WT vs. KO+GFP	0.2737	ns
		WT vs. KO+COX10	0.7048	ns
		KO+GFP vs. KO+COX10	0.097	ns
	HIPP	WT vs. KO+GFP	0.2174	ns
		WT vs. KO+COX10	0.1747	ns
		KO+GFP vs. KO+COX10	0.9836	ns
G	CA1 counts	WT vs. KO+GFP	0.0023	**
		WT vs. KO+COX10	0.0166	*
		KO+GFP vs. KO+COX10	0.1867	ns

FIGURE 7

Panel	Label	Comparison	p value	symbol
C	NDUFS3 CTX	WT vs. KO+GFP	0.146	ns
		WT vs. KO+NDUFS3	<0.0001	****
		KO+GFP vs. KO+NDUFS3	0.0028	**
	NDUFS3 HIPP	WT vs. KO+GFP	0.5858	ns
		WT vs. KO+NDUFS3	0.0002	***
		KO+GFP vs. KO+NDUFS3	0.004	**
	COX10 CTX	WT vs. KO+GFP	0.0039	**
		WT vs. KO+COX10	0.0995	ns
		KO+GFP vs. KO+COX10	0.268	ns
	COX10 HIPP	WT vs. KO+GFP	0.0218	*
		WT vs. KO+COX10	0.5831	ns
		KO+GFP vs. KO+COX10	0.1856	ns
E	Motor Cortex	Ndufs3-nKO vs COX10-nKO	0.5947	ns
	Piriform Cortex	Ndufs3-nKO vs COX10-nKO	0.5973	ns
	Hippocampus	Ndufs3-nKO vs COX10-nKO	0.4613	ns

FIGURE 8

Panel	Label	Comparison	p value	symbol
B	NDUFS3	WT vs. KO+GFP	0.0001	***
B	NDUFS3	WT vs. KO+NDUFS3	0.0075	**
B	NDUFS3	KO+GFP vs. KO+NDUFS3	0.063	ns
B	NDUFB8	WT vs. KO+GFP	0.0002	***
B	NDUFB8	WT vs. KO+NDUFS3	0.0483	*
B	NDUFB8	KO+GFP vs. KO+NDUFS3	0.0205	*
C	SDHA	WT vs. KO+GFP	0.9183	ns
C	SDHA	WT vs. KO+NDUFS3	0.495	ns
C	SDHA	KO+GFP vs. KO+NDUFS3	0.2933	ns
C	UQCRC1	WT vs. KO+GFP	0.9676	ns
C	UQCRC1	WT vs. KO+NDUFS3	0.4706	ns
C	UQCRC1	KO+GFP vs. KO+NDUFS3	0.616	ns
D	COX1	WT vs. KO+GFP	0.1688	ns
D	COX1	WT vs. KO+NDUFS3	0.9598	ns
D	COX1	KO+GFP vs. KO+NDUFS3	0.1042	ns
D	VDAC	WT vs. KO+GFP	0.5398	ns
D	VDAC	WT vs. KO+NDUFS3	0.9243	ns
D	VDAC	KO+GFP vs. KO+NDUFS3	0.7529	ns
F	NDUFS3	WT vs. KO+GFP	0.0003	***
F	NDUFS3	WT vs. KO+NDUFS3	0.8479	ns
F	NDUFS3	KO+GFP vs. KO+NDUFS3	0.0007	***
F	NDUFB8	WT vs. KO+GFP	0.0106	*
F	NDUFB8	WT vs. KO+NDUFS3	0.7189	ns
F	NDUFB8	KO+GFP vs. KO+NDUFS3	0.0431	*
G	SDHA	WT vs. KO+GFP	0.6642	ns
G	SDHA	WT vs. KO+NDUFS3	0.9069	ns
G	SDHA	KO+GFP vs. KO+NDUFS3	0.895	ns
G	UQCRC1	WT vs. KO+GFP	0.3431	ns
G	UQCRC1	WT vs. KO+NDUFS3	0.8261	ns
G	UQCRC1	KO+GFP vs. KO+NDUFS3	0.6799	ns
H	COX1	WT vs. KO+GFP	0.0333	*
H	COX1	WT vs. KO+NDUFS3	0.2482	ns
H	COX1	KO+GFP vs. KO+NDUFS3	0.5286	ns
H	VDAC	WT vs. KO+GFP	0.005	**
H	VDAC	WT vs. KO+NDUFS3	0.2804	ns
H	VDAC	KO+GFP vs. KO+NDUFS3	0.0305	*
J	CI SC	WT vs. KO+GFP	0.0007	***
J	CI SC	WT vs. KO+NDUFS3	0.0432	*
J	CI SC	KO+GFP vs. KO+NDUFS3	0.0118	*
J	CI-CIII	WT vs. KO+GFP	0.667	ns
J	CI-CIII	WT vs. KO+NDUFS3	0.0002	***
J	CI-CIII	KO+GFP vs. KO+NDUFS3	<0.0001	****
J	CIII	WT vs. KO+GFP	0.9967	ns
J	CIII	WT vs. KO+NDUFS3	0.1012	ns
J	CIII	KO+GFP vs. KO+NDUFS3	0.1166	ns
J	Total CIII	WT vs. KO+GFP	0.984	ns
J	Total CIII	WT vs. KO+NDUFS3	0.0231	*
J	Total CIII	KO+GFP vs. KO+NDUFS3	0.0161	*
J	CIV	WT vs. KO+GFP	0.2788	ns
J	CIV	WT vs. KO+NDUFS3	0.7806	ns
J	CIV	KO+GFP vs. KO+NDUFS3	0.5928	ns
L	CI Activity	WT vs. KO+GFP	0.0148	*
L	CI Activity	WT vs. KO+NDUFS3	0.2404	ns
L	CI Activity	KO+GFP vs. KO+NDUFS3	0.1344	ns
L	CIV Activity	WT vs. KO+GFP	0.7034	ns
L	CIV Activity	WT vs. KO+NDUFS3	0.7914	ns
L	CIV Activity	KO+GFP vs. KO+NDUFS3	0.3603	ns

FIGURE EV1

Panel	Label	Comparison	p value	symbol
GFAP (B)	CTX	WT vs. KO+GFP	0.0011	**
		WT vs. KO+NDUFS3	0.2184	ns
		KO+GFP vs. KO+NDUFS3	0.0059	**
	HIPP	WT vs. KO+GFP	0.0085	**
		WT vs. KO+NDUFS3	0.5863	ns
		KO+GFP vs. KO+NDUFS3	0.0262	*
Iba1 (B)	CTX	WT vs. KO+GFP	0.0436	*
		WT vs. KO+NDUFS3	0.9977	ns
		KO+GFP vs. KO+NDUFS3	0.0472	*
	HIPP	WT vs. KO+GFP	0.0032	**
		WT vs. KO+NDUFS3	0.4079	ns
		KO+GFP vs. KO+NDUFS3	0.0123	*
TUJI (B)	CTX	WT vs. KO+GFP	0.1459	ns
		WT vs. KO+NDUFS3	0.6239	ns
		KO+GFP vs. KO+NDUFS3	0.4669	ns
	HIPP	WT vs. KO+GFP	0.9983	ns
		WT vs. KO+NDUFS3	0.4804	ns
		KO+GFP vs. KO+NDUFS3	0.4528	ns

FIGURE EV2

	10M		10.25M		10.5M		10.75M		11M		11.25M		11.5M	
	p value	symbol	p value	symbol	p value	symbol	p value	symbol	p value	symbol	p value	symbol	p value	symbol
WT vs. KO+NDUFS3	0.9985	ns	0.9807	ns	0.9602	ns	0.9847	ns	0.9827	ns	0.813	ns	>0.9999	ns
WT vs. KO+COX10	0.1935	ns	0.3734	ns	0.3734	ns	0.4485	ns	0.3388	ns	0.5166	ns	0.1336	ns

	11.75M		12M		12.25M		12.5M		12.75M		13M		13.25M	
	p value	symbol	p value	symbol	p value	symbol	p value	symbol	p value	symbol	p value	symbol	p value	symbol
WT vs. KO+NDUFS3	0.9046	ns	0.9132	ns	0.9572	ns	0.9002	ns	0.7024	ns	0.8721	ns	0.9017	ns
WT vs. KO+COX10	0.1129	ns	0.221	ns	0.2973	ns	0.2175	ns	0.3153	ns	0.2139	ns	0.2374	ns

	13.5M		13.75M		14M		14.25M		14.5M		14.75M		15M	
	p value	symbol	p value	symbol	p value	symbol	p value	symbol	p value	symbol	p value	symbol	p value	symbol
WT vs. KO+NDUFS3	0.6216	ns	0.7038	ns	0.9397	ns	0.9506	ns	0.798	ns	0.8128	ns	0.9612	ns
WT vs. KO+COX10	0.253	ns	0.3005	ns	0.1138	ns	0.2626	ns	0.2223	ns	0.2487	ns	0.0958	ns

FIGURE EV3A

Panel	Label	Comparison	p value	symbol
B	10	Ndufs3-nKO+Ndufs3 vs. WT	0.8187	ns
		WT vs. COX10-nKO+COX10	0.8187	ns
	11	Ndufs3-nKO+Ndufs3 vs. WT	0.3828	ns
		WT vs. COX10-nKO+COX10	0.4979	ns
	12	Ndufs3-nKO+Ndufs3 vs. WT	0.7015	ns
		WT vs. COX10-nKO+COX10	0.7246	ns
	13	Ndufs3-nKO+Ndufs3 vs. WT	0.8986	ns
		WT vs. COX10-nKO+COX10	0.7252	ns
	14	Ndufs3-nKO+Ndufs3 vs. WT	0.6632	ns
		WT vs. COX10-nKO+COX10	0.8807	ns
	15	Ndufs3-nKO+Ndufs3 vs. WT	0.6546	ns
		WT vs. COX10-nKO+COX10	0.4403	ns
C	10	Ndufs3-nKO+Ndufs3 vs. WT	0.1614	ns
		WT vs. COX10-nKO+COX10	0.2049	ns
	11	Ndufs3-nKO+Ndufs3 vs. WT	0.0242	*
		WT vs. COX10-nKO+COX10	0.6191	ns
	12	Ndufs3-nKO+Ndufs3 vs. WT	0.0747	ns
		WT vs. COX10-nKO+COX10	0.7734	ns
	13	Ndufs3-nKO+Ndufs3 vs. WT	0.0283	*
		WT vs. COX10-nKO+COX10	0.9488	ns
	14	Ndufs3-nKO+Ndufs3 vs. WT	0.0676	ns
		WT vs. COX10-nKO+COX10	0.9907	ns
	15	Ndufs3-nKO+Ndufs3 vs. WT	0.0158	*
		WT vs. COX10-nKO+COX10	0.646	ns
E	NDUFS3 (CTX)	Ndufs3-nKO+Ndufs3 vs. WT	>0.9999	ns
		WT vs. COX10-nKO+COX10	0.8974	ns
	NDUFS3 (HIPP)	Ndufs3-nKO+Ndufs3 vs. WT	0.2914	ns
		WT vs. COX10-nKO+COX10	0.1646	ns
	COX1 (CTX)	Ndufs3-nKO+Ndufs3 vs. WT	0.6549	ns
		WT vs. COX10-nKO+COX10	0.9635	ns
	COX1 (HIPP)	Ndufs3-nKO+Ndufs3 vs. WT	0.6	ns
		WT vs. COX10-nKO+COX10	0.9721	ns
G	CI SC	Ndufs3-nKO+Ndufs3 vs. WT	0.6412	ns
		WT vs. COX10-nKO+COX10	0.2759	ns
	CIV	Ndufs3-nKO+Ndufs3 vs. WT	0.796	ns
		WT vs. COX10-nKO+COX10	0.7417	ns
I	CI Activity	Ndufs3-nKO+Ndufs3 vs. WT	0.8577	ns
		WT vs. COX10-nKO+COX10	0.2881	ns
	CIV Activity	Ndufs3-nKO+Ndufs3 vs. WT	0.1938	ns
		WT vs. COX10-nKO+COX10	0.9518	ns

FIGURE EV3B-I

Panel	Label	Comparison	p value	symbol
GFAP (D)	CTX	WT vs. Ndufs3-nKO+Ndufs3	0.2386	ns
		WT vs. COX10-nKO+COX10	0.8915	ns
	HIPP	WT vs. Ndufs3-nKO+Ndufs3	0.6757	ns
		WT vs. COX10-nKO+COX10	0.9771	ns
Iba1 (D)	CTX	WT vs. Ndufs3-nKO+Ndufs3	0.841	ns
		WT vs. COX10-nKO+COX10	0.6233	ns
	HIPP	WT vs. Ndufs3-nKO+Ndufs3	0.8	ns
		WT vs. COX10-nKO+COX10	0.0327	*
TUJI (D)	CTX	WT vs. Ndufs3-nKO+Ndufs3	0.4812	ns
		WT vs. COX10-nKO+COX10	0.9569	ns
	HIPP	WT vs. Ndufs3-nKO+Ndufs3	0.7032	ns
		WT vs. COX10-nKO+COX10	0.4235	ns
E	brain weight	WT vs. Ndufs3-nKO+Ndufs3	0.1333	ns
		WT vs. COX10-nKO+COX10	0.1991	ns

FIGURE EV4

Panel	Label	p value	symbol
A	2	0.2672	ns
	2.5	0.2672	ns
	3	0.0561	ns
	3.5	0.1049	ns
	4	0.1049	ns
	4.5	0.2203	ns
	5	0.2672	ns
B	2.5	0.8212	ns
	3.5	>0.9999	ns
	4	0.8212	ns
	4.5	>0.9999	ns
	5	0.8212	ns
C	2.5	0.9843	ns
	3.5	0.9492	ns
	4	0.9670	ns
	4.5	0.8393	ns
	5	0.8393	ns
G	NDUFS3	0.1762	ns
	NDUFB	0.1762	ns
H	SDHA	0.8724	ns
	UQCRC1	0.8724	ns
	COX1	0.1634	ns
I	VDAC	0.3148	ns
K	NDUFS3	0.4129	ns
	NDUFB	0.4129	ns
L	SDHA	0.4282	ns
	UQCRC1	0.3553	ns
	COX1	0.3553	ns
M	VDAC	0.5416	ns
O	GFAP (CTX)	0.0292	*
	GFAP (HPP)	0.0017	**
	IBA1 (CTX)	0.0398	*
	IBA1 (HPP)	0.0398	*
	TUJI (CTX)	0.9014	ns
	TUJI (HPP)	0.9014	ns

FIGURE EV5

Gene	Primer	Size
CaMKIIα-Cre	F: 5′-GCGGTCTGGCAGTAAAAACTATC-3′	287bp
	R: 5′-GTGAAACAGCATTGCTGTCACTT-3′	
NDUFS3	F: 5′-GCAAAGATTGCCCAACACAG-3′	W = 333bp
	R: 5′-CTCCAAGCCCTCCCTGAAG-3′	F = 473bp
	R2: 5’-TGCCCTTTCTCACCTTTAGTCC-3’	D = 408bp
COX10	F: 5′-ACCATTAGAAACTGCTGATGGCT-3′	W = 700bp
	R: 5′-CACTGACGCAGCGCCAGCATCTT-3′	F = 800bp

Appendix Table S2: Genotyping primers

Target	Forward Primer (5'-3')	Reverse Primer (5'-3')
ND1	GCCTGACCCATAGCCATAAT	CGGCTGCGTATTCTACGTTA
	Probe: 56-FAM/TCTCAACCC/ZEN/TAGCAGAAACAAACCGG/31ABkFQ	
18S	GCCCGAAGCGTTTACTTTG	CCGCGGTCCTATTCCATTAT
	Probe: 5CY5/TTAGAGTGTTCAAAGCAGG	
hSYN	GTGTGTCTAGACTGCAGAGG	GGGTCGGTCGTCAGGTA
	Probe: 56-FAM/CCTGCGTAT/ZEN/GAGTGCAAGTGGGTT/31ABkFQ	
TTR	GACAGGATGGCTTCCCTTC	GTCTAACTGCCATGTCTGGAT
	Probe: 5Cy5/CCTCGCTGG/TAO/ACTGGTATTTGTGTCT/3IAbRQSp	

Appendix Table S3: dPCR primer/probe sets
