# Supplementary figures and images for: Restoration of defective oxidative phosphorylation to a subset of neurons prevents mitochondrial encephalopathy (part 1 of 2)
Source: EMBO Mol Med. 2024 Aug 21;16(9):13. doi: 10.1038/s44321-024-00111-4 (PMC11392956; doi:10.1038/s44321-024-00111-4)

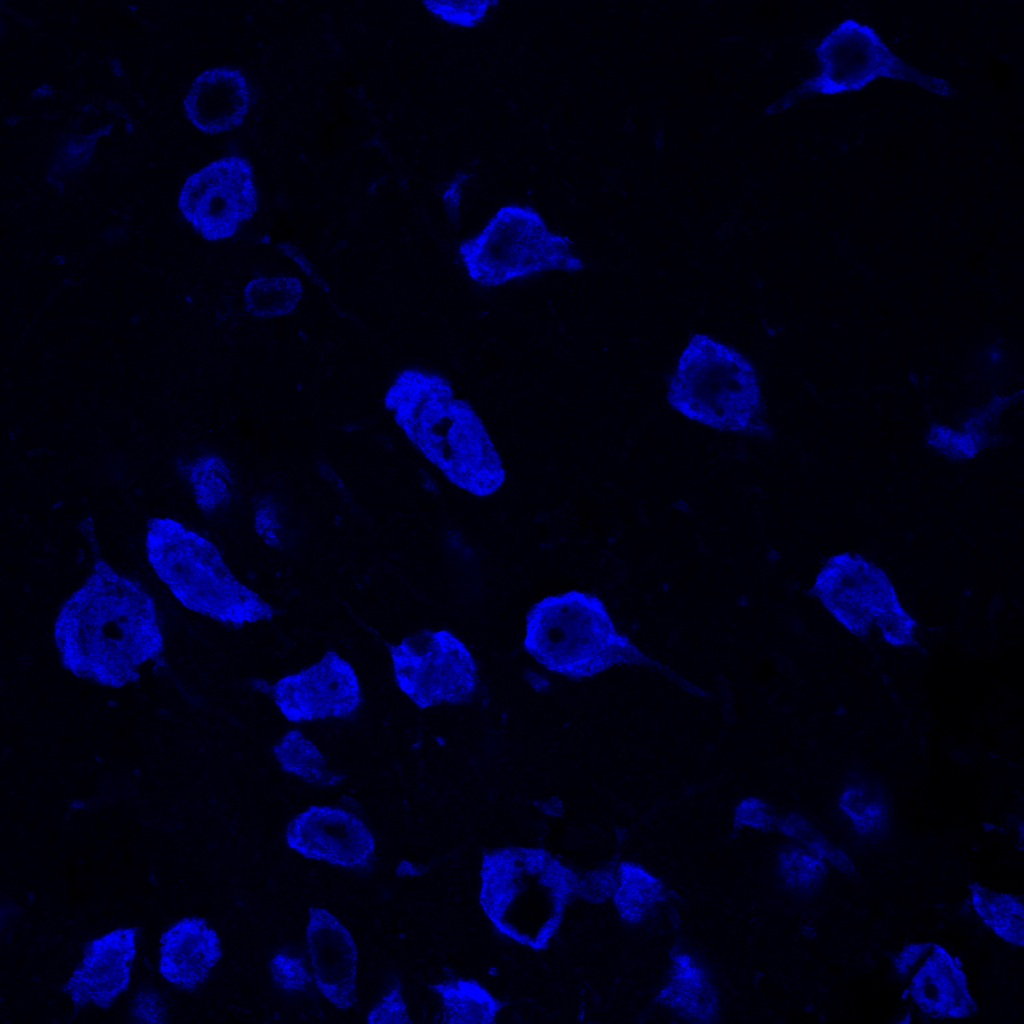

Supplement: Supplementary file 4 — Source data Fig. 3 [file 44321_2024_111_MOESM4_ESM.zip › EMM-2024-19843_SourceData-Figure3/3J/NeuN IHC - WT.tiff]

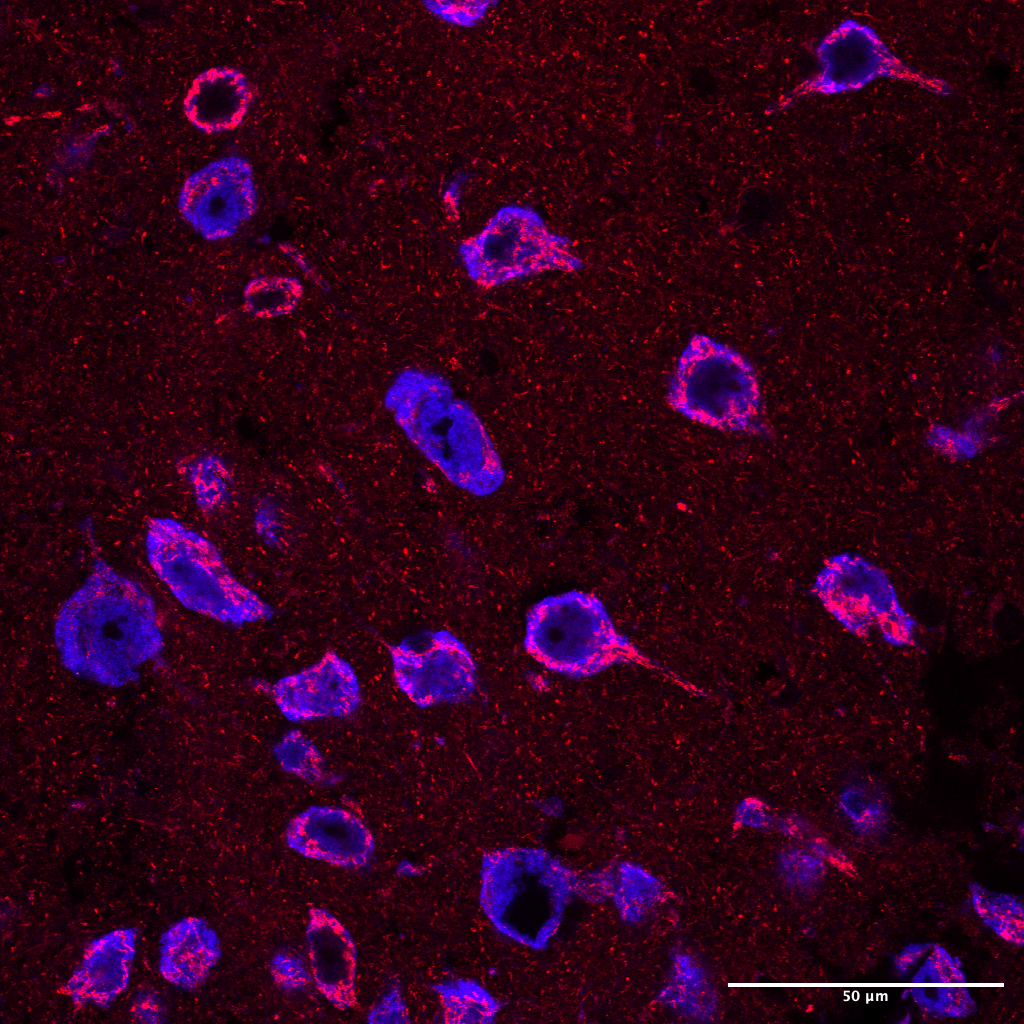

Supplement: Supplementary file 4 — Source data Fig. 3 [file 44321_2024_111_MOESM4_ESM.zip › EMM-2024-19843_SourceData-Figure3/3J/NDUFS3 NeuN IHC - WT.tiff]

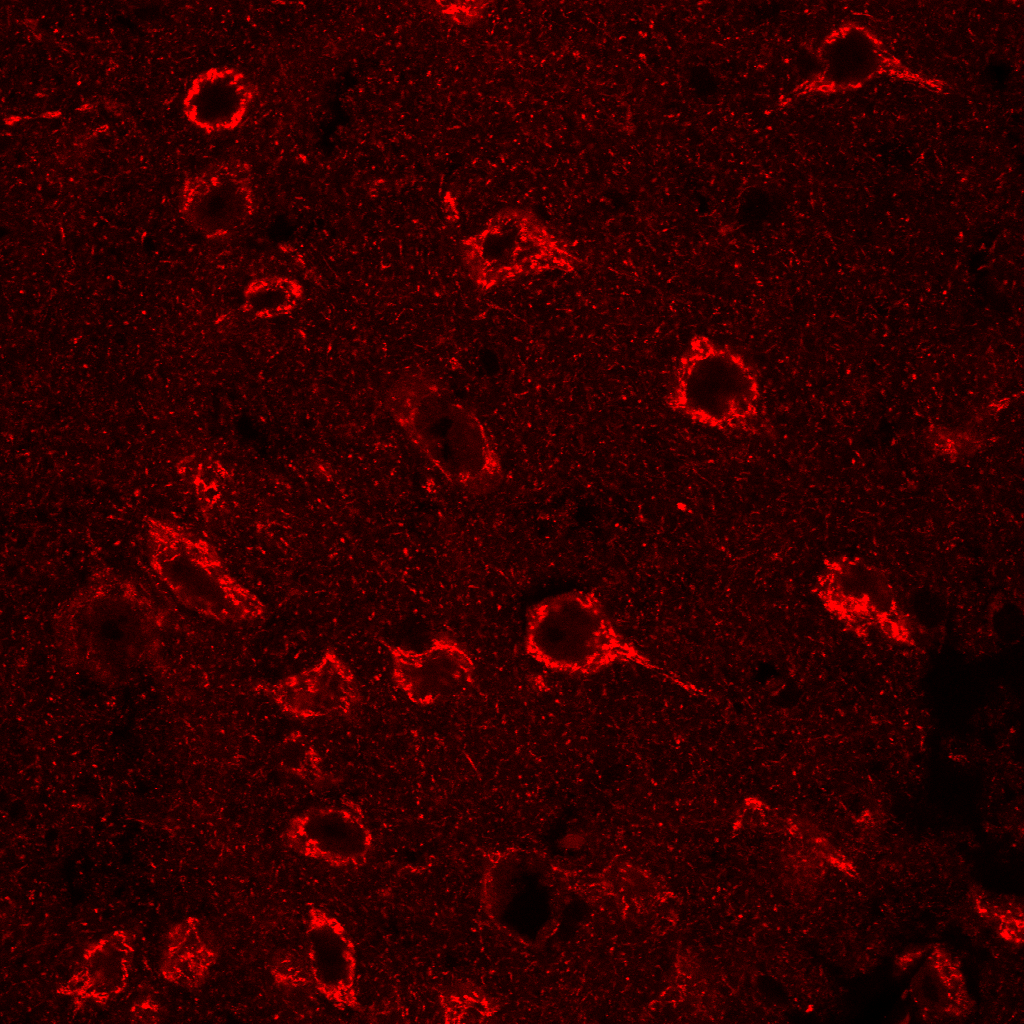

Supplement: Supplementary file 4 — Source data Fig. 3 [file 44321_2024_111_MOESM4_ESM.zip › EMM-2024-19843_SourceData-Figure3/3J/NDUFS3 IHC - WT.tiff]

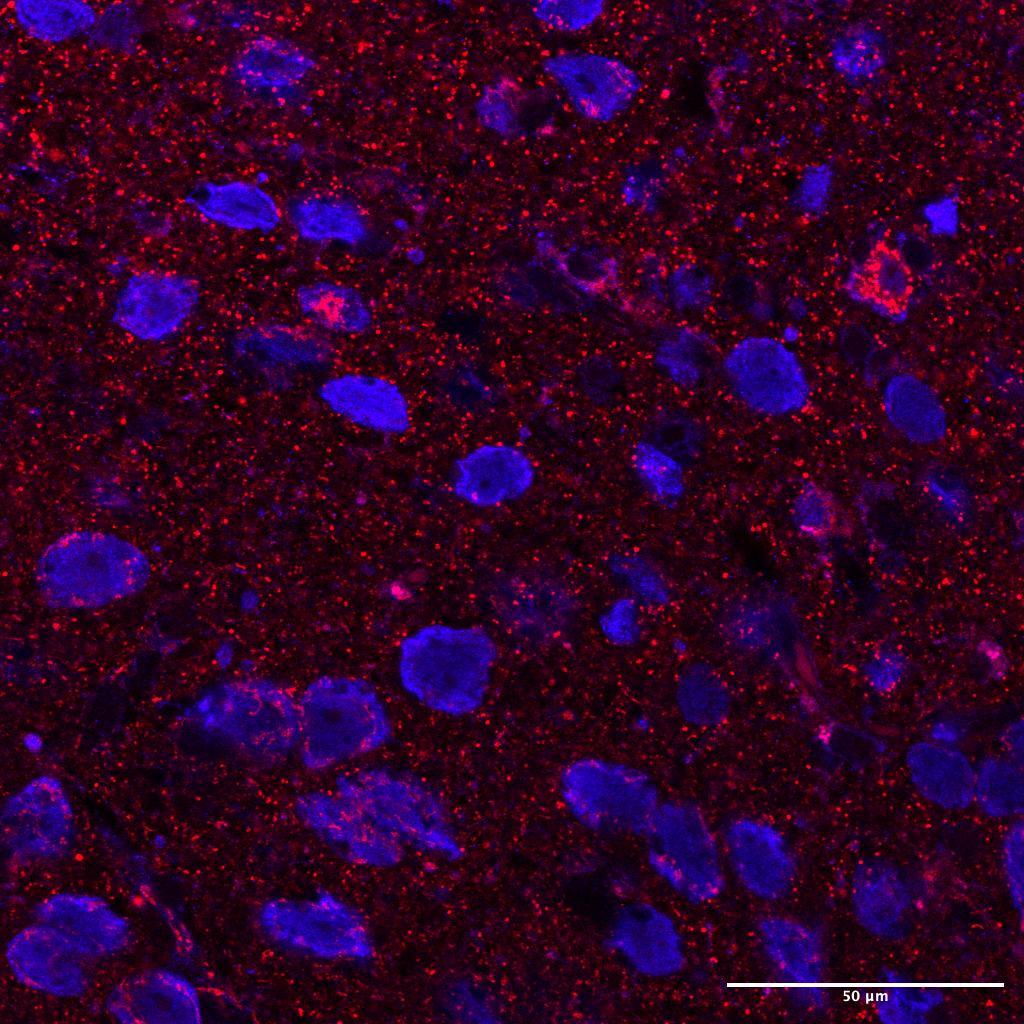

Supplement: Supplementary file 4 — Source data Fig. 3 [file 44321_2024_111_MOESM4_ESM.zip › EMM-2024-19843_SourceData-Figure3/3J/NDUFS3 NeuN IHC - KO+GFP.tiff]

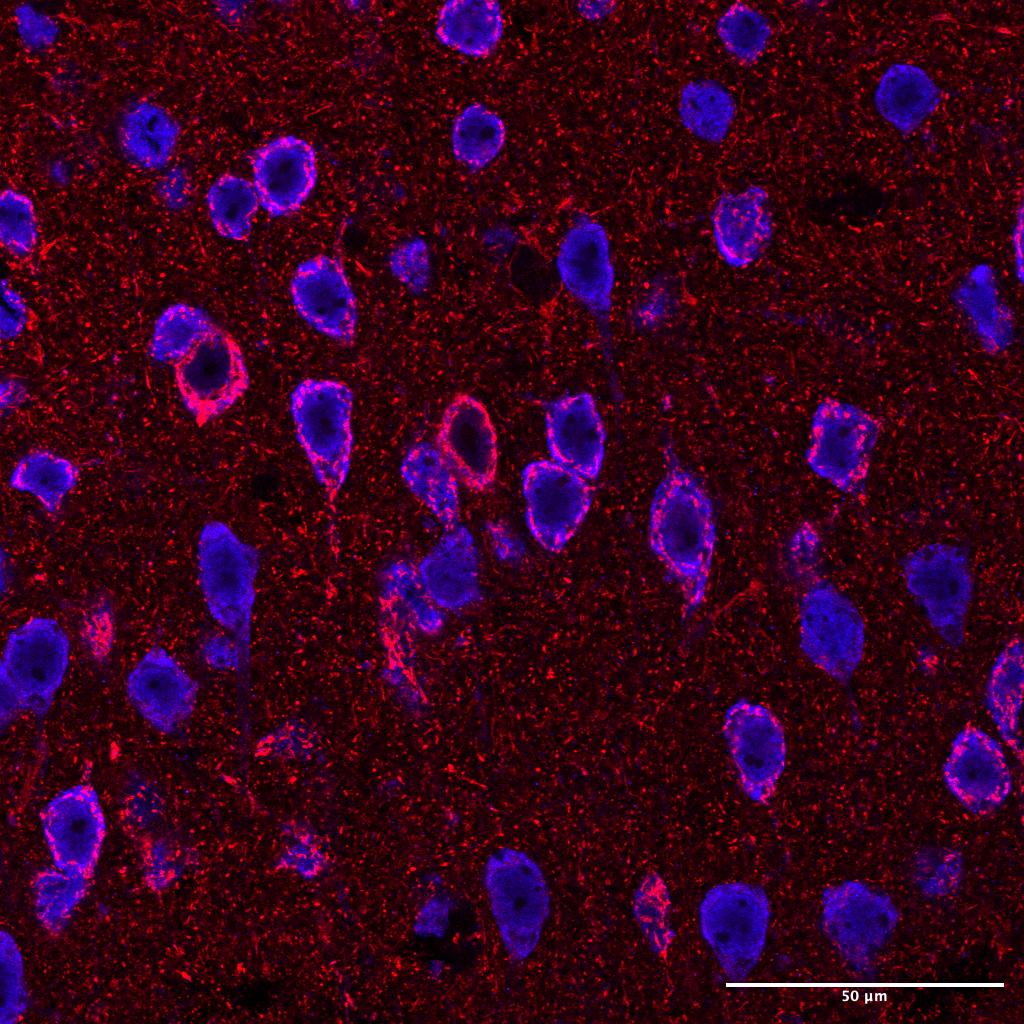

Supplement: Supplementary file 4 — Source data Fig. 3 [file 44321_2024_111_MOESM4_ESM.zip › EMM-2024-19843_SourceData-Figure3/3J/NDUFS3 NeuN IHC - KO+NDUFS3.tiff]

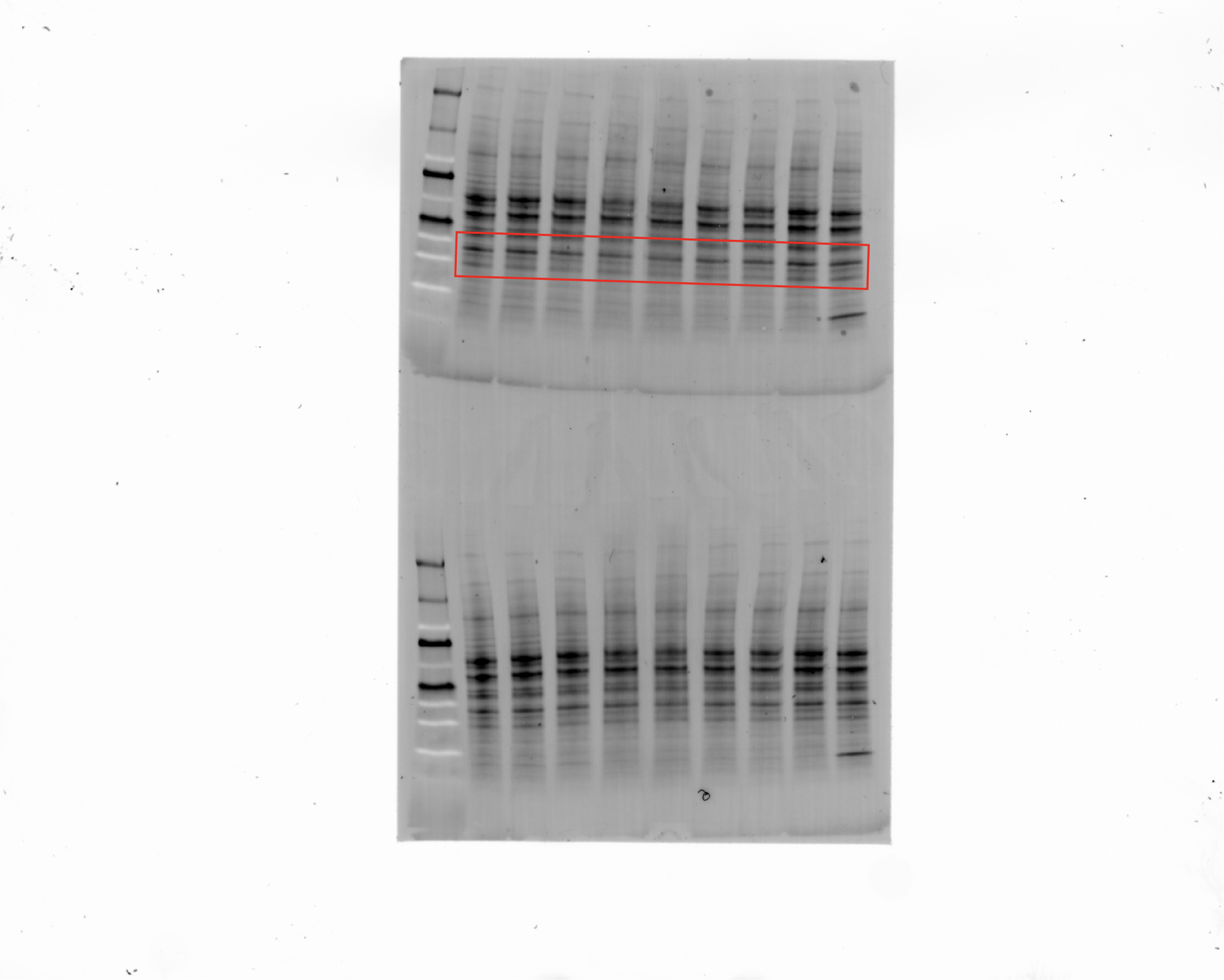

Supplement: Supplementary file 4 — Source data Fig. 3 [file 44321_2024_111_MOESM4_ESM.zip › EMM-2024-19843_SourceData-Figure3/3A/western Total Protein.tif]

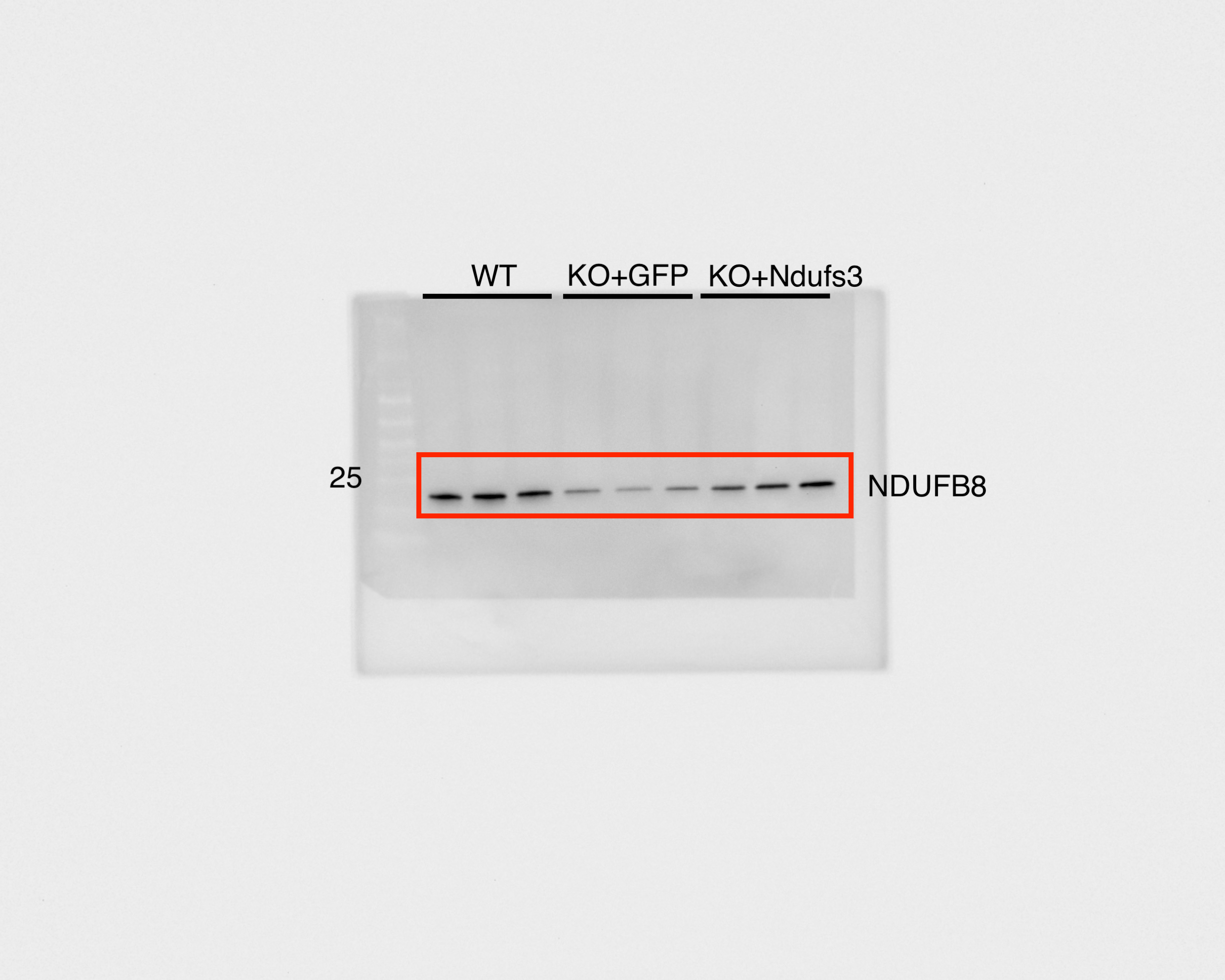

Supplement: Supplementary file 4 — Source data Fig. 3 [file 44321_2024_111_MOESM4_ESM.zip › EMM-2024-19843_SourceData-Figure3/3A/western NDUFB8.tiff]

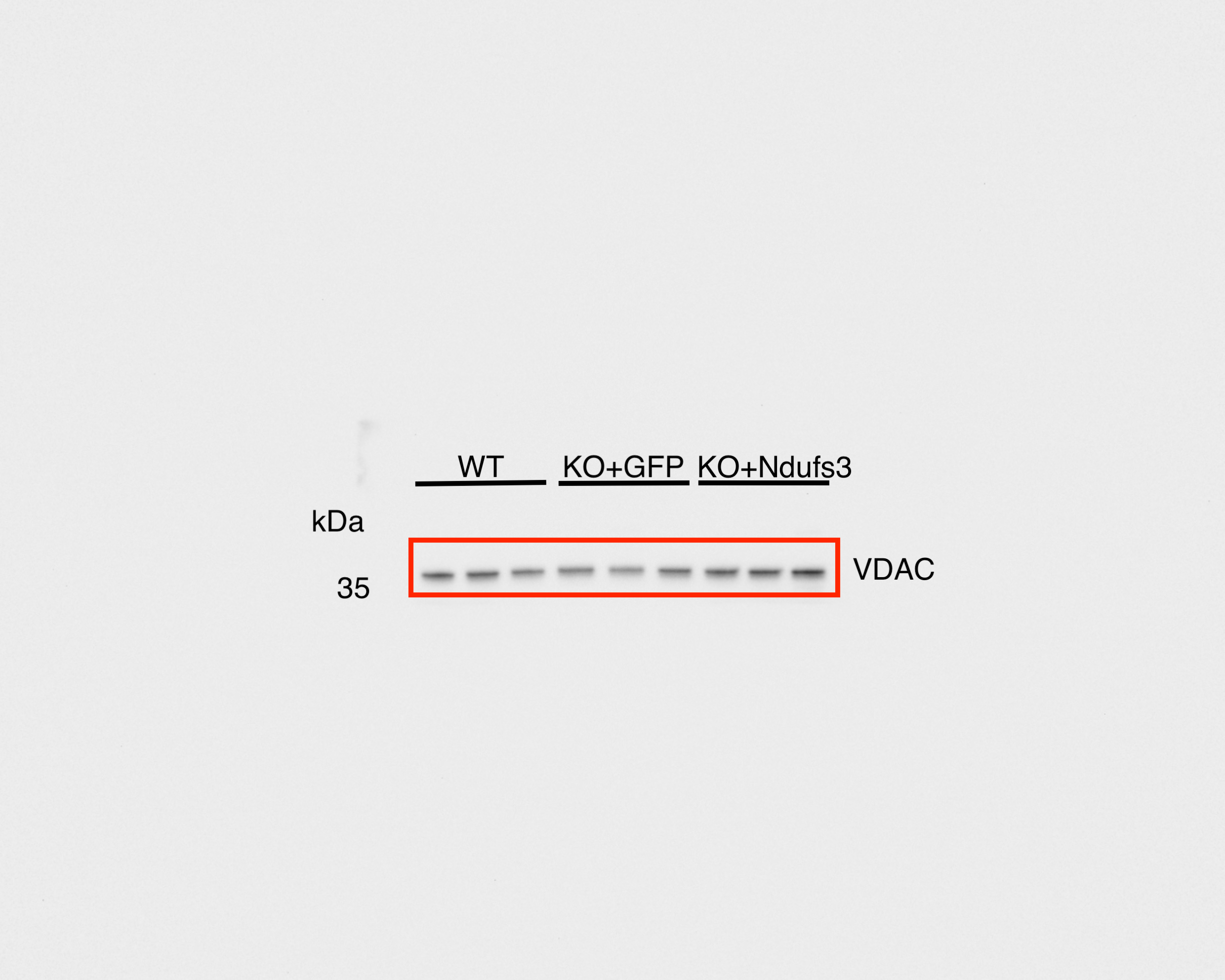

Supplement: Supplementary file 4 — Source data Fig. 3 [file 44321_2024_111_MOESM4_ESM.zip › EMM-2024-19843_SourceData-Figure3/3A/western VDAC.tif]

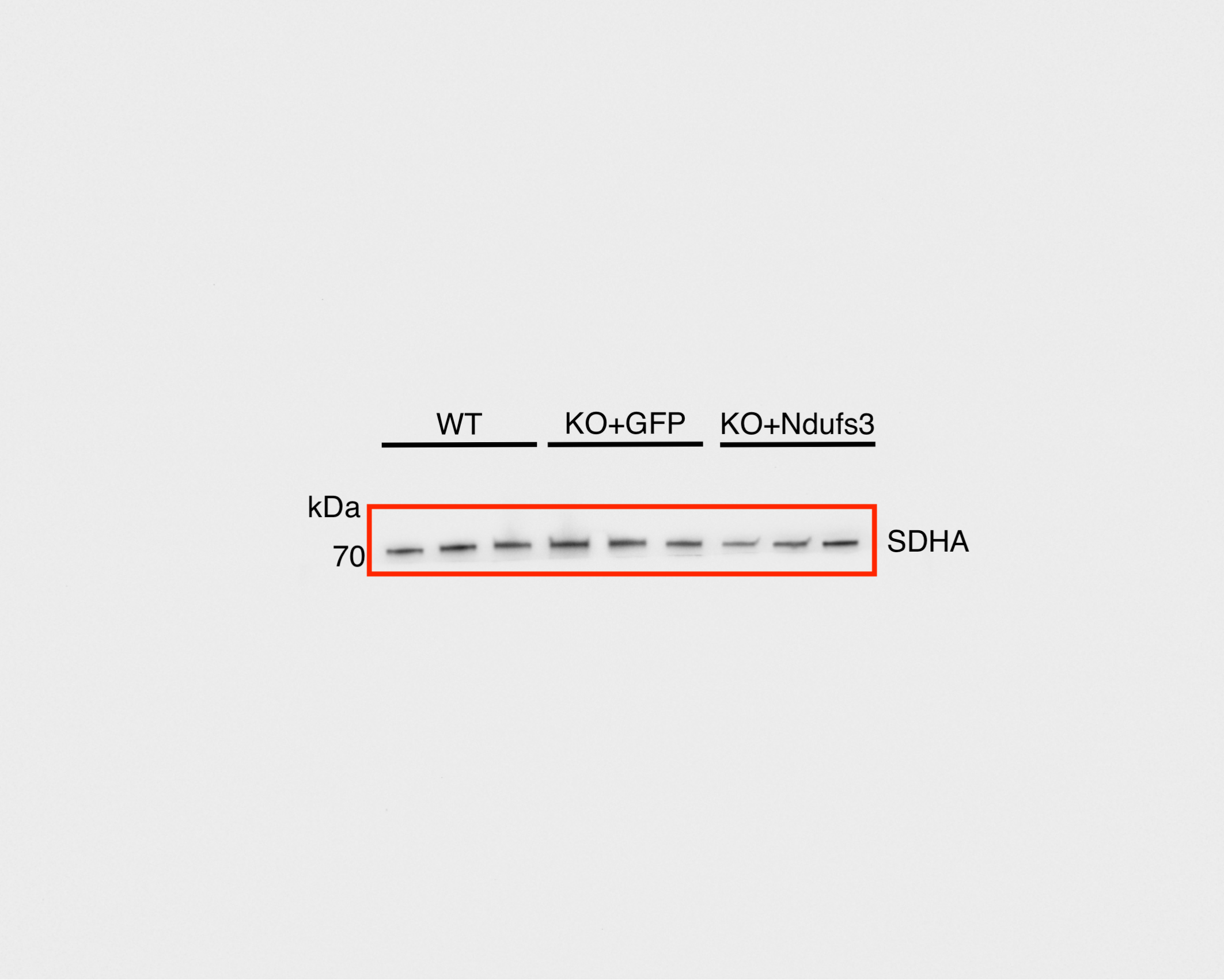

Supplement: Supplementary file 4 — Source data Fig. 3 [file 44321_2024_111_MOESM4_ESM.zip › EMM-2024-19843_SourceData-Figure3/3A/western SDHA.png]

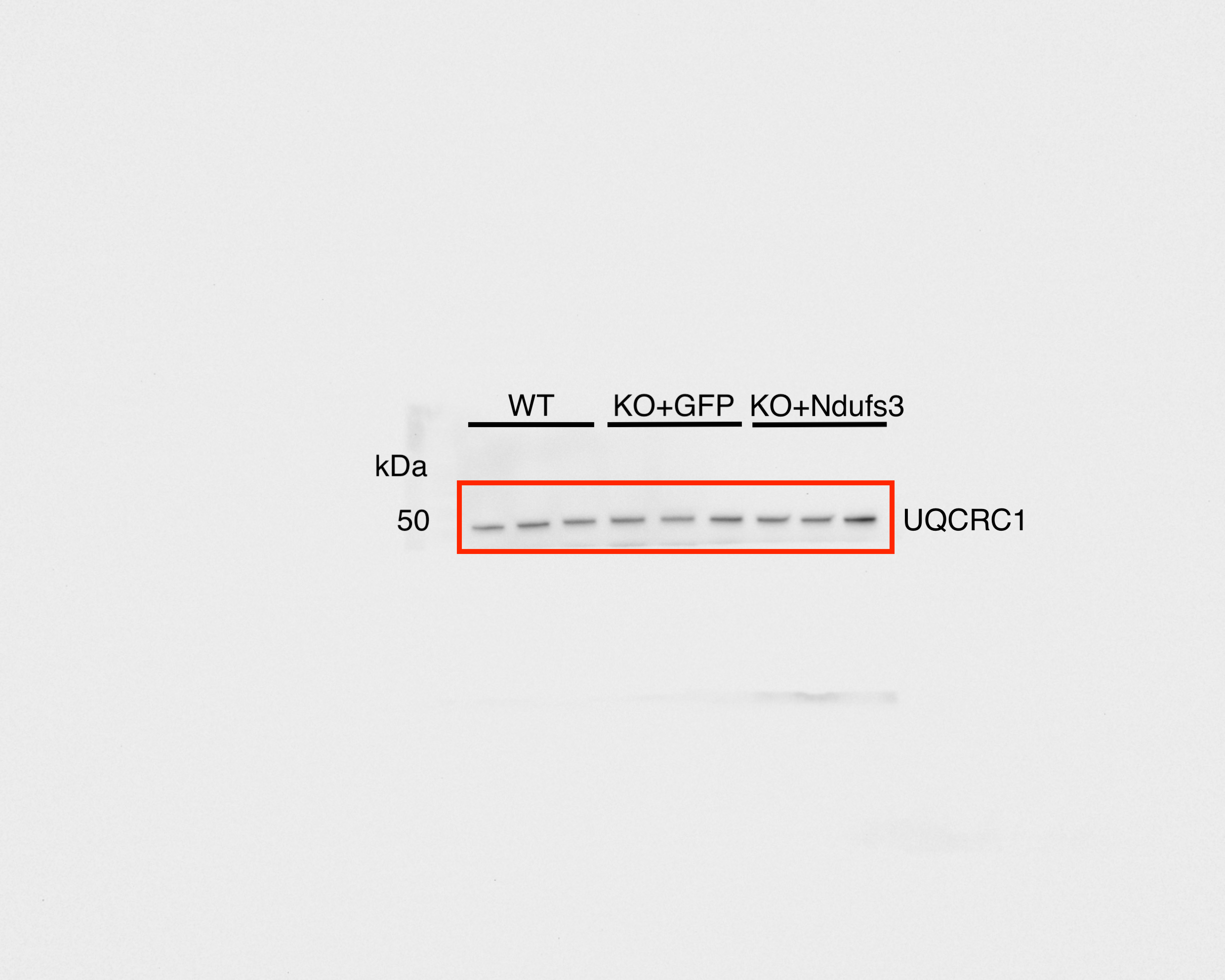

Supplement: Supplementary file 4 — Source data Fig. 3 [file 44321_2024_111_MOESM4_ESM.zip › EMM-2024-19843_SourceData-Figure3/3A/western UQCRC1.png]

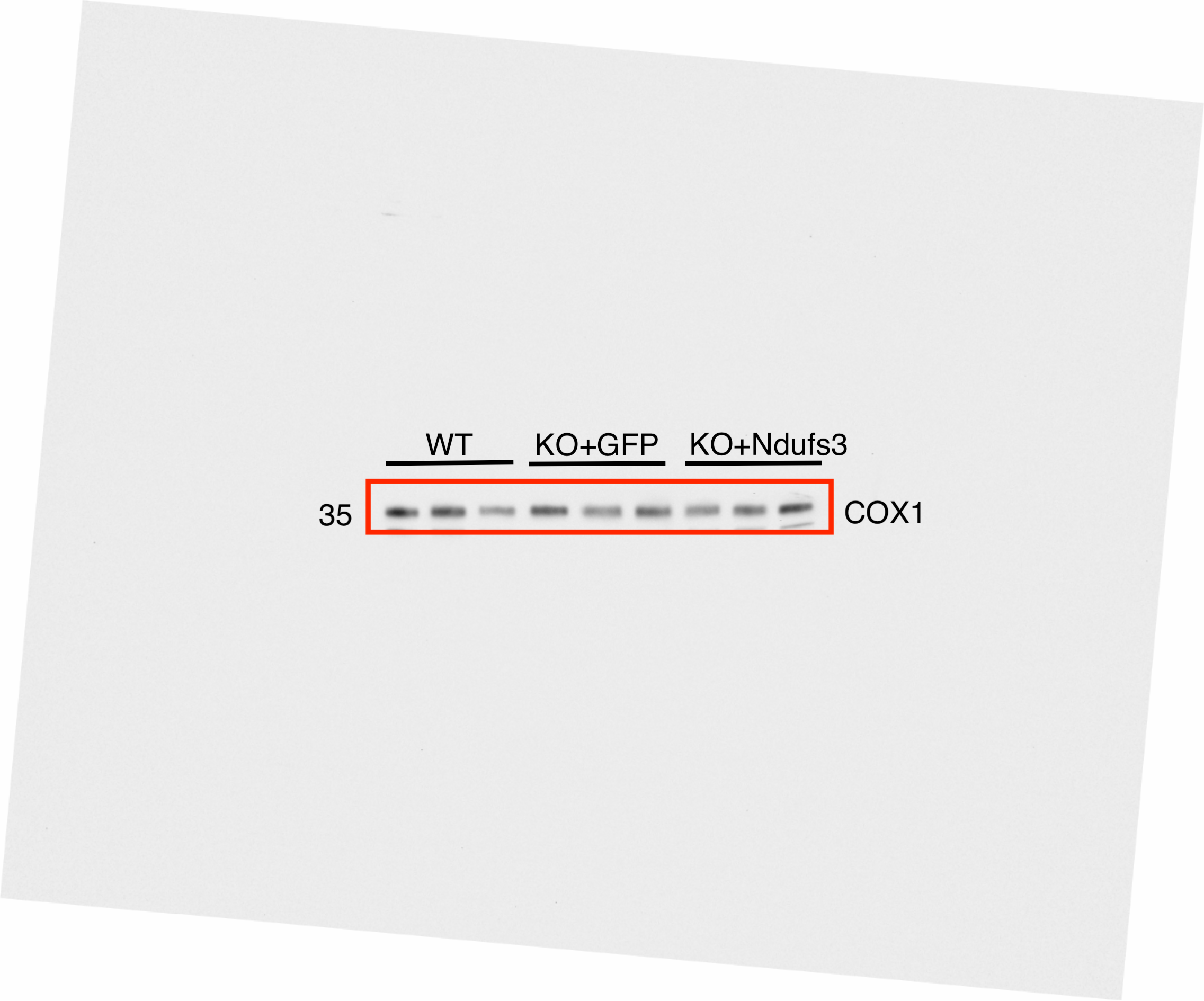

Supplement: Supplementary file 4 — Source data Fig. 3 [file 44321_2024_111_MOESM4_ESM.zip › EMM-2024-19843_SourceData-Figure3/3A/western COXI.tiff]

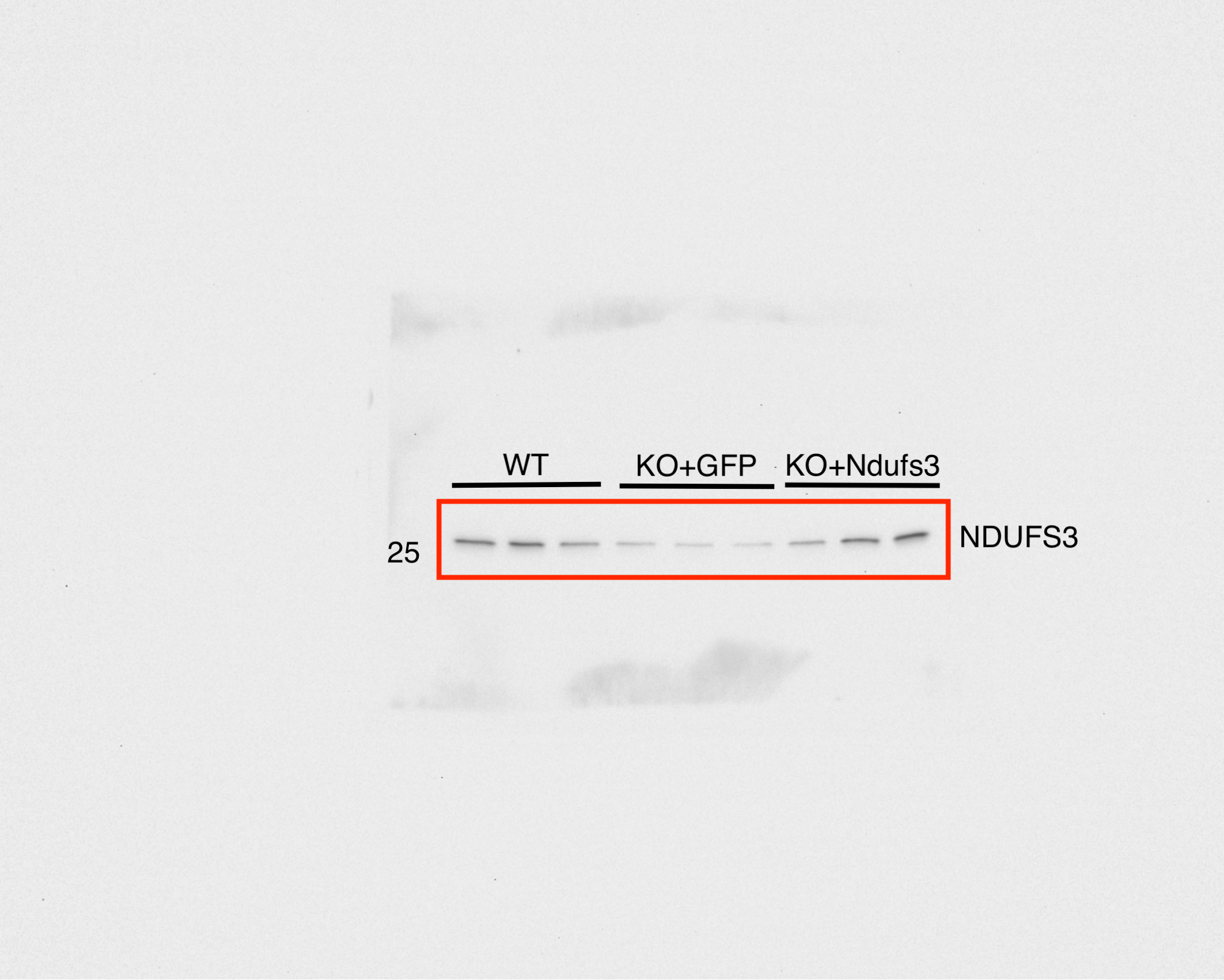

Supplement: Supplementary file 4 — Source data Fig. 3 [file 44321_2024_111_MOESM4_ESM.zip › EMM-2024-19843_SourceData-Figure3/3E/western NDUFS3.tiff]

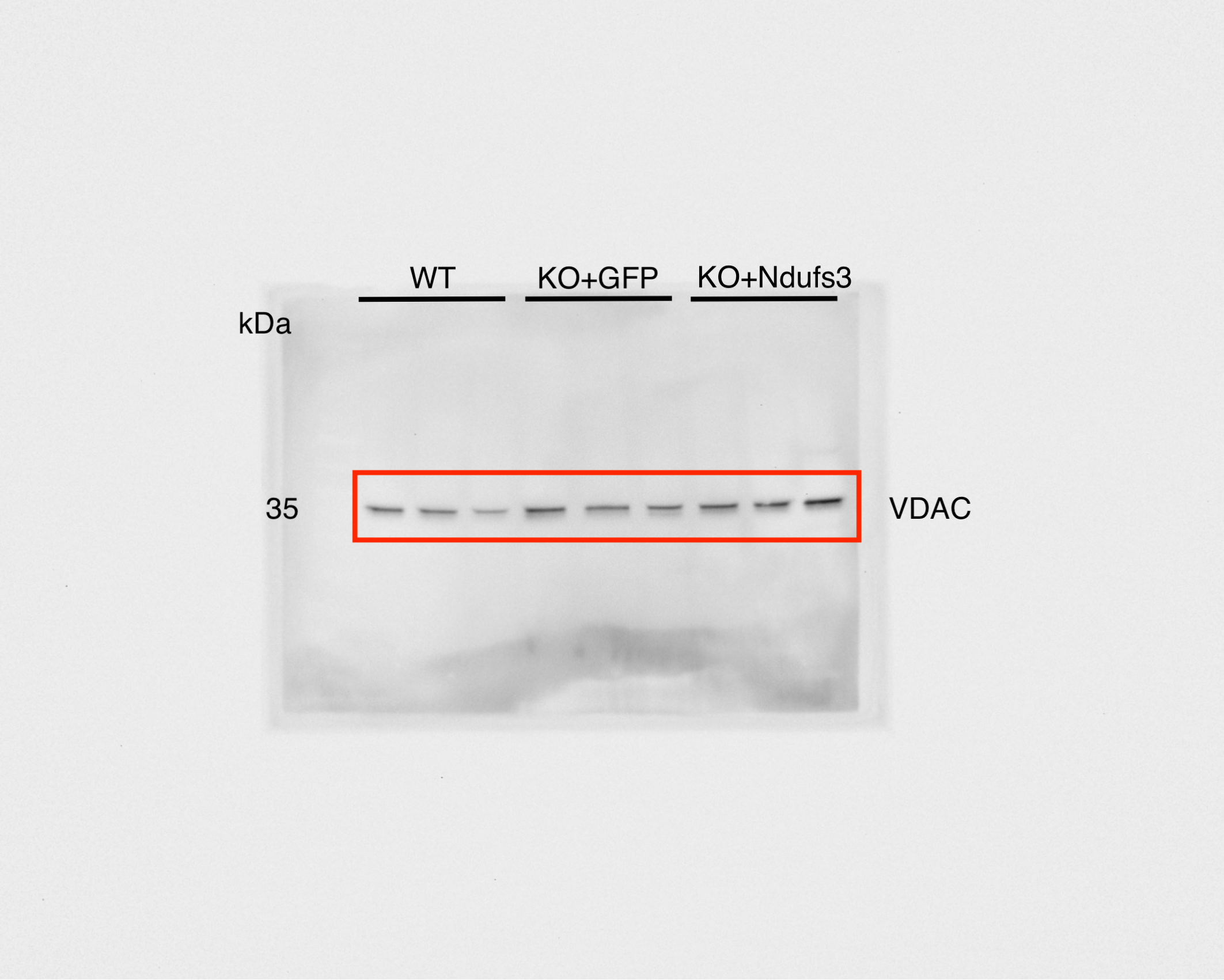

Supplement: Supplementary file 4 — Source data Fig. 3 [file 44321_2024_111_MOESM4_ESM.zip › EMM-2024-19843_SourceData-Figure3/3E/western VDAC.tiff]

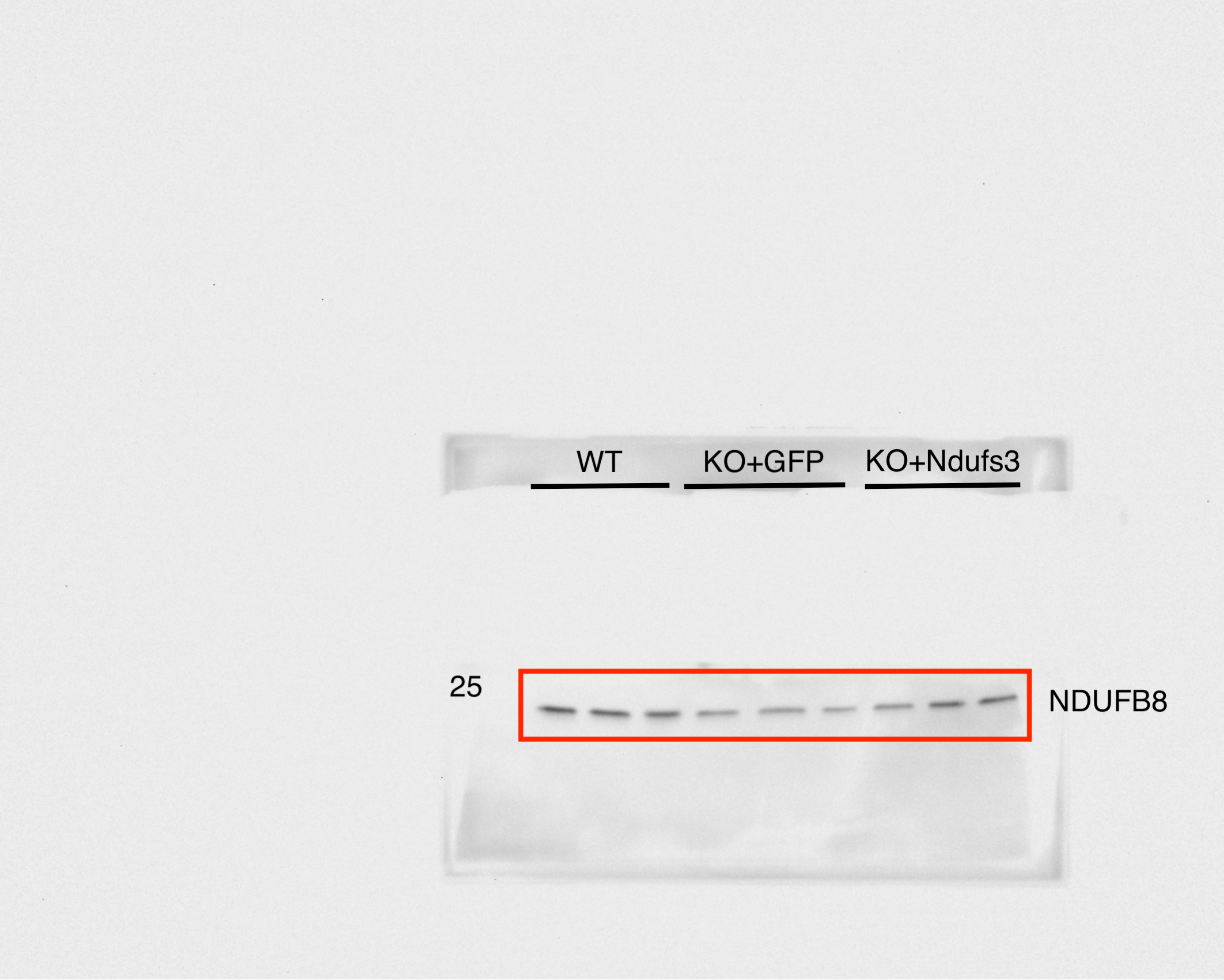

Supplement: Supplementary file 4 — Source data Fig. 3 [file 44321_2024_111_MOESM4_ESM.zip › EMM-2024-19843_SourceData-Figure3/3E/western NDUFB8.tiff]

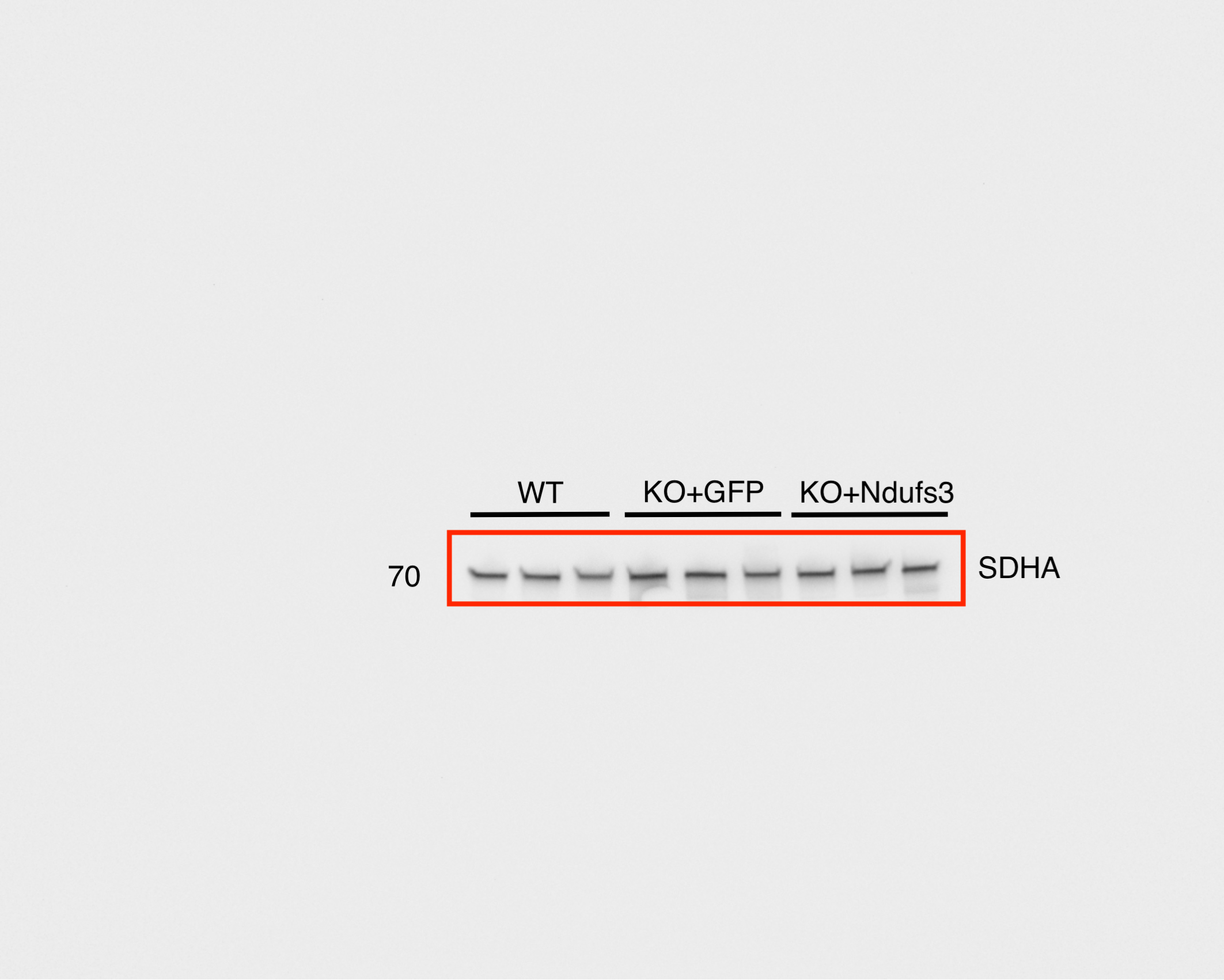

Supplement: Supplementary file 4 — Source data Fig. 3 [file 44321_2024_111_MOESM4_ESM.zip › EMM-2024-19843_SourceData-Figure3/3E/western SDHA.tiff]

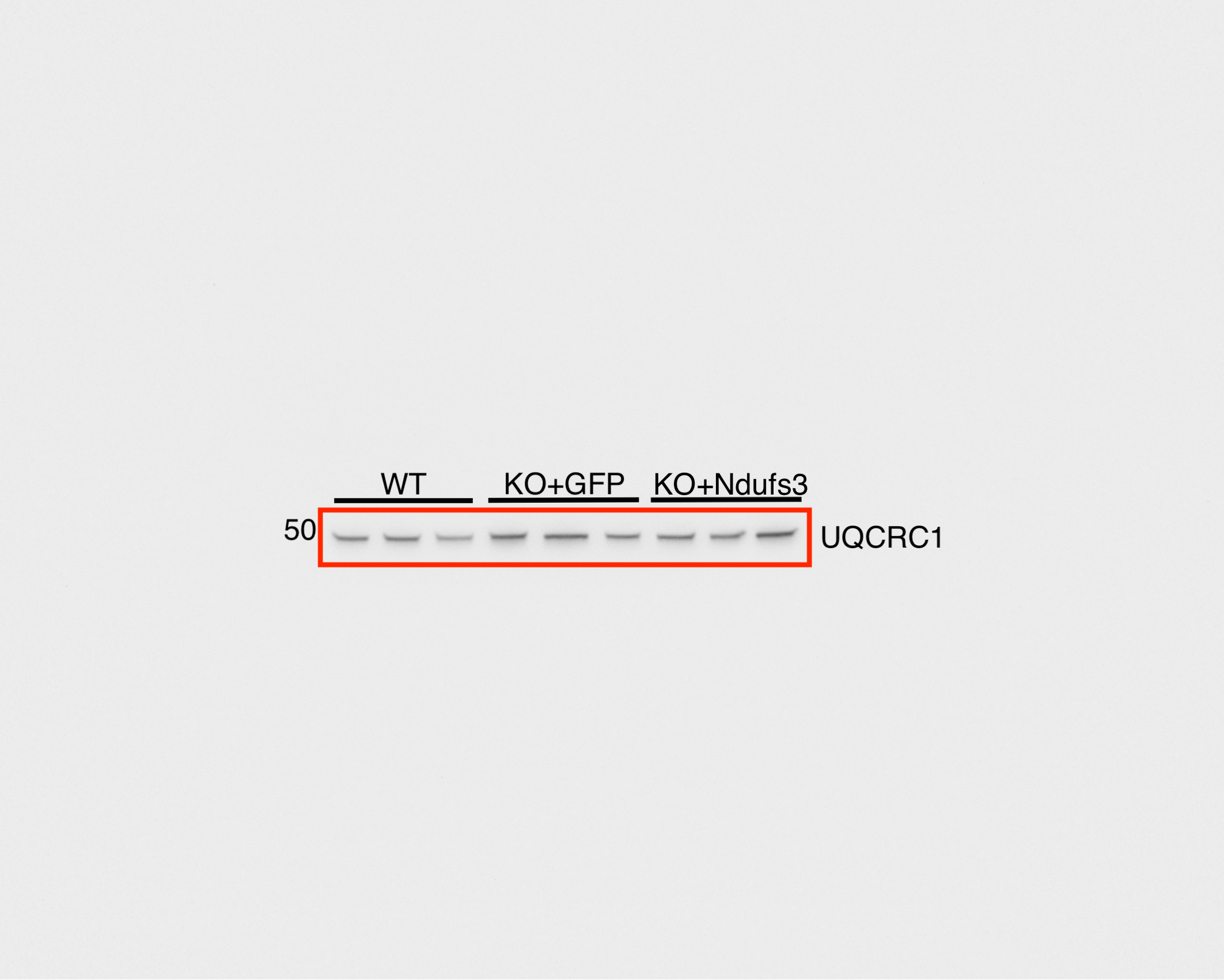

Supplement: Supplementary file 4 — Source data Fig. 3 [file 44321_2024_111_MOESM4_ESM.zip › EMM-2024-19843_SourceData-Figure3/3E/western UQCRC1.tiff]

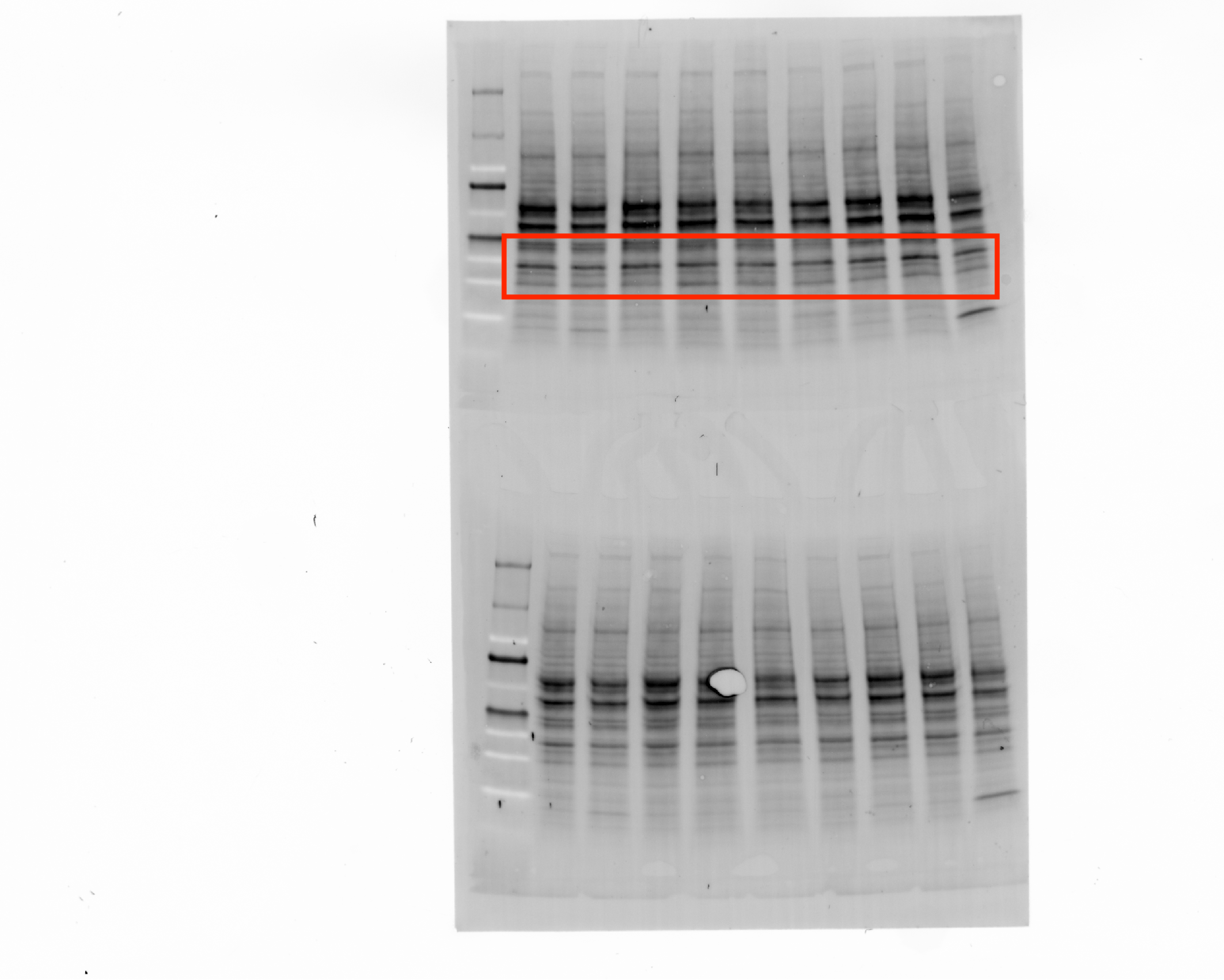

Supplement: Supplementary file 4 — Source data Fig. 3 [file 44321_2024_111_MOESM4_ESM.zip › EMM-2024-19843_SourceData-Figure3/3E/western Total Protein.tiff]

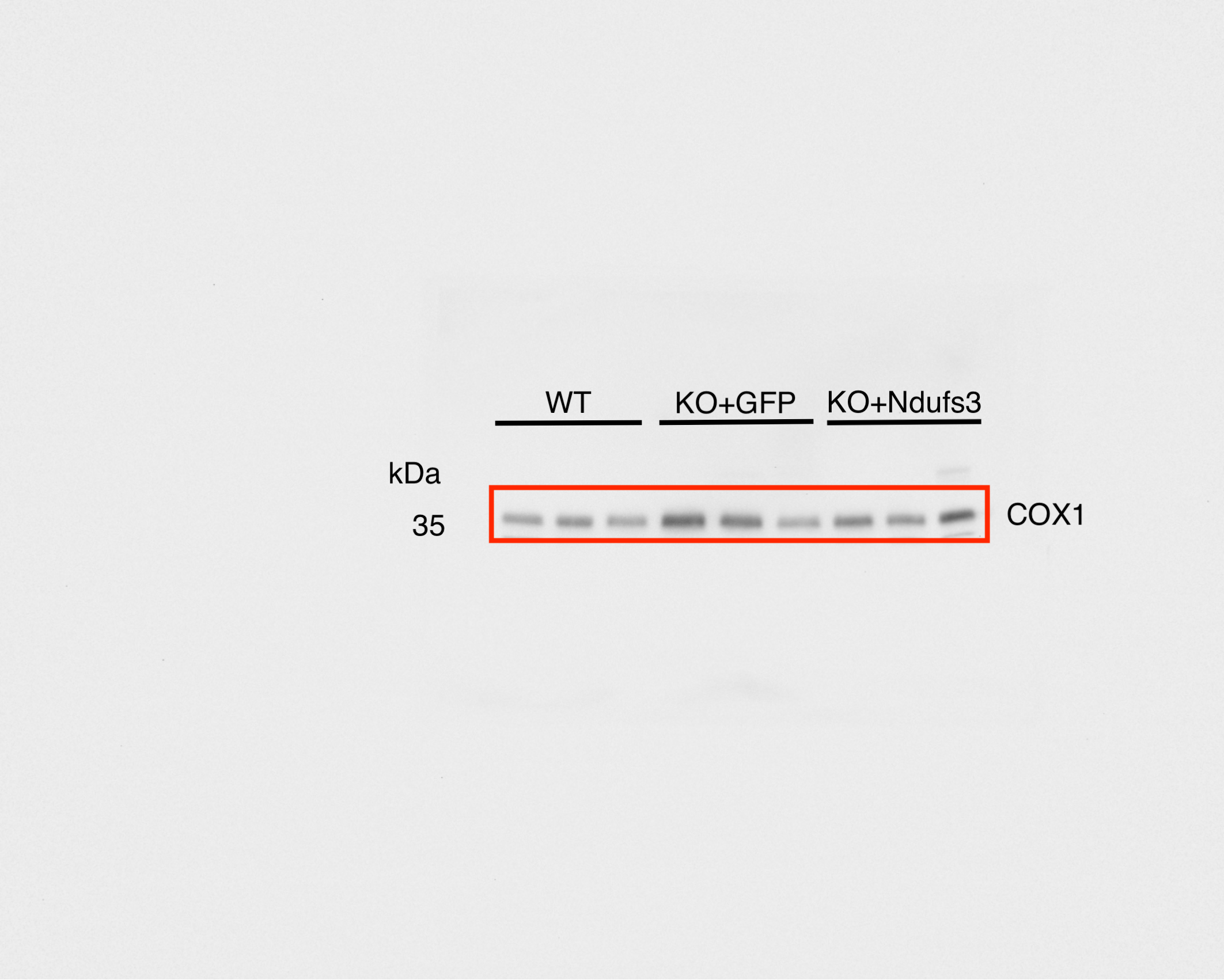

Supplement: Supplementary file 4 — Source data Fig. 3 [file 44321_2024_111_MOESM4_ESM.zip › EMM-2024-19843_SourceData-Figure3/3E/western COXI.tiff]

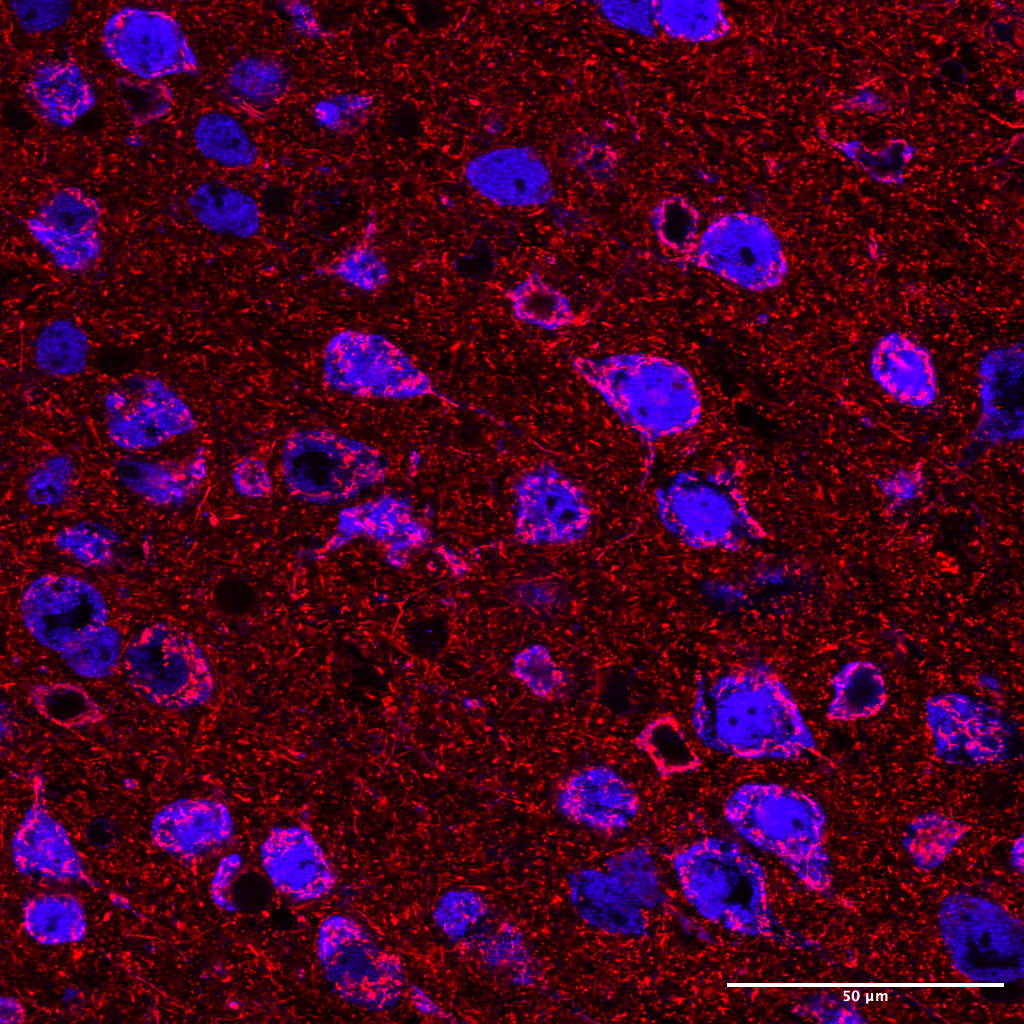

Supplement: Supplementary file 5 — Source data Fig. 4 [file 44321_2024_111_MOESM5_ESM.zip › EMM-2024-19843_SourceData-Figure4/4J/COX1 NeuN IHC - KO+COX10.tiff]

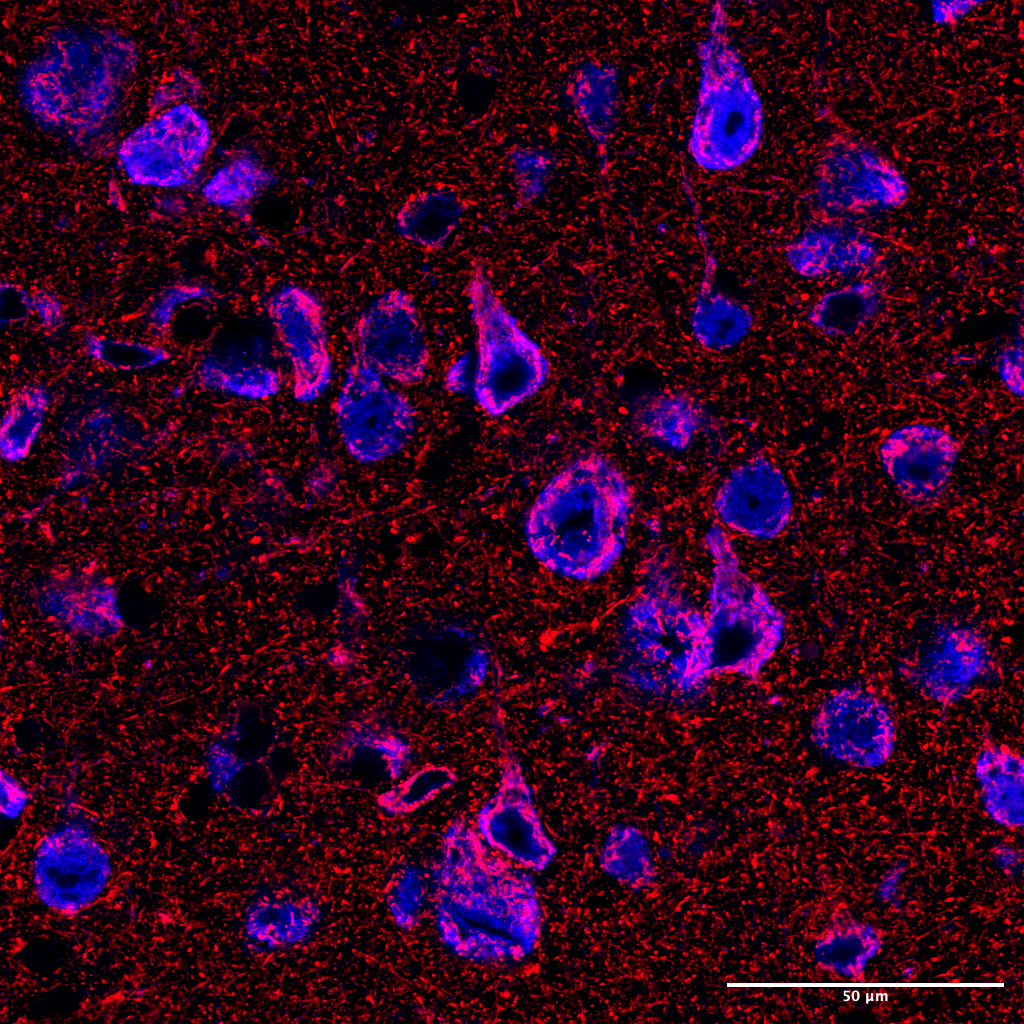

Supplement: Supplementary file 5 — Source data Fig. 4 [file 44321_2024_111_MOESM5_ESM.zip › EMM-2024-19843_SourceData-Figure4/4J/COX1 NeuN IHC - WT.tiff]

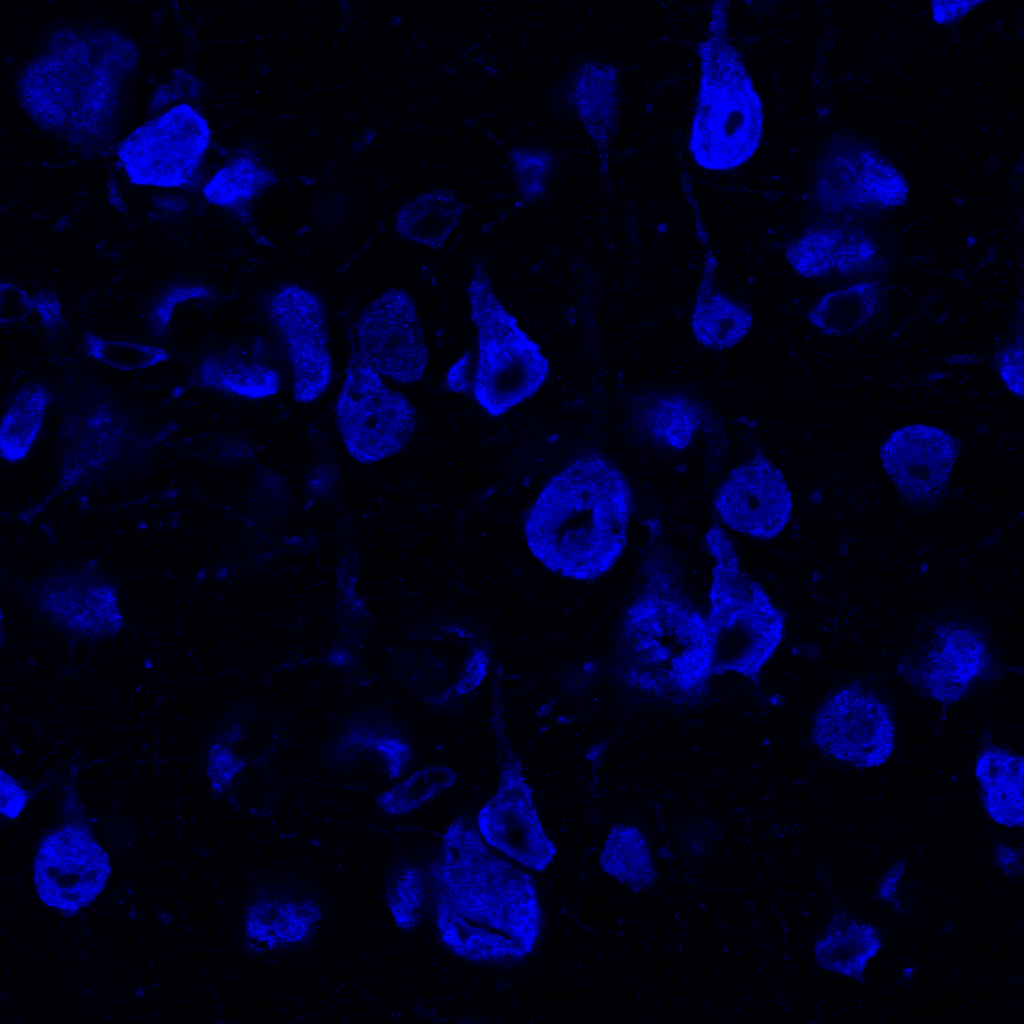

Supplement: Supplementary file 5 — Source data Fig. 4 [file 44321_2024_111_MOESM5_ESM.zip › EMM-2024-19843_SourceData-Figure4/4J/NeuN IHC - WT.tiff]

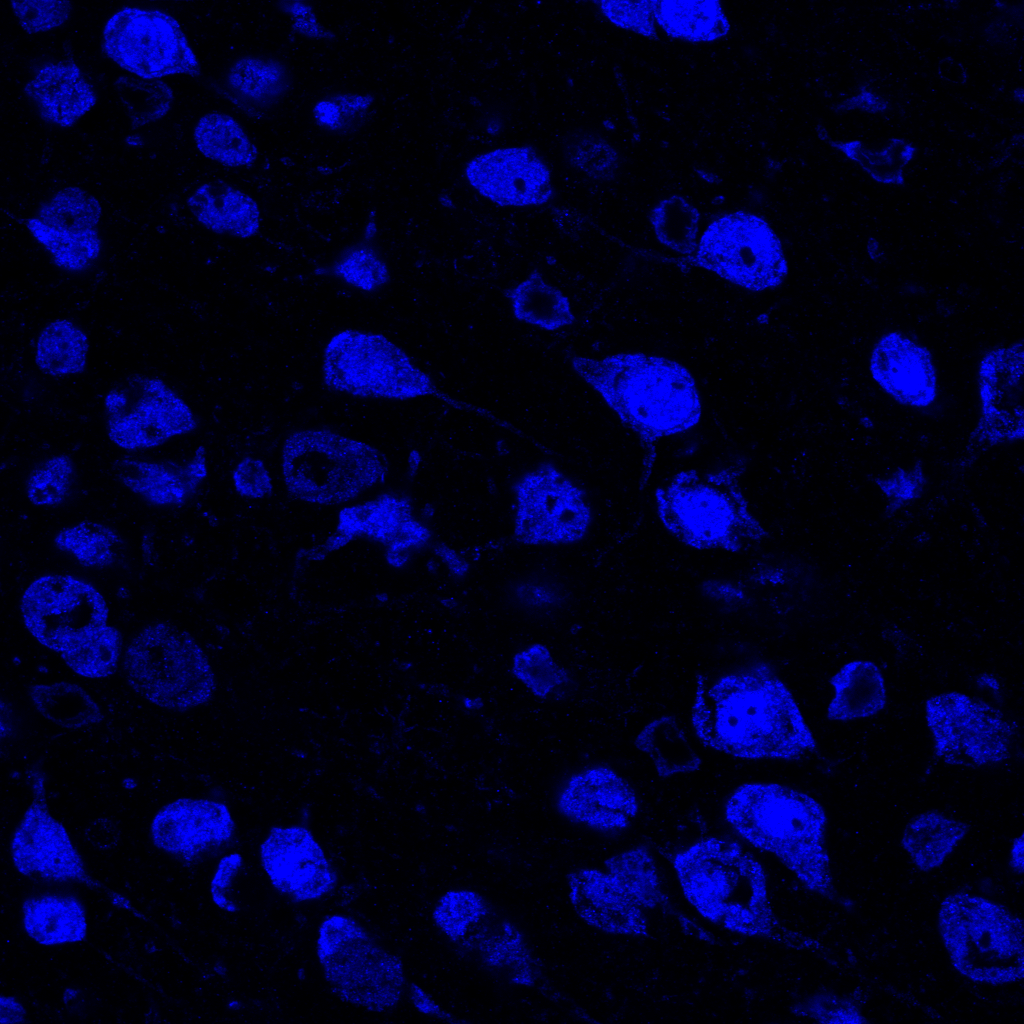

Supplement: Supplementary file 5 — Source data Fig. 4 [file 44321_2024_111_MOESM5_ESM.zip › EMM-2024-19843_SourceData-Figure4/4J/NeuN IHC - KO+COX10.tiff]

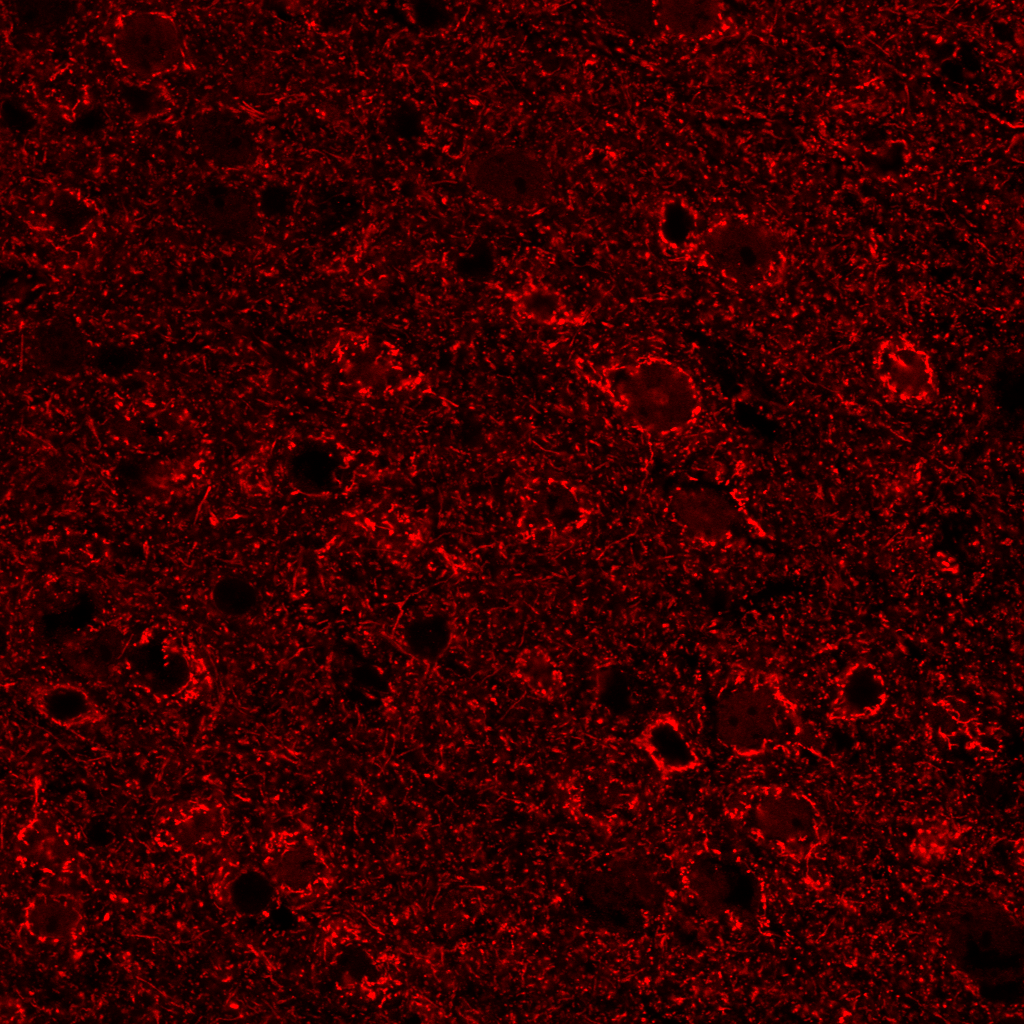

Supplement: Supplementary file 5 — Source data Fig. 4 [file 44321_2024_111_MOESM5_ESM.zip › EMM-2024-19843_SourceData-Figure4/4J/COX1 IHC - KO+COX10.tiff]

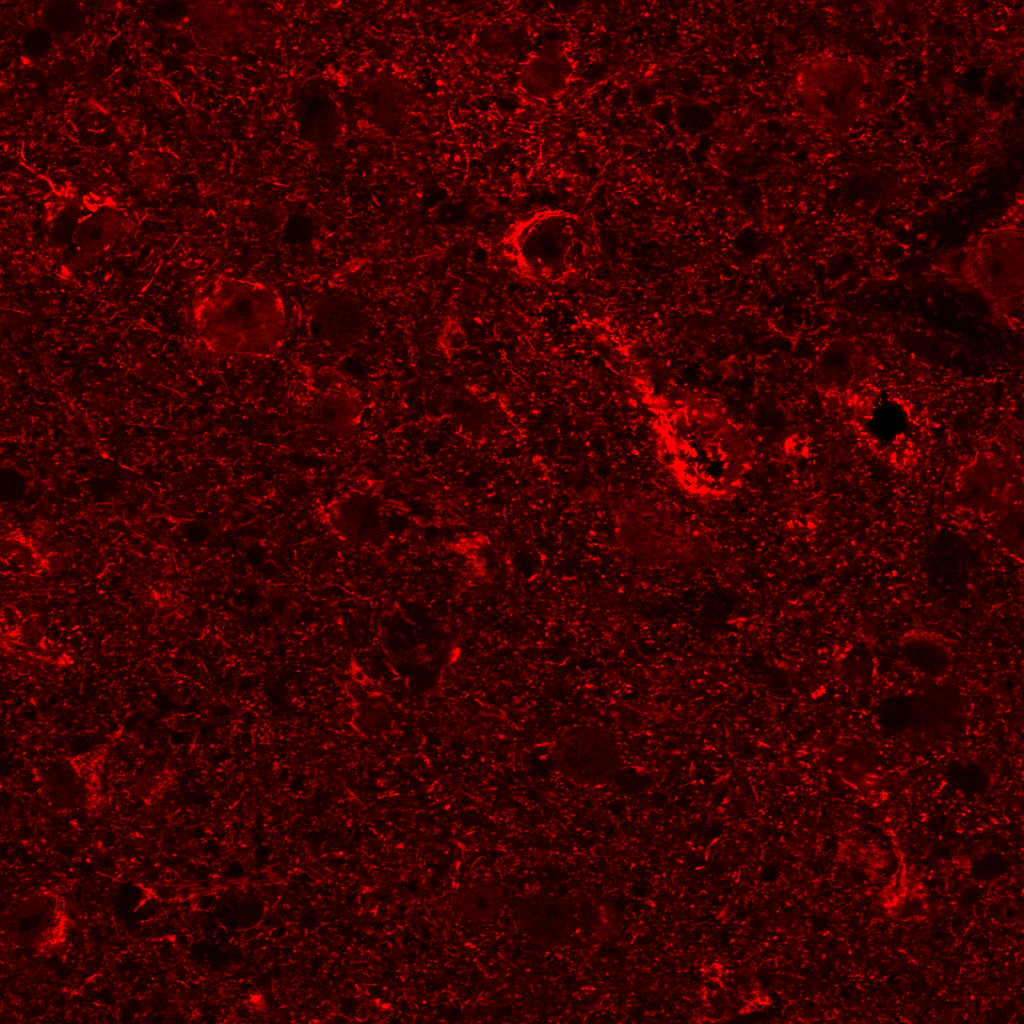

Supplement: Supplementary file 5 — Source data Fig. 4 [file 44321_2024_111_MOESM5_ESM.zip › EMM-2024-19843_SourceData-Figure4/4J/COX1 IHC - KO+GFP.tiff]

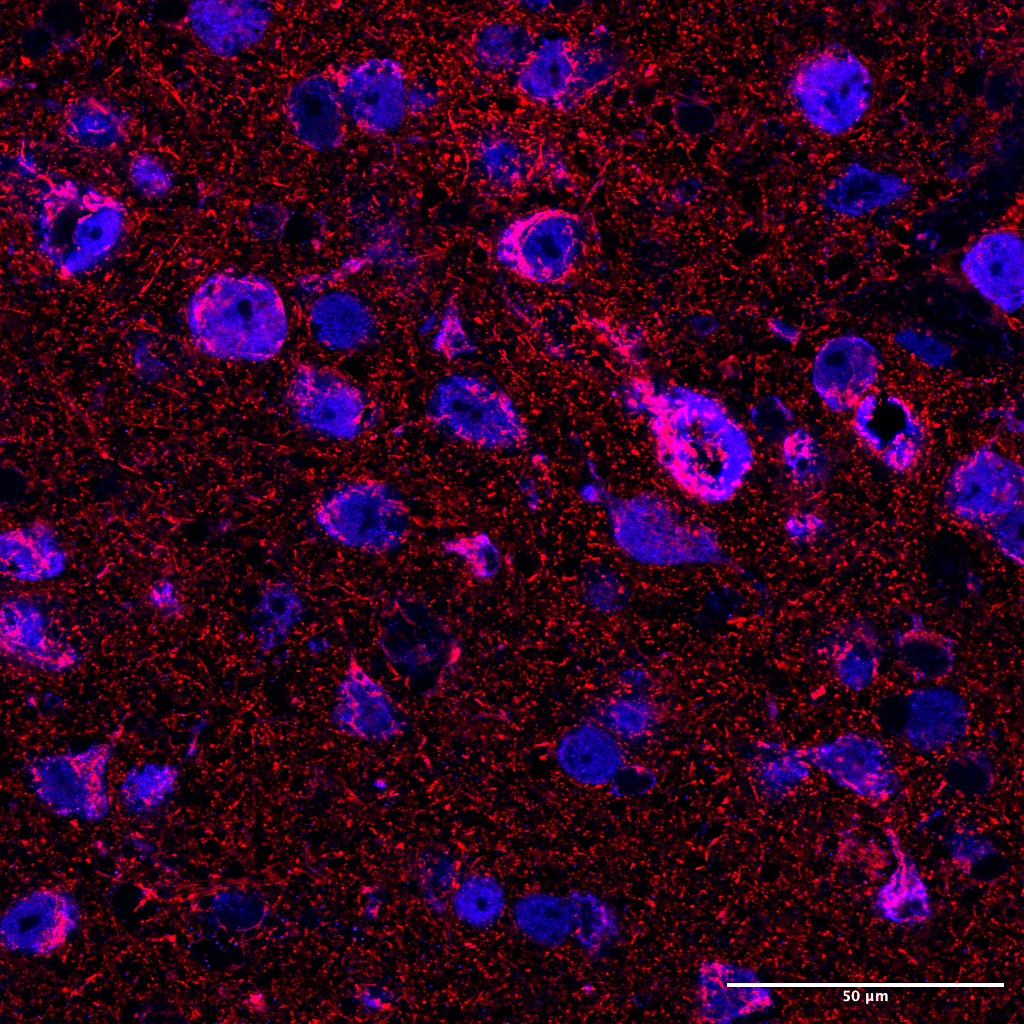

Supplement: Supplementary file 5 — Source data Fig. 4 [file 44321_2024_111_MOESM5_ESM.zip › EMM-2024-19843_SourceData-Figure4/4J/COX1 NeuN IHC - KO+GFP.tiff]

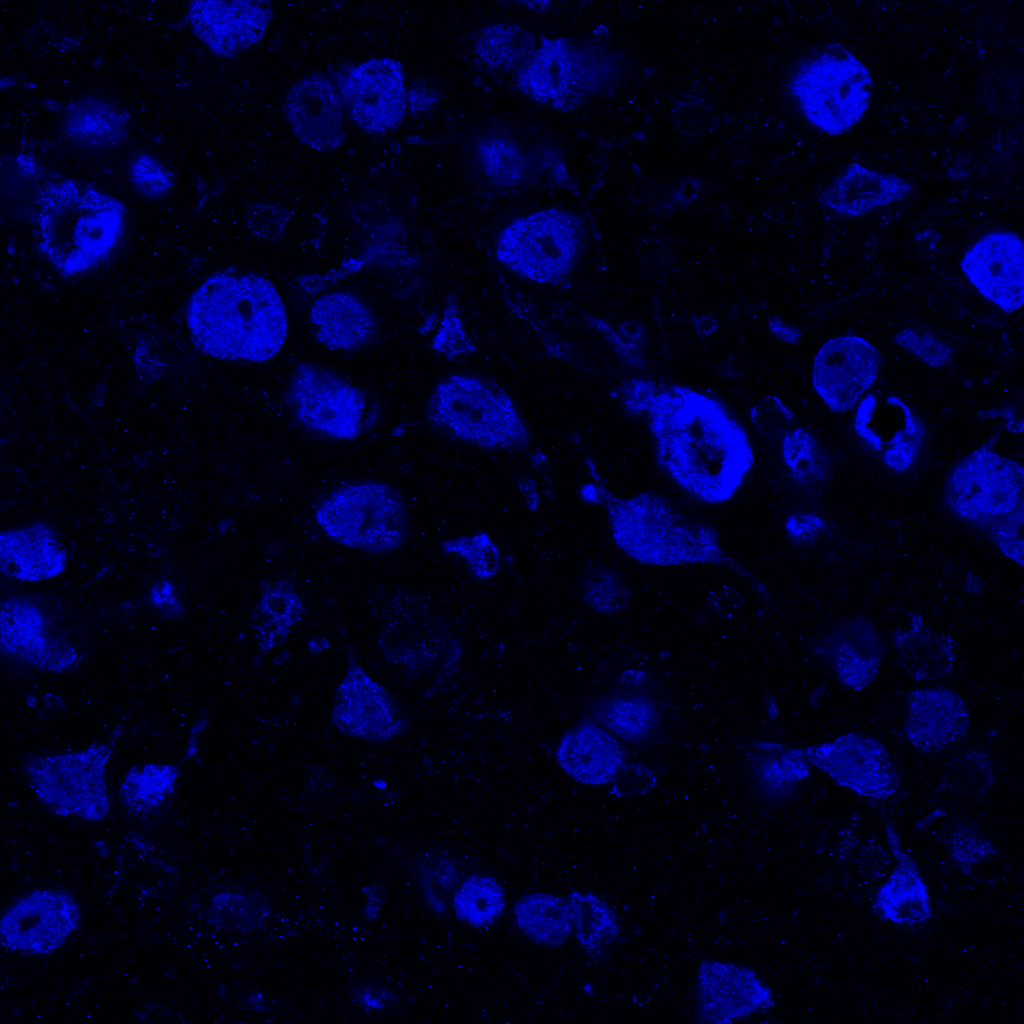

Supplement: Supplementary file 5 — Source data Fig. 4 [file 44321_2024_111_MOESM5_ESM.zip › EMM-2024-19843_SourceData-Figure4/4J/NeuN IHC - KO+GFP.tiff]

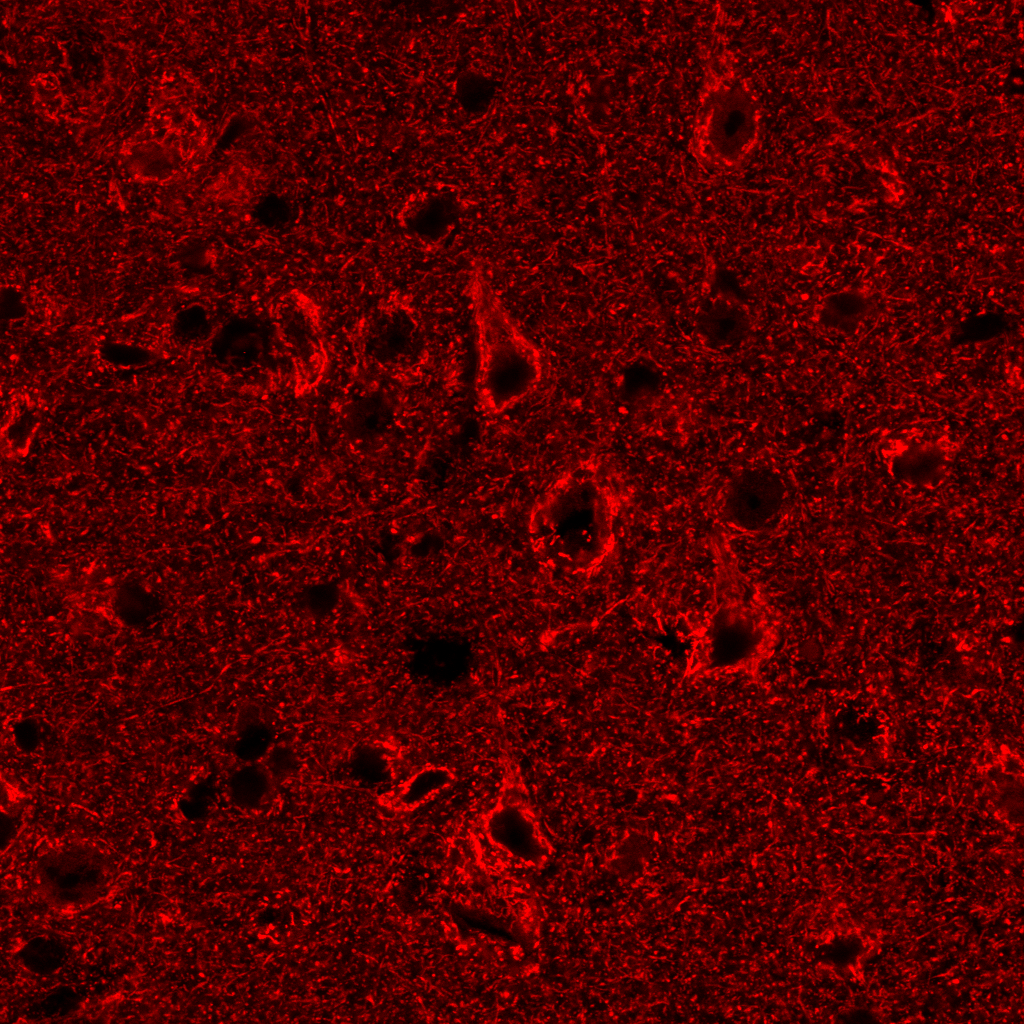

Supplement: Supplementary file 5 — Source data Fig. 4 [file 44321_2024_111_MOESM5_ESM.zip › EMM-2024-19843_SourceData-Figure4/4J/COX1 IHC - WT.tiff]

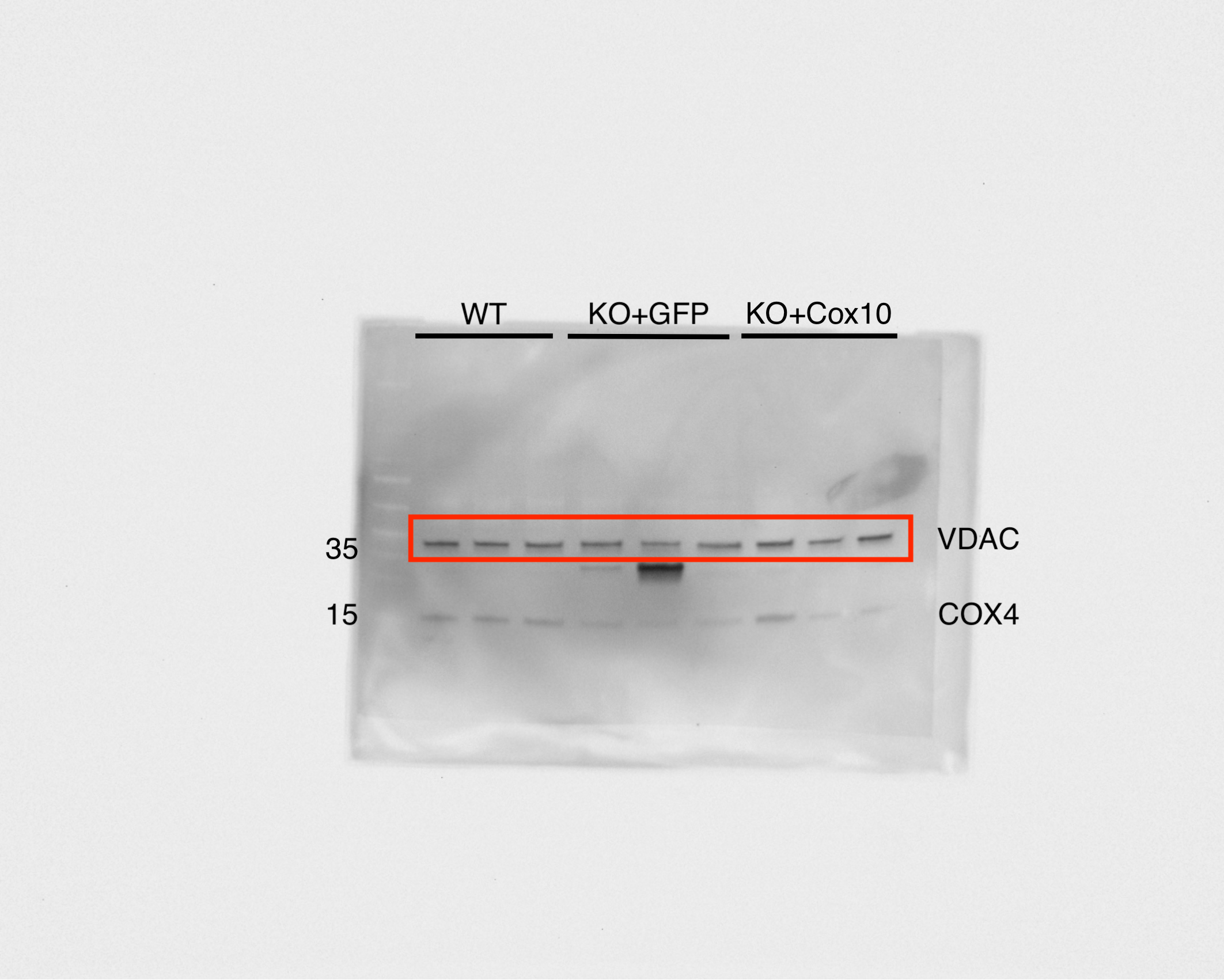

Supplement: Supplementary file 5 — Source data Fig. 4 [file 44321_2024_111_MOESM5_ESM.zip › EMM-2024-19843_SourceData-Figure4/4E/western VDAC.tiff]

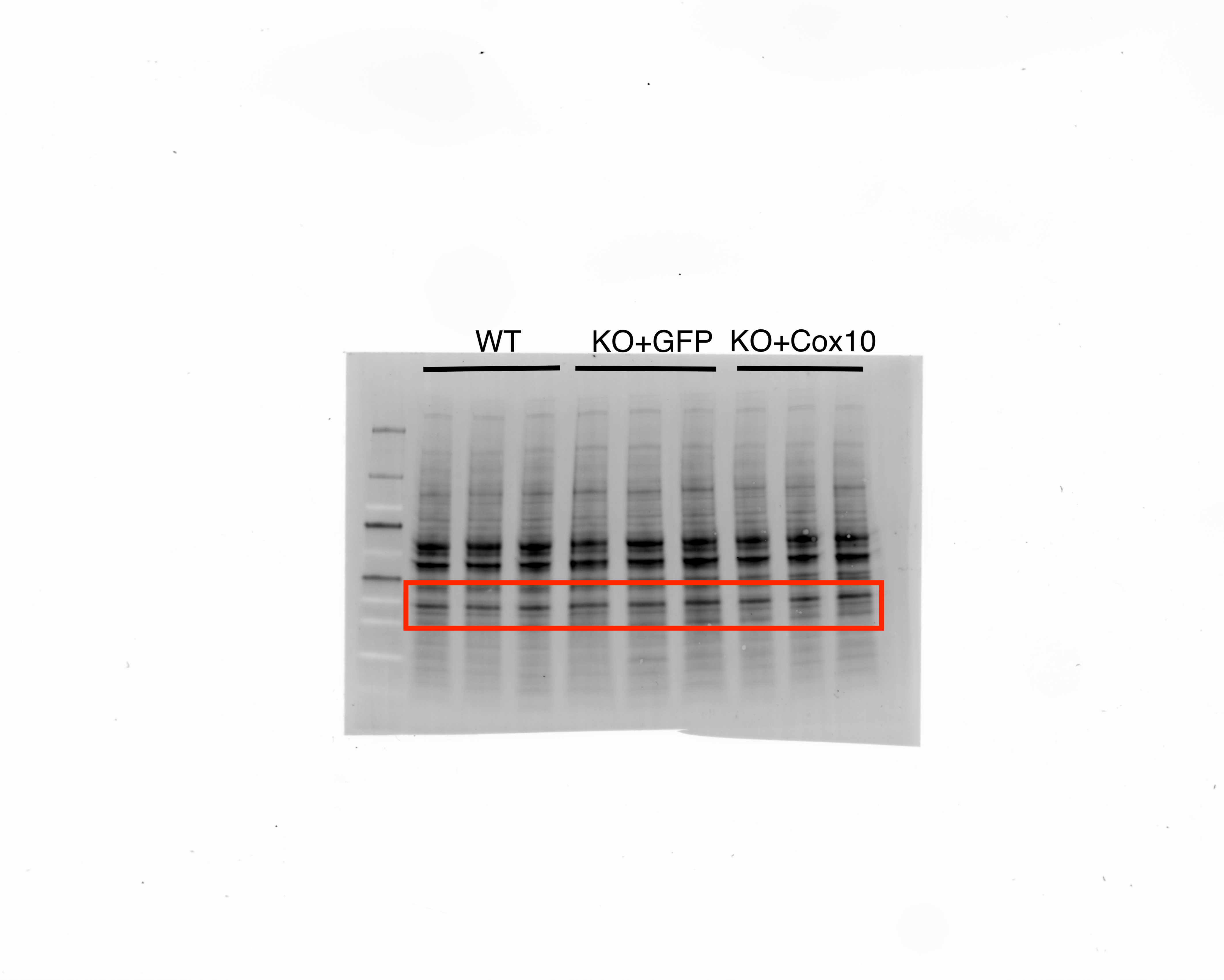

Supplement: Supplementary file 5 — Source data Fig. 4 [file 44321_2024_111_MOESM5_ESM.zip › EMM-2024-19843_SourceData-Figure4/4E/western Total Protein.tif]

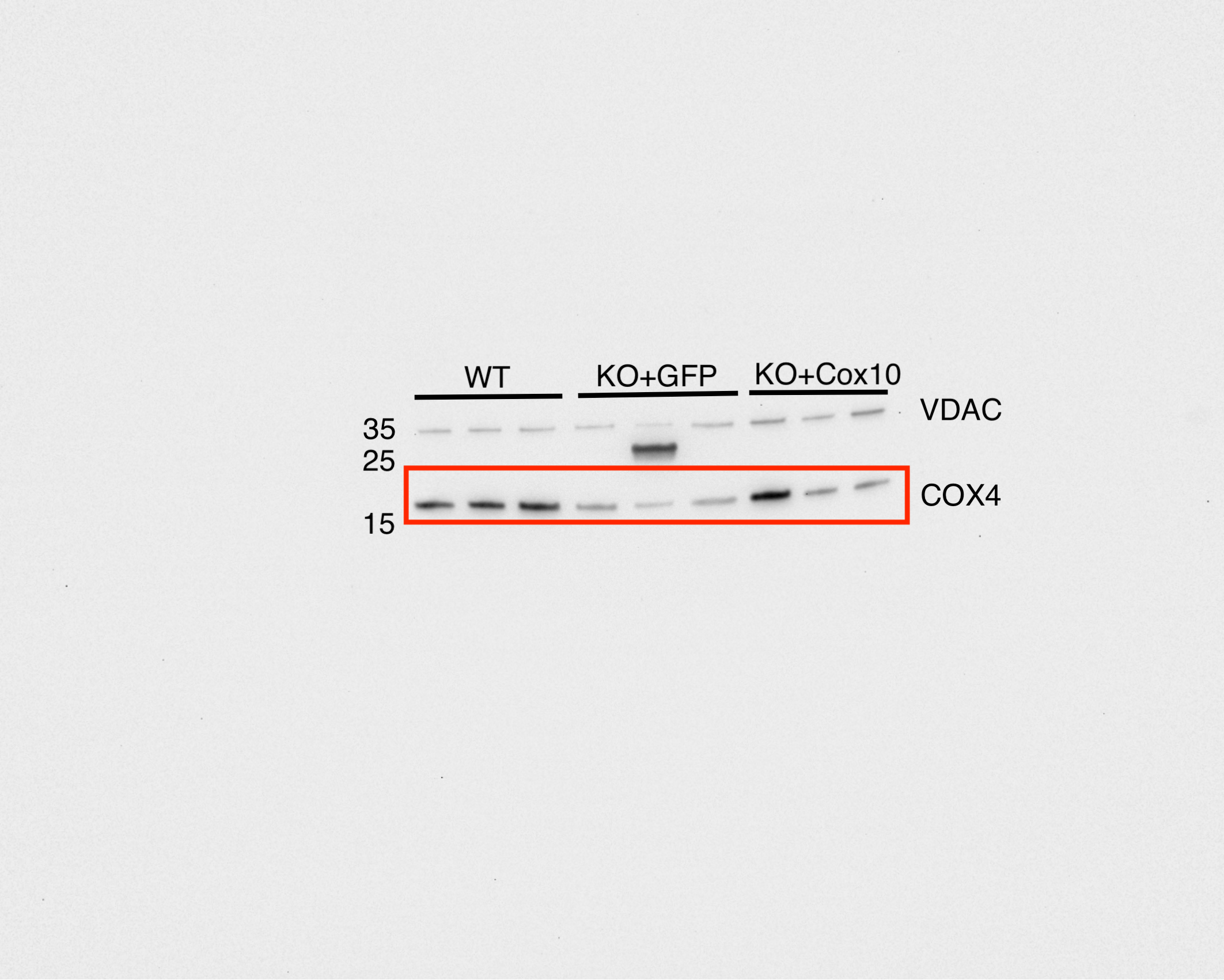

Supplement: Supplementary file 5 — Source data Fig. 4 [file 44321_2024_111_MOESM5_ESM.zip › EMM-2024-19843_SourceData-Figure4/4E/western COX4.tiff]

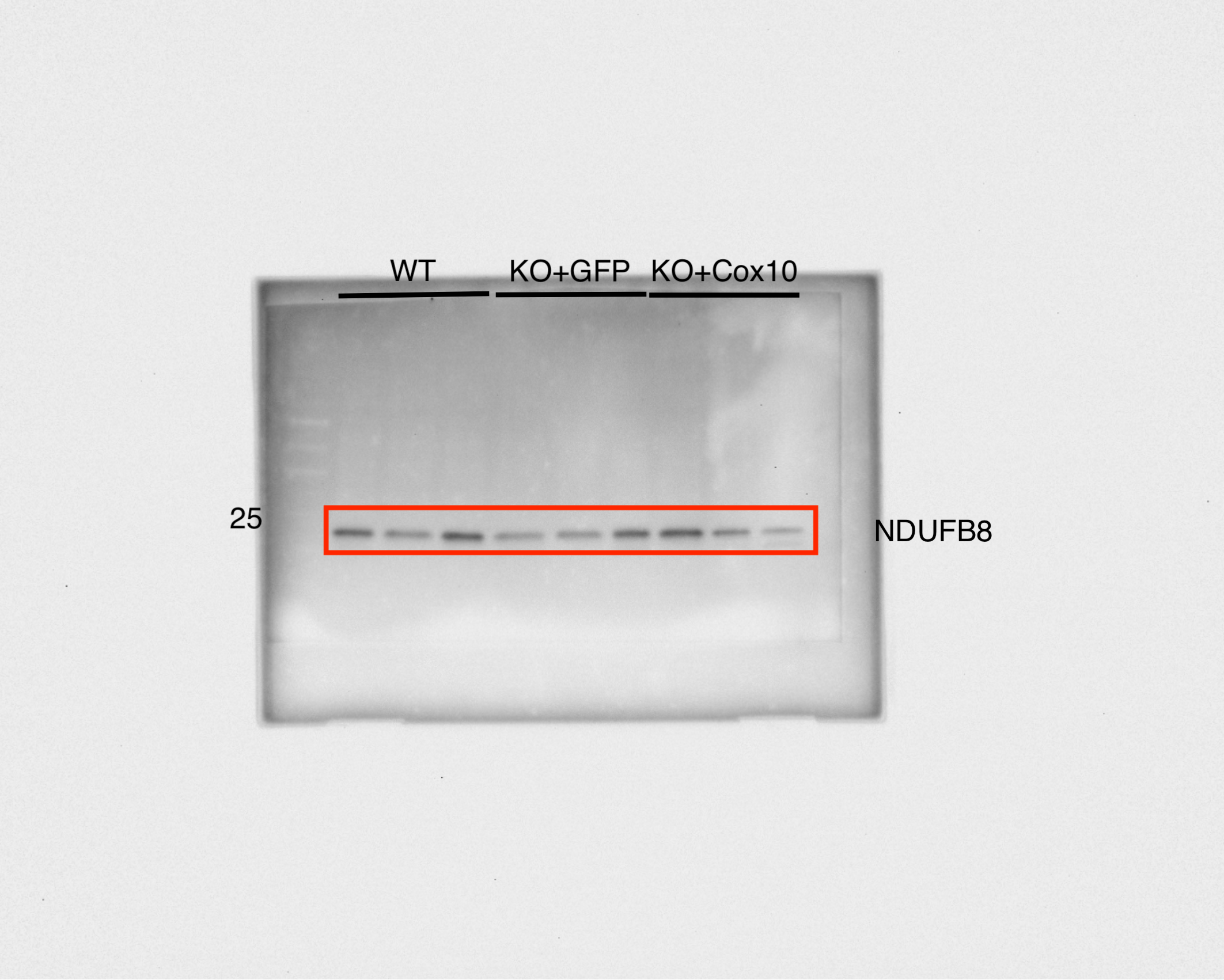

Supplement: Supplementary file 5 — Source data Fig. 4 [file 44321_2024_111_MOESM5_ESM.zip › EMM-2024-19843_SourceData-Figure4/4E/western NDUFB8.tiff]

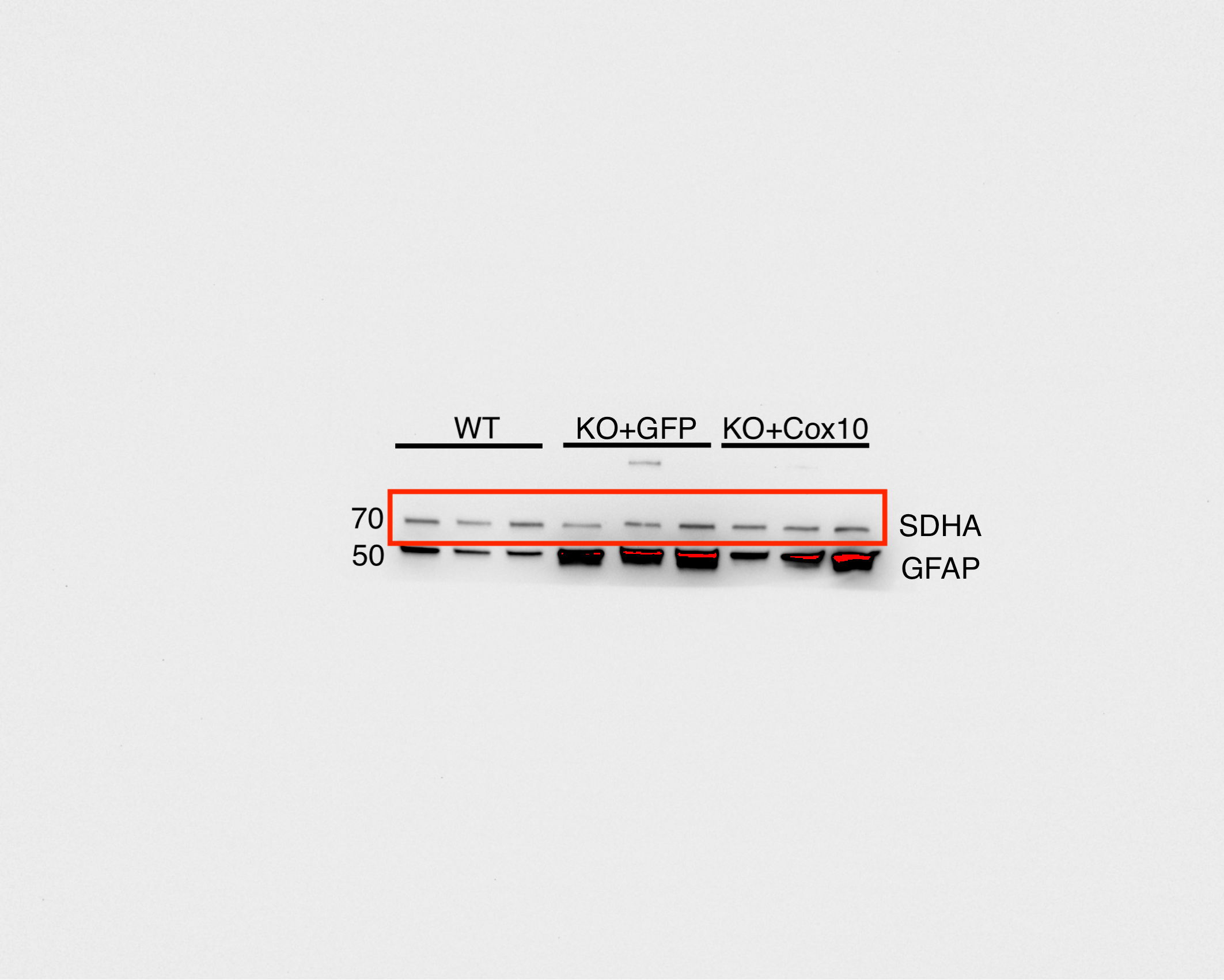

Supplement: Supplementary file 5 — Source data Fig. 4 [file 44321_2024_111_MOESM5_ESM.zip › EMM-2024-19843_SourceData-Figure4/4E/western SDHA.tiff]

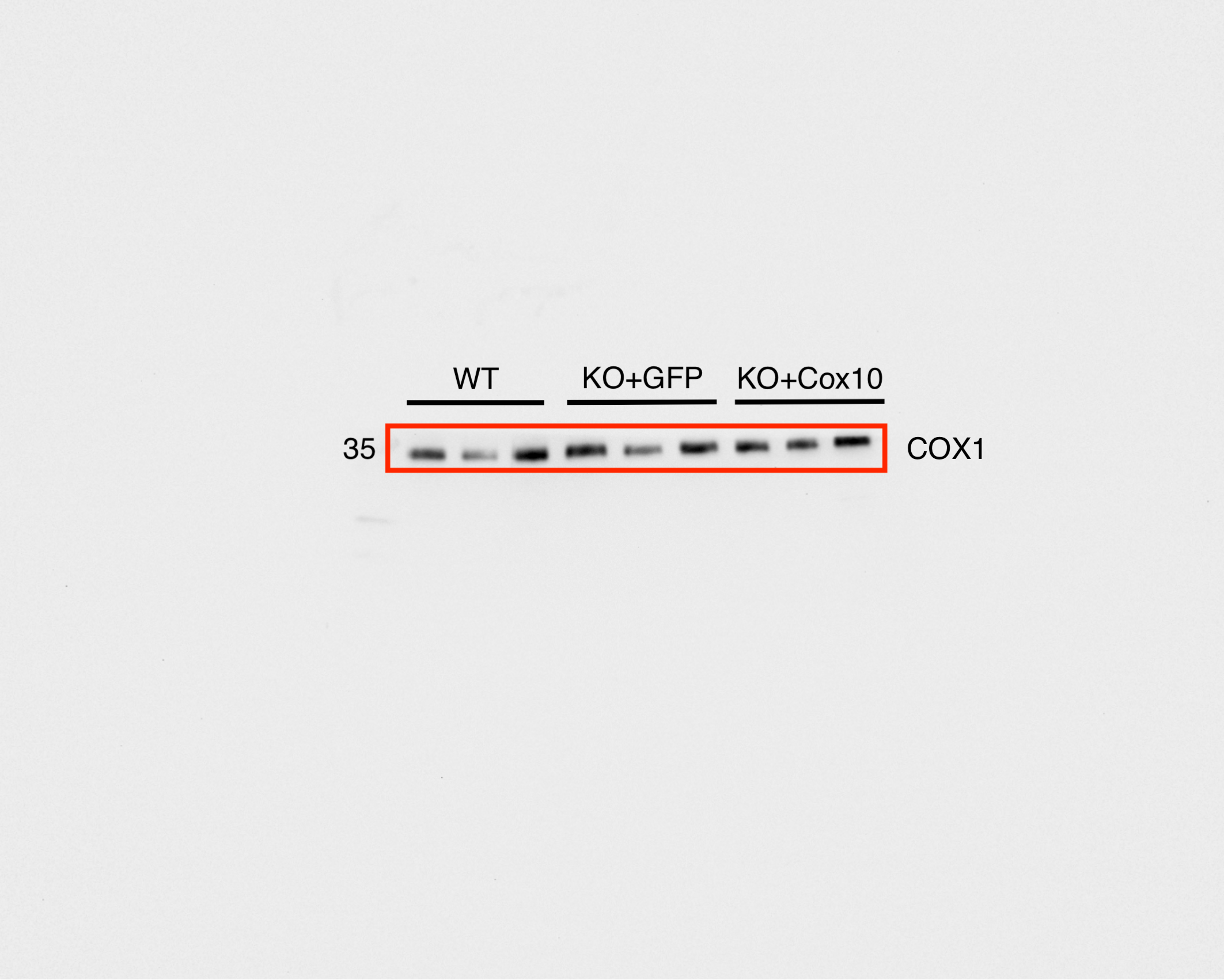

Supplement: Supplementary file 5 — Source data Fig. 4 [file 44321_2024_111_MOESM5_ESM.zip › EMM-2024-19843_SourceData-Figure4/4E/western COX1.tiff]

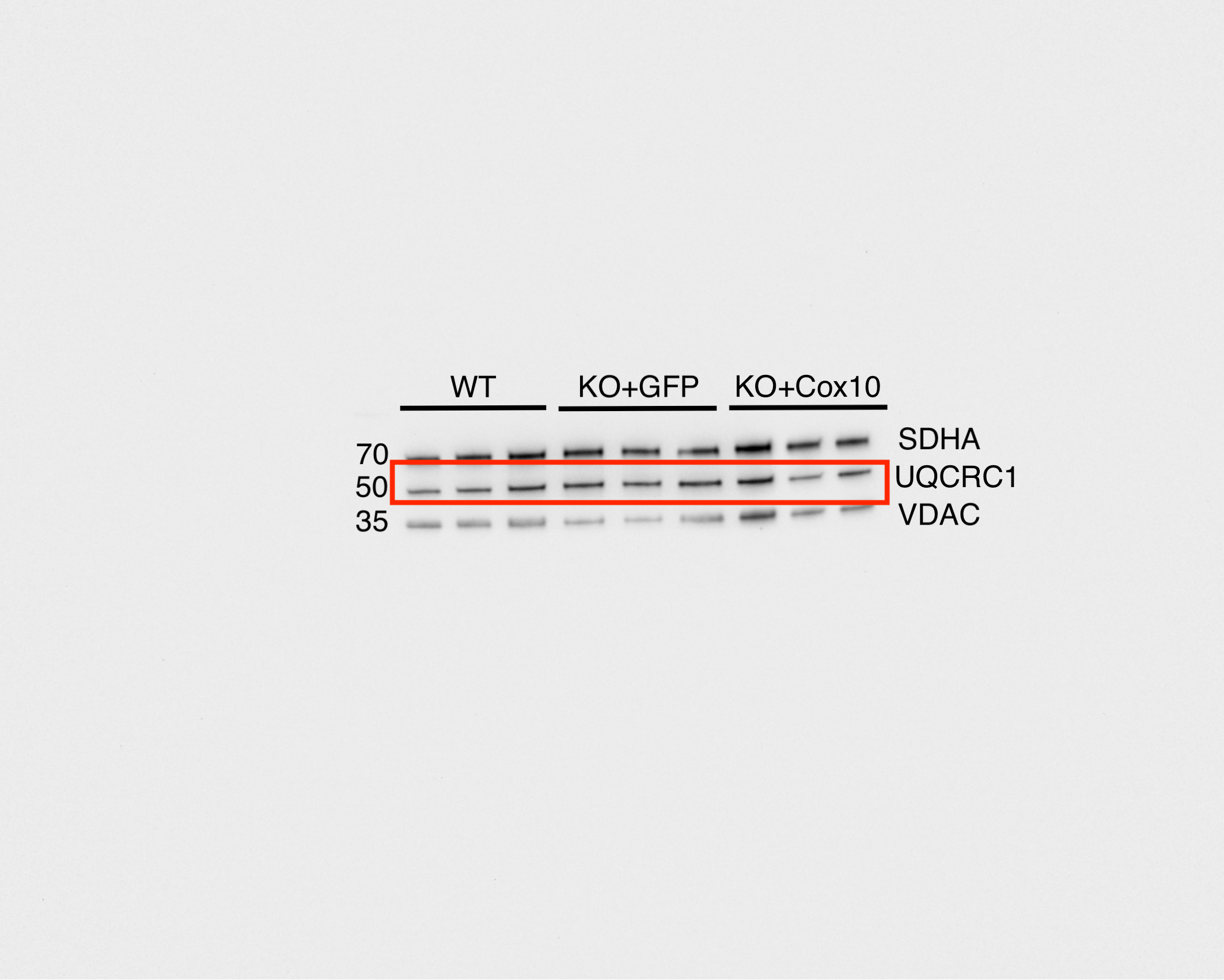

Supplement: Supplementary file 5 — Source data Fig. 4 [file 44321_2024_111_MOESM5_ESM.zip › EMM-2024-19843_SourceData-Figure4/4E/western UQCRC1.tiff]

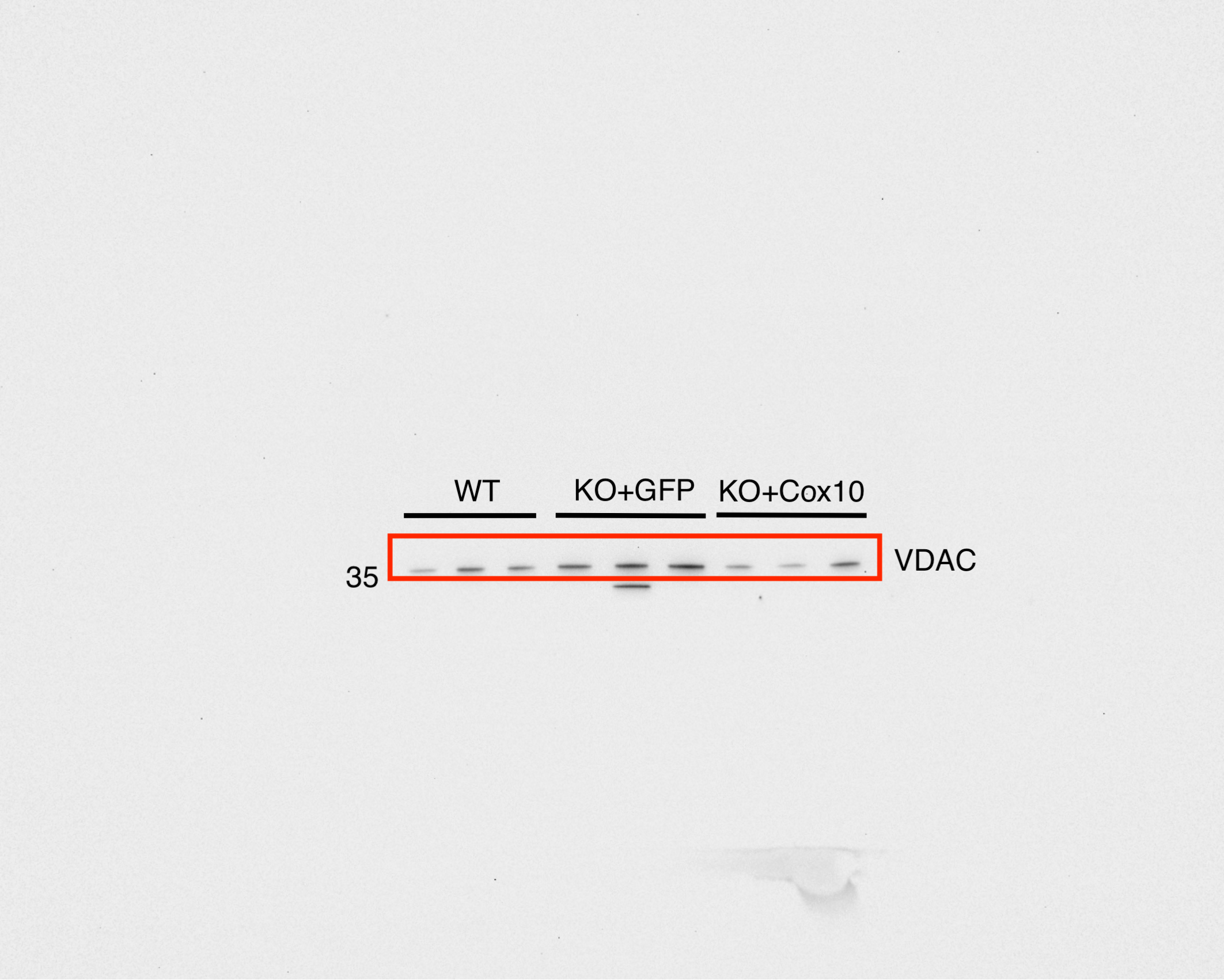

Supplement: Supplementary file 5 — Source data Fig. 4 [file 44321_2024_111_MOESM5_ESM.zip › EMM-2024-19843_SourceData-Figure4/4A/western VDAC.tiff]

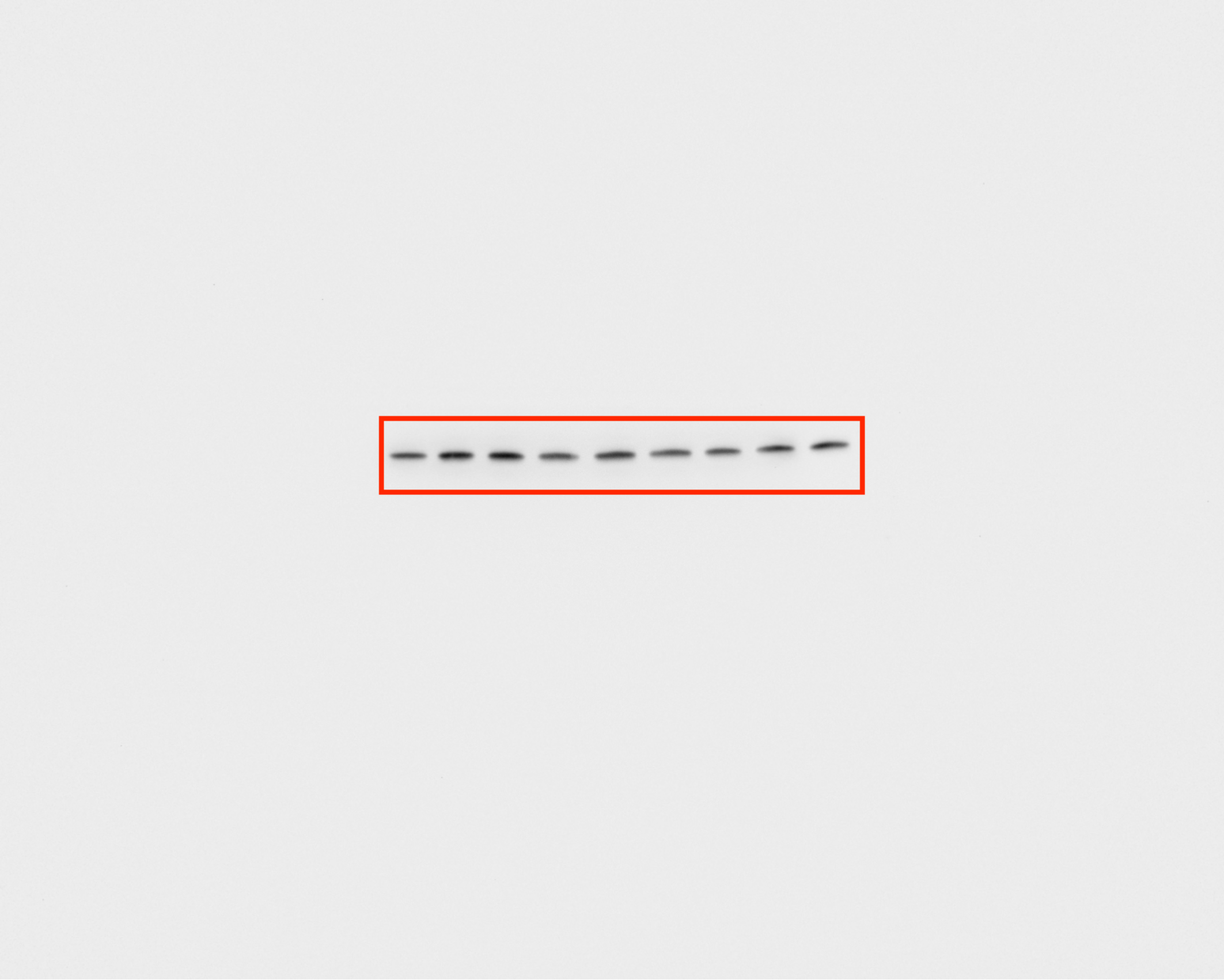

Supplement: Supplementary file 5 — Source data Fig. 4 [file 44321_2024_111_MOESM5_ESM.zip › EMM-2024-19843_SourceData-Figure4/4A/western COX4.tiff]

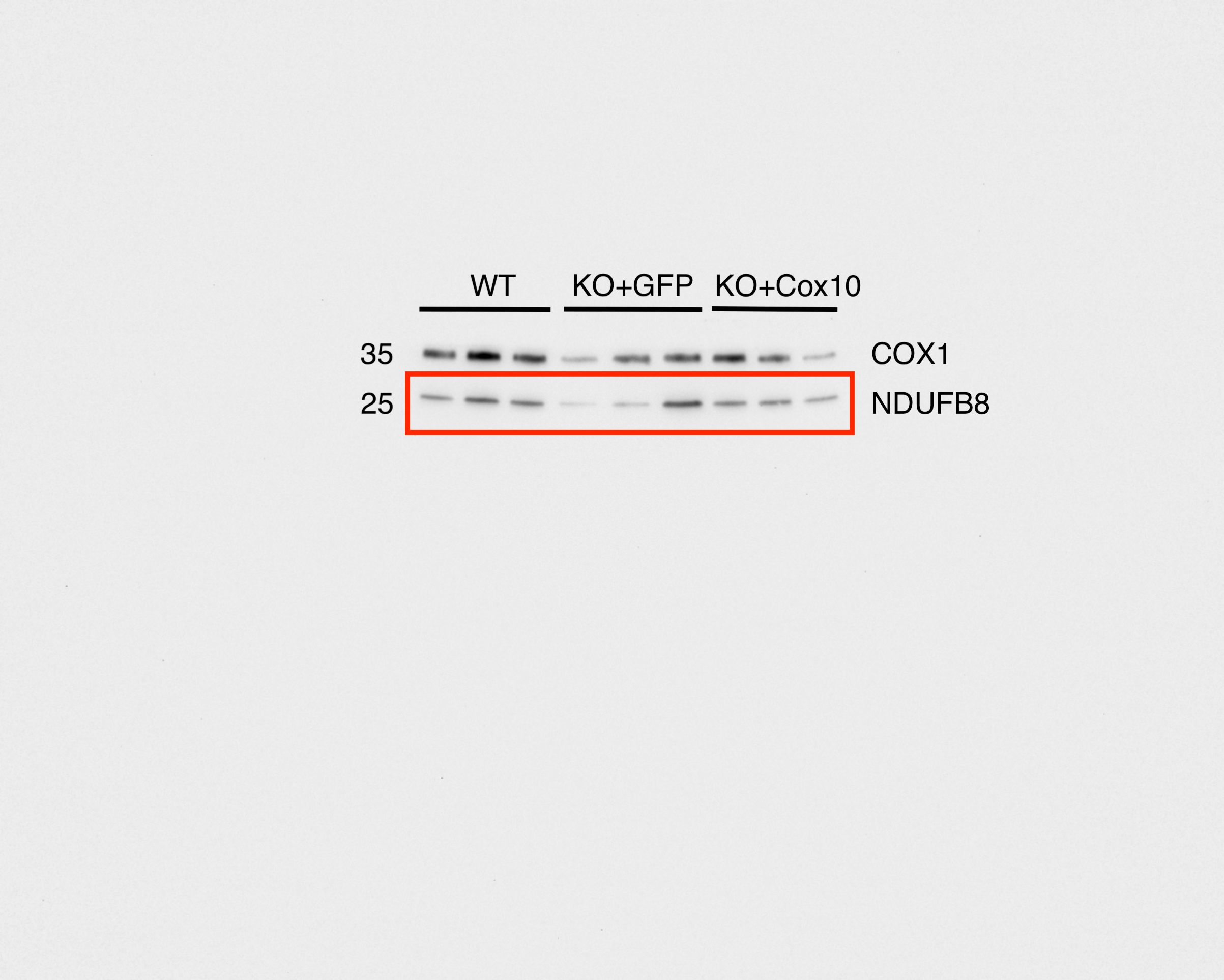

Supplement: Supplementary file 5 — Source data Fig. 4 [file 44321_2024_111_MOESM5_ESM.zip › EMM-2024-19843_SourceData-Figure4/4A/western NDUFB8.tiff]

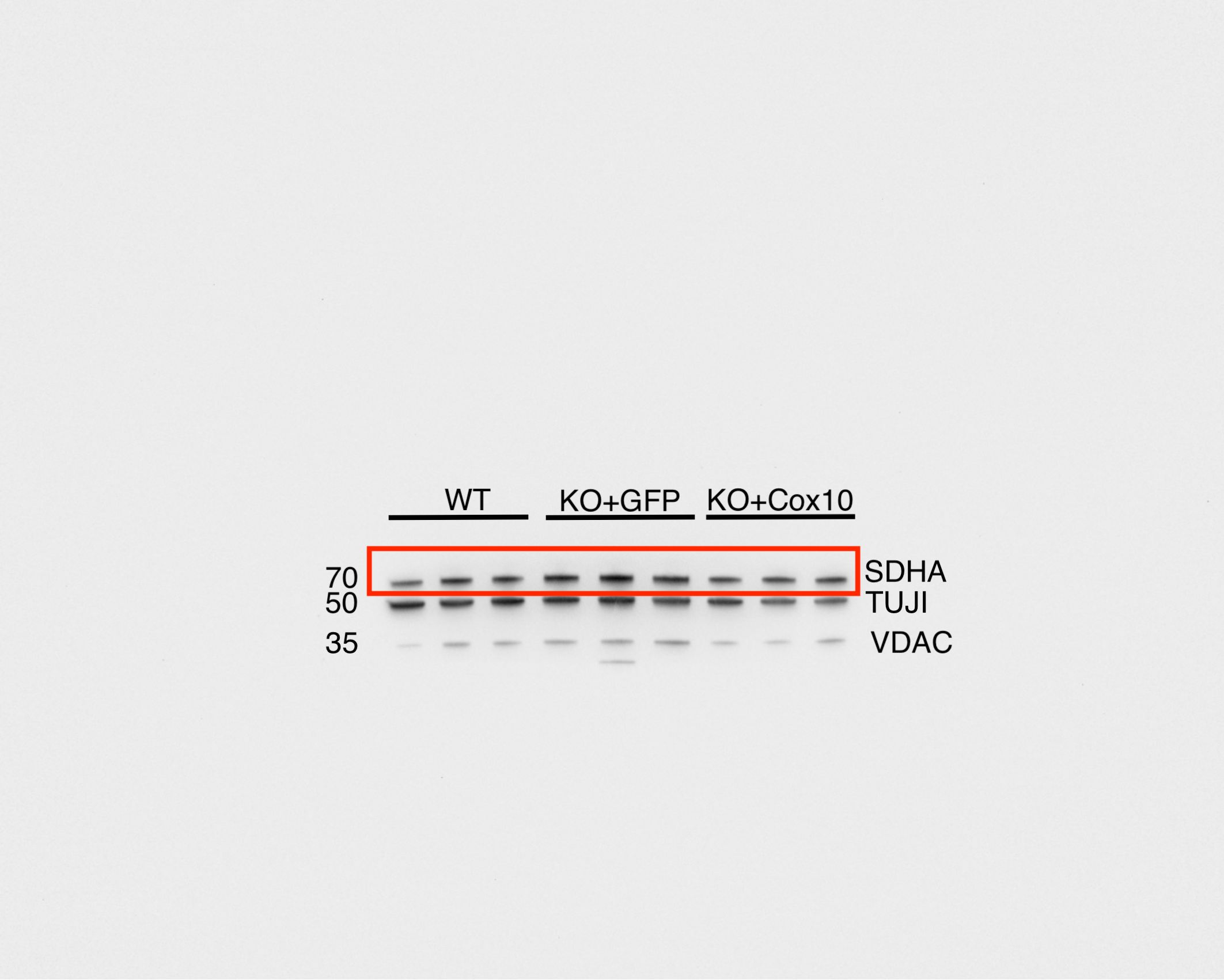

Supplement: Supplementary file 5 — Source data Fig. 4 [file 44321_2024_111_MOESM5_ESM.zip › EMM-2024-19843_SourceData-Figure4/4A/western SDHA.tiff]

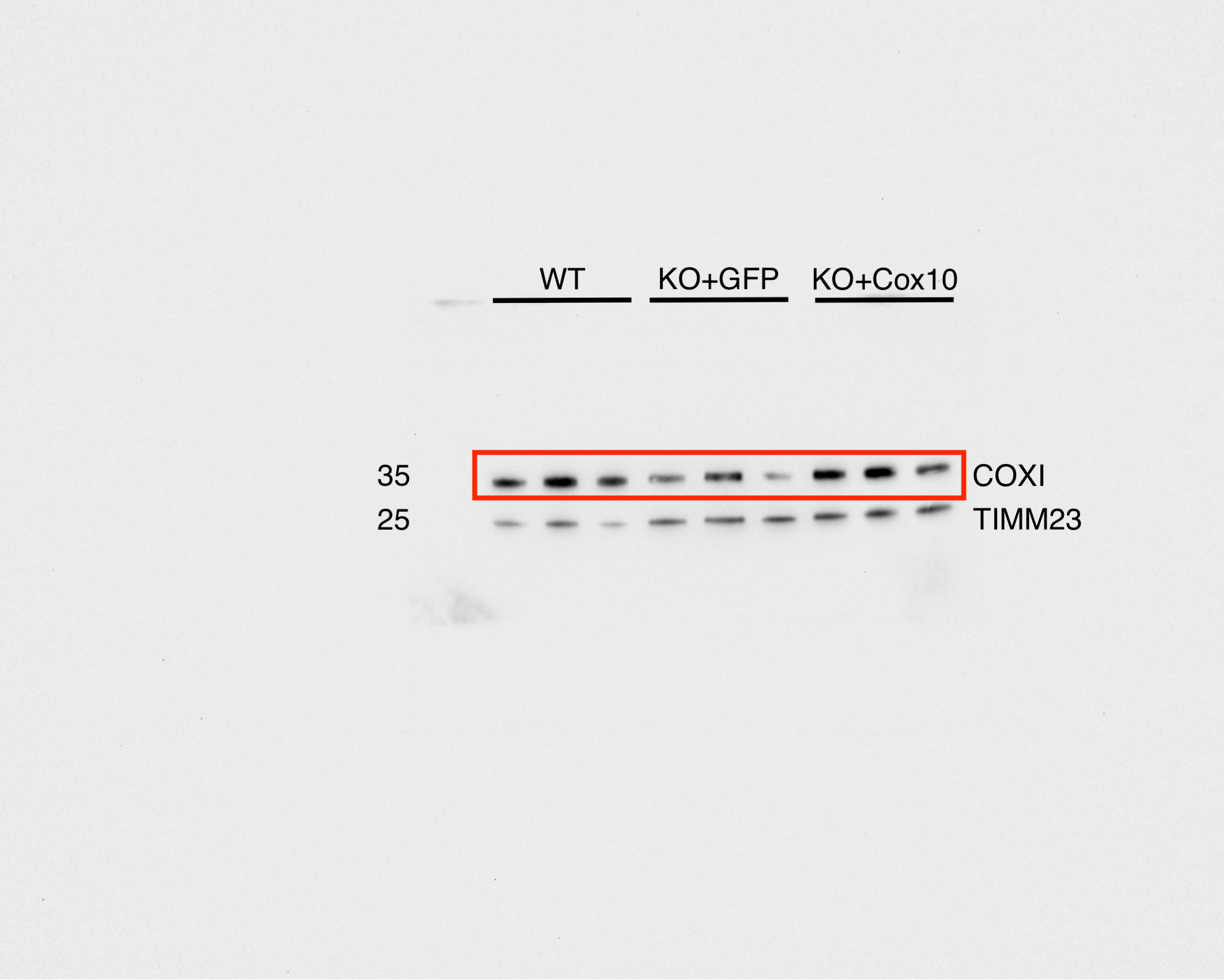

Supplement: Supplementary file 5 — Source data Fig. 4 [file 44321_2024_111_MOESM5_ESM.zip › EMM-2024-19843_SourceData-Figure4/4A/western COX1.tiff]

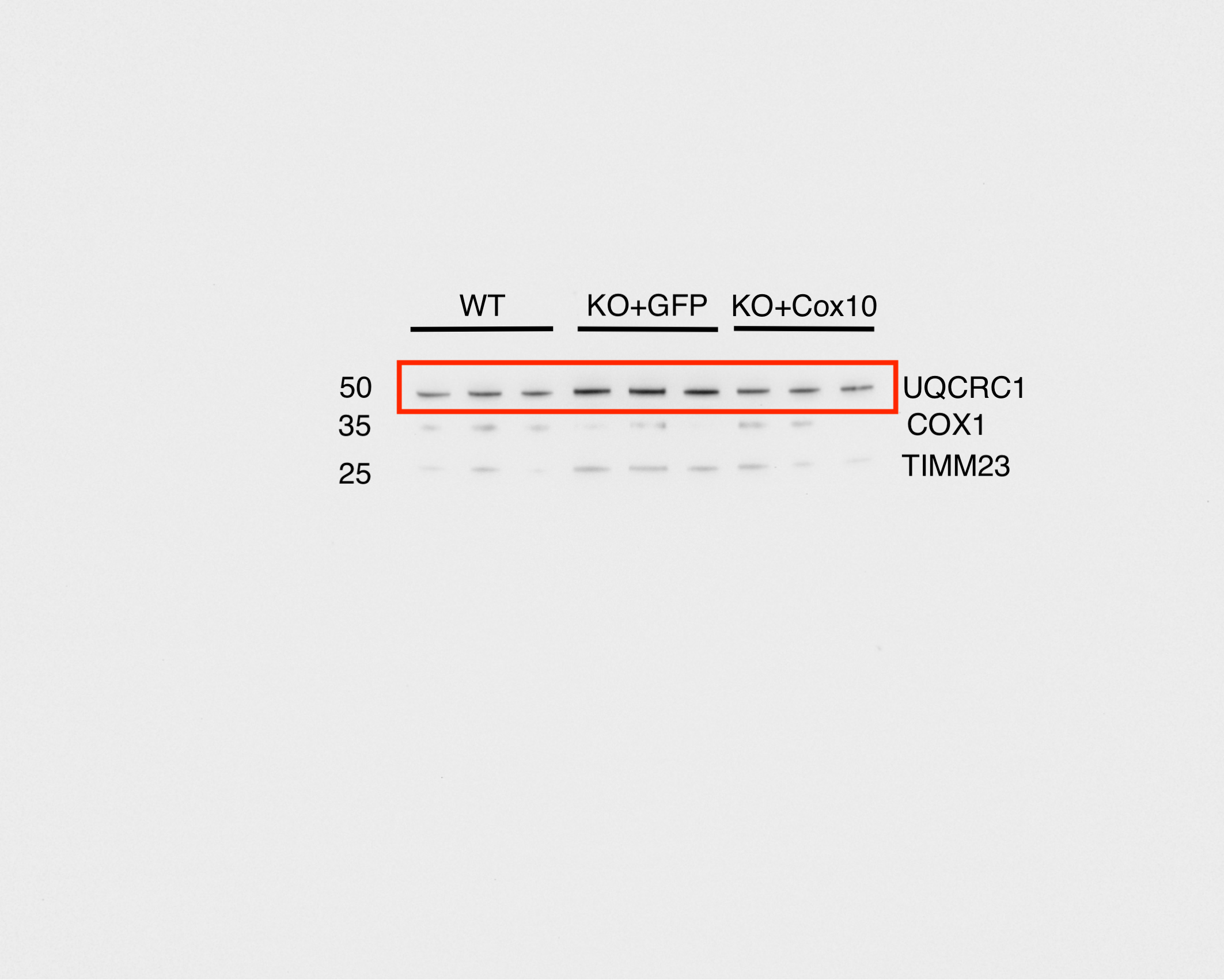

Supplement: Supplementary file 5 — Source data Fig. 4 [file 44321_2024_111_MOESM5_ESM.zip › EMM-2024-19843_SourceData-Figure4/4A/western UQCRC1.tiff]

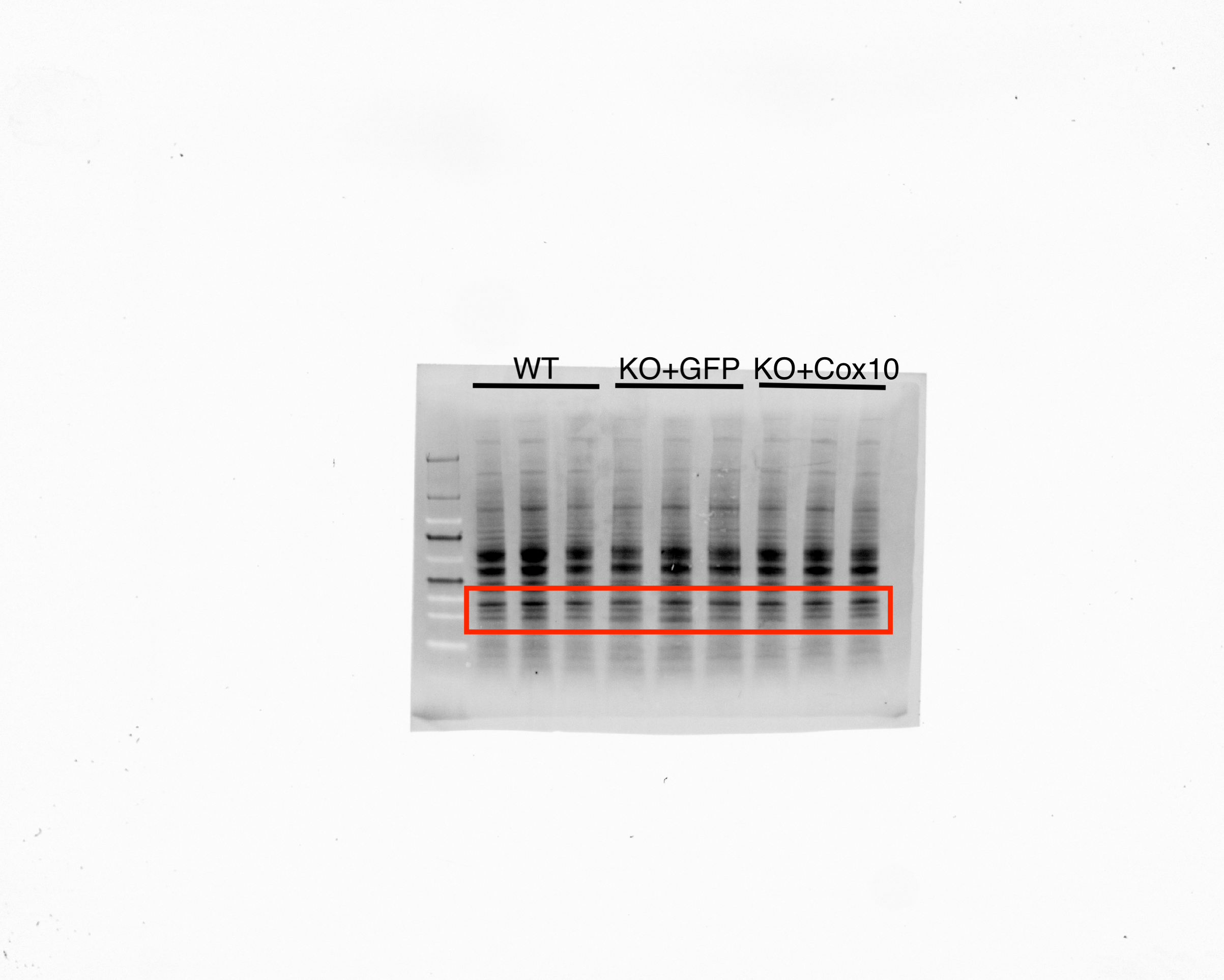

Supplement: Supplementary file 5 — Source data Fig. 4 [file 44321_2024_111_MOESM5_ESM.zip › EMM-2024-19843_SourceData-Figure4/4A/western Total Protein.tiff]

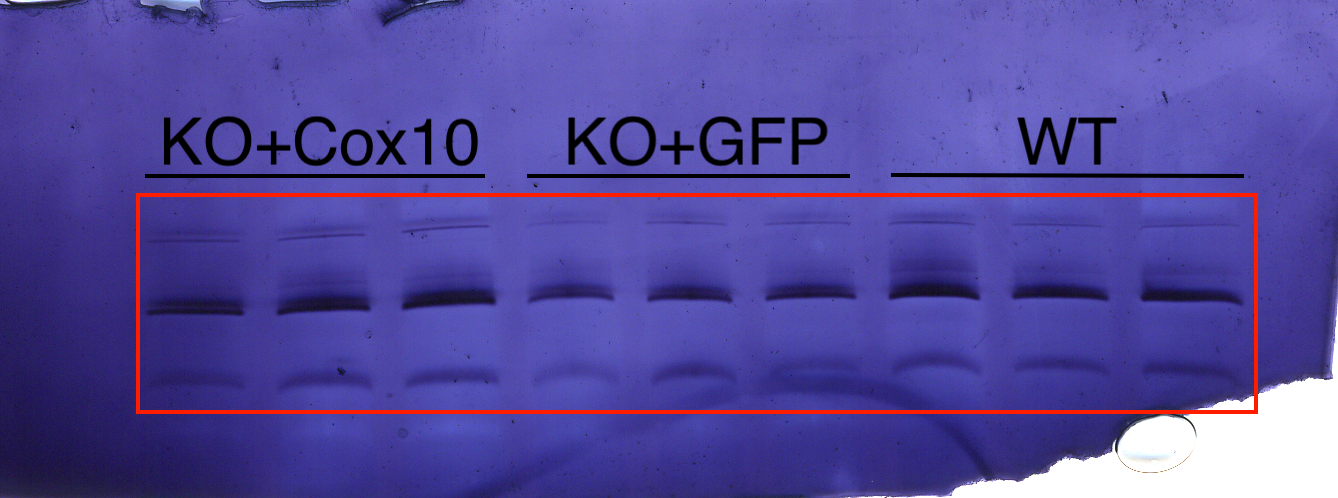

Supplement: Supplementary file 6 — Source data Fig. 5 [file 44321_2024_111_MOESM6_ESM.zip › EMM-2024-19843_SourceData-Figure5/5G/IGA - Complex I.tiff]

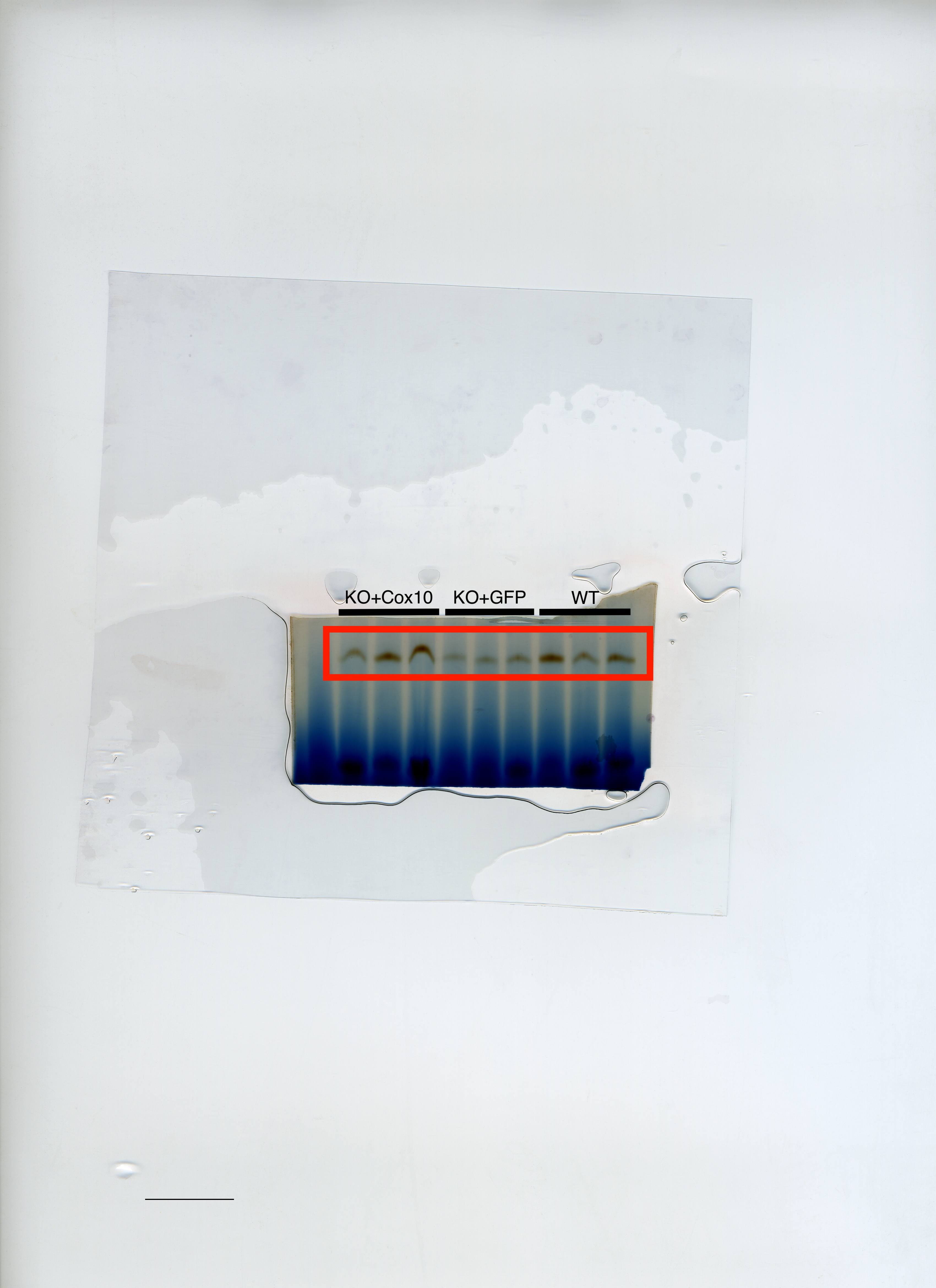

Supplement: Supplementary file 6 — Source data Fig. 5 [file 44321_2024_111_MOESM6_ESM.zip › EMM-2024-19843_SourceData-Figure5/5G/IGA - Complex IV.tiff]

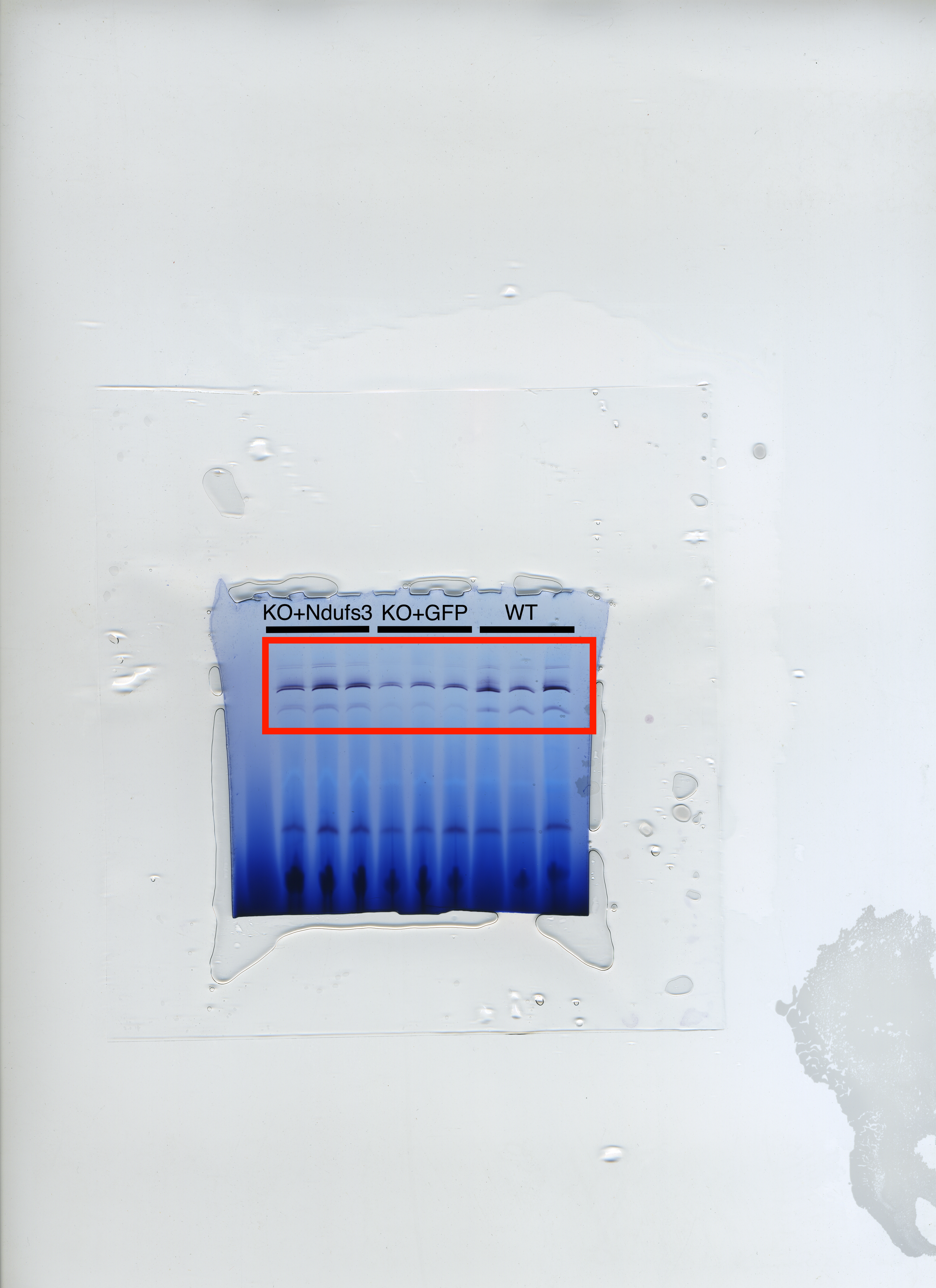

Supplement: Supplementary file 6 — Source data Fig. 5 [file 44321_2024_111_MOESM6_ESM.zip › EMM-2024-19843_SourceData-Figure5/5C/IGA - Complex I.tiff]

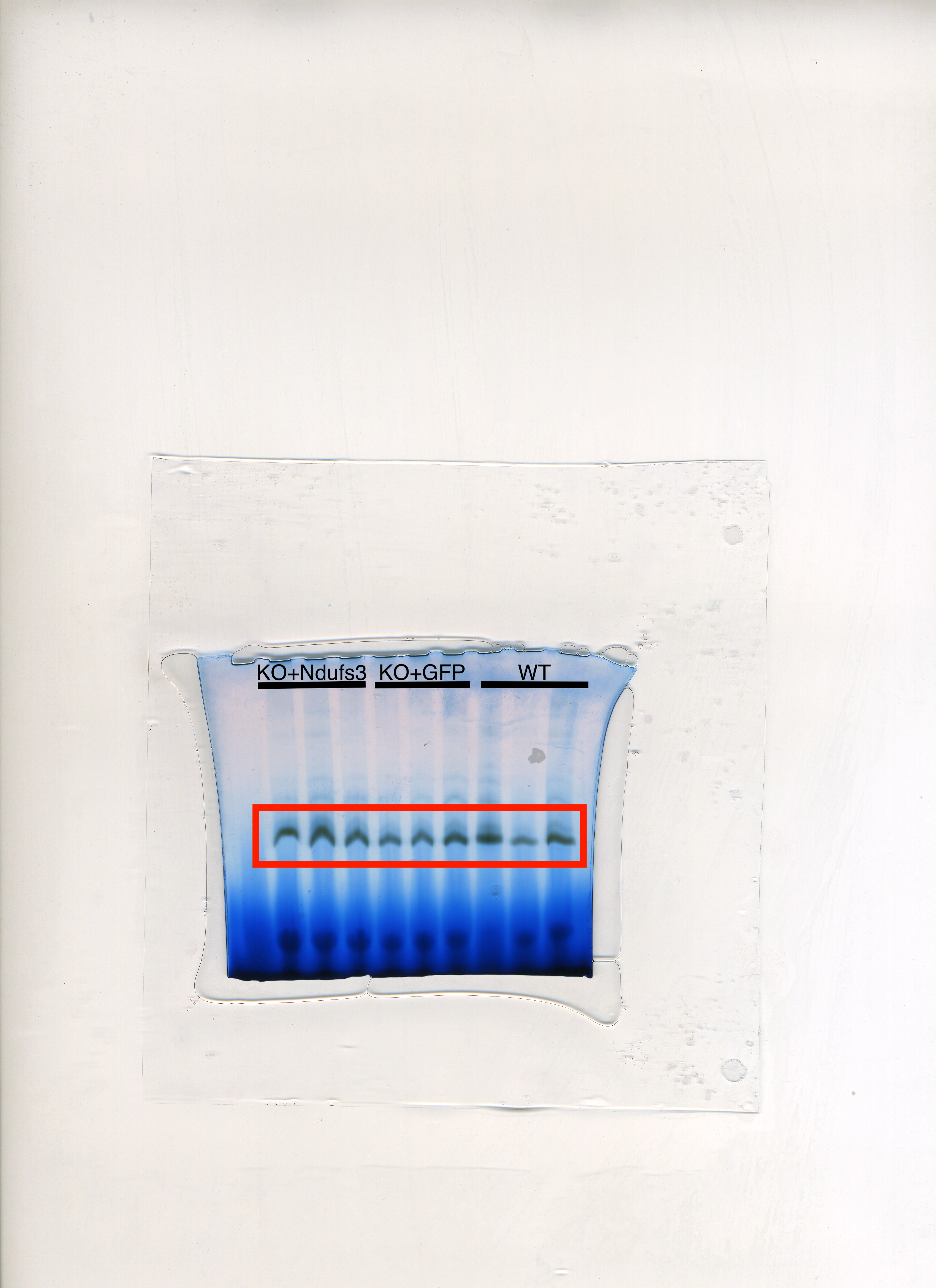

Supplement: Supplementary file 6 — Source data Fig. 5 [file 44321_2024_111_MOESM6_ESM.zip › EMM-2024-19843_SourceData-Figure5/5C/IGA - Complex IV.tiff]

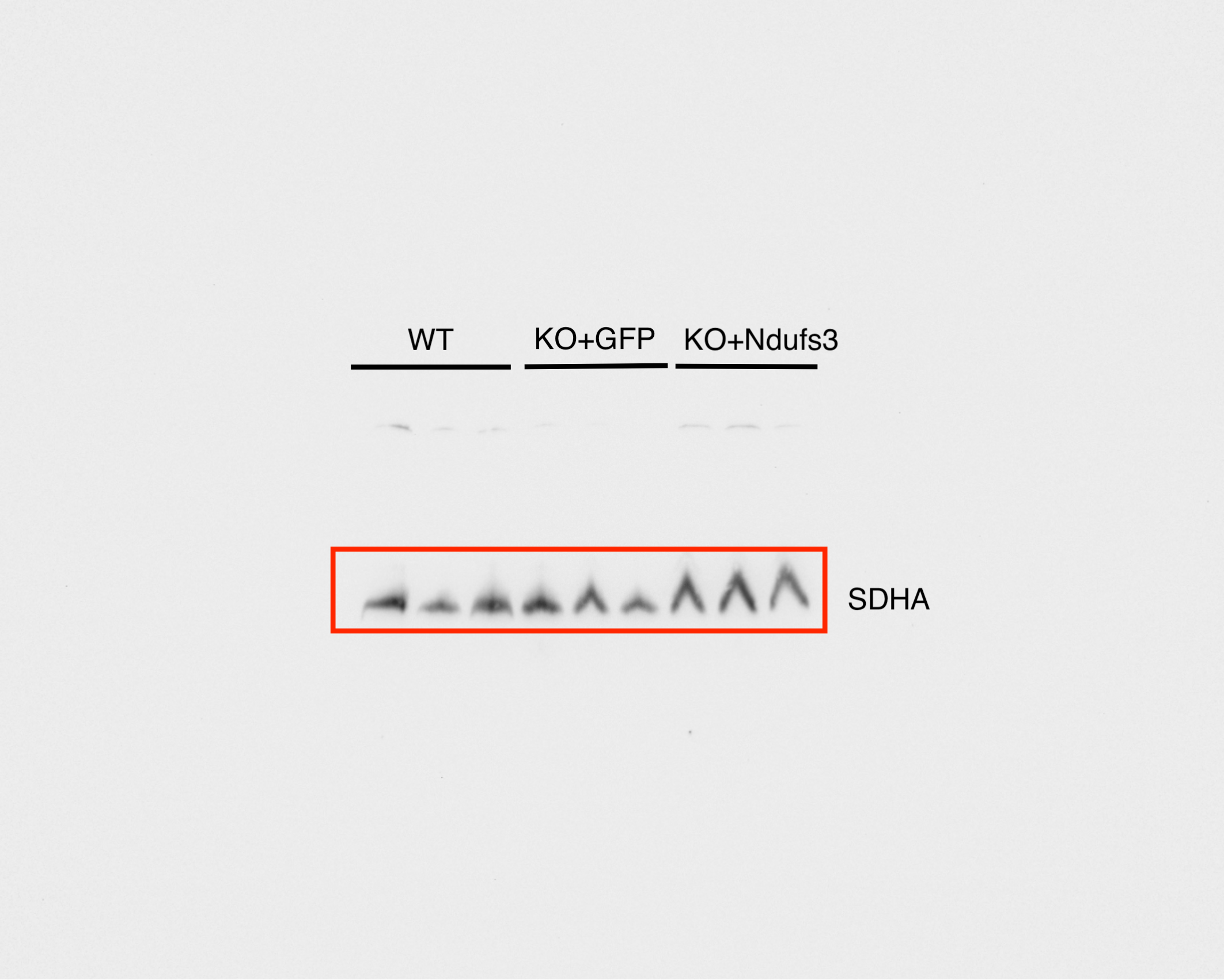

Supplement: Supplementary file 6 — Source data Fig. 5 [file 44321_2024_111_MOESM6_ESM.zip › EMM-2024-19843_SourceData-Figure5/5A/western SDHA.tiff]

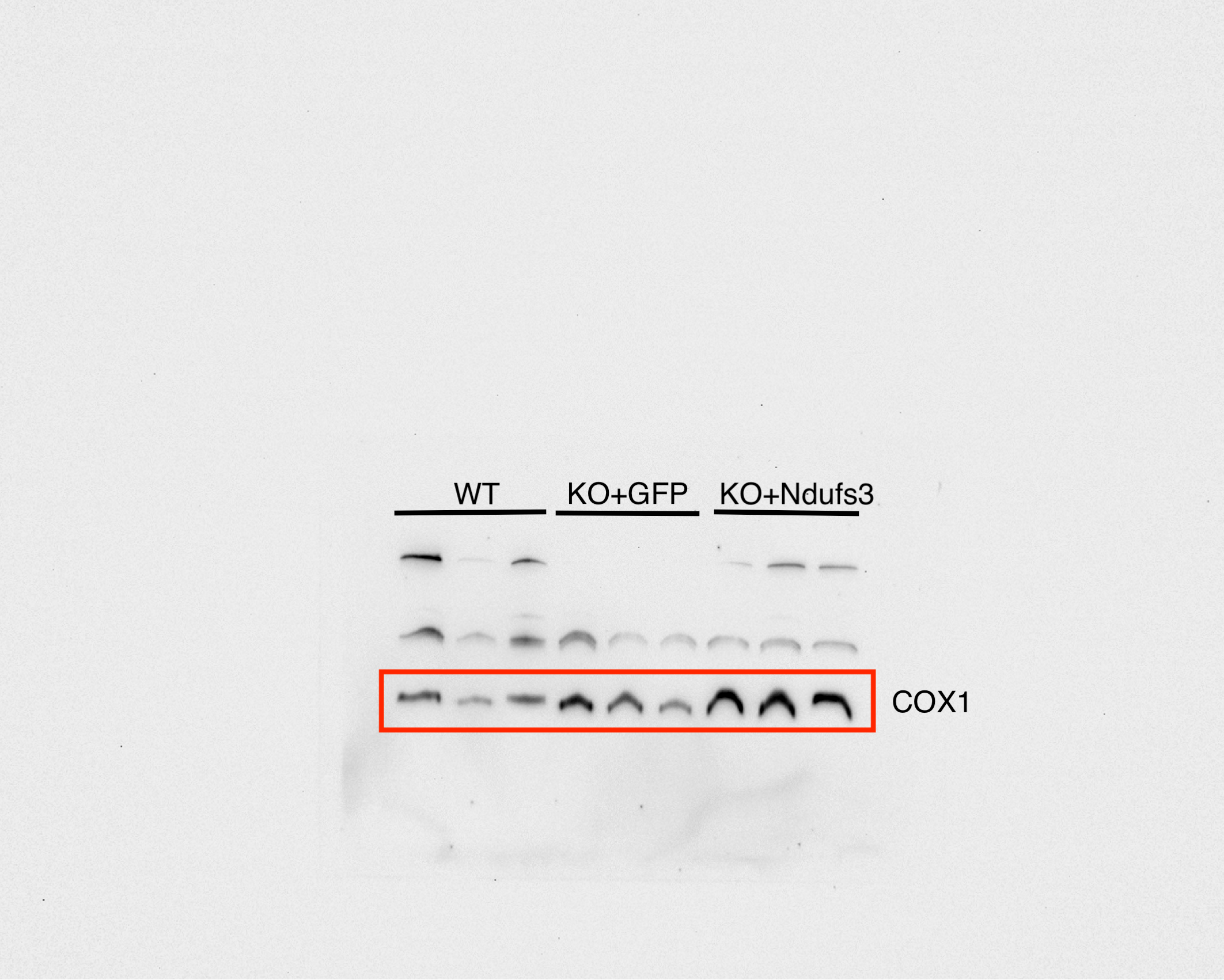

Supplement: Supplementary file 6 — Source data Fig. 5 [file 44321_2024_111_MOESM6_ESM.zip › EMM-2024-19843_SourceData-Figure5/5A/western COX1.tiff]

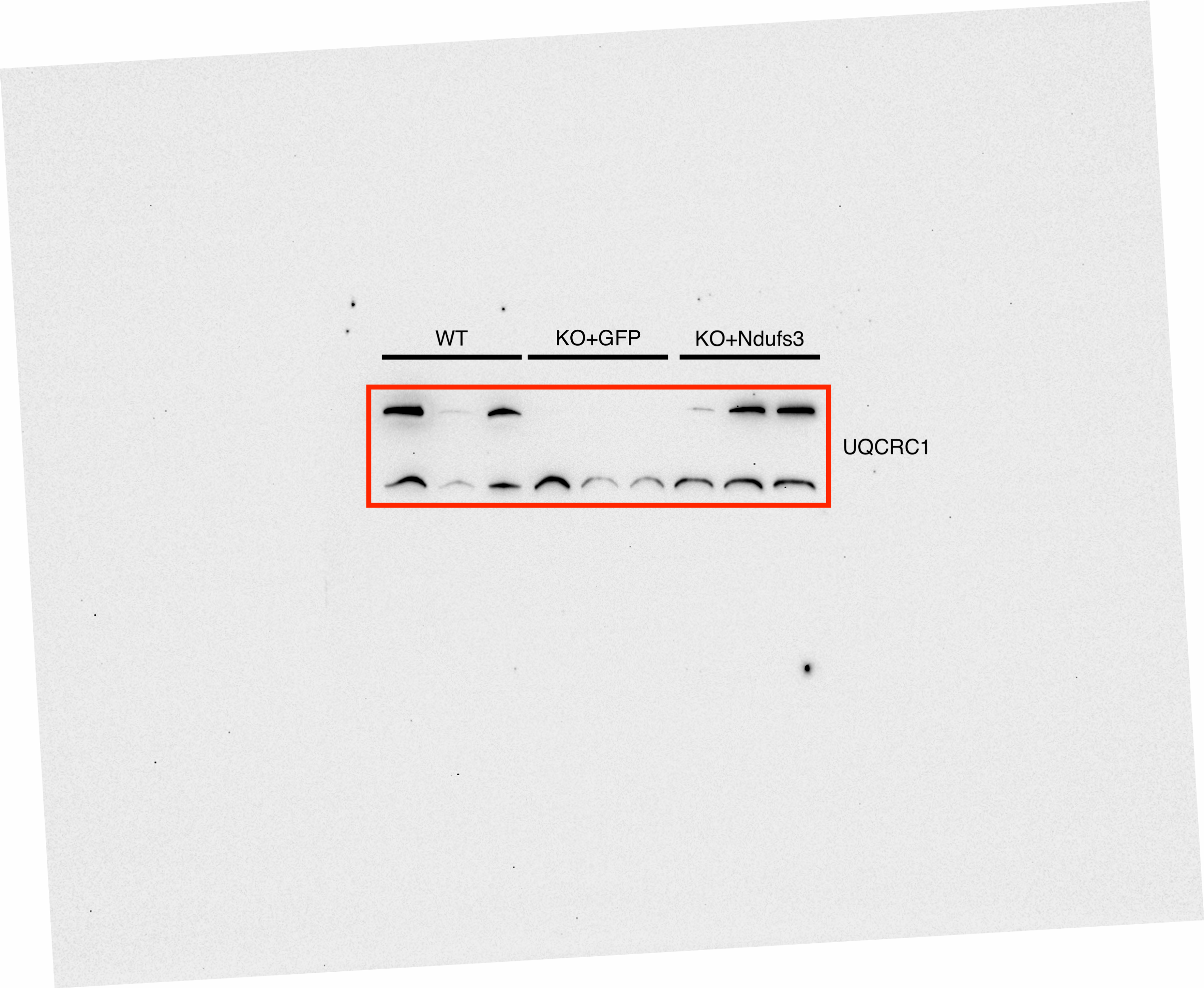

Supplement: Supplementary file 6 — Source data Fig. 5 [file 44321_2024_111_MOESM6_ESM.zip › EMM-2024-19843_SourceData-Figure5/5A/western UQCRC1.tiff]

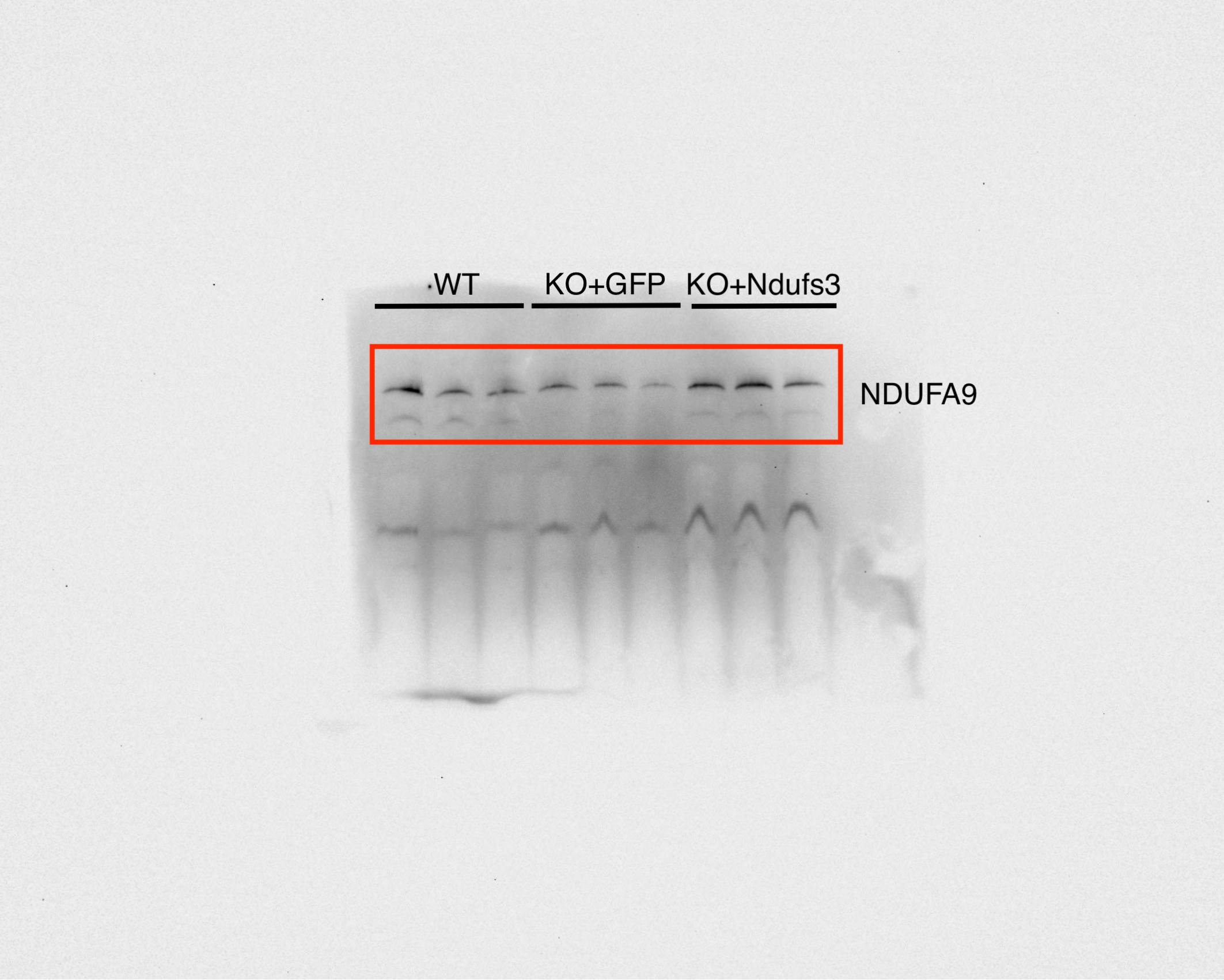

Supplement: Supplementary file 6 — Source data Fig. 5 [file 44321_2024_111_MOESM6_ESM.zip › EMM-2024-19843_SourceData-Figure5/5A/western NDUFA9.tiff]

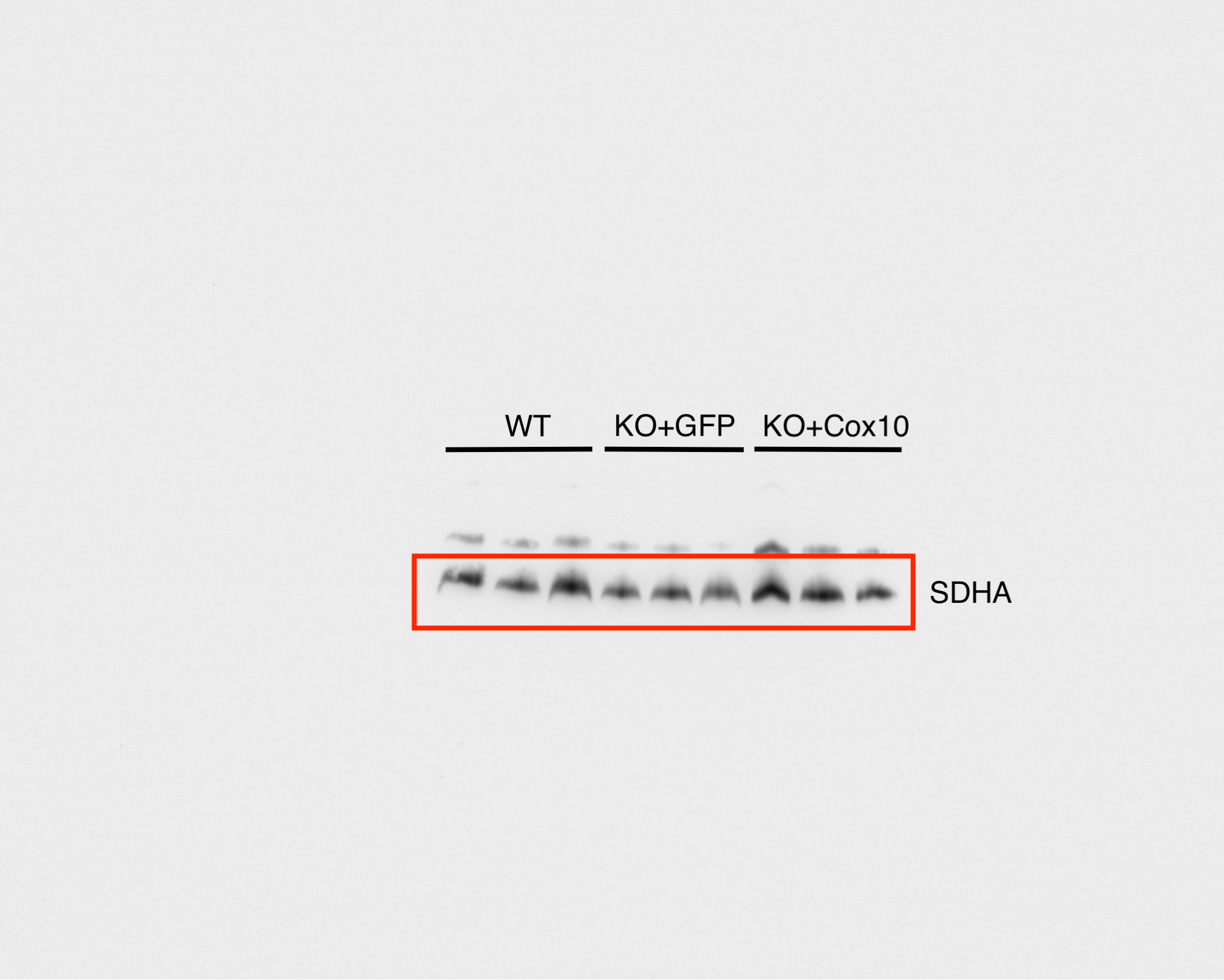

Supplement: Supplementary file 6 — Source data Fig. 5 [file 44321_2024_111_MOESM6_ESM.zip › EMM-2024-19843_SourceData-Figure5/5E/western SDHA.tiff]

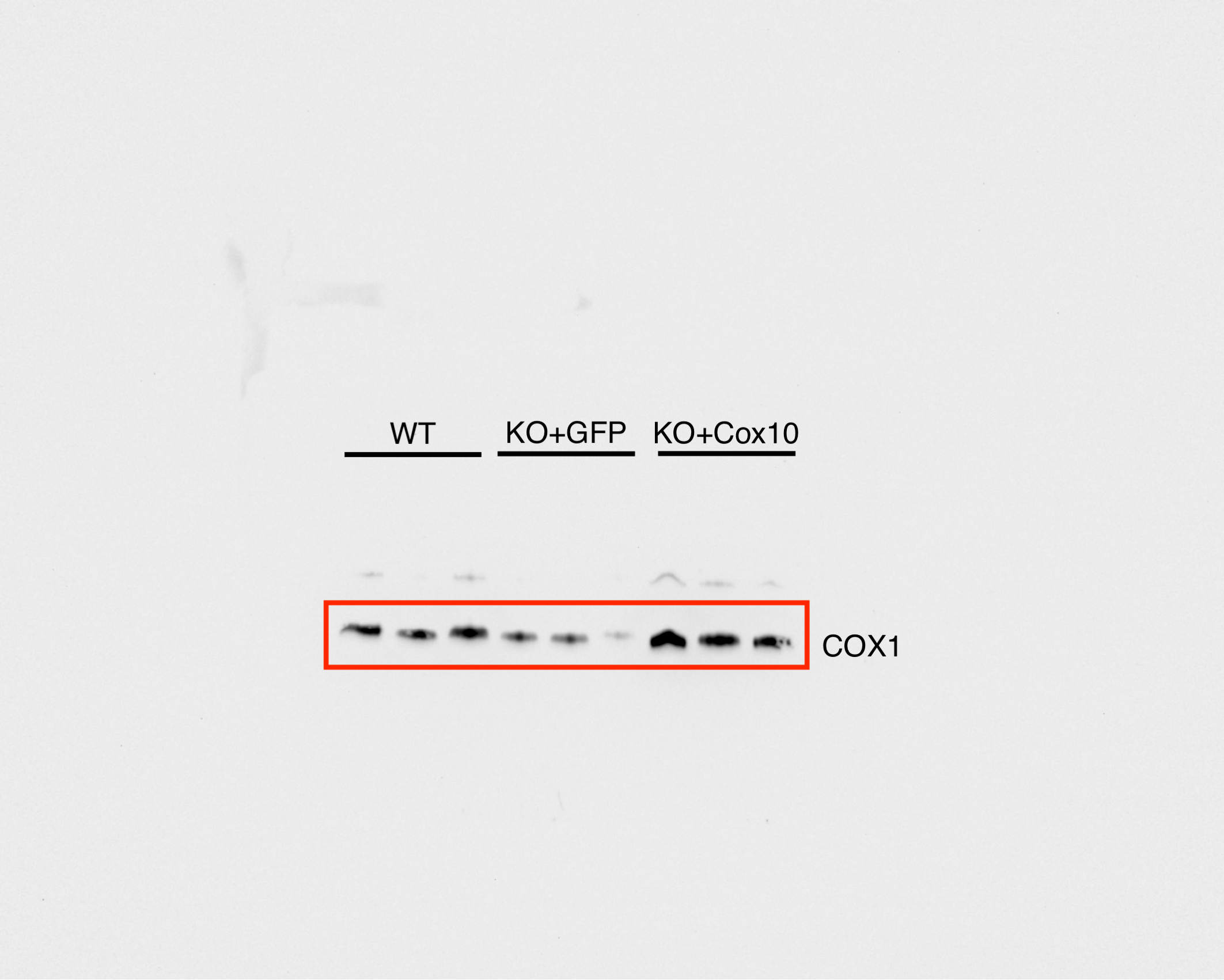

Supplement: Supplementary file 6 — Source data Fig. 5 [file 44321_2024_111_MOESM6_ESM.zip › EMM-2024-19843_SourceData-Figure5/5E/western COX1.tiff]

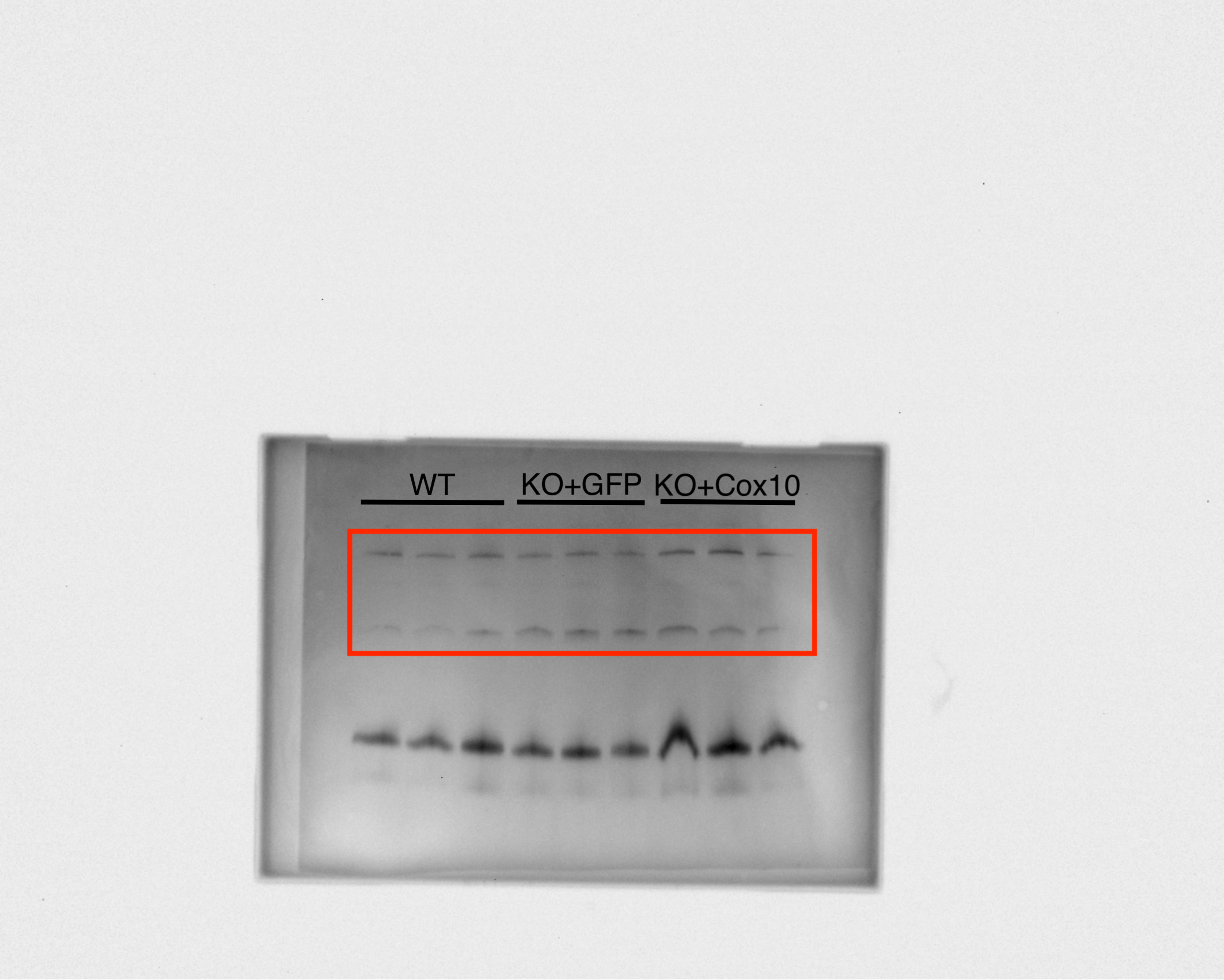

Supplement: Supplementary file 6 — Source data Fig. 5 [file 44321_2024_111_MOESM6_ESM.zip › EMM-2024-19843_SourceData-Figure5/5E/western UQCRC1.tiff]

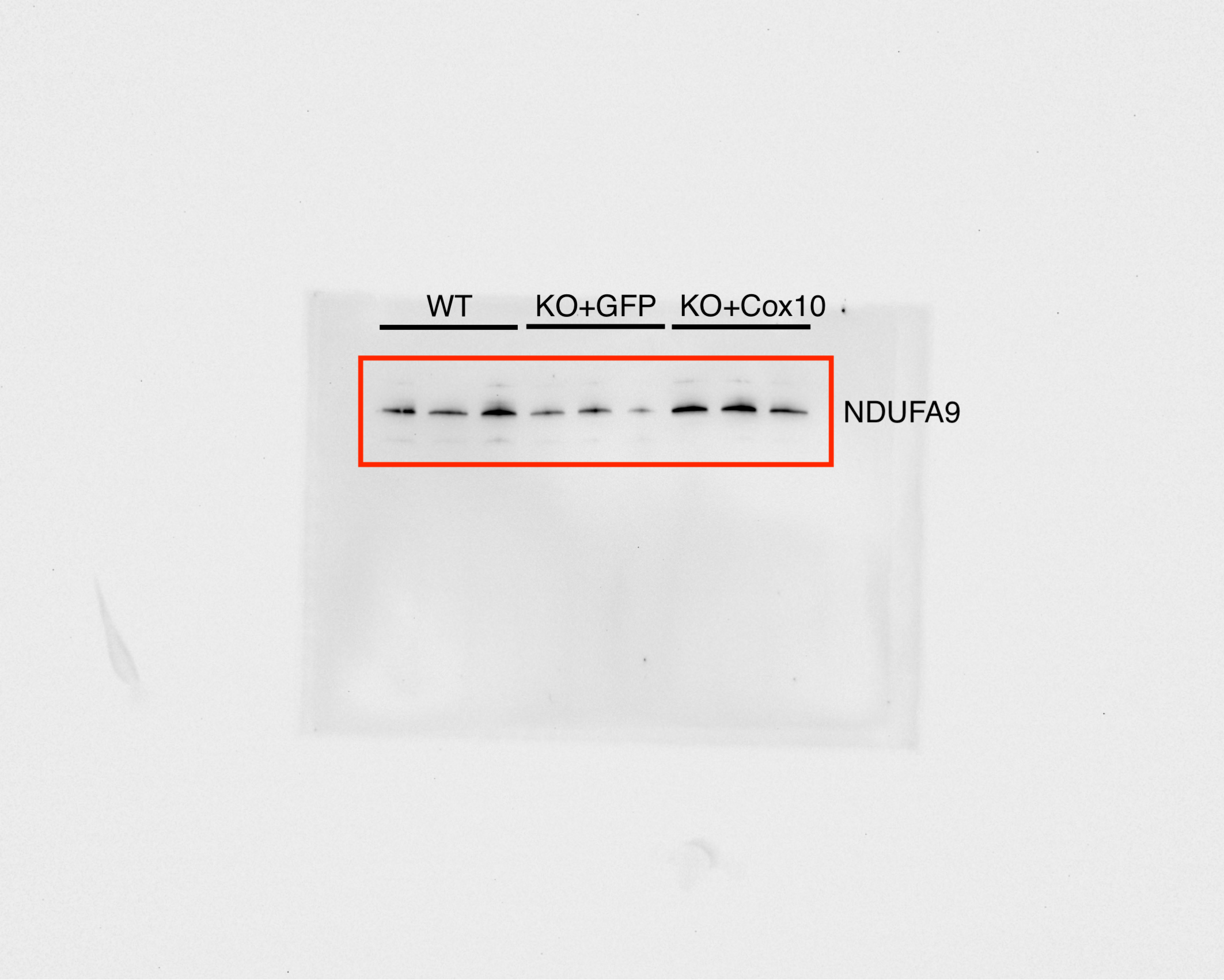

Supplement: Supplementary file 6 — Source data Fig. 5 [file 44321_2024_111_MOESM6_ESM.zip › EMM-2024-19843_SourceData-Figure5/5E/western NDUFA9.tiff]

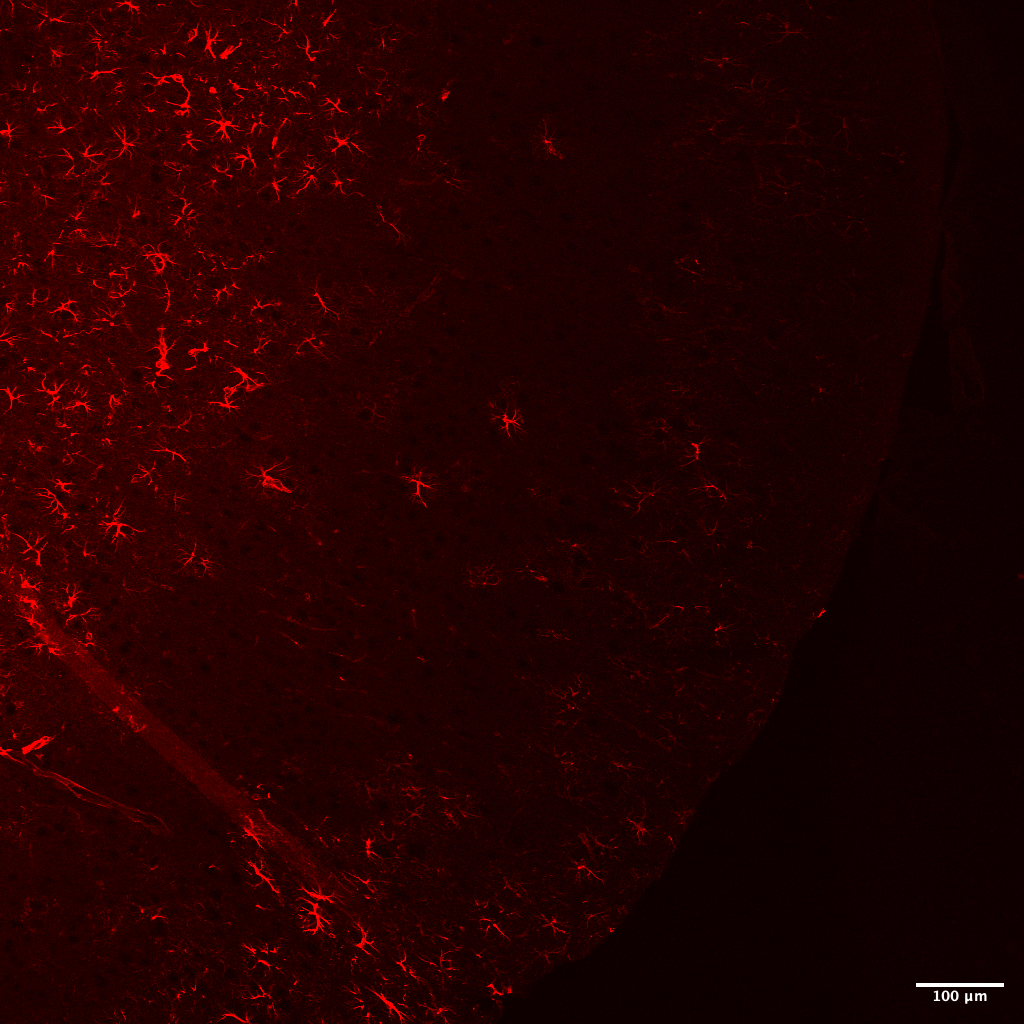

Supplement: Supplementary file 7 — Source data Fig. 6 [file 44321_2024_111_MOESM7_ESM.zip › EMM-2024-19843_SourceData-Figure6/6A/GFAP IHC - KO+NDUFS3.tiff]

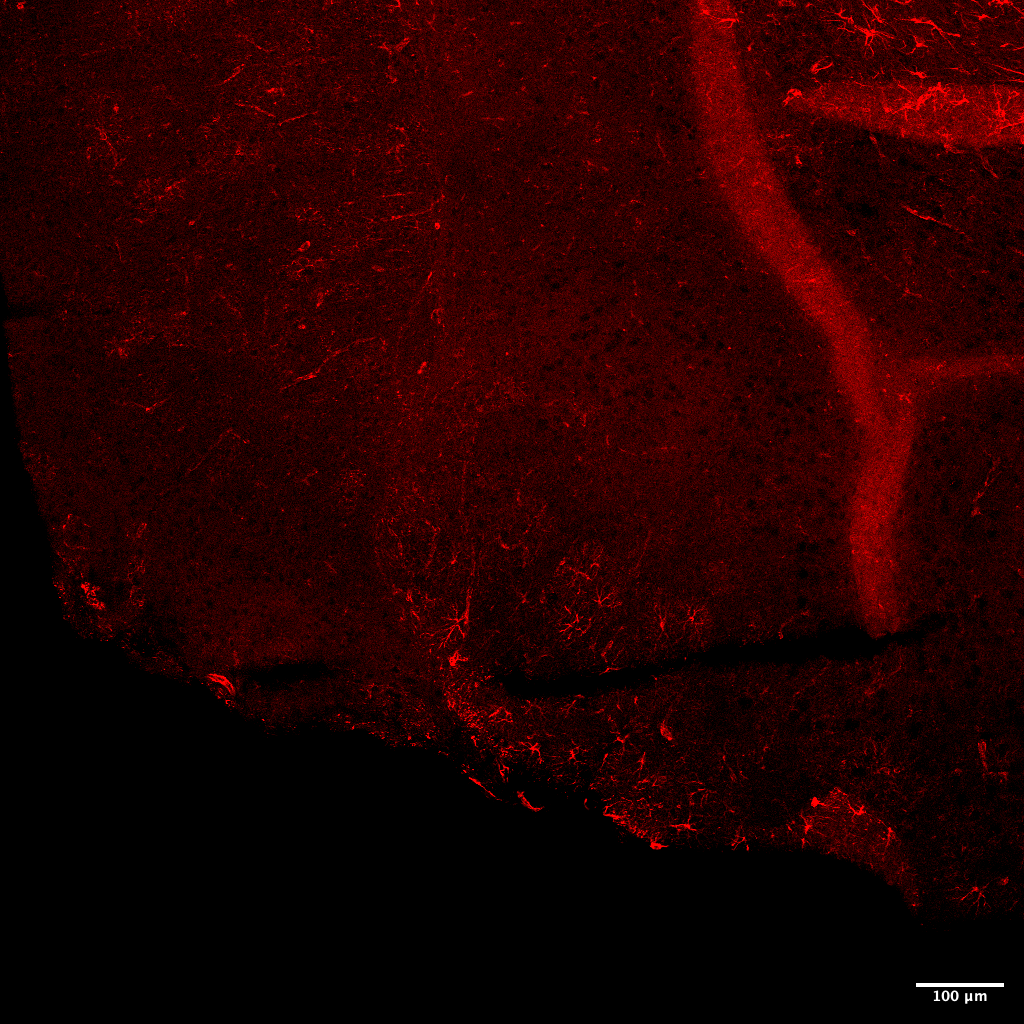

Supplement: Supplementary file 7 — Source data Fig. 6 [file 44321_2024_111_MOESM7_ESM.zip › EMM-2024-19843_SourceData-Figure6/6A/GFAP IHC - WT.tiff]

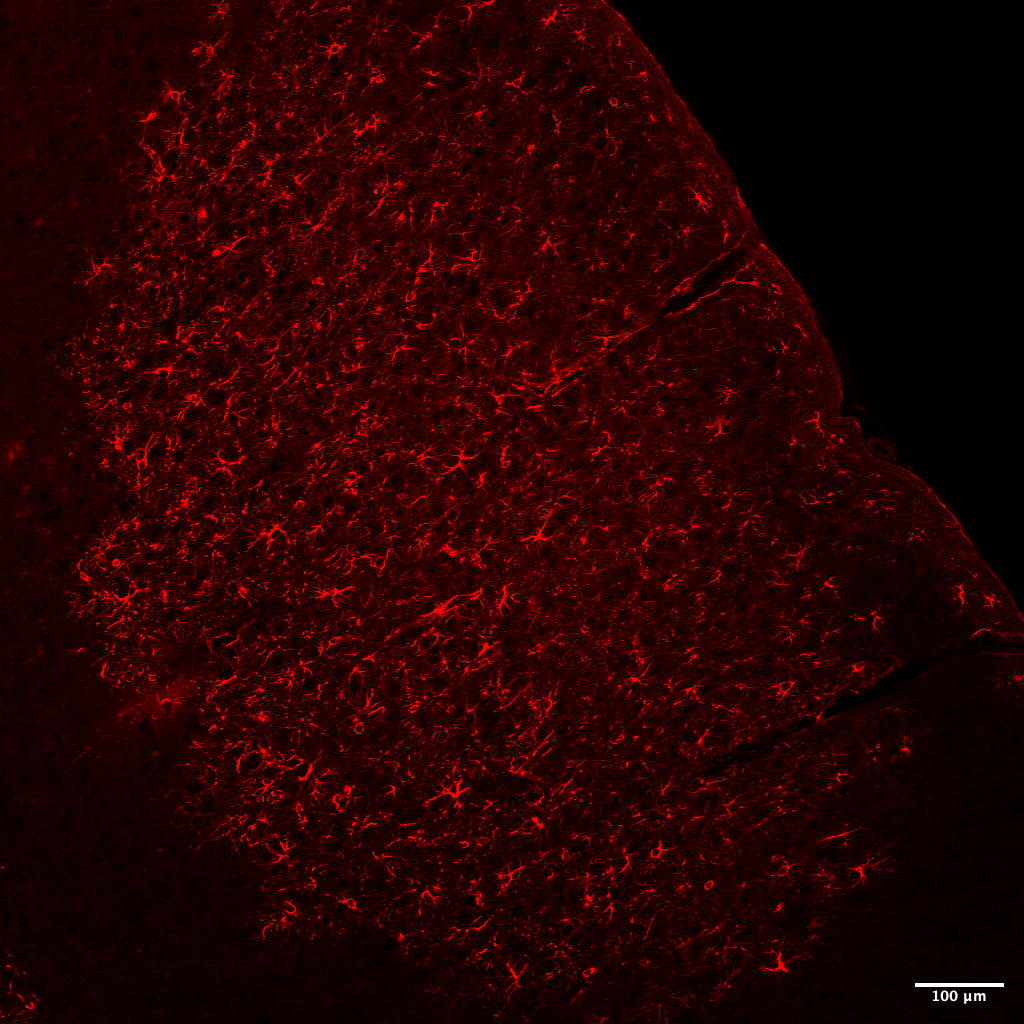

Supplement: Supplementary file 7 — Source data Fig. 6 [file 44321_2024_111_MOESM7_ESM.zip › EMM-2024-19843_SourceData-Figure6/6A/GFAP IHC - KO+GFP.tiff]

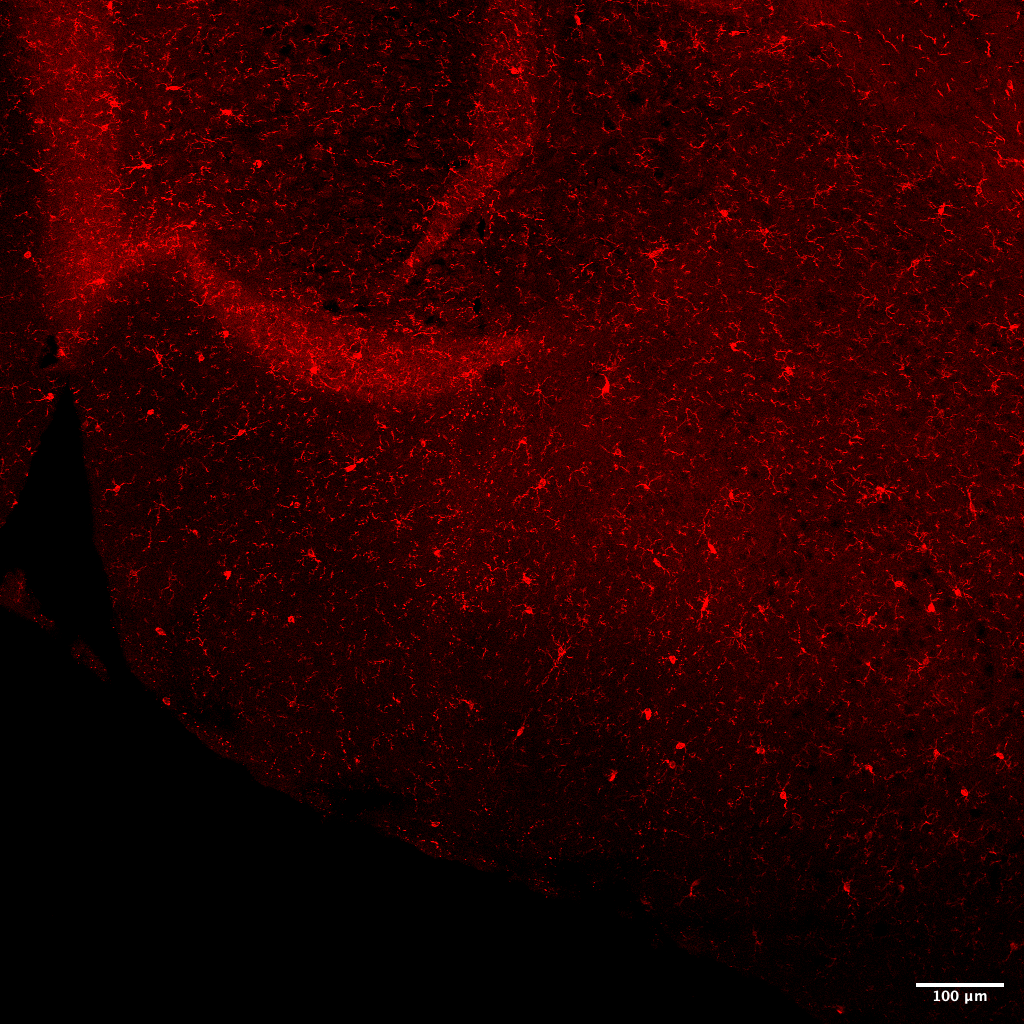

Supplement: Supplementary file 7 — Source data Fig. 6 [file 44321_2024_111_MOESM7_ESM.zip › EMM-2024-19843_SourceData-Figure6/6B/Iba1 IHC - WT.tiff]

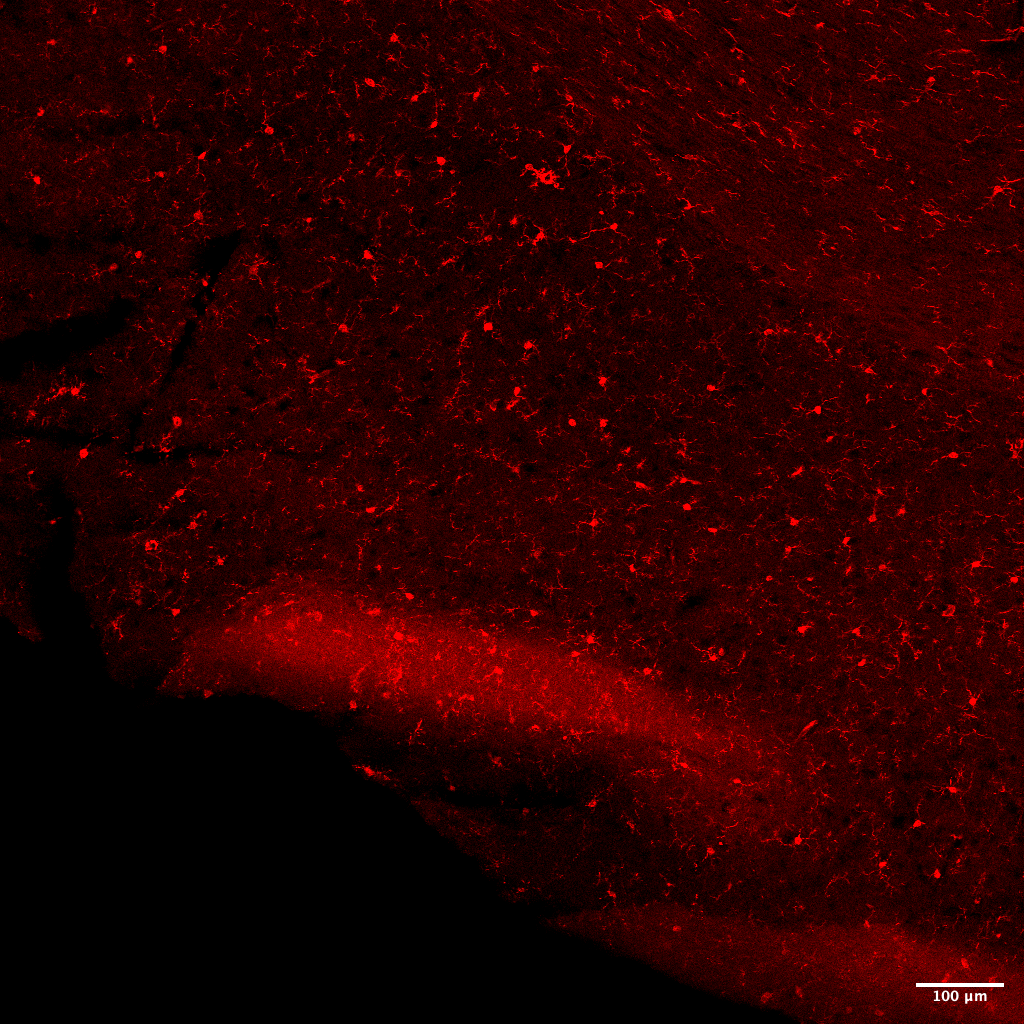

Supplement: Supplementary file 7 — Source data Fig. 6 [file 44321_2024_111_MOESM7_ESM.zip › EMM-2024-19843_SourceData-Figure6/6B/Iba1 IHC - KO+NDUFS3.tiff]

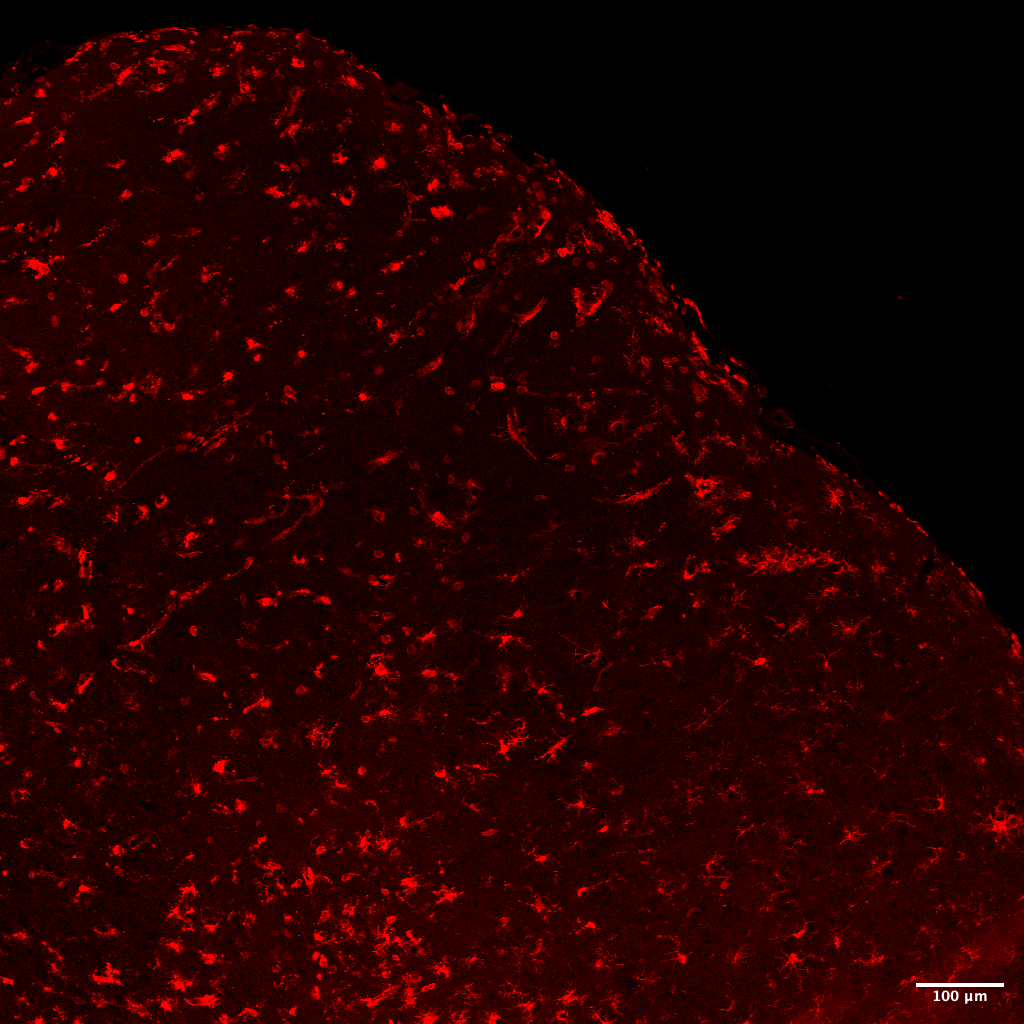

Supplement: Supplementary file 7 — Source data Fig. 6 [file 44321_2024_111_MOESM7_ESM.zip › EMM-2024-19843_SourceData-Figure6/6B/Iba1 IHC - KO+GFP.tiff]

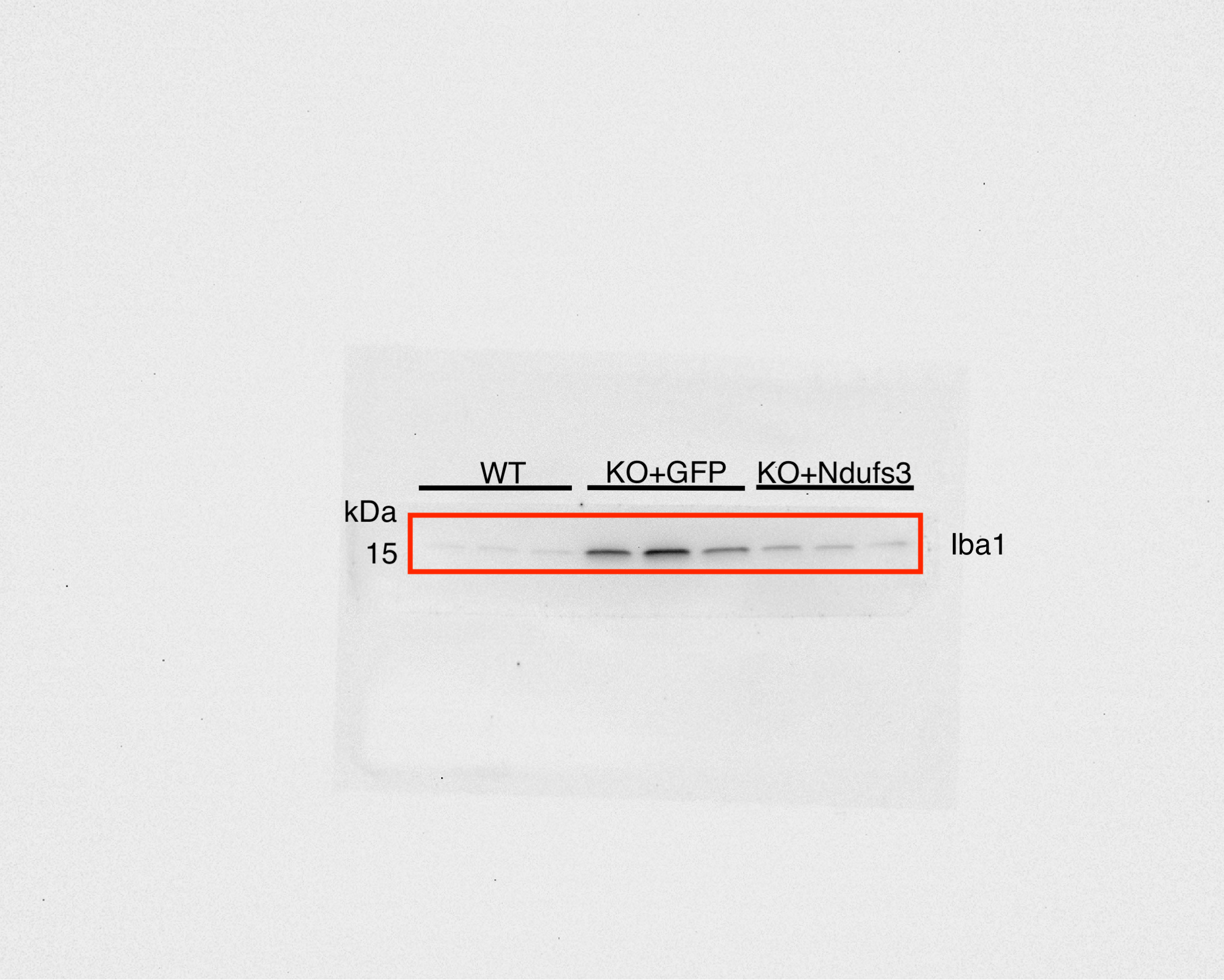

Supplement: Supplementary file 7 — Source data Fig. 6 [file 44321_2024_111_MOESM7_ESM.zip › EMM-2024-19843_SourceData-Figure6/6C/Hippocampus/western Iba1.tiff]

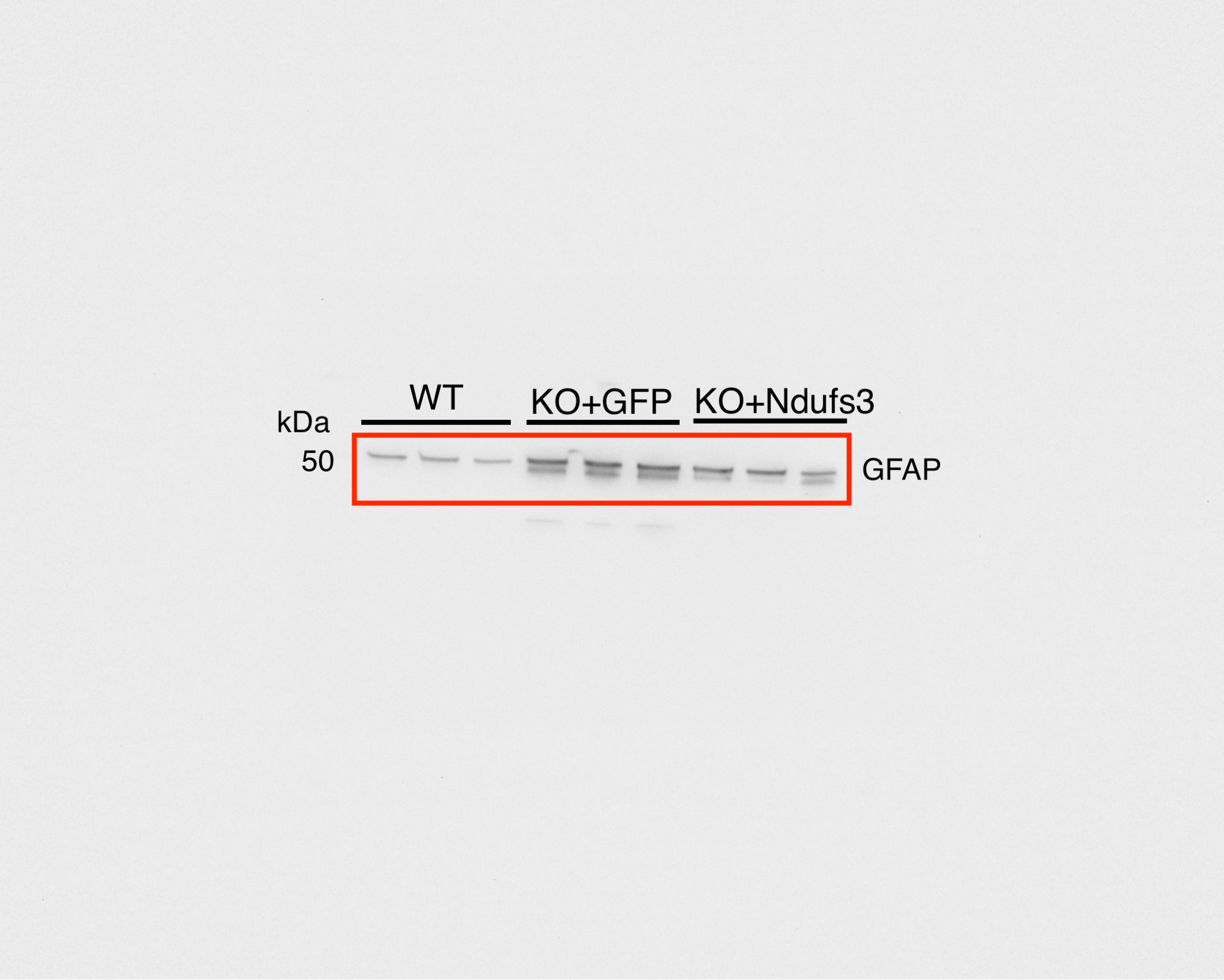

Supplement: Supplementary file 7 — Source data Fig. 6 [file 44321_2024_111_MOESM7_ESM.zip › EMM-2024-19843_SourceData-Figure6/6C/Hippocampus/western GFAP.tiff]

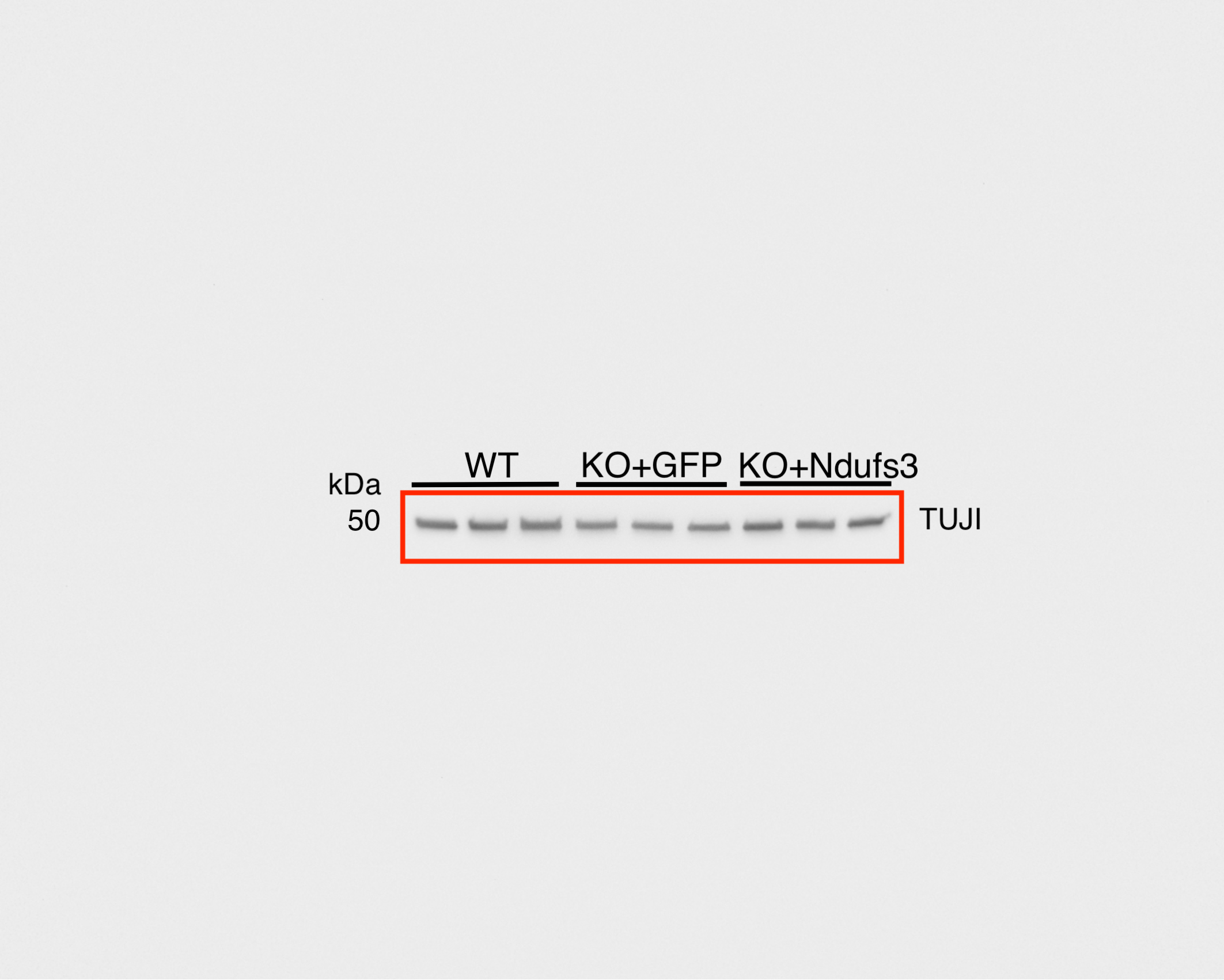

Supplement: Supplementary file 7 — Source data Fig. 6 [file 44321_2024_111_MOESM7_ESM.zip › EMM-2024-19843_SourceData-Figure6/6C/Hippocampus/western TUJI.tiff]

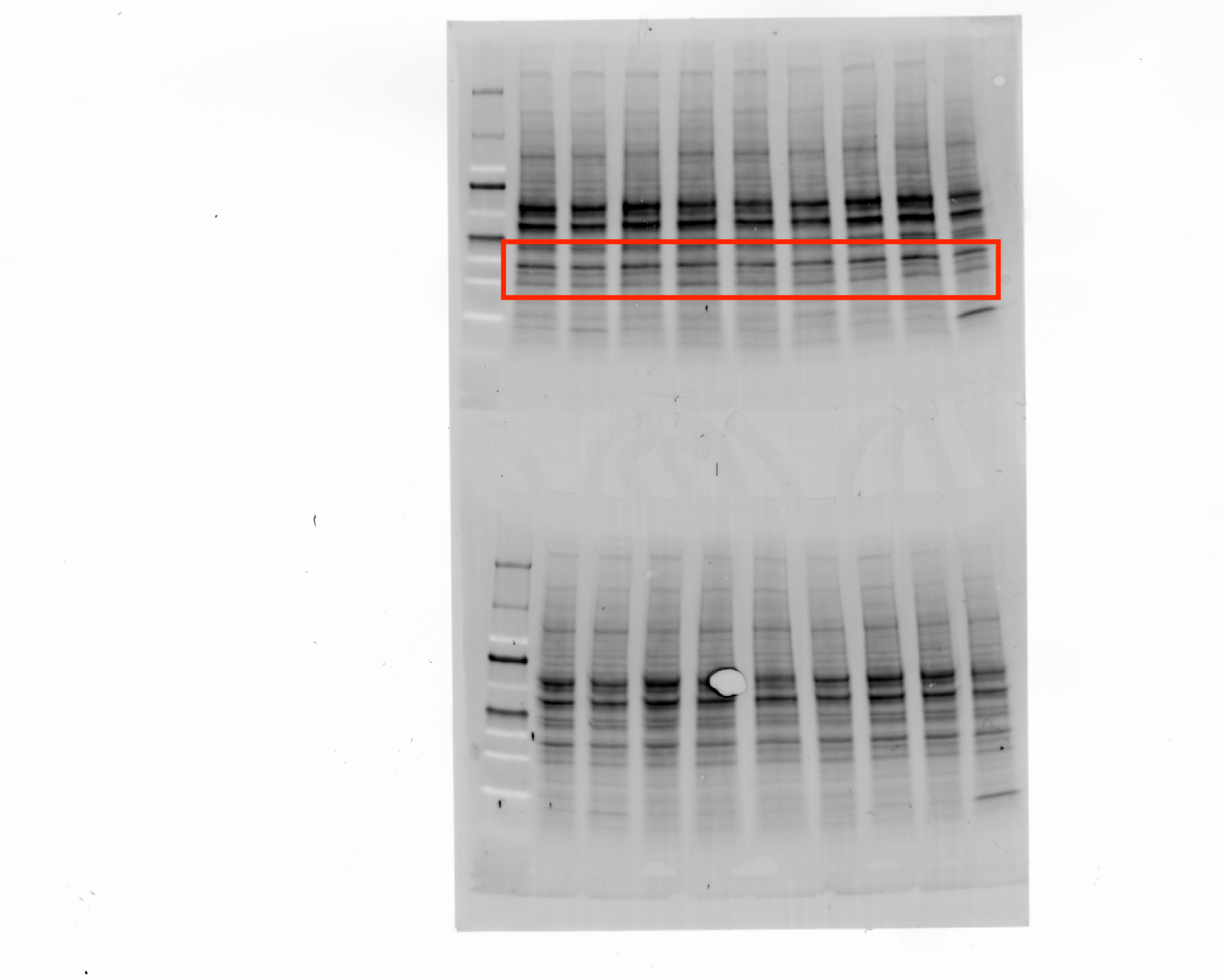

Supplement: Supplementary file 7 — Source data Fig. 6 [file 44321_2024_111_MOESM7_ESM.zip › EMM-2024-19843_SourceData-Figure6/6C/Hippocampus/western Total Protein.tiff]

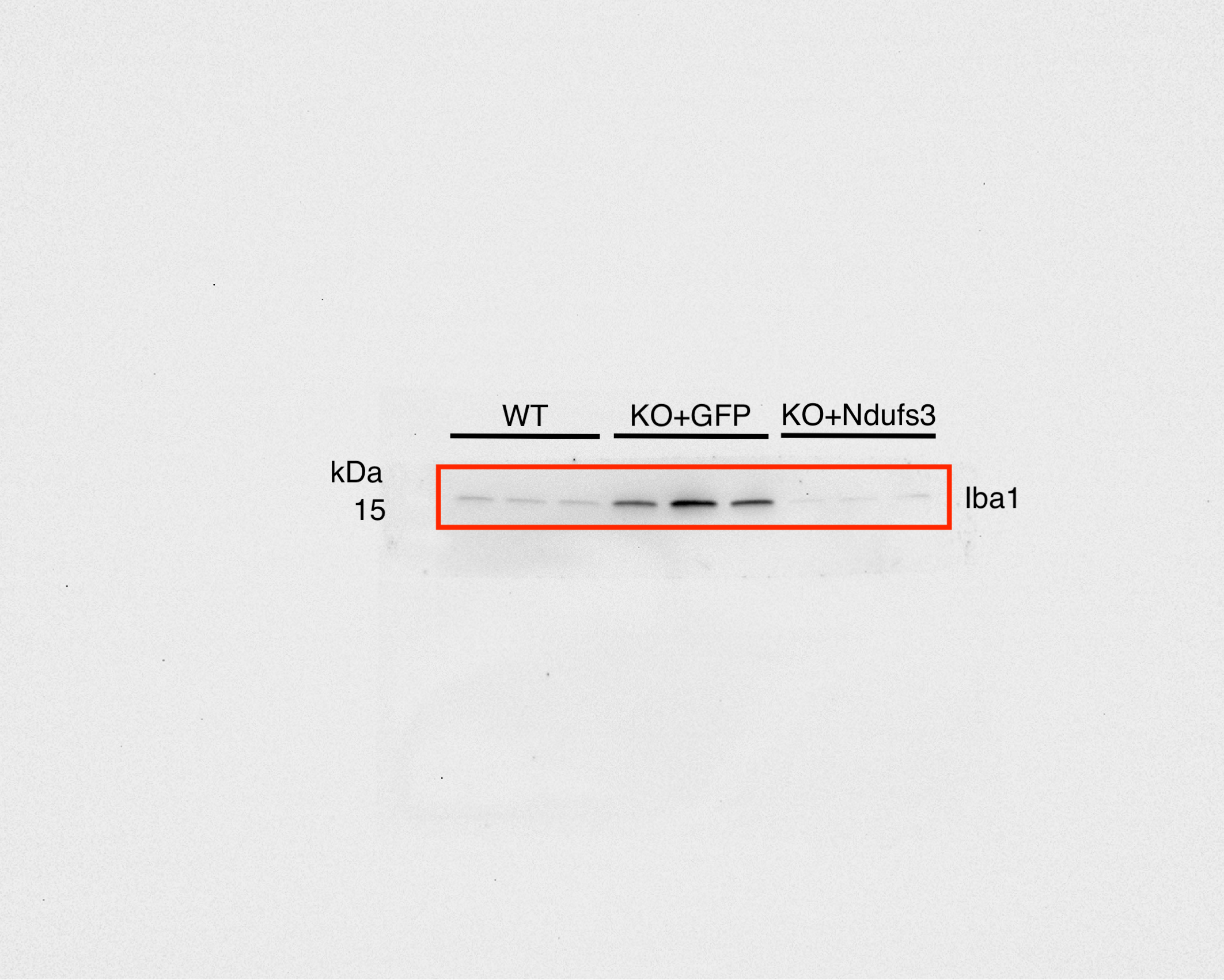

Supplement: Supplementary file 7 — Source data Fig. 6 [file 44321_2024_111_MOESM7_ESM.zip › EMM-2024-19843_SourceData-Figure6/6C/Cortex/western Iba1.tiff]

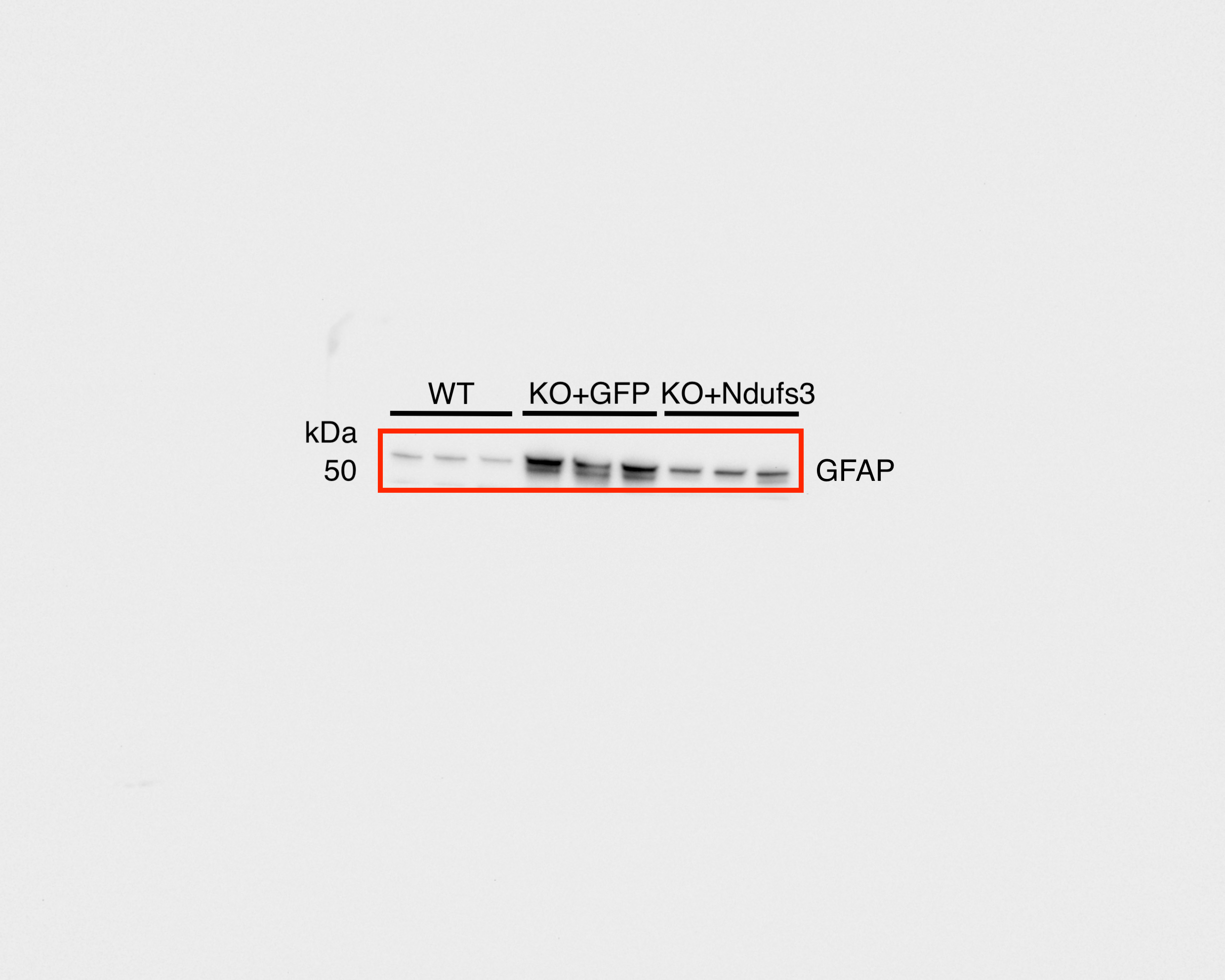

Supplement: Supplementary file 7 — Source data Fig. 6 [file 44321_2024_111_MOESM7_ESM.zip › EMM-2024-19843_SourceData-Figure6/6C/Cortex/western GFAP.tiff]

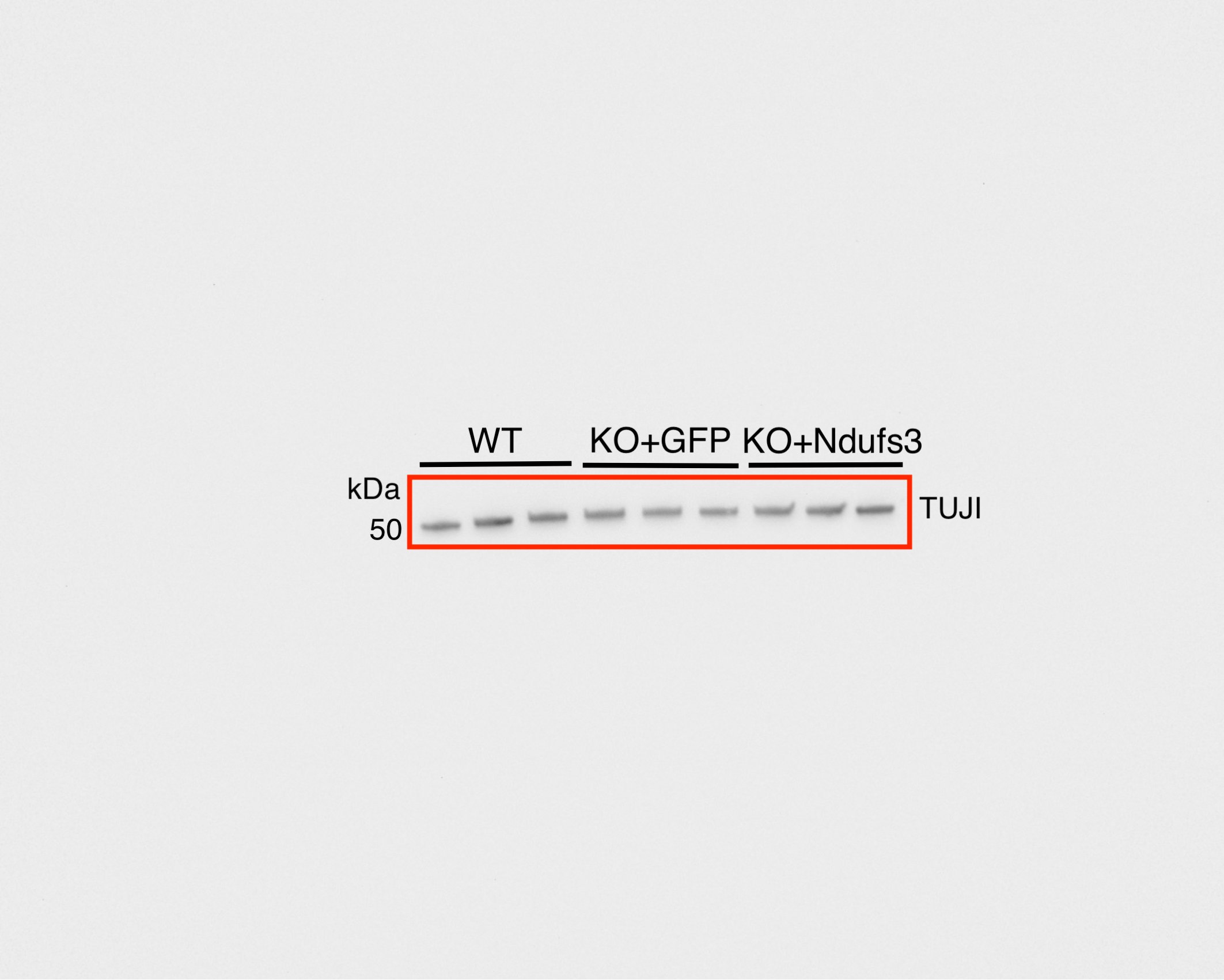

Supplement: Supplementary file 7 — Source data Fig. 6 [file 44321_2024_111_MOESM7_ESM.zip › EMM-2024-19843_SourceData-Figure6/6C/Cortex/western TUJI.tiff]

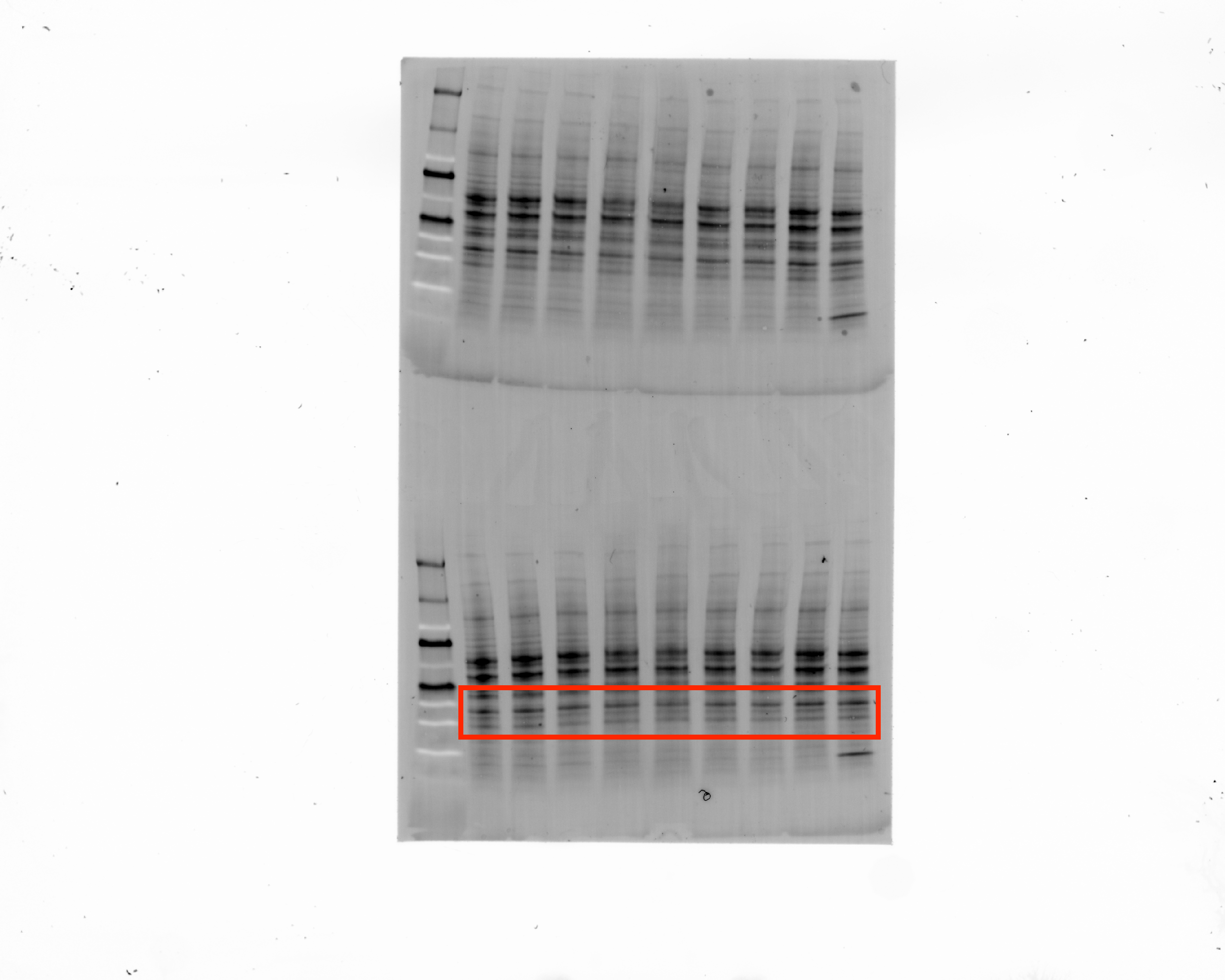

Supplement: Supplementary file 7 — Source data Fig. 6 [file 44321_2024_111_MOESM7_ESM.zip › EMM-2024-19843_SourceData-Figure6/6C/Cortex/western Total Protein.tiff]

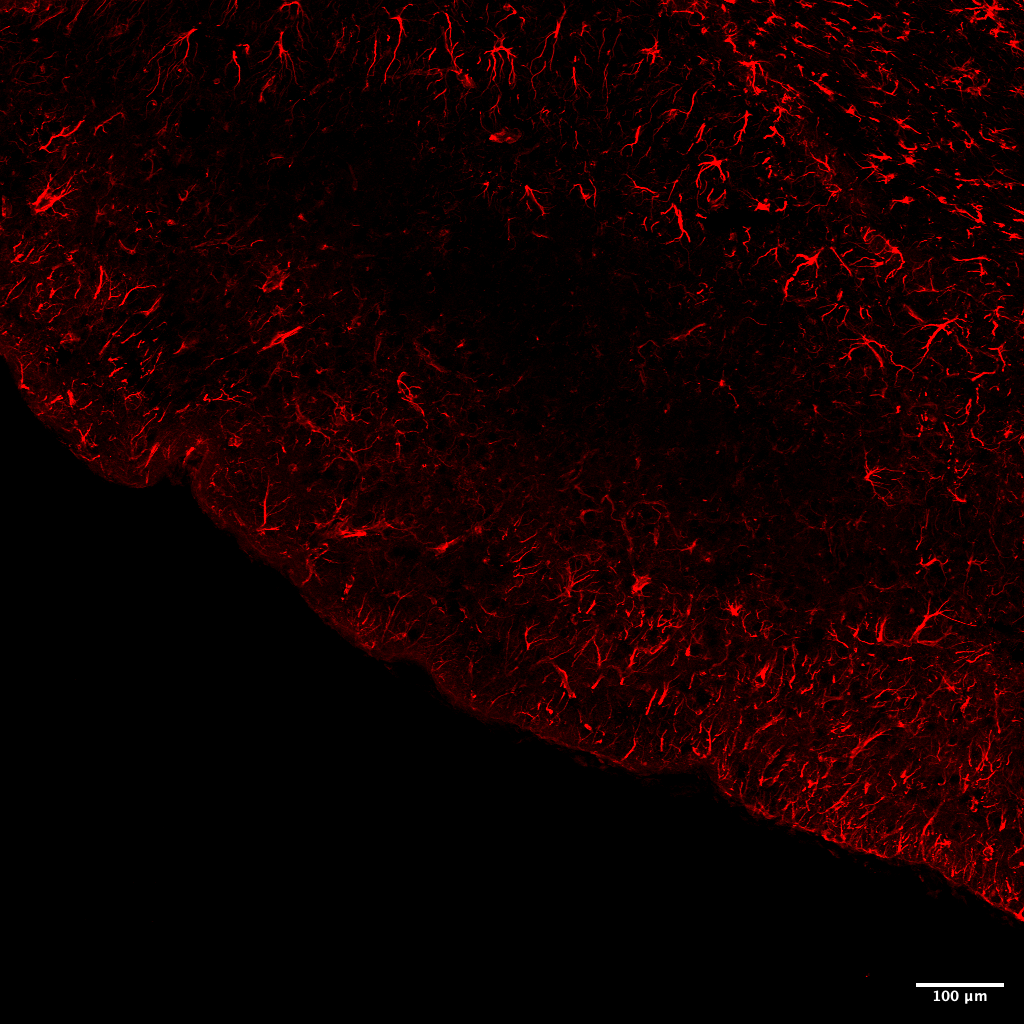

Supplement: Supplementary file 8 — Source data Fig. 7 [file 44321_2024_111_MOESM8_ESM.zip › EMM-2024-19843_SourceData-Figure7/7C/GFAP IHC - KO+COX10.tiff]

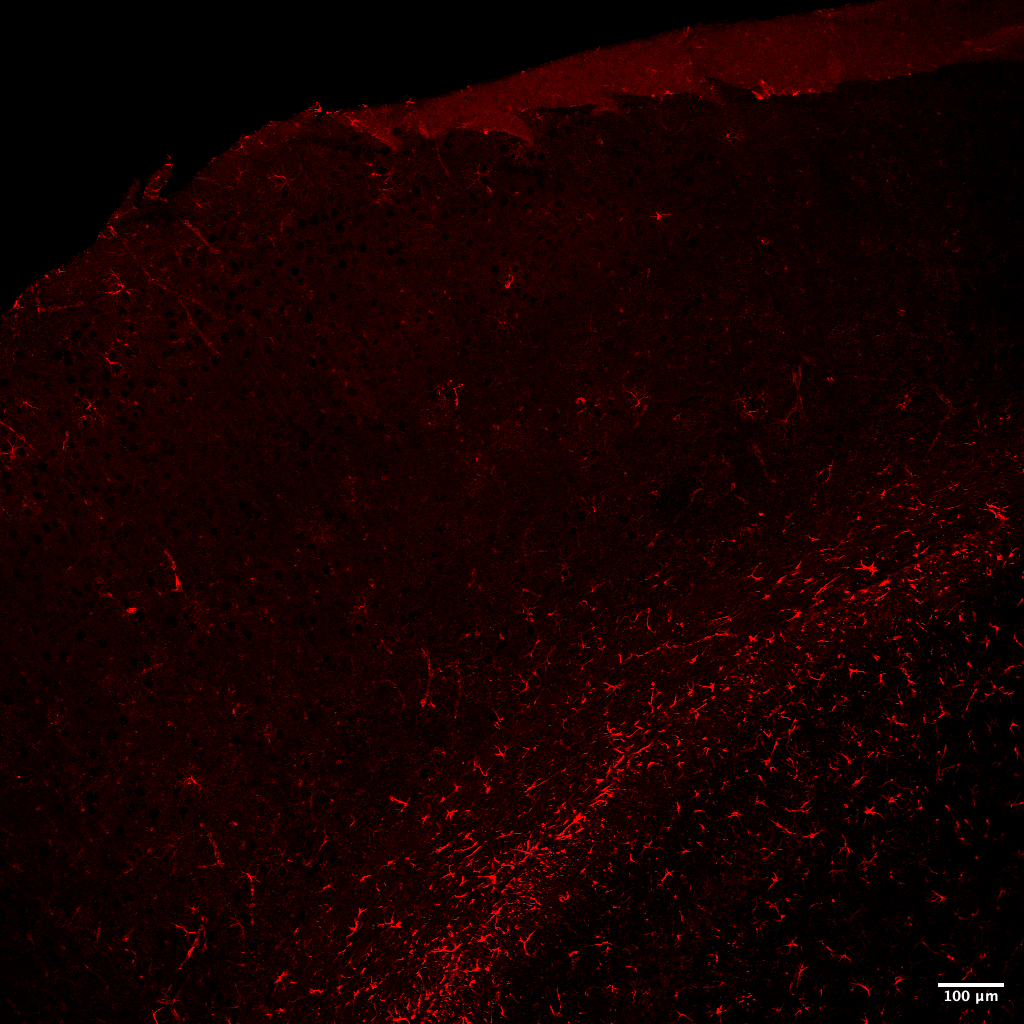

Supplement: Supplementary file 8 — Source data Fig. 7 [file 44321_2024_111_MOESM8_ESM.zip › EMM-2024-19843_SourceData-Figure7/7C/GFAP IHC - WT.tiff]

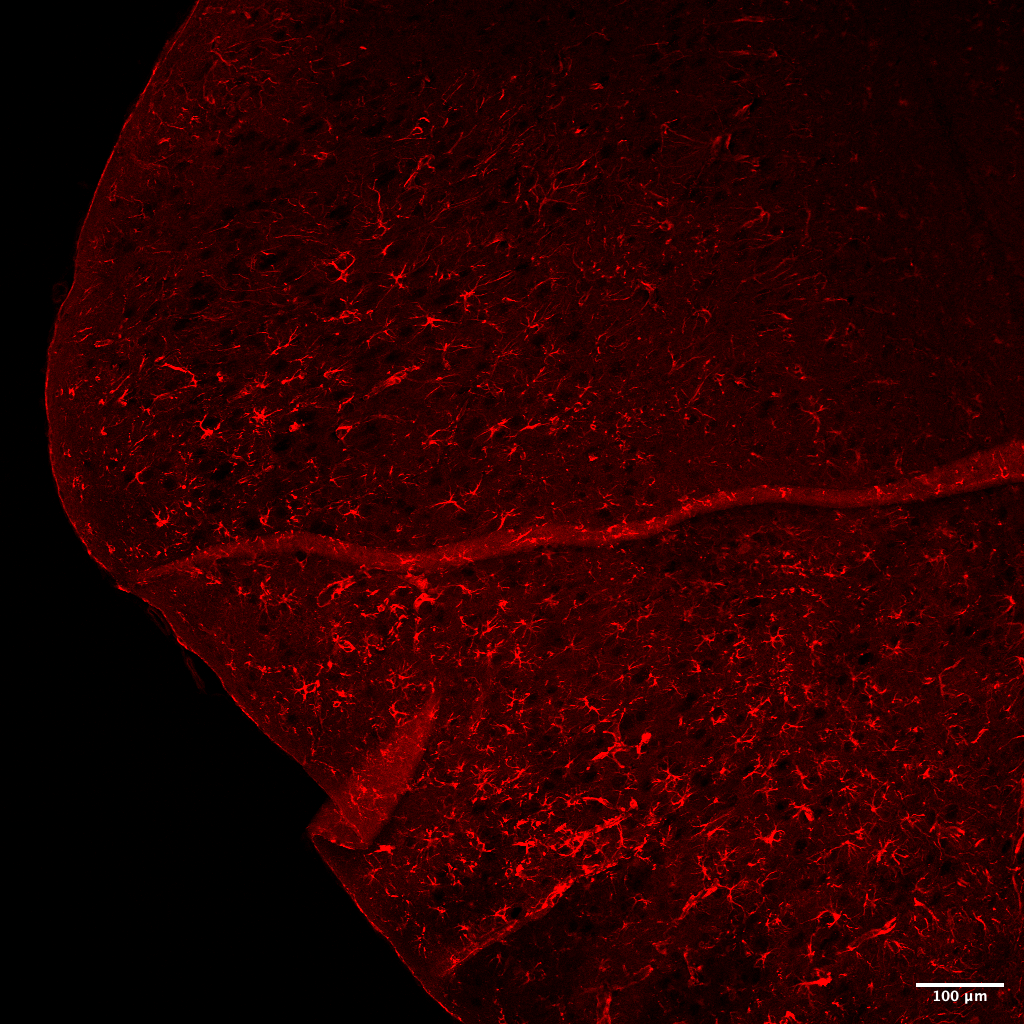

Supplement: Supplementary file 8 — Source data Fig. 7 [file 44321_2024_111_MOESM8_ESM.zip › EMM-2024-19843_SourceData-Figure7/7C/GFAP IHC - KO+GFP.tiff]

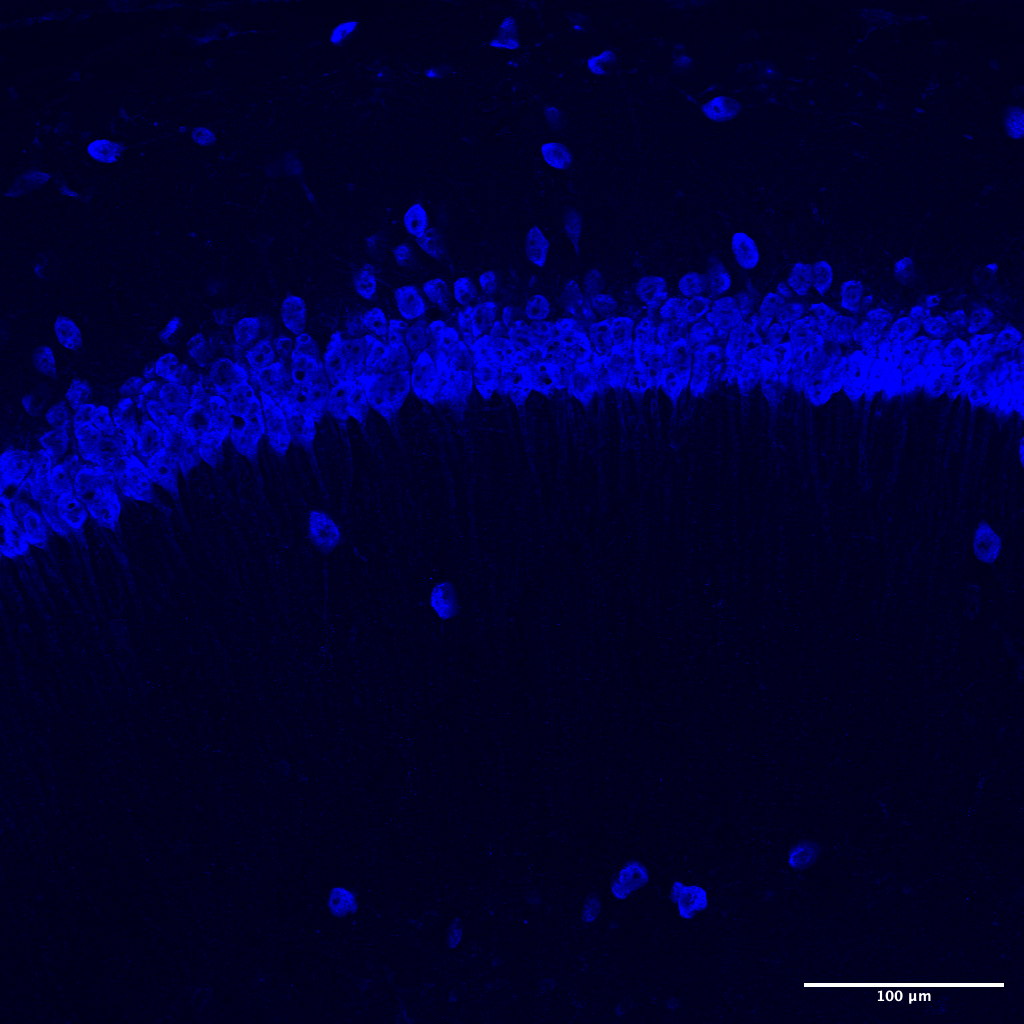

Supplement: Supplementary file 8 — Source data Fig. 7 [file 44321_2024_111_MOESM8_ESM.zip › EMM-2024-19843_SourceData-Figure7/7G/NeuN IHC - WT.tiff]

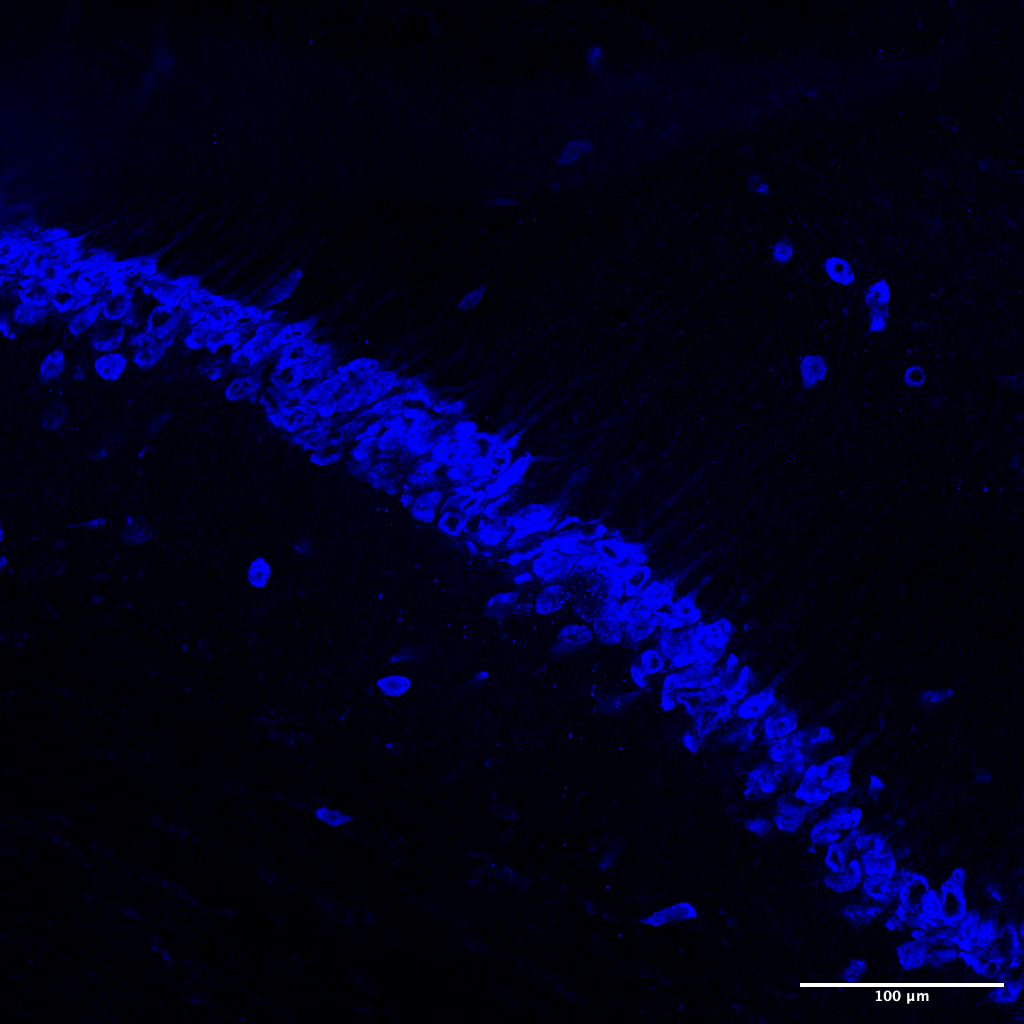

Supplement: Supplementary file 8 — Source data Fig. 7 [file 44321_2024_111_MOESM8_ESM.zip › EMM-2024-19843_SourceData-Figure7/7G/NeuN IHC - KO+COX10.tiff]

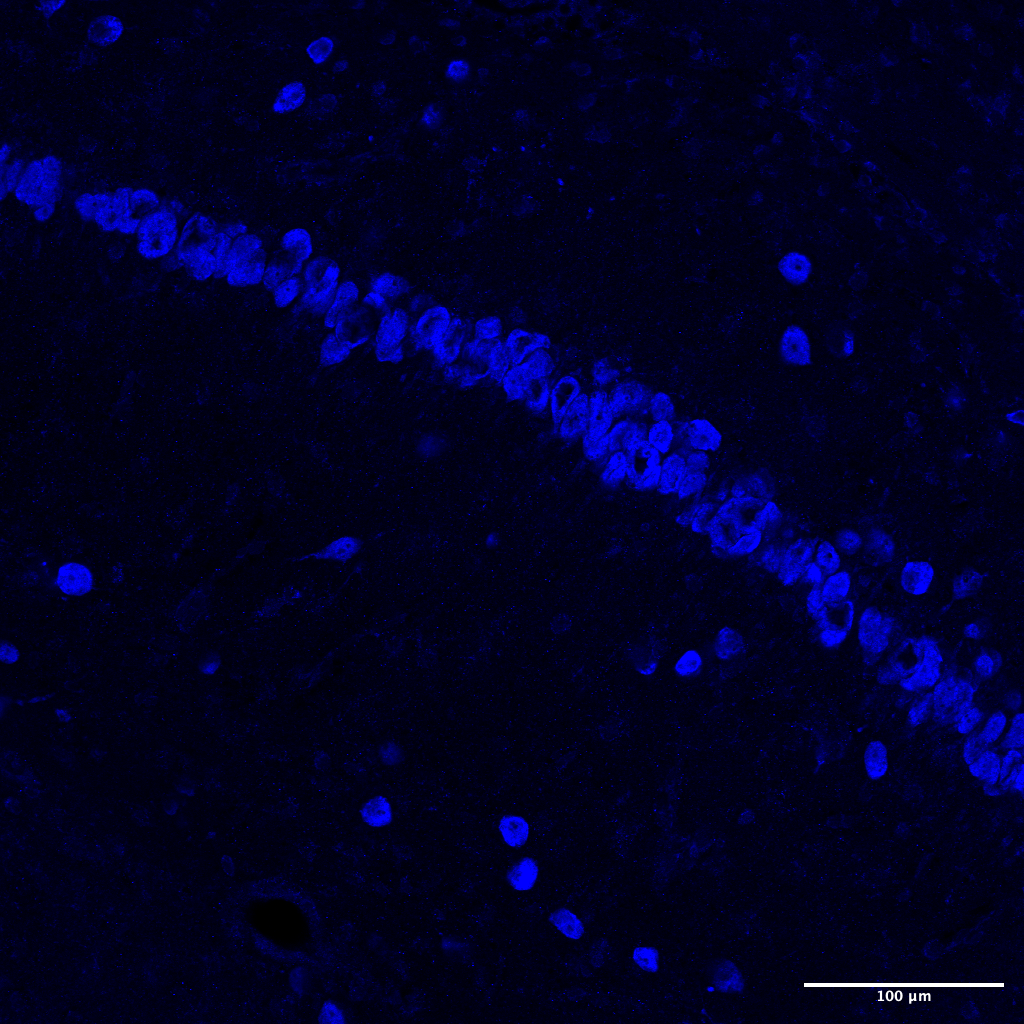

Supplement: Supplementary file 8 — Source data Fig. 7 [file 44321_2024_111_MOESM8_ESM.zip › EMM-2024-19843_SourceData-Figure7/7G/NeuN IHC - KO+GFP.tiff]

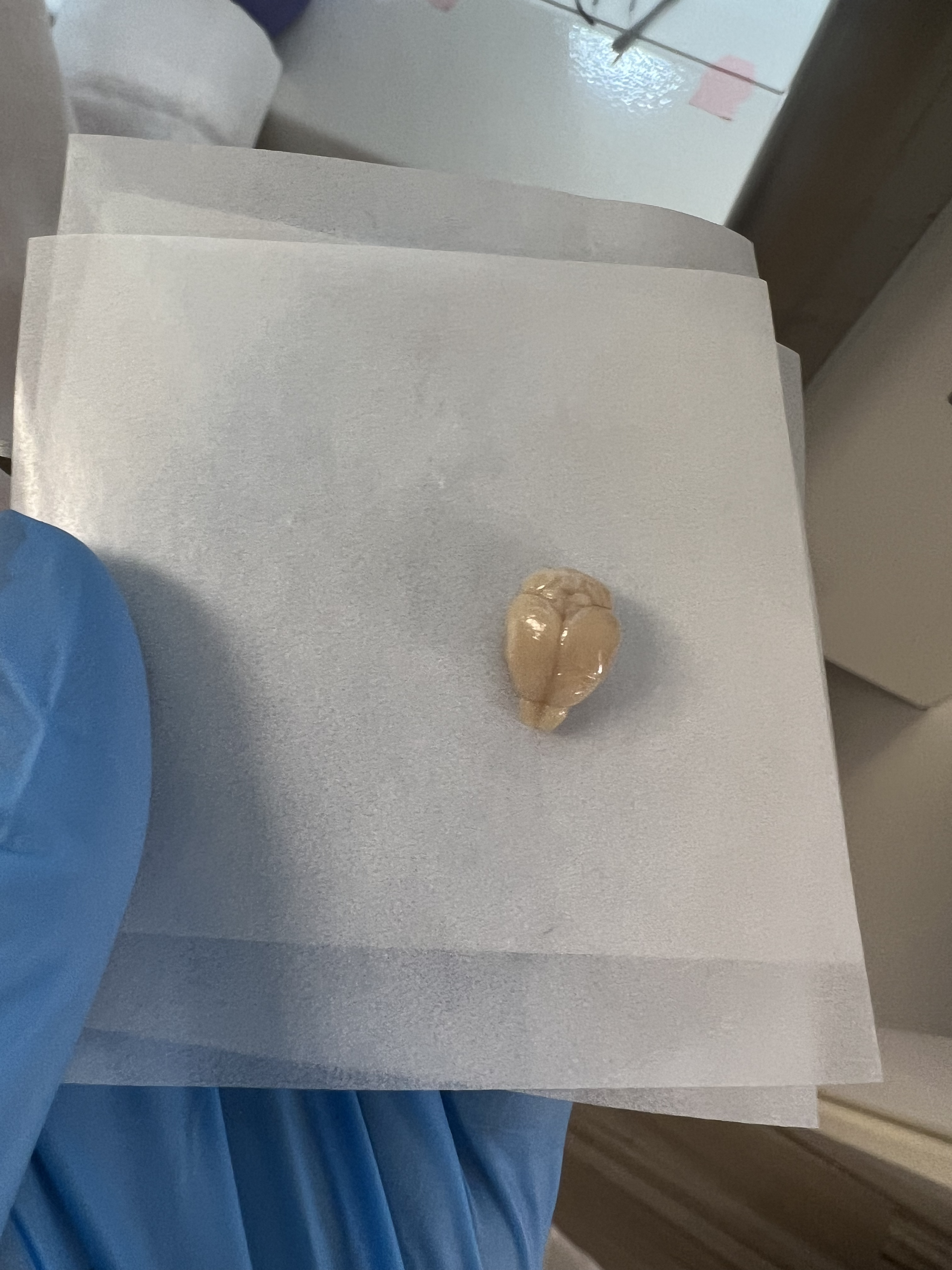

Supplement: Supplementary file 8 — Source data Fig. 7 [file 44321_2024_111_MOESM8_ESM.zip › EMM-2024-19843_SourceData-Figure7/7A/brain - KO+COX10.tiff]

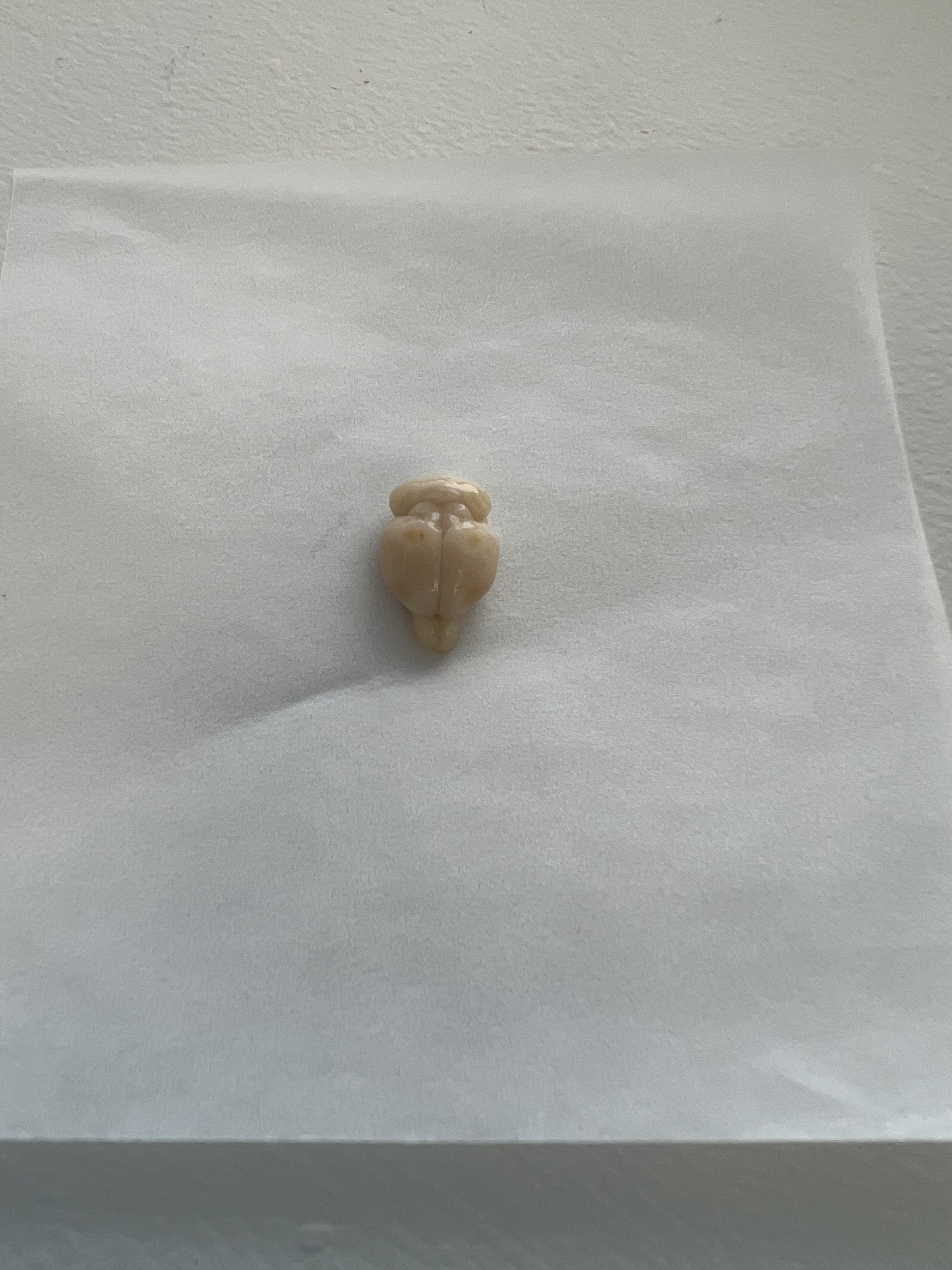

Supplement: Supplementary file 8 — Source data Fig. 7 [file 44321_2024_111_MOESM8_ESM.zip › EMM-2024-19843_SourceData-Figure7/7A/brain - KO+GFP.tiff]

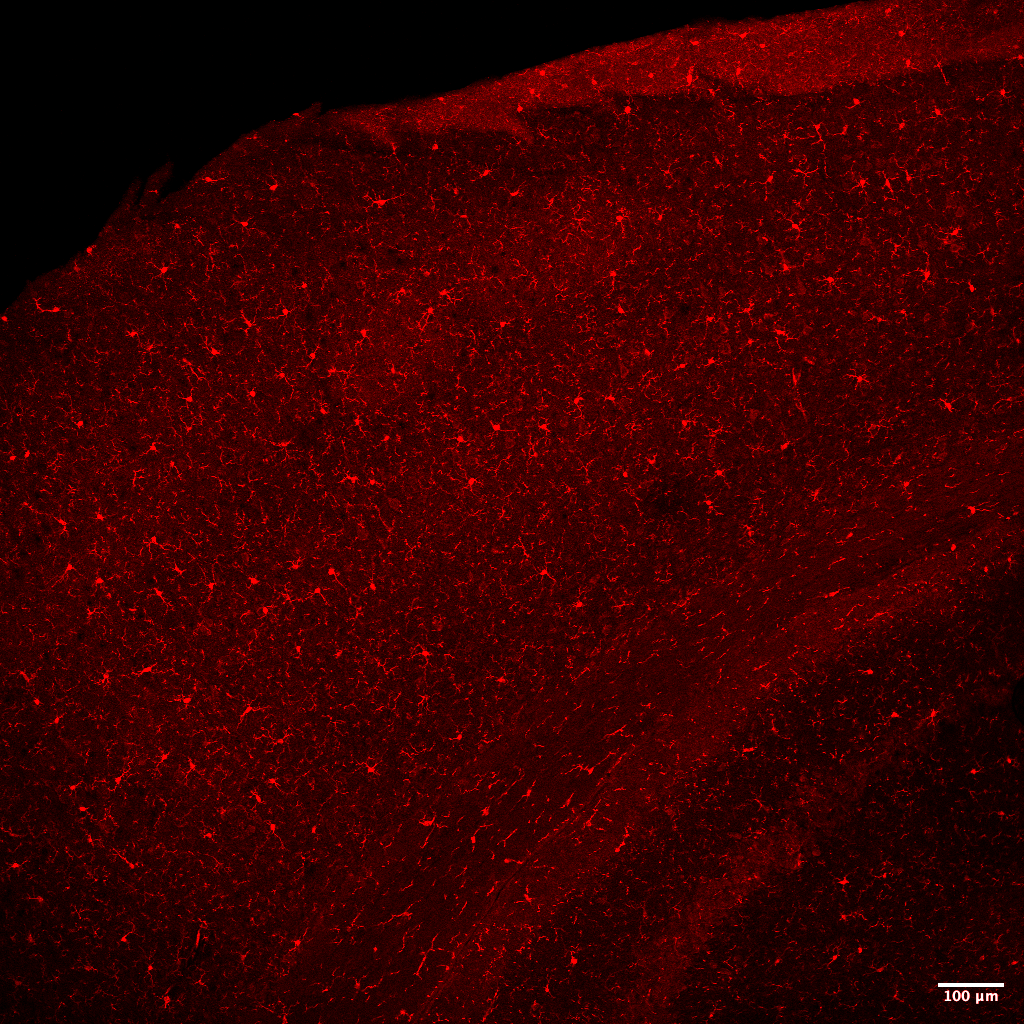

Supplement: Supplementary file 8 — Source data Fig. 7 [file 44321_2024_111_MOESM8_ESM.zip › EMM-2024-19843_SourceData-Figure7/7D/Iba1 IHC - WT.tiff]

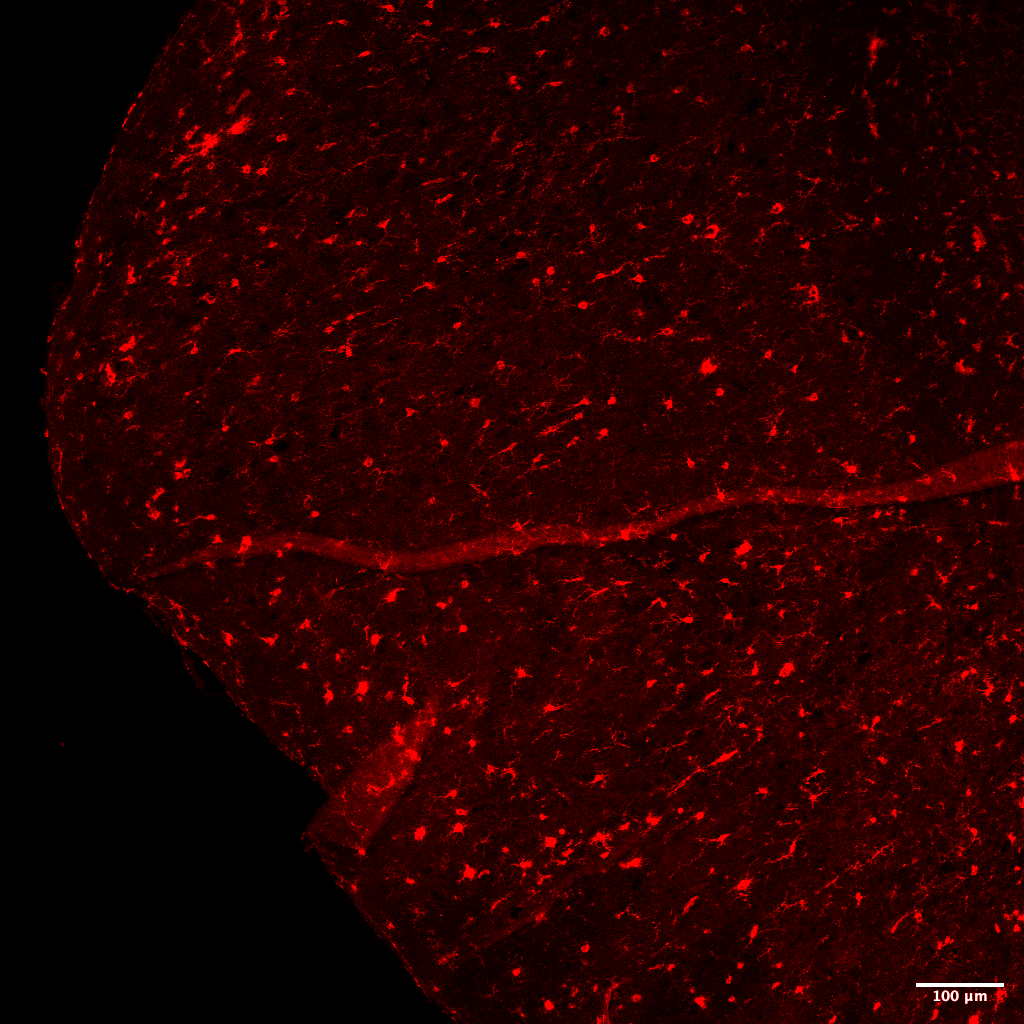

Supplement: Supplementary file 8 — Source data Fig. 7 [file 44321_2024_111_MOESM8_ESM.zip › EMM-2024-19843_SourceData-Figure7/7D/Iba1 IHC - KO+GFP.tiff]

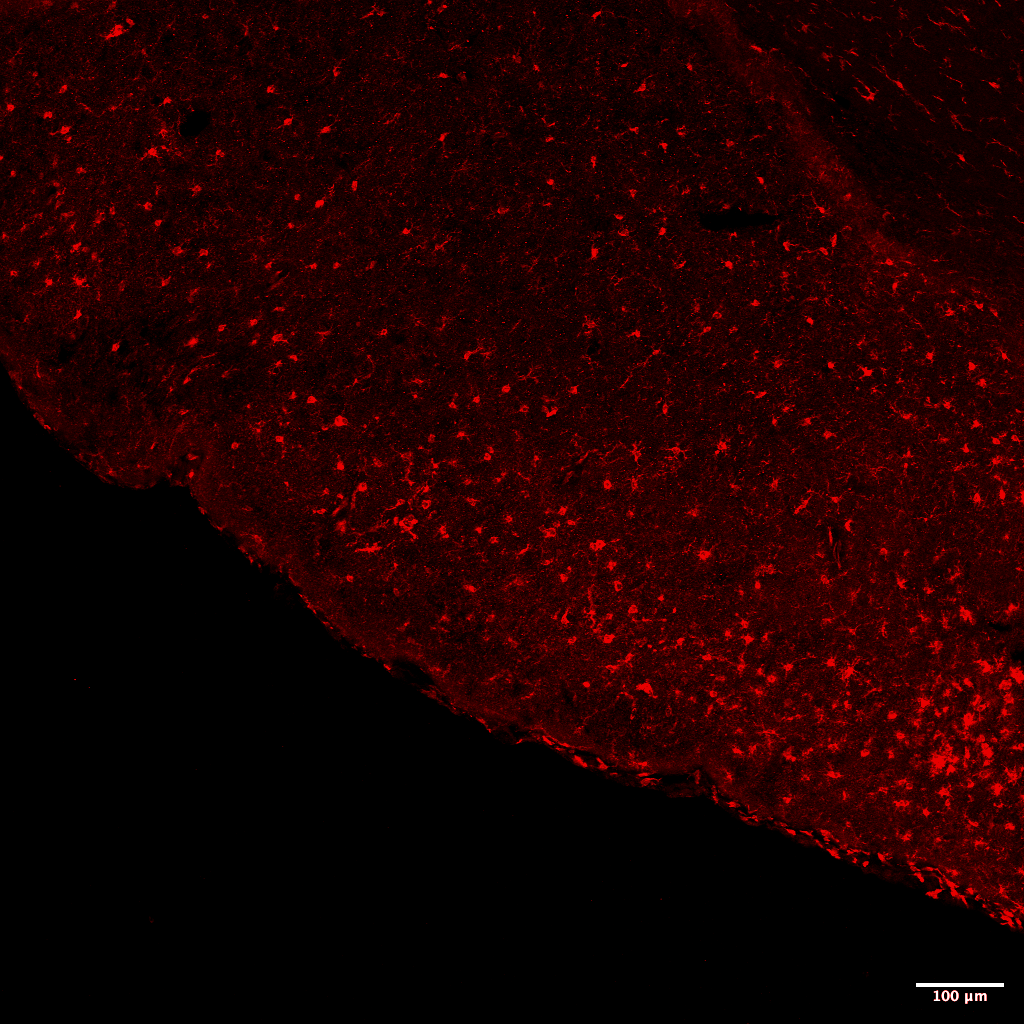

Supplement: Supplementary file 8 — Source data Fig. 7 [file 44321_2024_111_MOESM8_ESM.zip › EMM-2024-19843_SourceData-Figure7/7D/Iba1 IHC - KO+COX10.tiff]

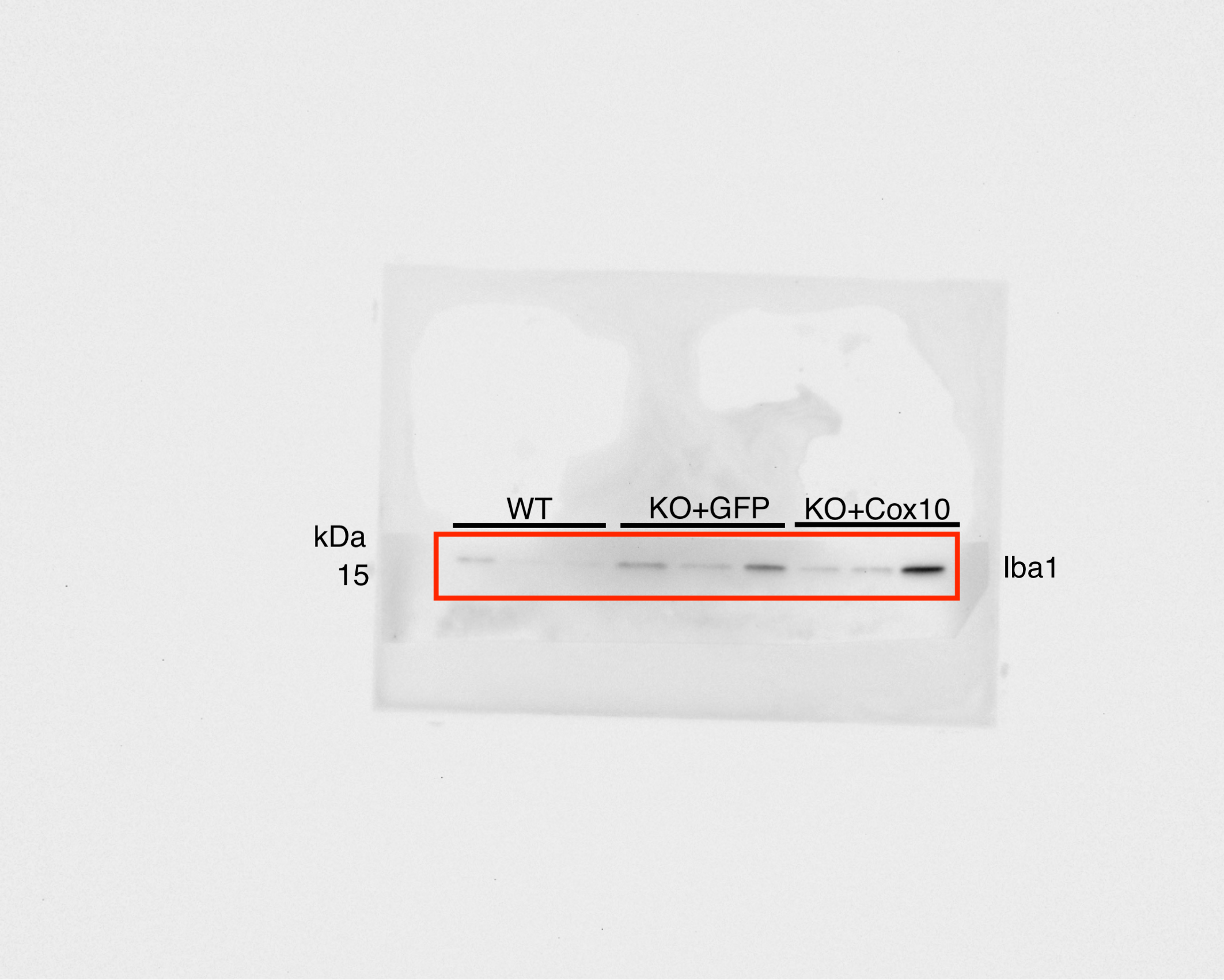

Supplement: Supplementary file 8 — Source data Fig. 7 [file 44321_2024_111_MOESM8_ESM.zip › EMM-2024-19843_SourceData-Figure7/7E/Hippocampus/western Iba1.tiff]

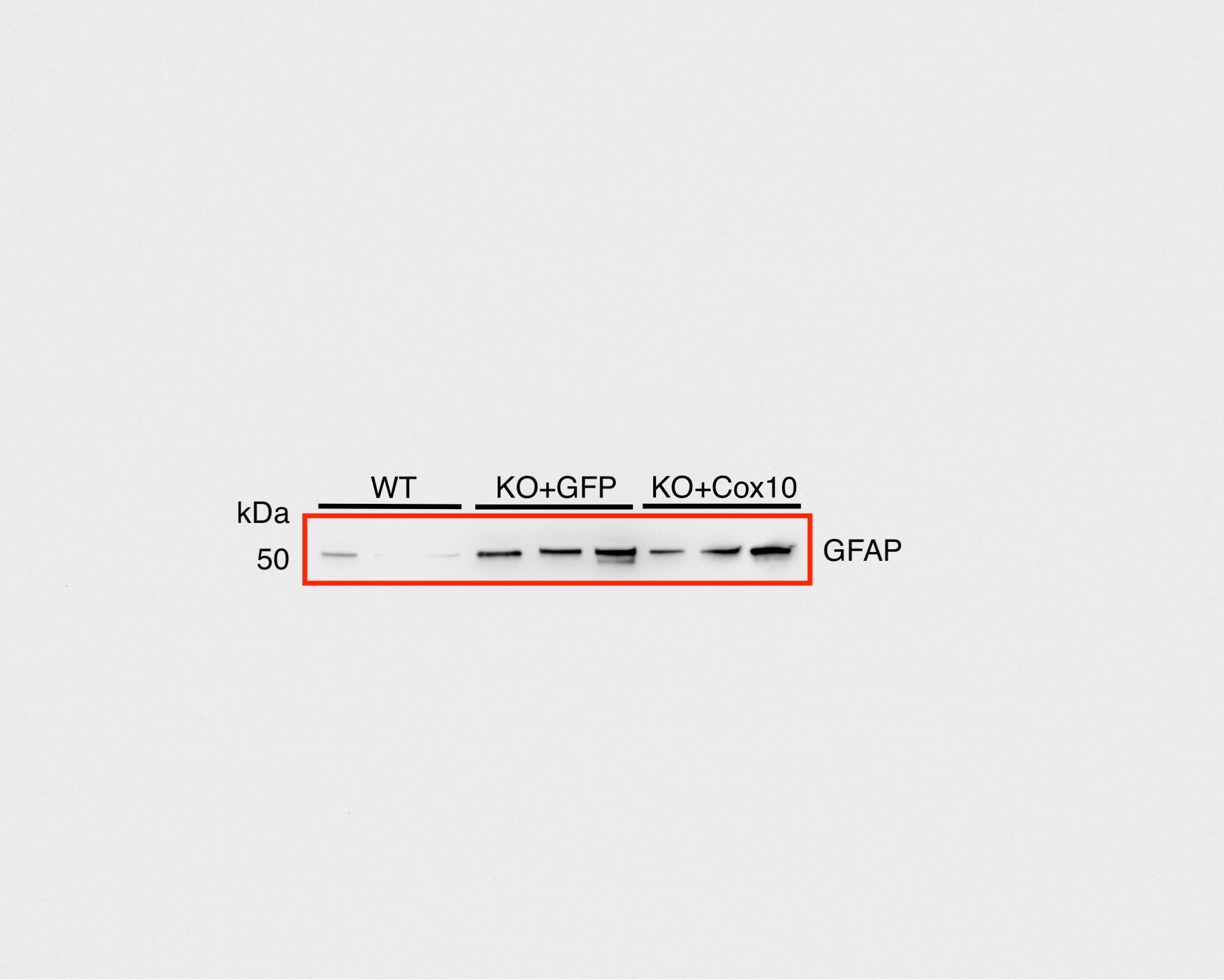

Supplement: Supplementary file 8 — Source data Fig. 7 [file 44321_2024_111_MOESM8_ESM.zip › EMM-2024-19843_SourceData-Figure7/7E/Hippocampus/western GFAP.tiff]

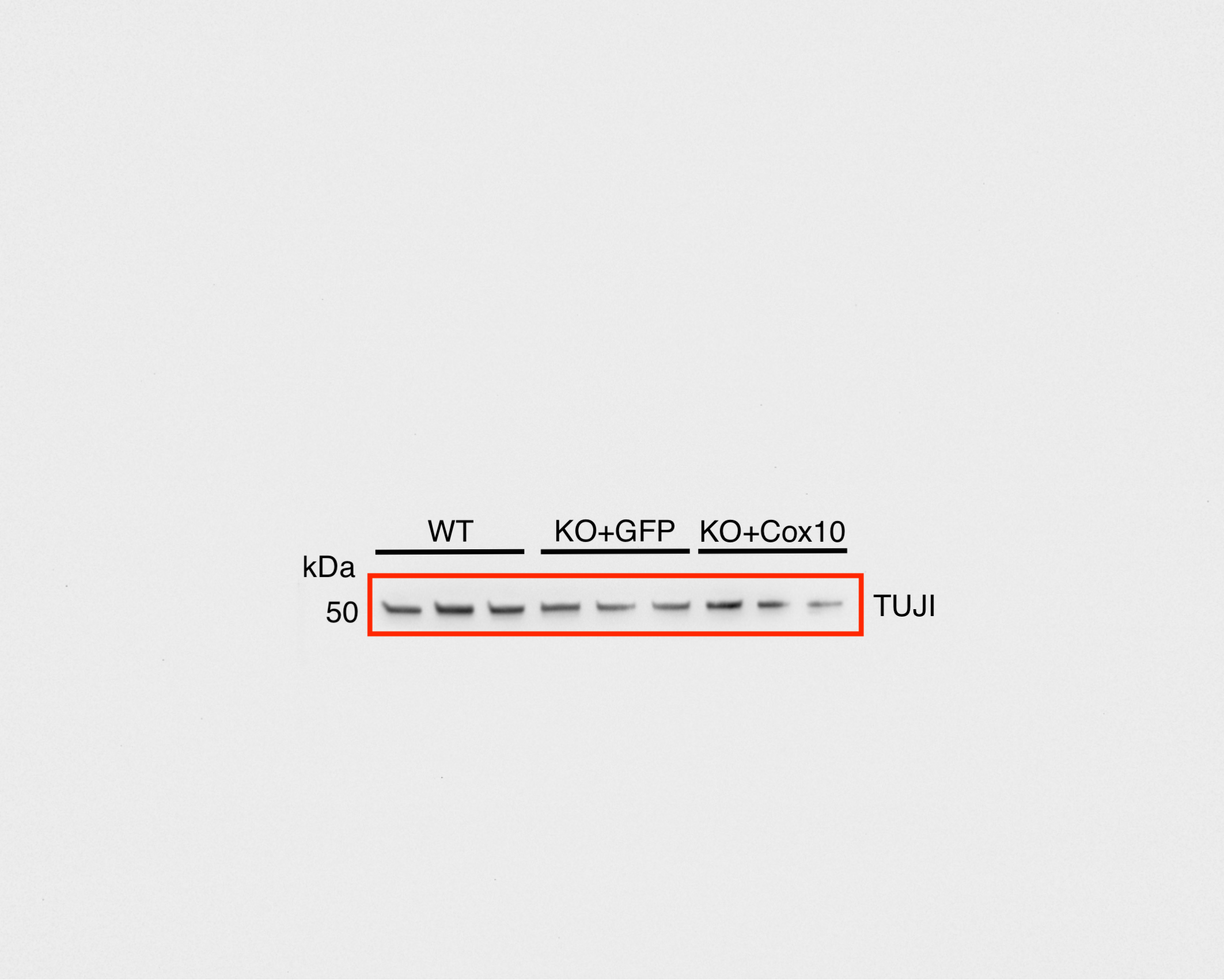

Supplement: Supplementary file 8 — Source data Fig. 7 [file 44321_2024_111_MOESM8_ESM.zip › EMM-2024-19843_SourceData-Figure7/7E/Hippocampus/western TUJI.tiff]

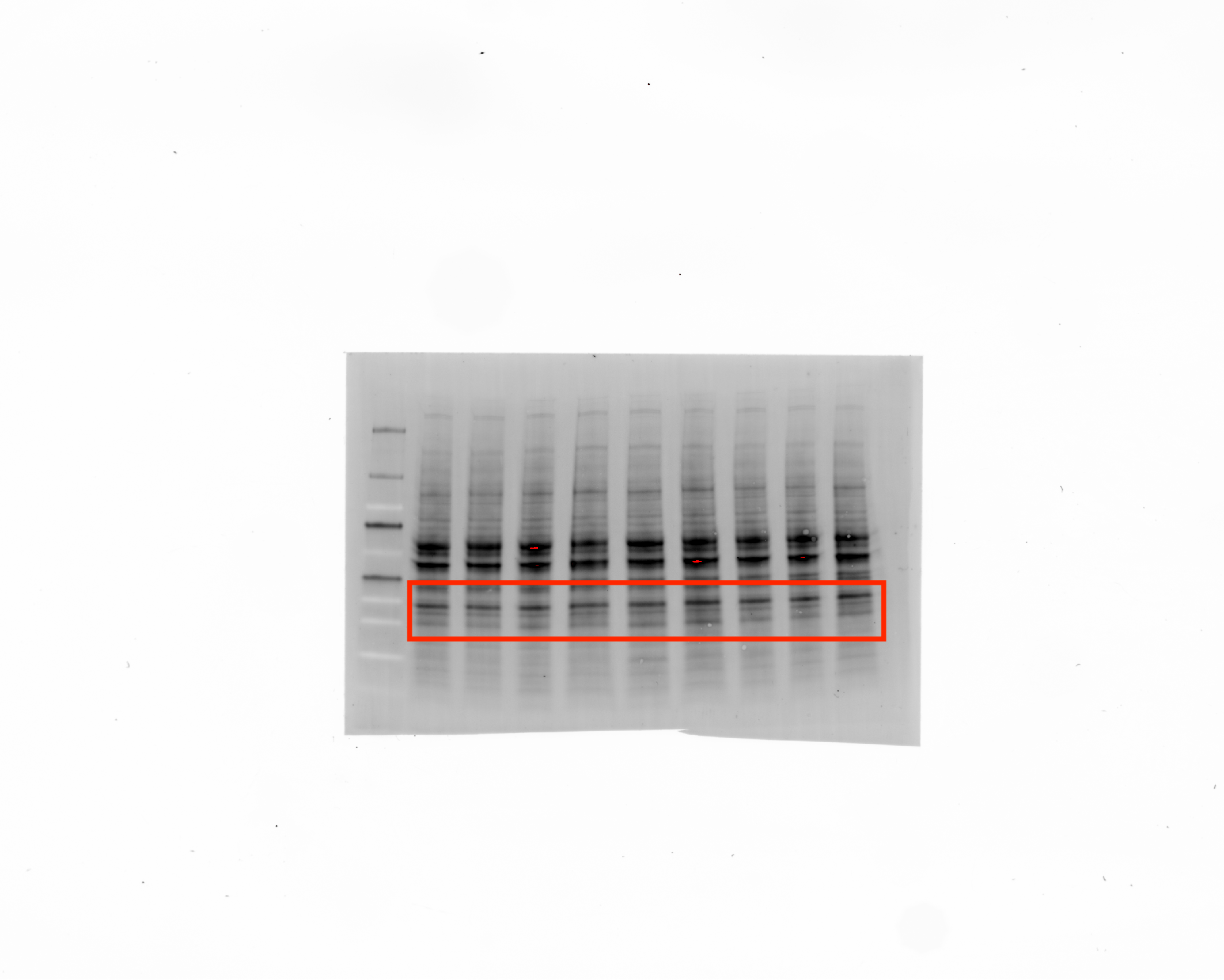

Supplement: Supplementary file 8 — Source data Fig. 7 [file 44321_2024_111_MOESM8_ESM.zip › EMM-2024-19843_SourceData-Figure7/7E/Hippocampus/western Total Protein.tiff]

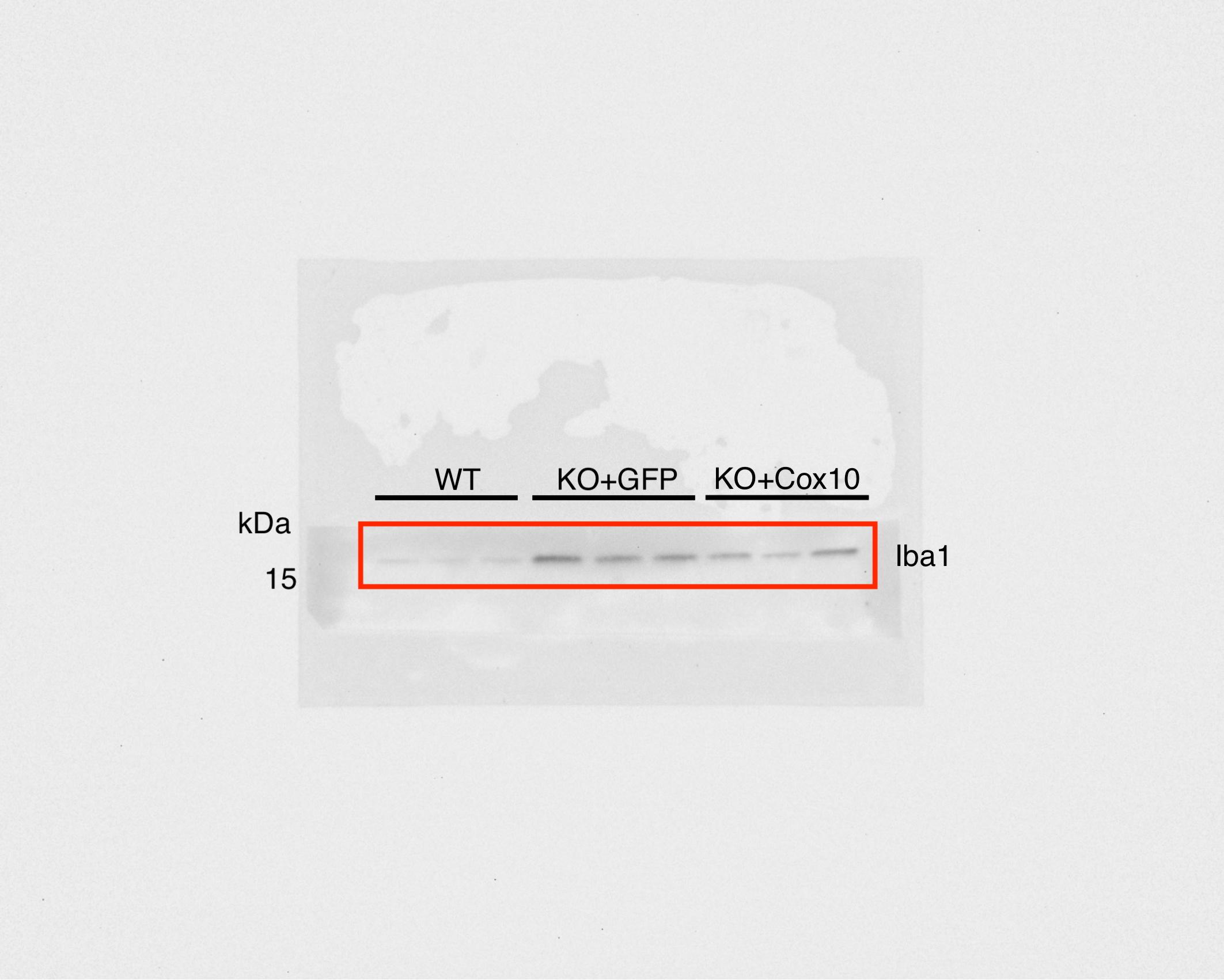

Supplement: Supplementary file 8 — Source data Fig. 7 [file 44321_2024_111_MOESM8_ESM.zip › EMM-2024-19843_SourceData-Figure7/7E/Cortex/western Iba1.tiff]

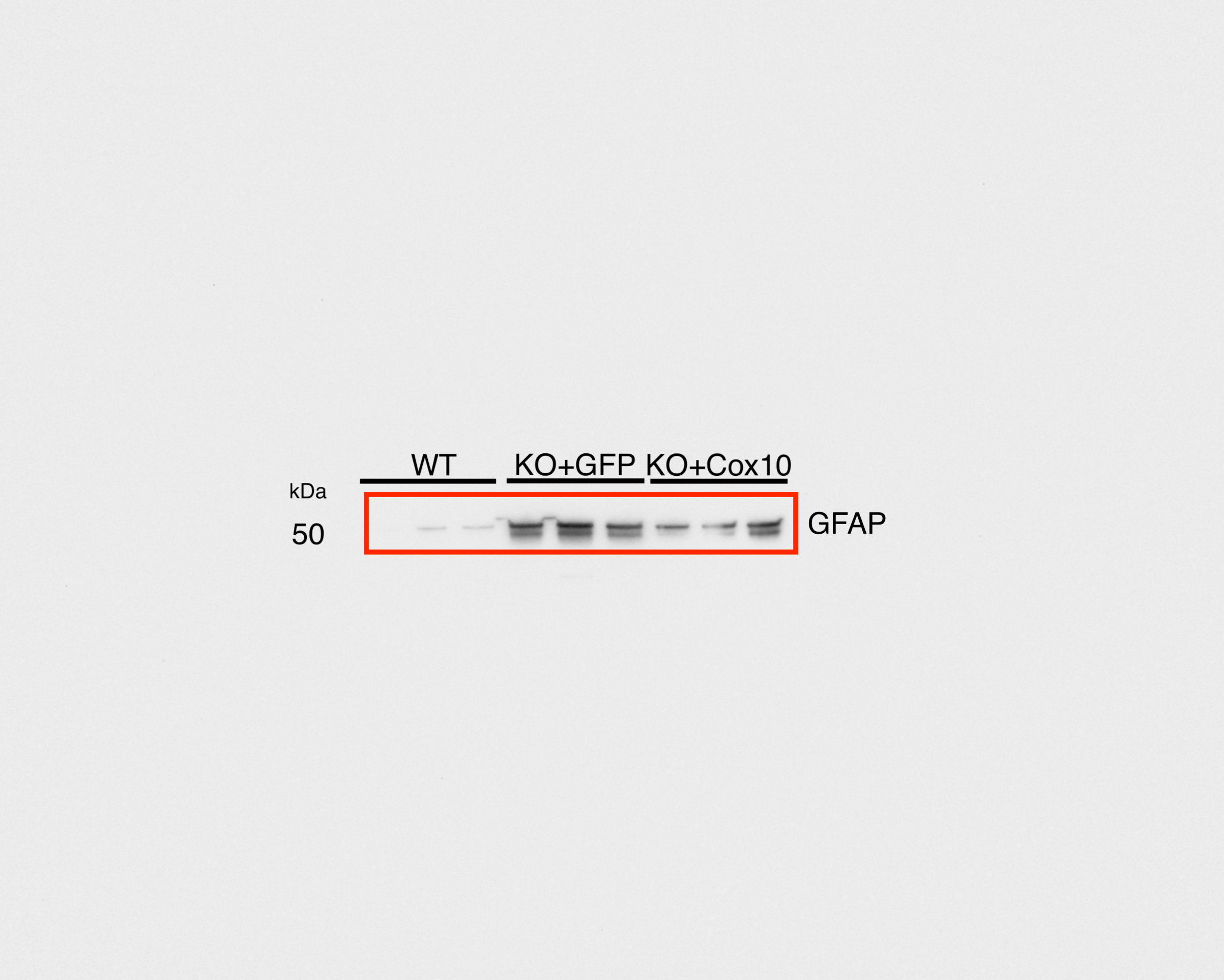

Supplement: Supplementary file 8 — Source data Fig. 7 [file 44321_2024_111_MOESM8_ESM.zip › EMM-2024-19843_SourceData-Figure7/7E/Cortex/western GFAP.tiff]

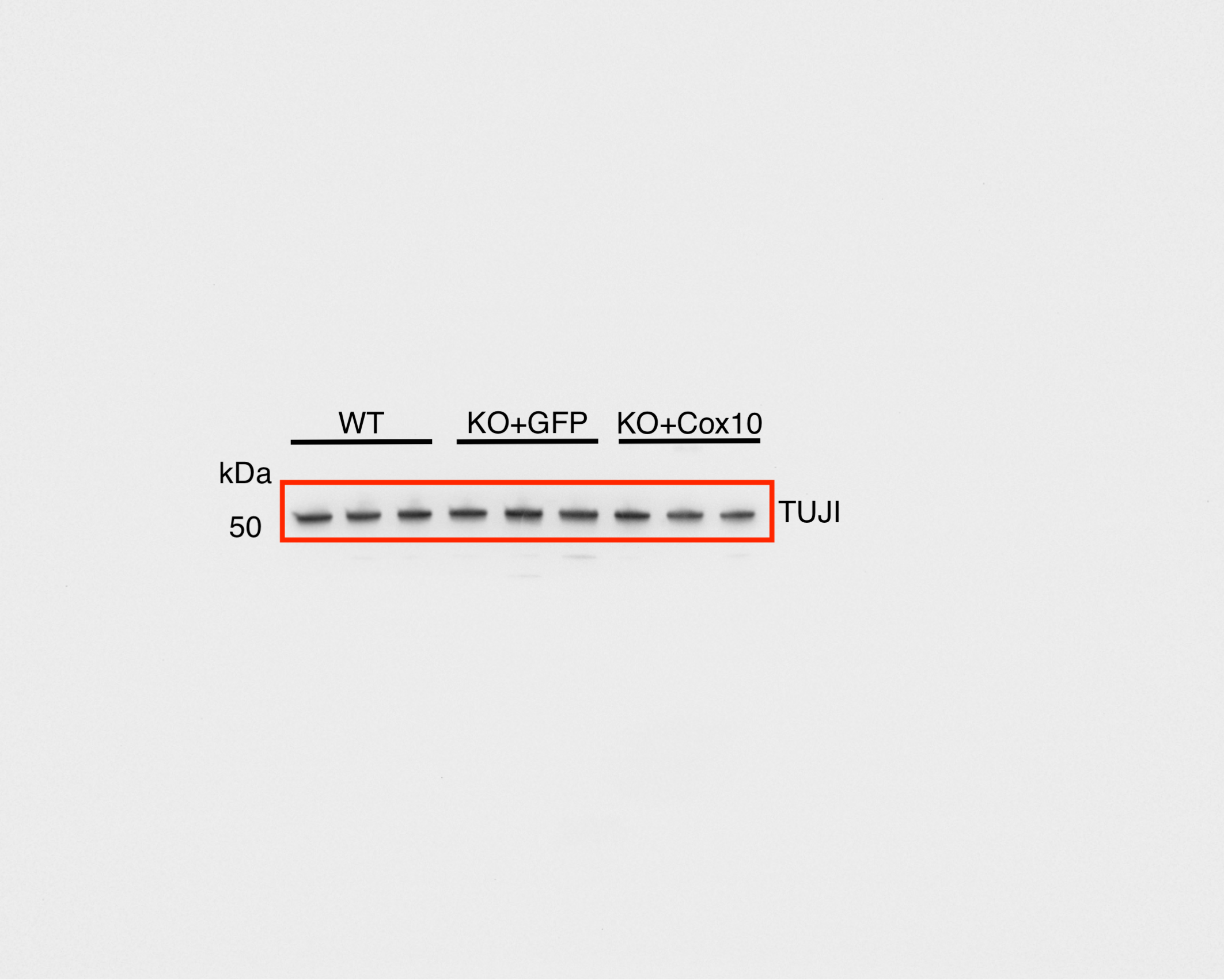

Supplement: Supplementary file 8 — Source data Fig. 7 [file 44321_2024_111_MOESM8_ESM.zip › EMM-2024-19843_SourceData-Figure7/7E/Cortex/western TUJI.tiff]

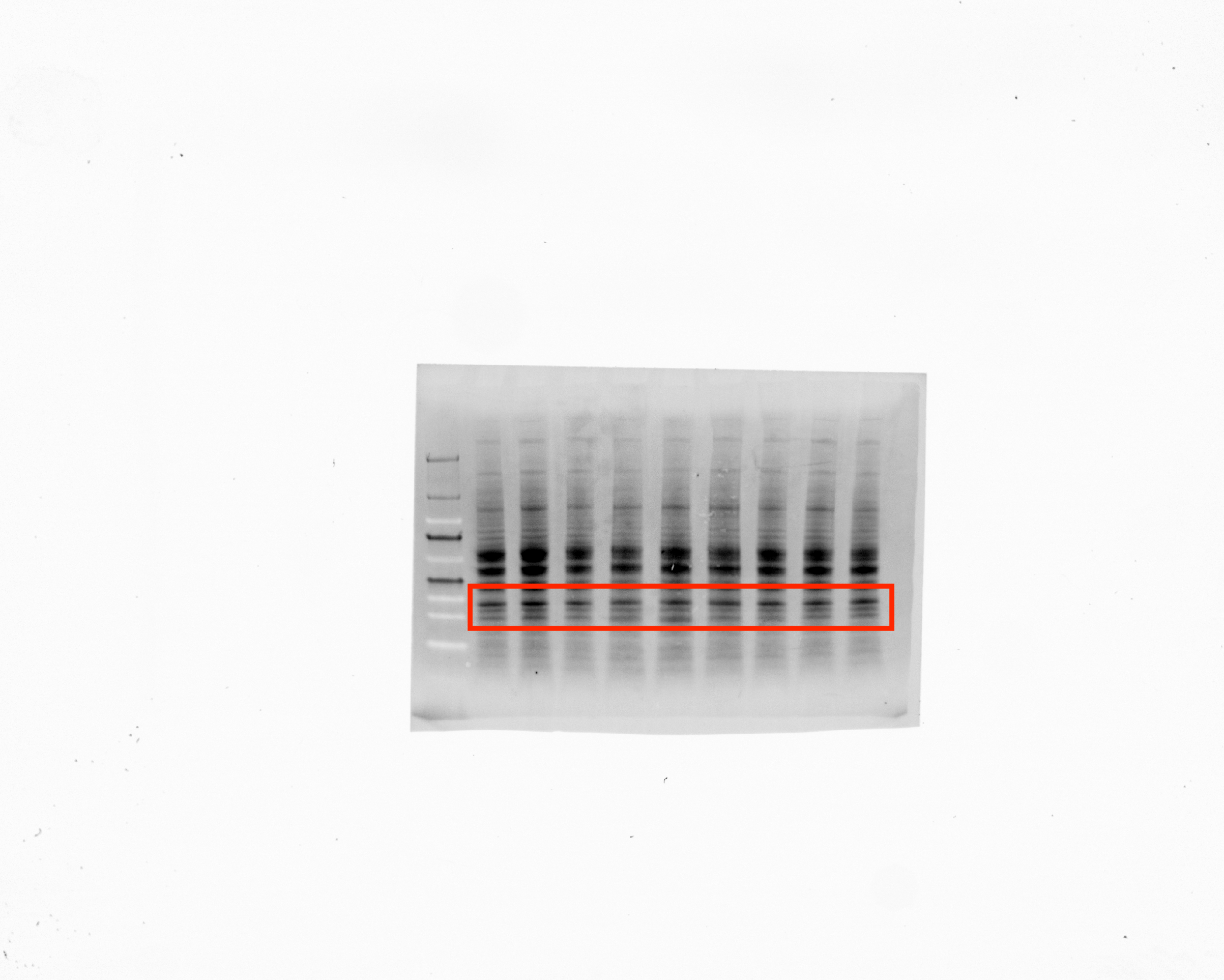

Supplement: Supplementary file 8 — Source data Fig. 7 [file 44321_2024_111_MOESM8_ESM.zip › EMM-2024-19843_SourceData-Figure7/7E/Cortex/western Total Protein.tiff]

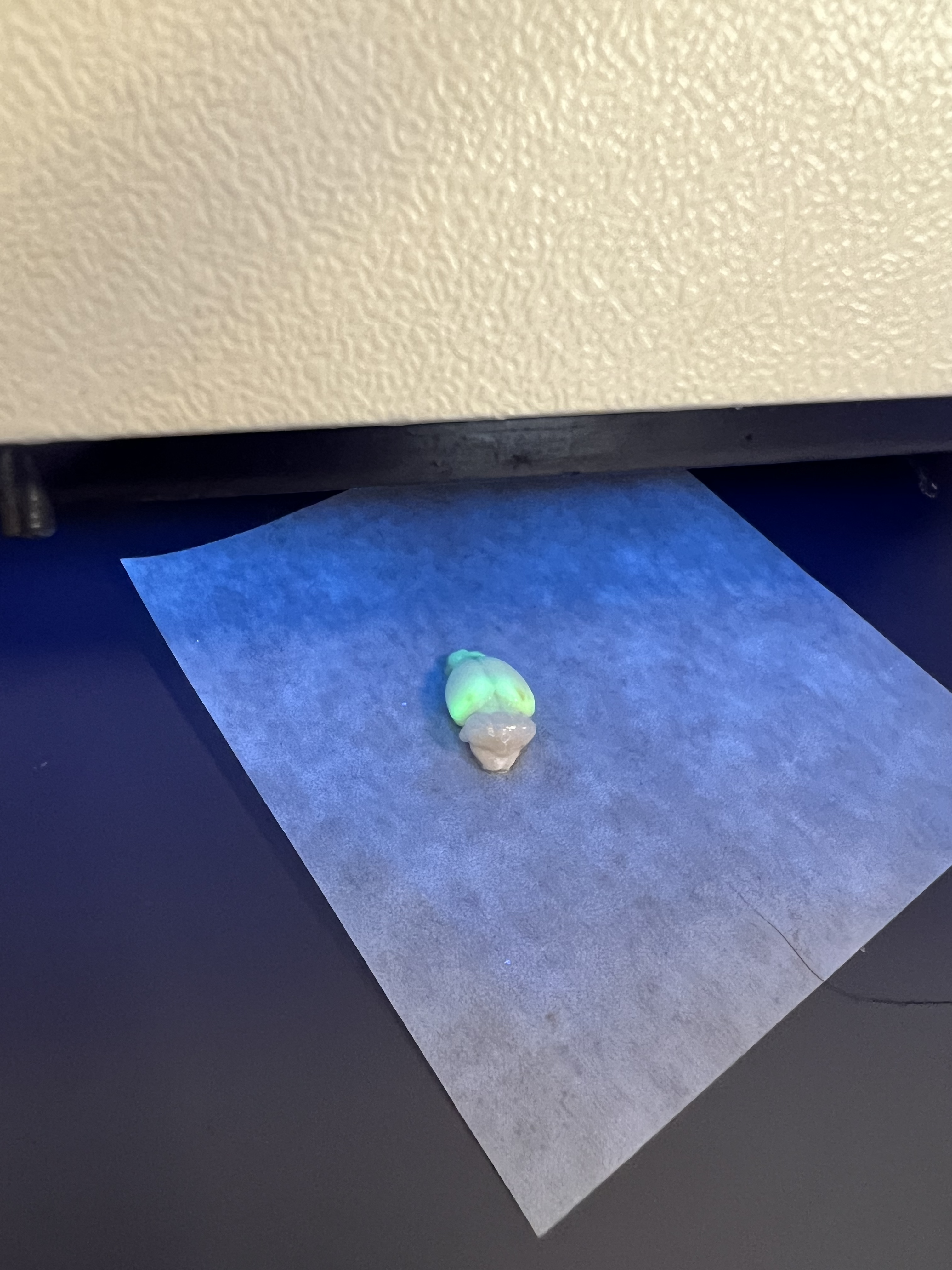

Supplement: Supplementary file 9 — Source data Fig. 8 [file 44321_2024_111_MOESM9_ESM.zip › EMM-2024-19843_SourceData-Figure8/8B.tiff]

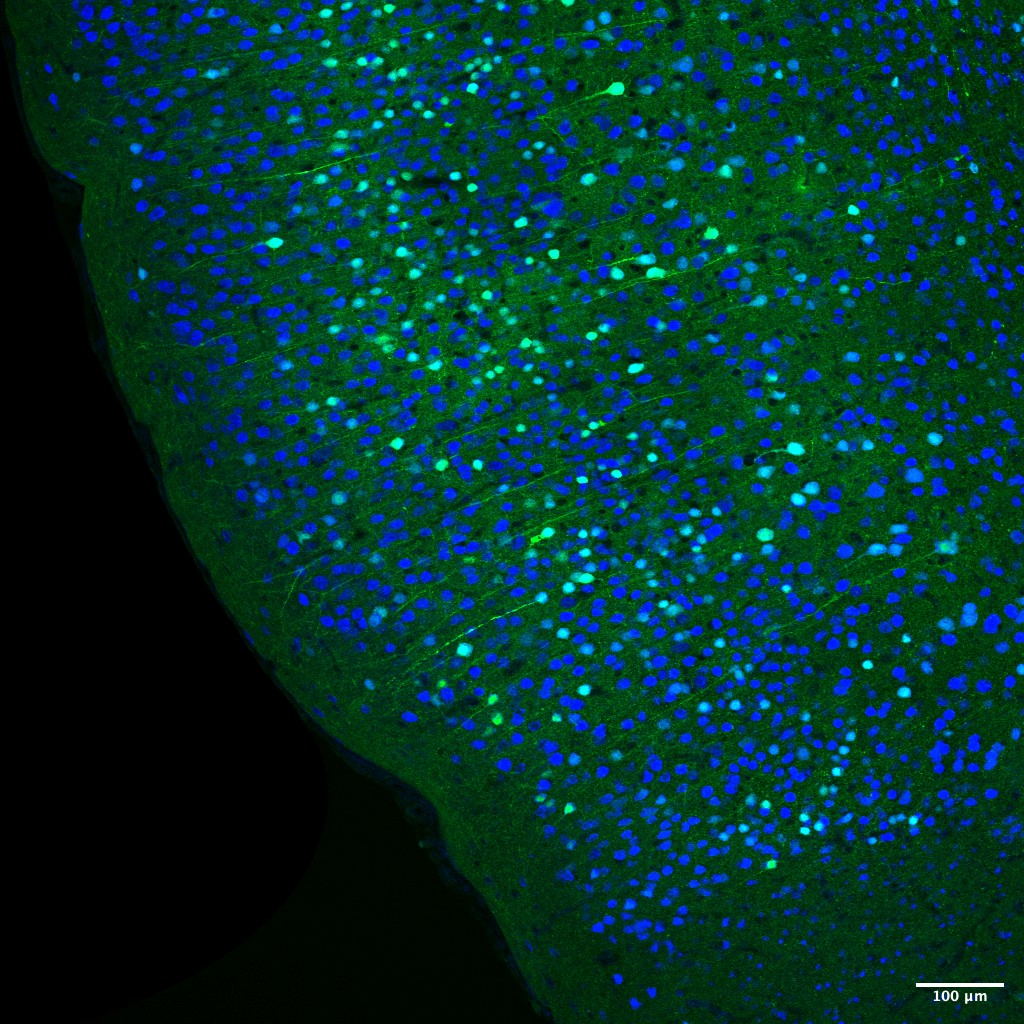

Supplement: Supplementary file 9 — Source data Fig. 8 [file 44321_2024_111_MOESM9_ESM.zip › EMM-2024-19843_SourceData-Figure8/8D-E/GFP NeuN IHC - Motor Cortex.tiff]

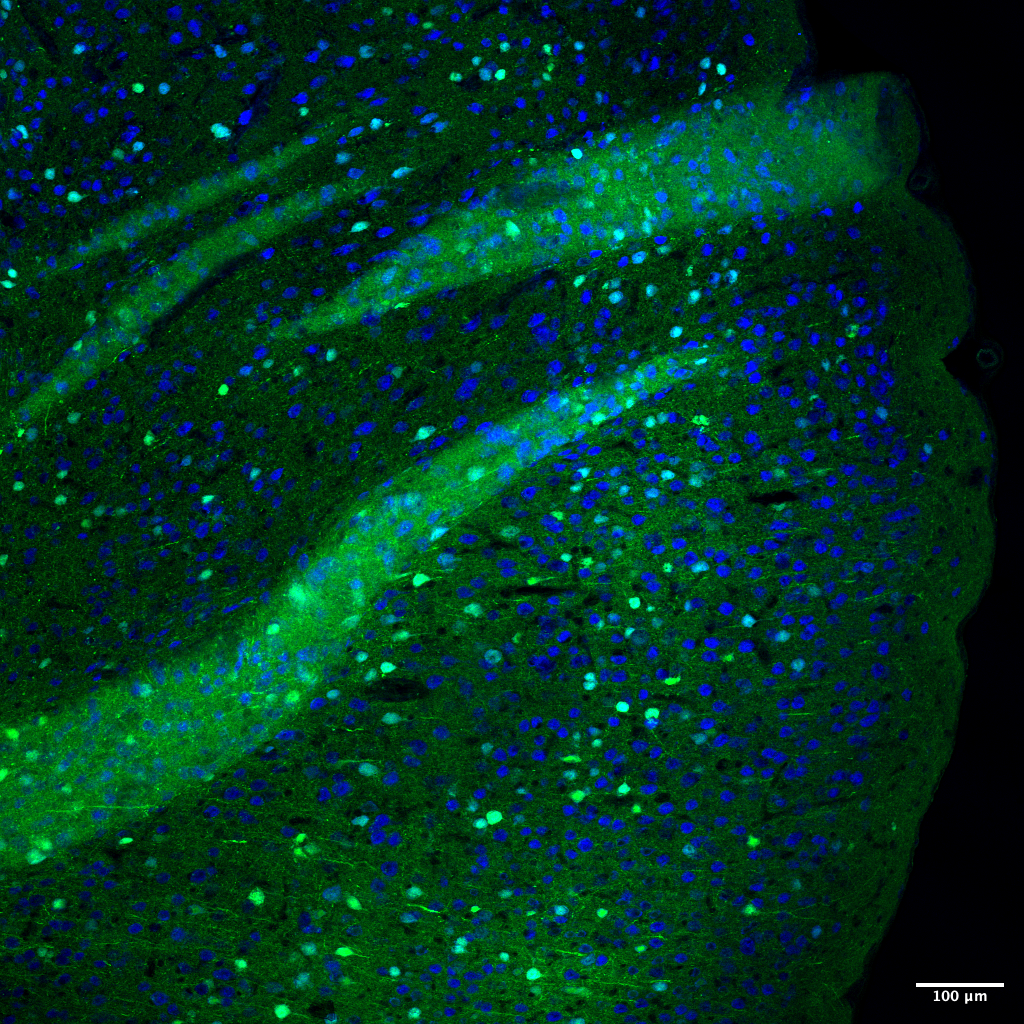

Supplement: Supplementary file 9 — Source data Fig. 8 [file 44321_2024_111_MOESM9_ESM.zip › EMM-2024-19843_SourceData-Figure8/8D-E/GFP NeuN IHC - Piriform Cortex.tiff]

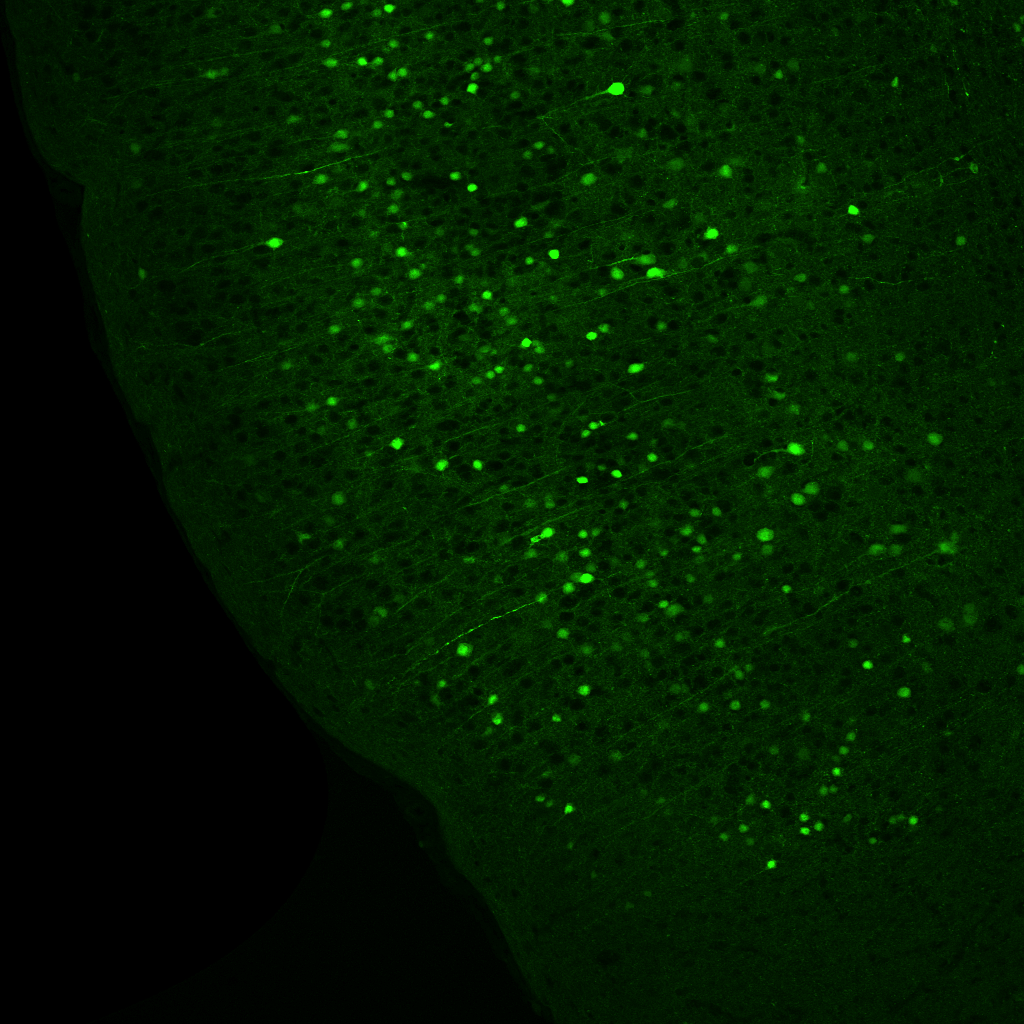

Supplement: Supplementary file 9 — Source data Fig. 8 [file 44321_2024_111_MOESM9_ESM.zip › EMM-2024-19843_SourceData-Figure8/8D-E/GFP IHC - Motor Cortex.tiff]

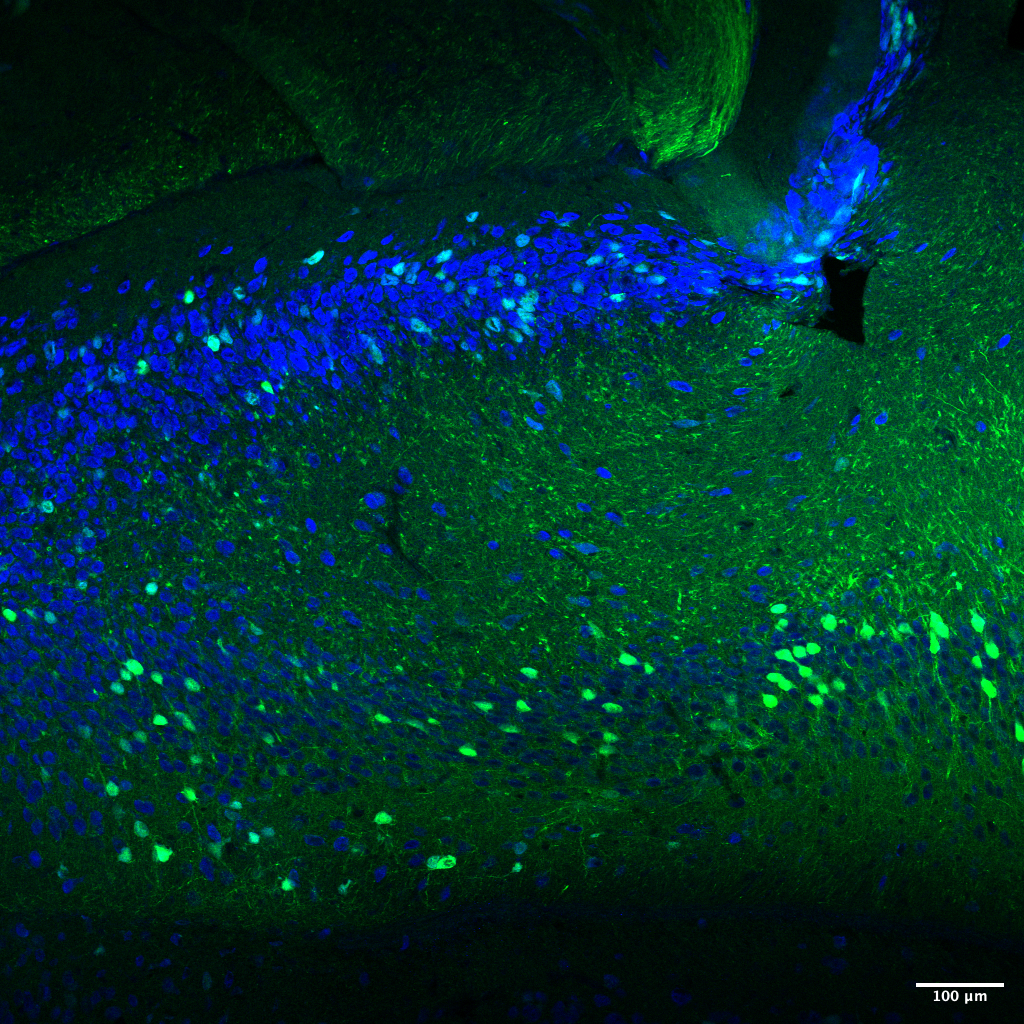

Supplement: Supplementary file 9 — Source data Fig. 8 [file 44321_2024_111_MOESM9_ESM.zip › EMM-2024-19843_SourceData-Figure8/8D-E/GFP NeuN IHC - Hippocampus.tiff]

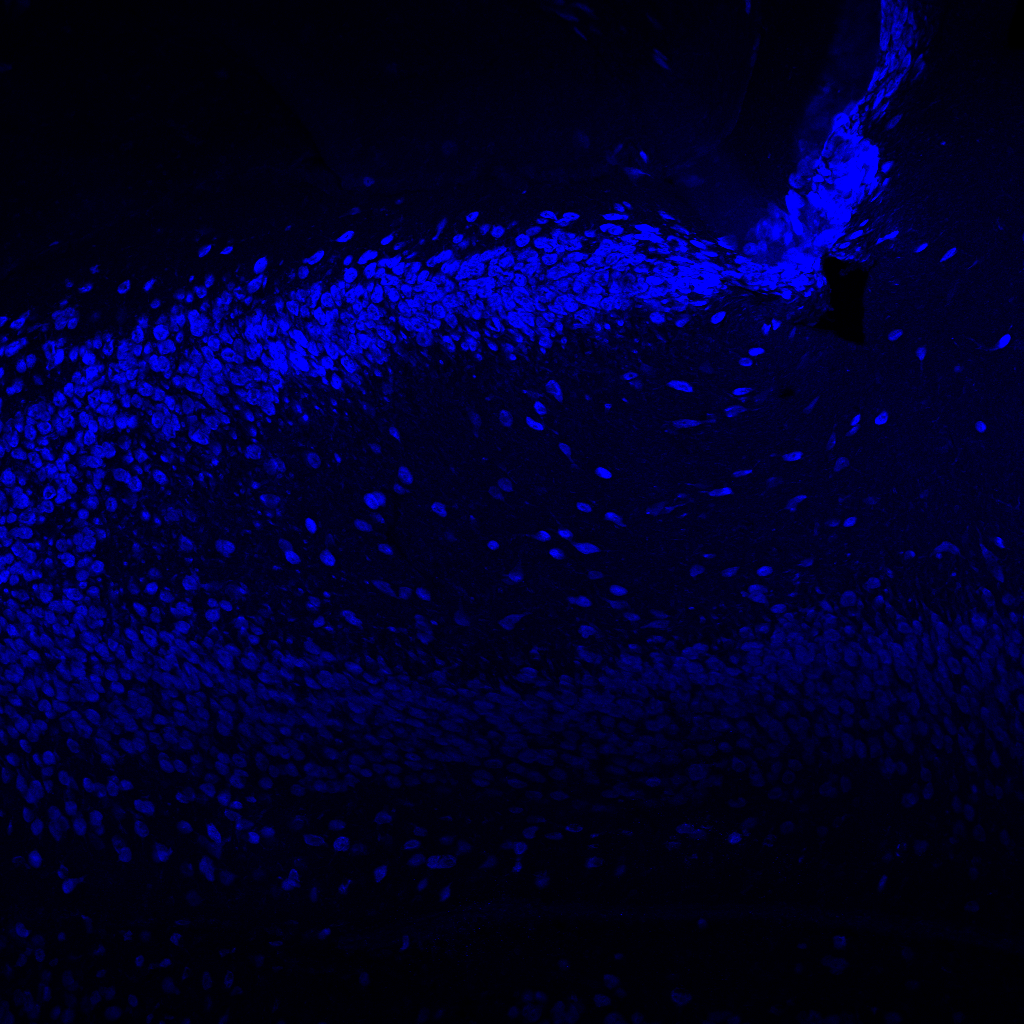

Supplement: Supplementary file 9 — Source data Fig. 8 [file 44321_2024_111_MOESM9_ESM.zip › EMM-2024-19843_SourceData-Figure8/8D-E/NeuN IHC - Hippocampus.tiff]

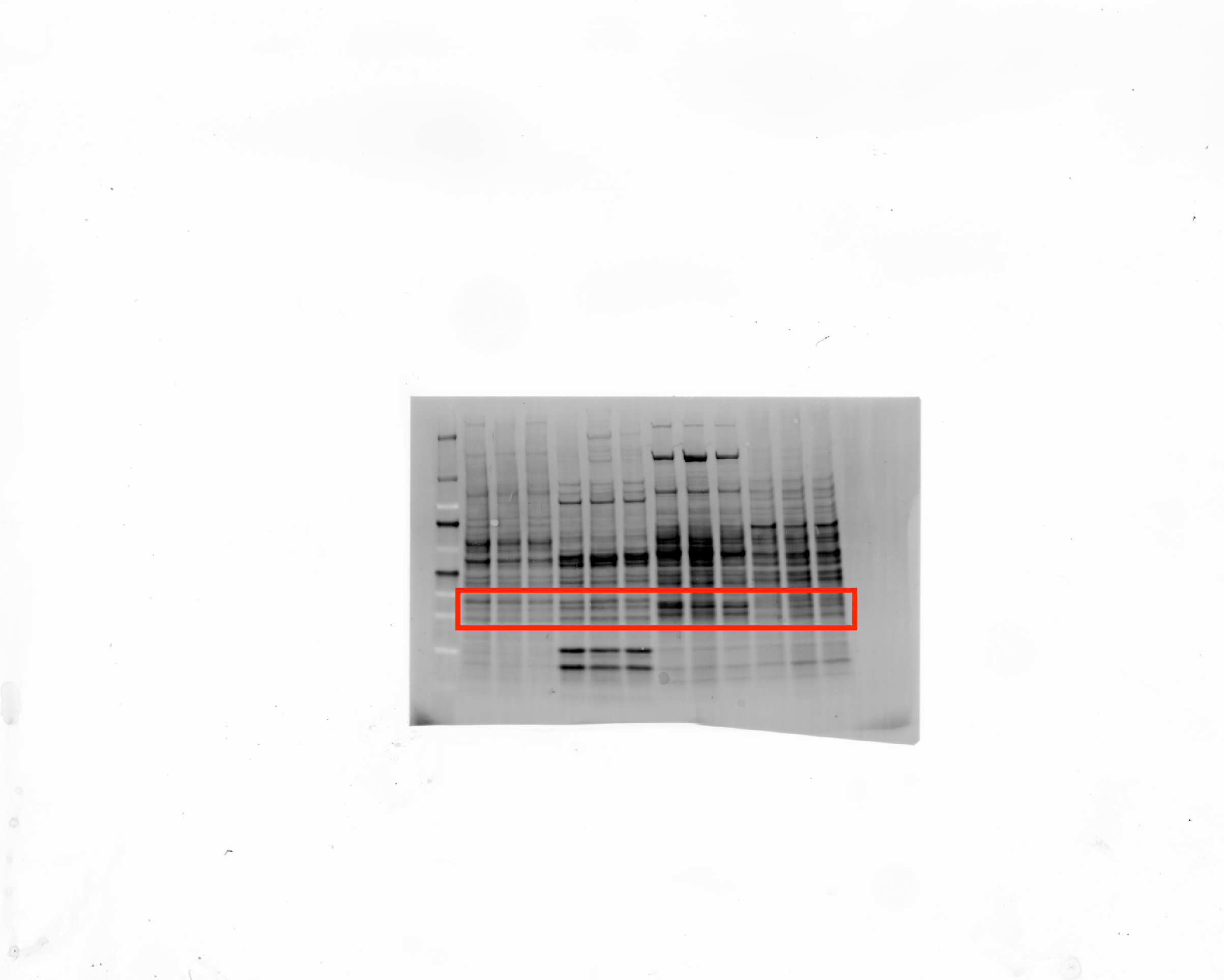

Supplement: Supplementary file 9 — Source data Fig. 8 [file 44321_2024_111_MOESM9_ESM.zip › EMM-2024-19843_SourceData-Figure8/8A/western Total Protein.tiff]

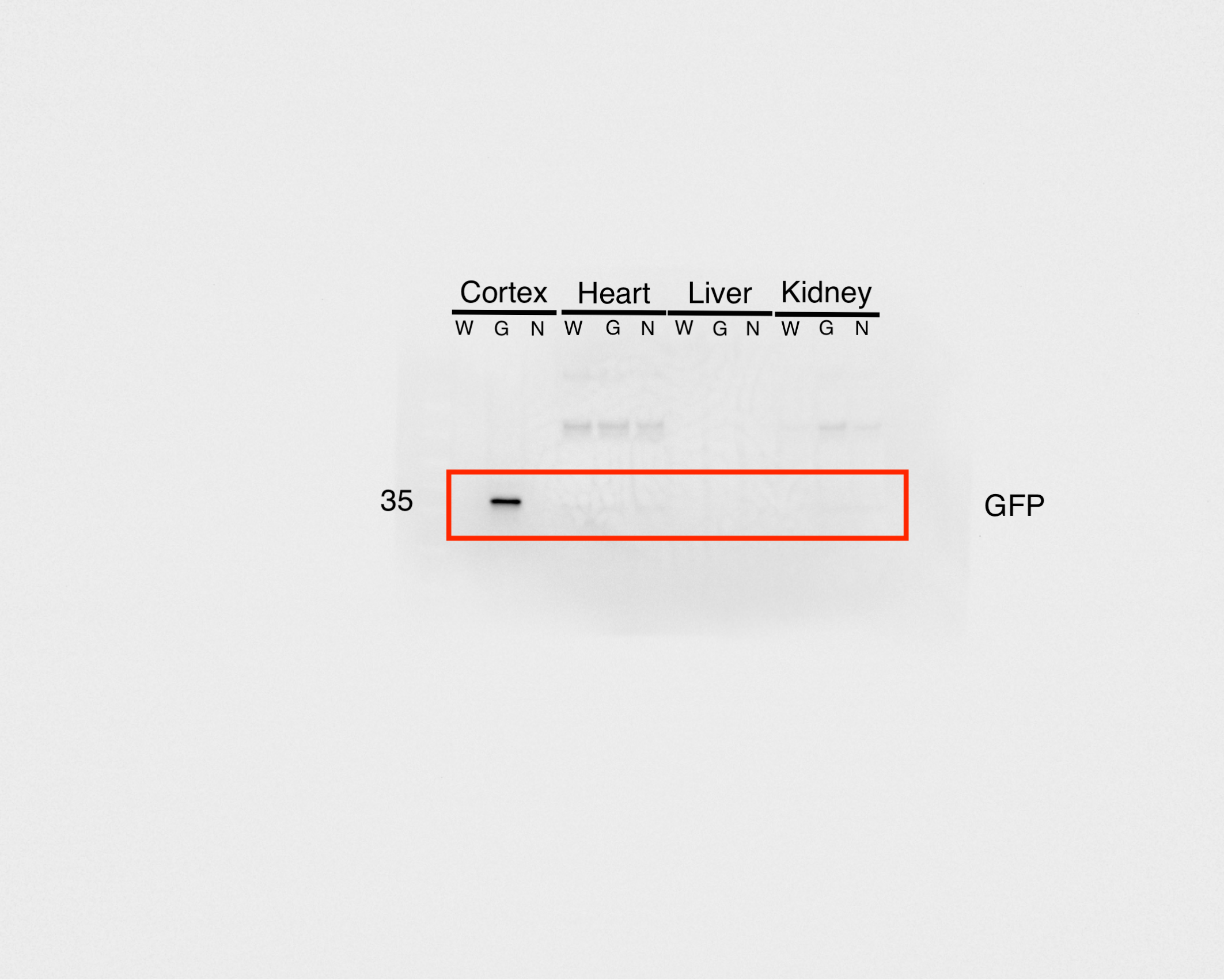

Supplement: Supplementary file 9 — Source data Fig. 8 [file 44321_2024_111_MOESM9_ESM.zip › EMM-2024-19843_SourceData-Figure8/8A/western GFP.tiff]

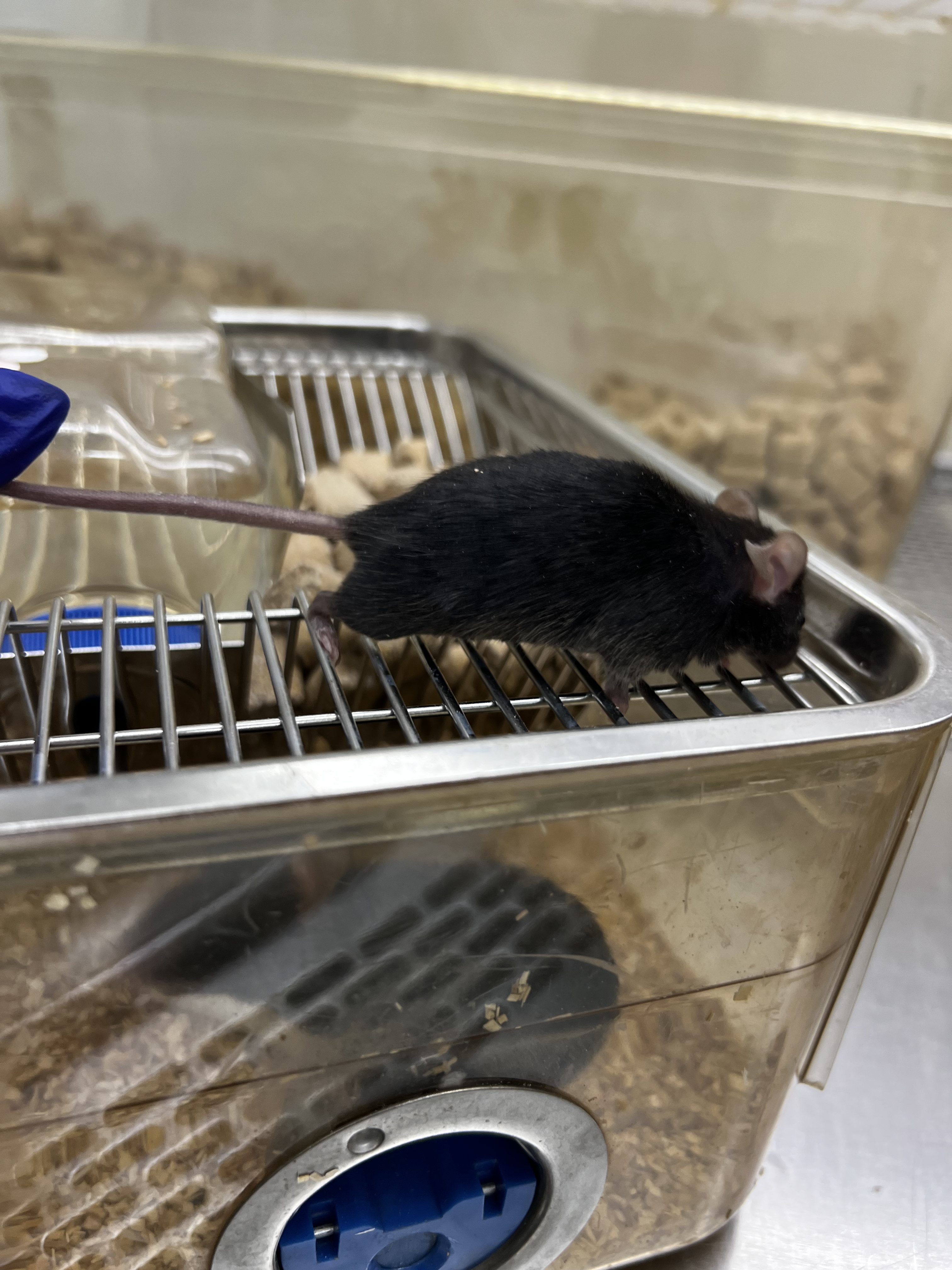

Supplement: Supplementary file 10 — EV and Appendix Figure Source Data [file 44321_2024_111_MOESM10_ESM.zip › Source Data for Expanded View and Appendix/EMM-2024-19843_SourceData-FigureEV5/EV5D.tiff]

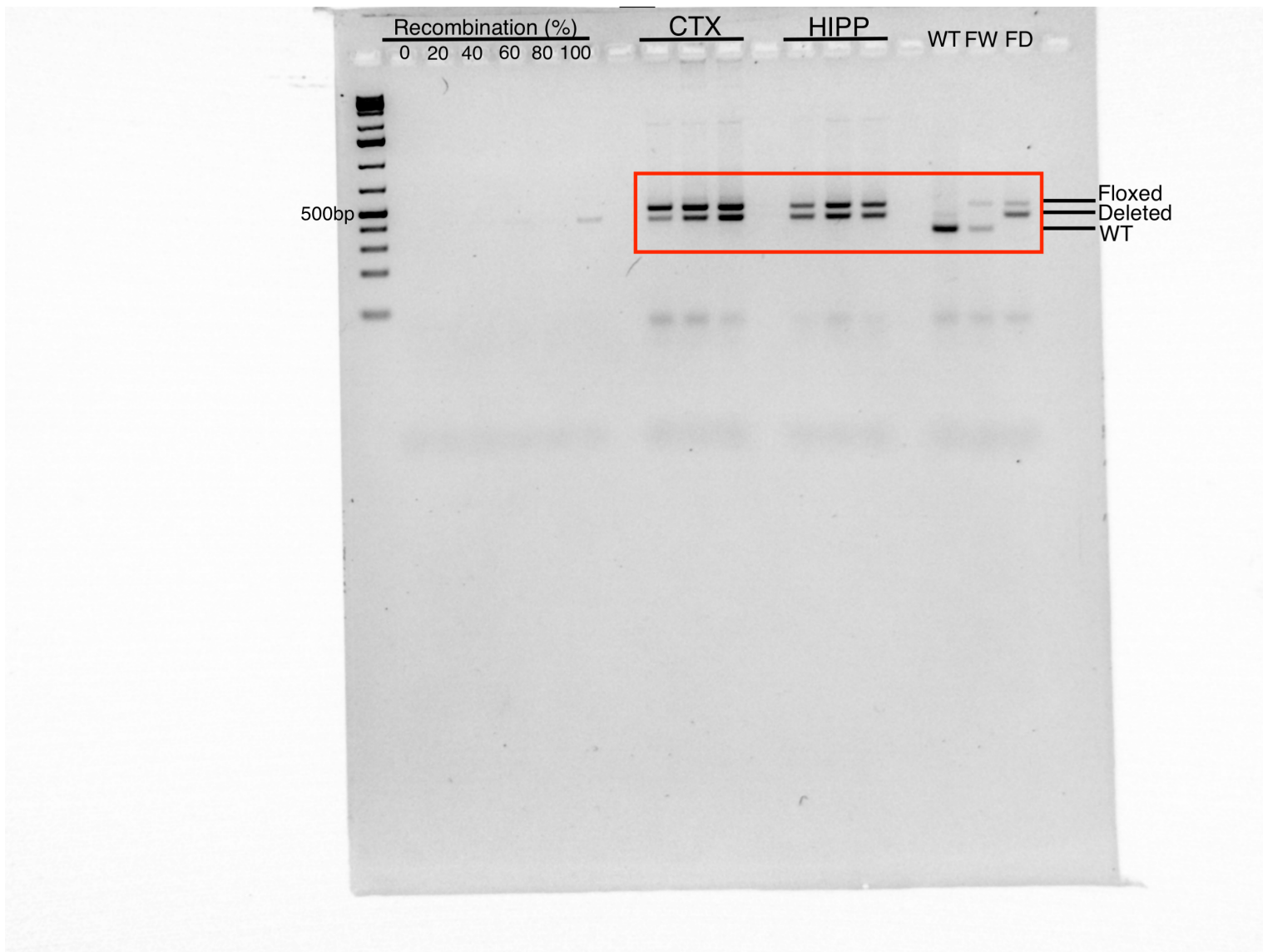

Supplement: Supplementary file 10 — EV and Appendix Figure Source Data [file 44321_2024_111_MOESM10_ESM.zip › Source Data for Expanded View and Appendix/EMM-2024-19843_SourceData-FigureEV5/EV5E-annotated.pdf]

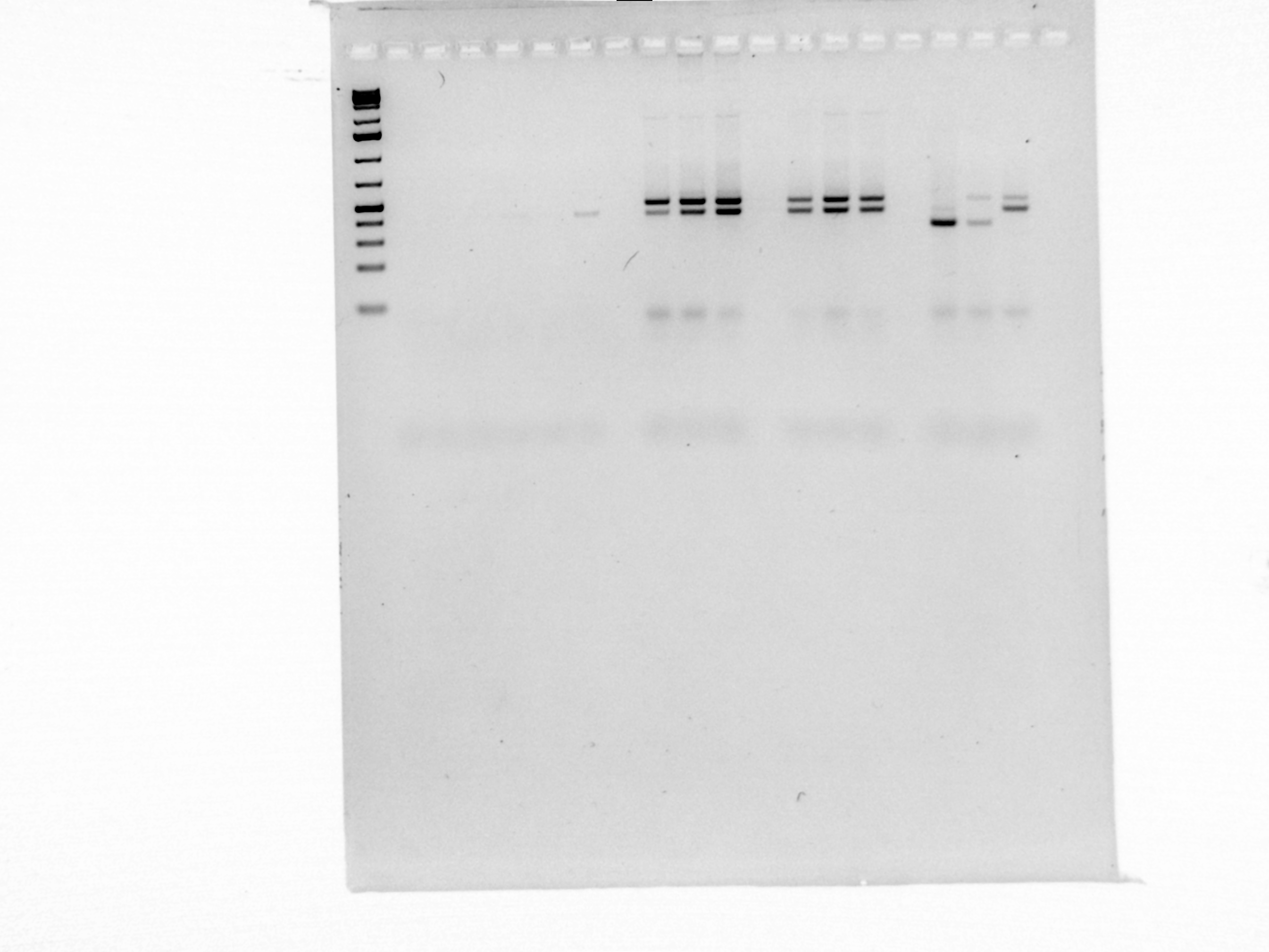

Supplement: Supplementary file 10 — EV and Appendix Figure Source Data [file 44321_2024_111_MOESM10_ESM.zip › Source Data for Expanded View and Appendix/EMM-2024-19843_SourceData-FigureEV5/EV5E.tiff]

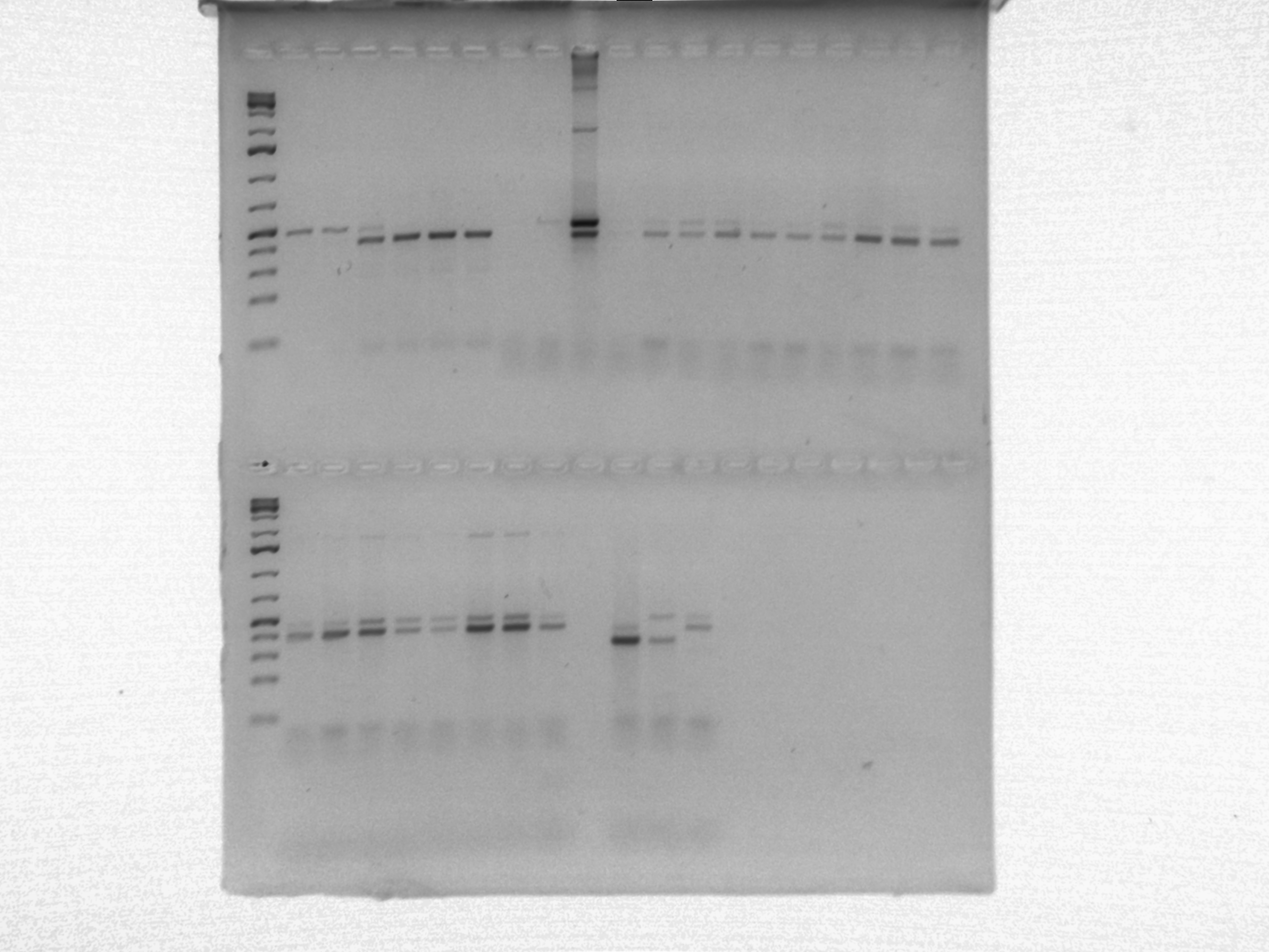

Supplement: Supplementary file 10 — EV and Appendix Figure Source Data [file 44321_2024_111_MOESM10_ESM.zip › Source Data for Expanded View and Appendix/Appendix/EMM-2024-19843_SourceData-FigureS3/S3A.tiff]

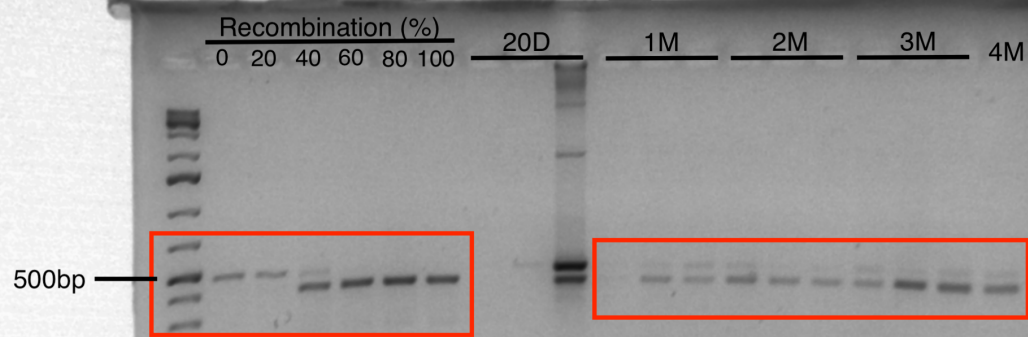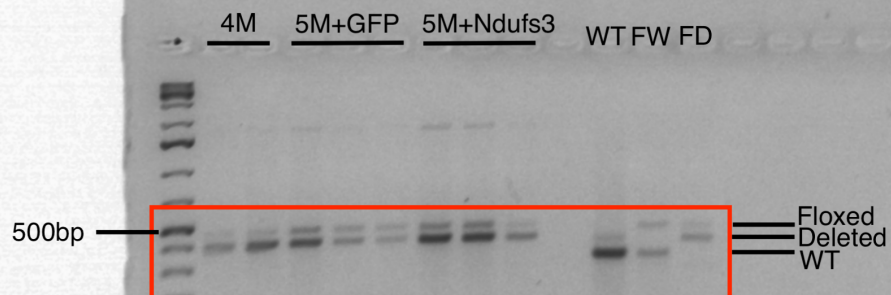

Supplement: Supplementary file 10 — EV and Appendix Figure Source Data [file 44321_2024_111_MOESM10_ESM.zip › Source Data for Expanded View and Appendix/Appendix/EMM-2024-19843_SourceData-FigureS3/S3A-annotated.pdf]

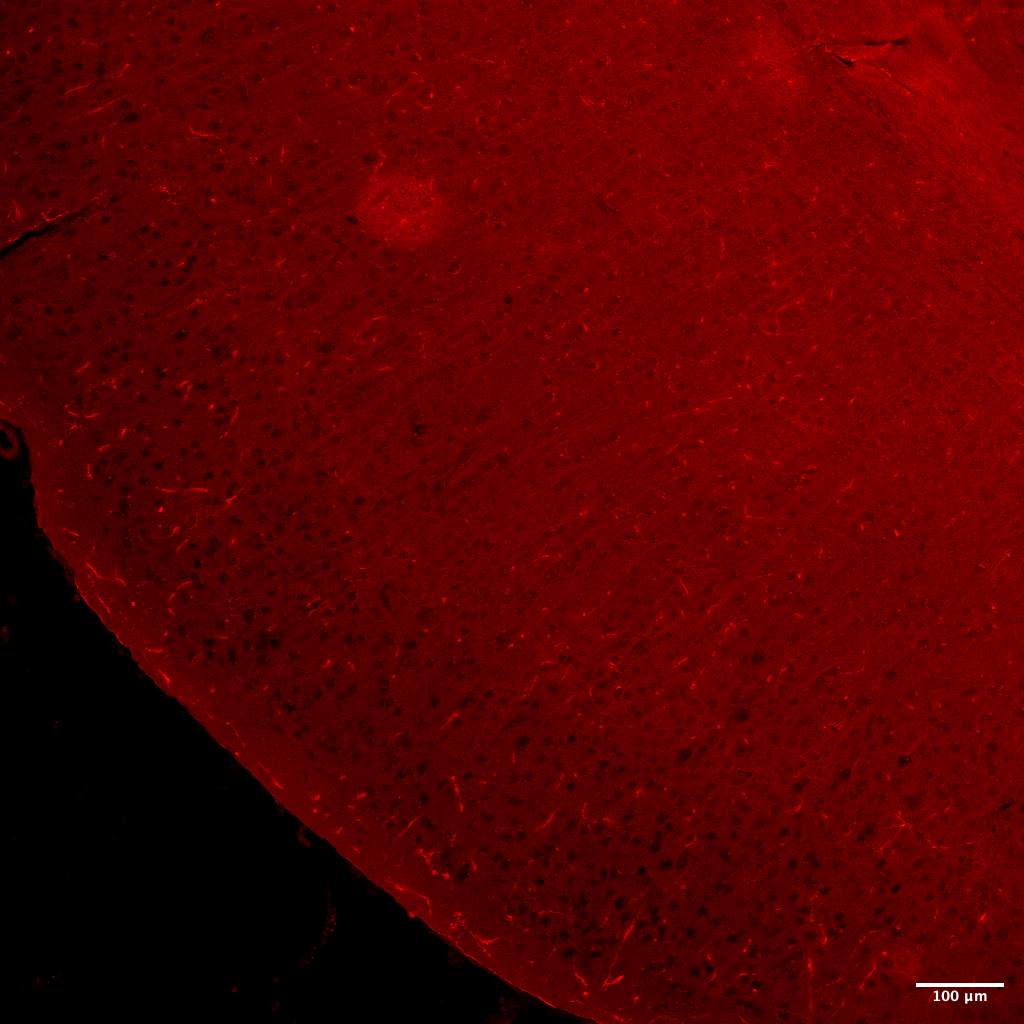

Supplement: Supplementary file 10 — EV and Appendix Figure Source Data [file 44321_2024_111_MOESM10_ESM.zip › Source Data for Expanded View and Appendix/EMM-2024-19843_SourceData-FigureEV4/EV4A/GFAP IHC - KO+COX10.tiff]
