# Supplementary figures and images for: Restoration of defective oxidative phosphorylation to a subset of neurons prevents mitochondrial encephalopathy (part 2 of 2)
Source: EMBO Mol Med. 2024 Aug 21;16(9):13. doi: 10.1038/s44321-024-00111-4 (PMC11392956; doi:10.1038/s44321-024-00111-4)

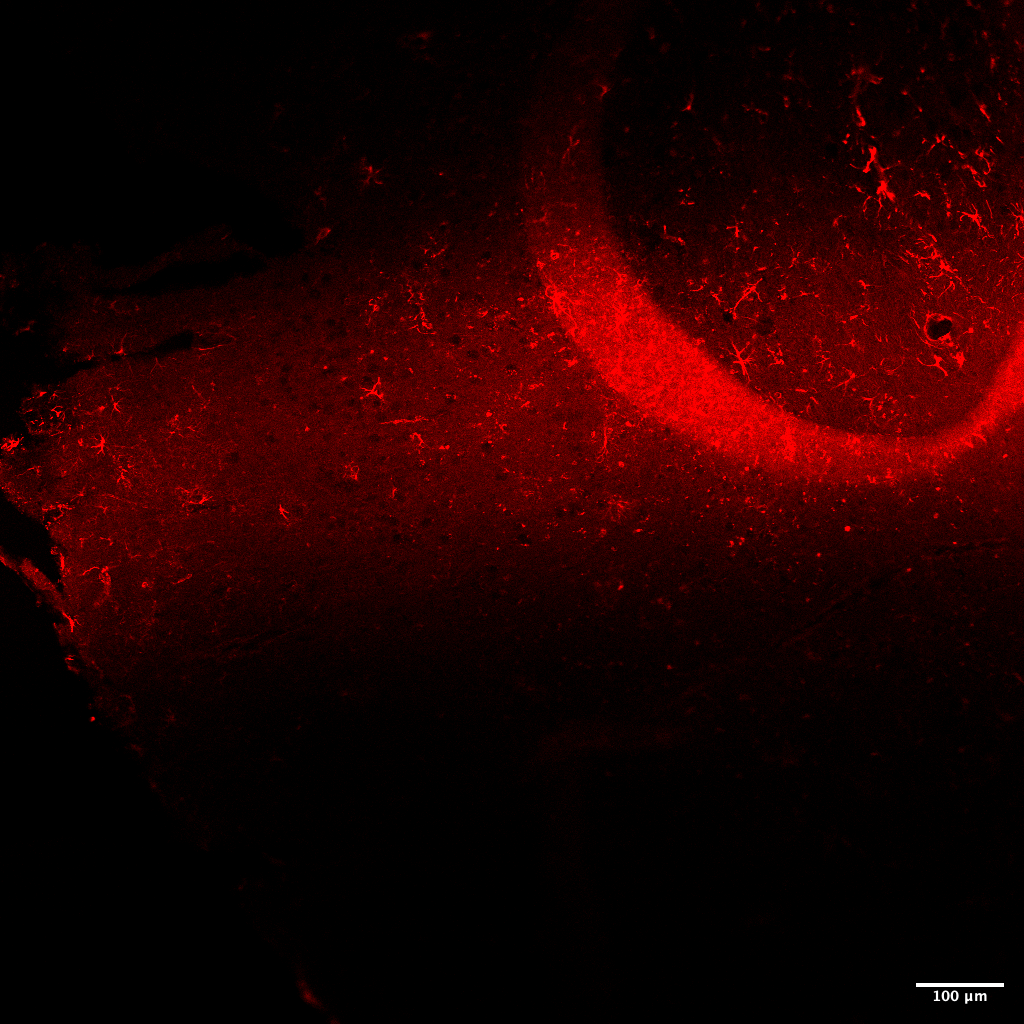

Supplement: Supplementary file 10 — EV and Appendix Figure Source Data [file 44321_2024_111_MOESM10_ESM.zip › Source Data for Expanded View and Appendix/EMM-2024-19843_SourceData-FigureEV4/EV4A/GFAP IHC - KO+NDUFS3.tiff]

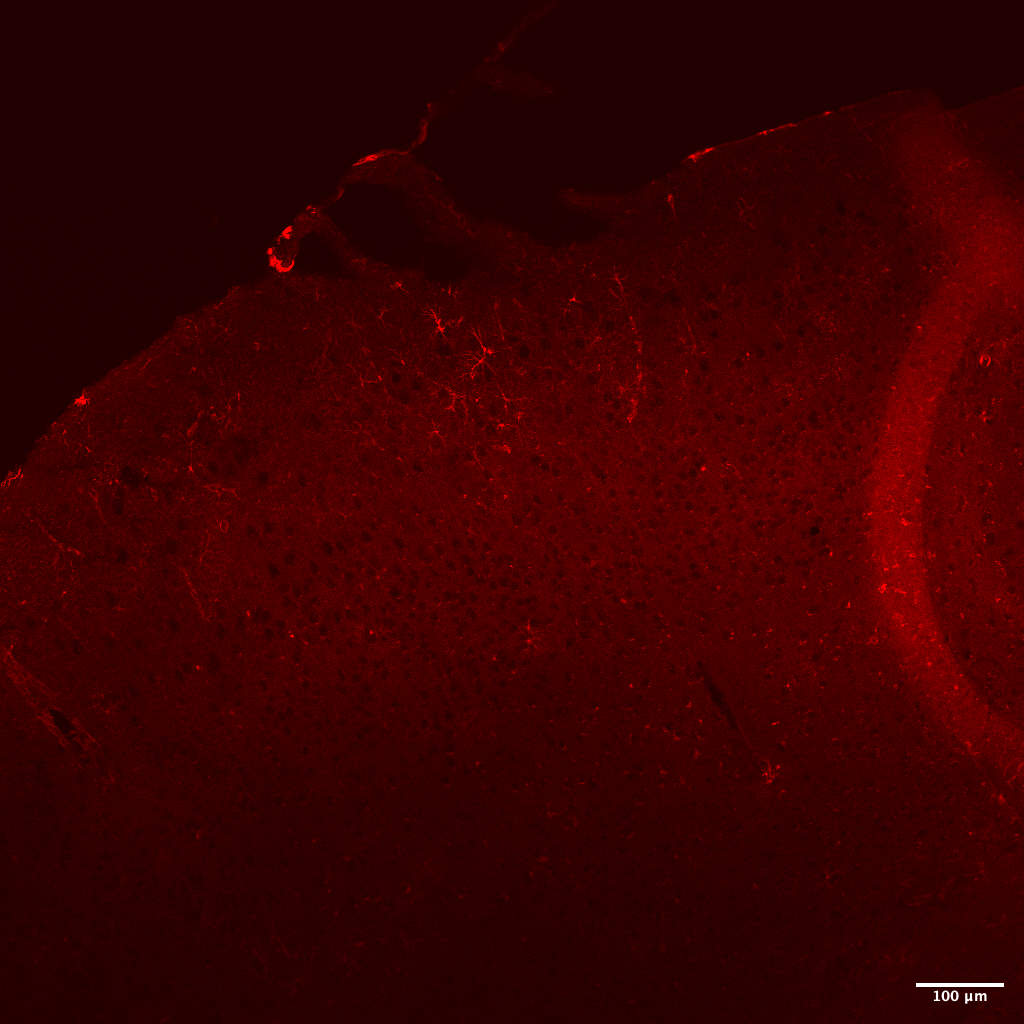

Supplement: Supplementary file 10 — EV and Appendix Figure Source Data [file 44321_2024_111_MOESM10_ESM.zip › Source Data for Expanded View and Appendix/EMM-2024-19843_SourceData-FigureEV4/EV4A/GFAP IHC - WT.tiff]

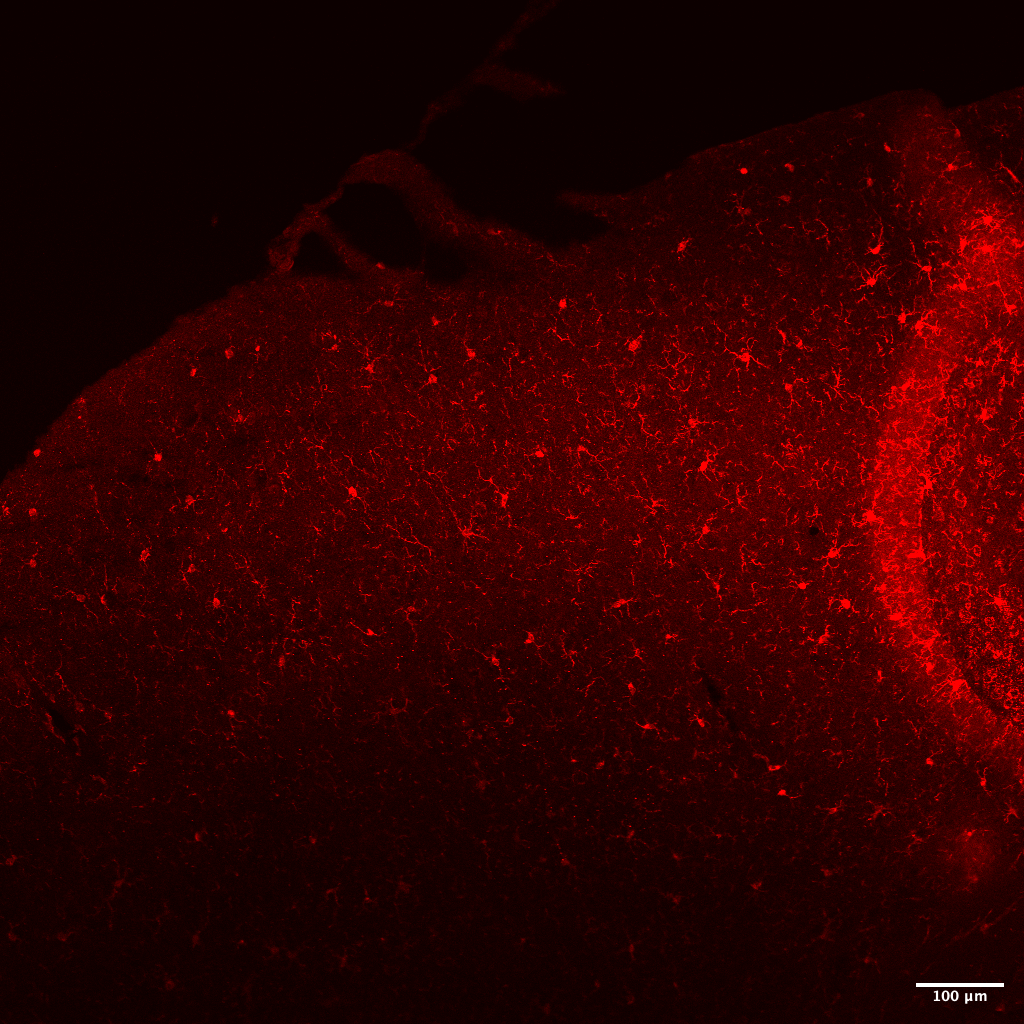

Supplement: Supplementary file 10 — EV and Appendix Figure Source Data [file 44321_2024_111_MOESM10_ESM.zip › Source Data for Expanded View and Appendix/EMM-2024-19843_SourceData-FigureEV4/EV4B/IBA1 IHC - WT.tiff]

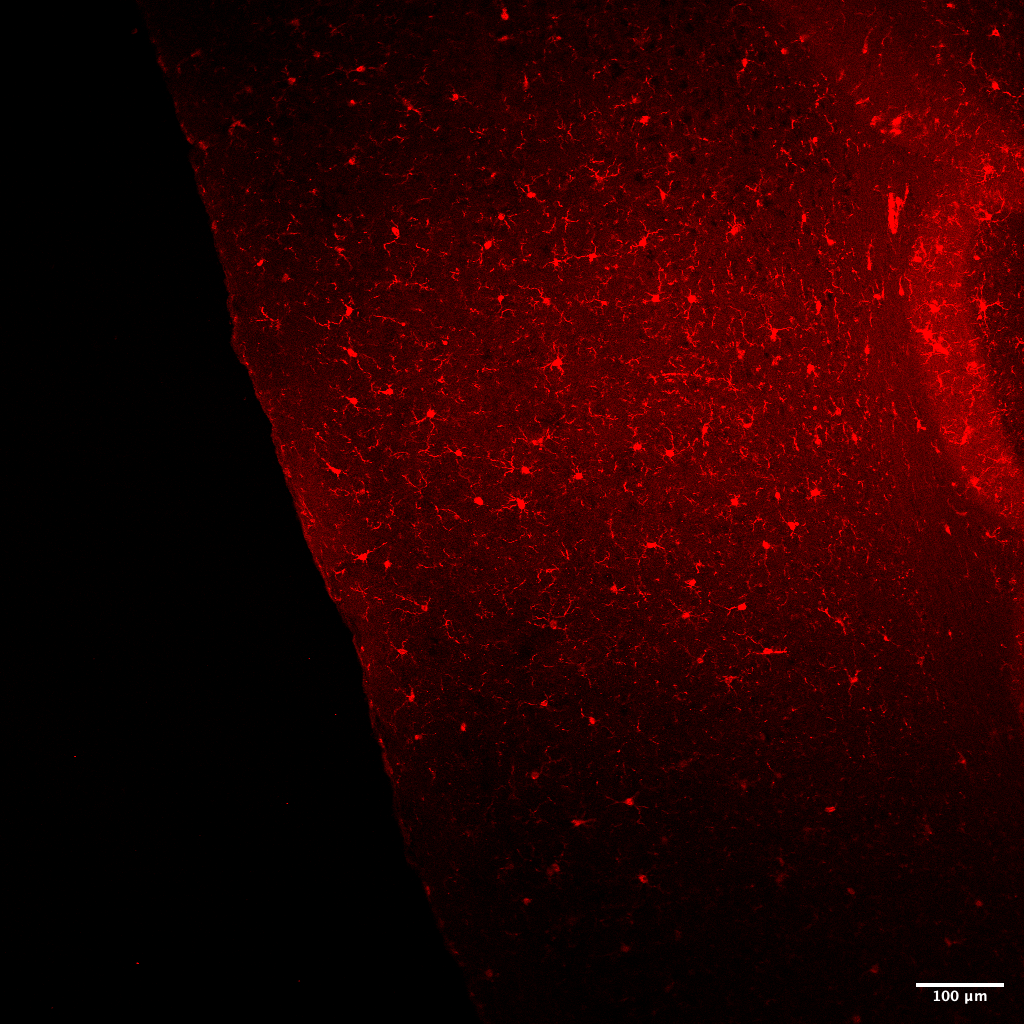

Supplement: Supplementary file 10 — EV and Appendix Figure Source Data [file 44321_2024_111_MOESM10_ESM.zip › Source Data for Expanded View and Appendix/EMM-2024-19843_SourceData-FigureEV4/EV4B/IBA1 IHC - KO+NDUFS3.tiff]

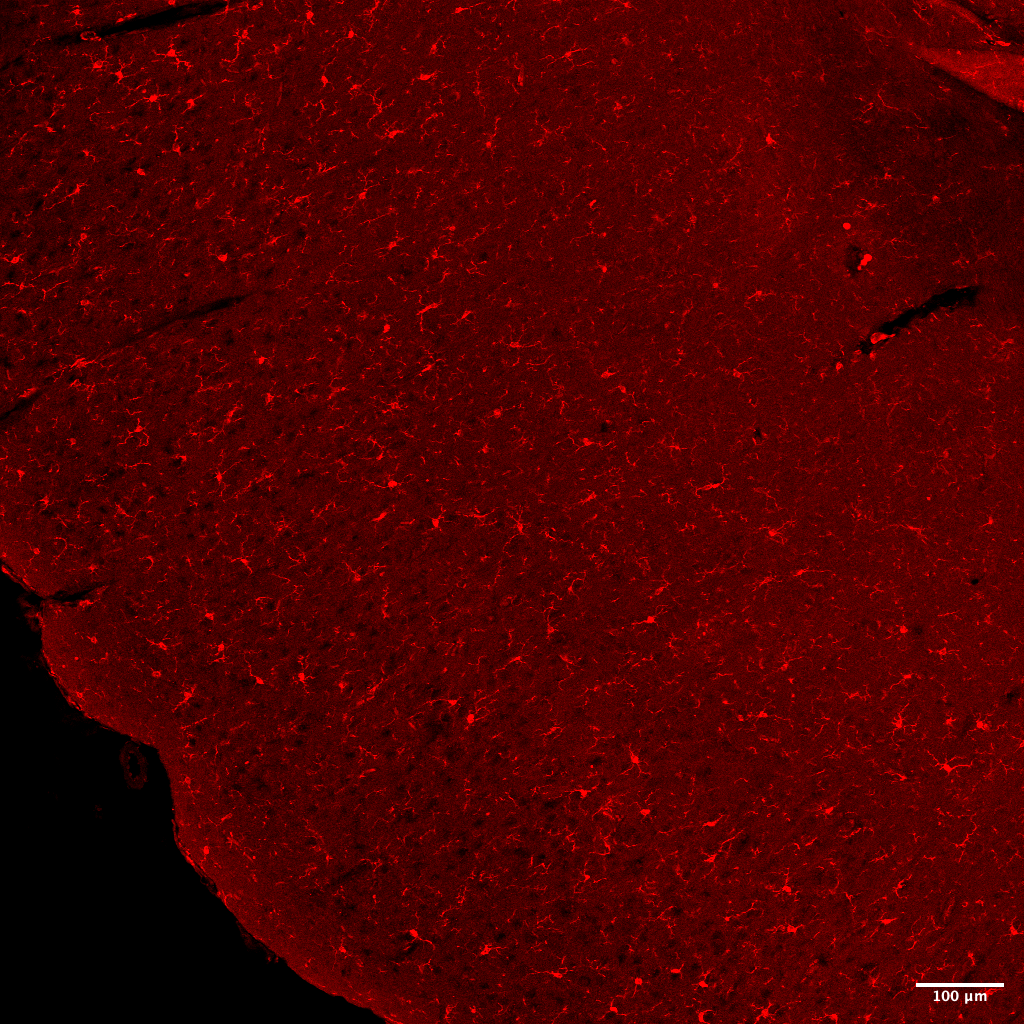

Supplement: Supplementary file 10 — EV and Appendix Figure Source Data [file 44321_2024_111_MOESM10_ESM.zip › Source Data for Expanded View and Appendix/EMM-2024-19843_SourceData-FigureEV4/EV4B/IBA1 IHC - KO+COX10.tiff]

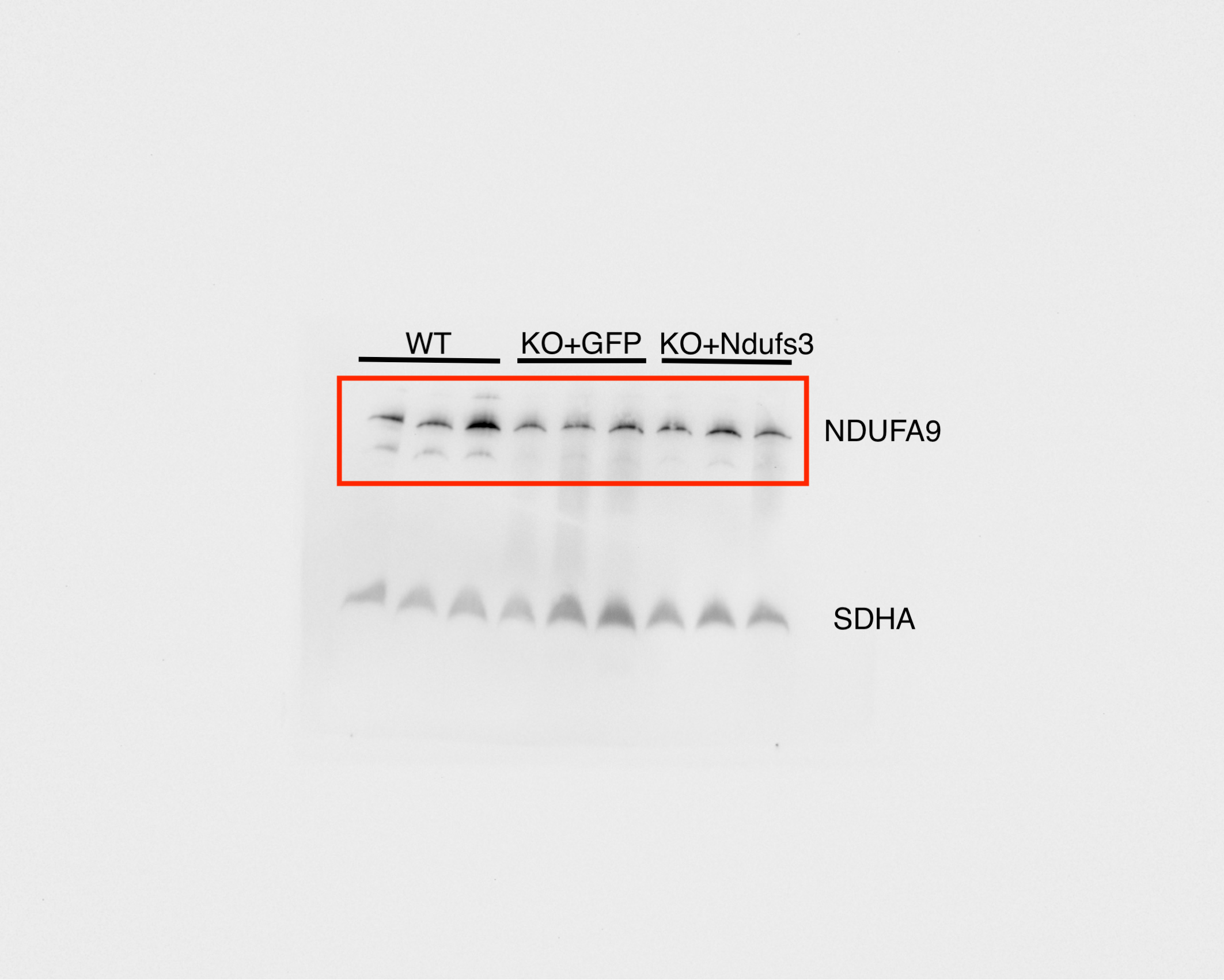

Supplement: Supplementary file 10 — EV and Appendix Figure Source Data [file 44321_2024_111_MOESM10_ESM.zip › Source Data for Expanded View and Appendix/EMM-2024-19843_SourceData-FigureEV1/EV1I/western - NDUFA9.tiff]

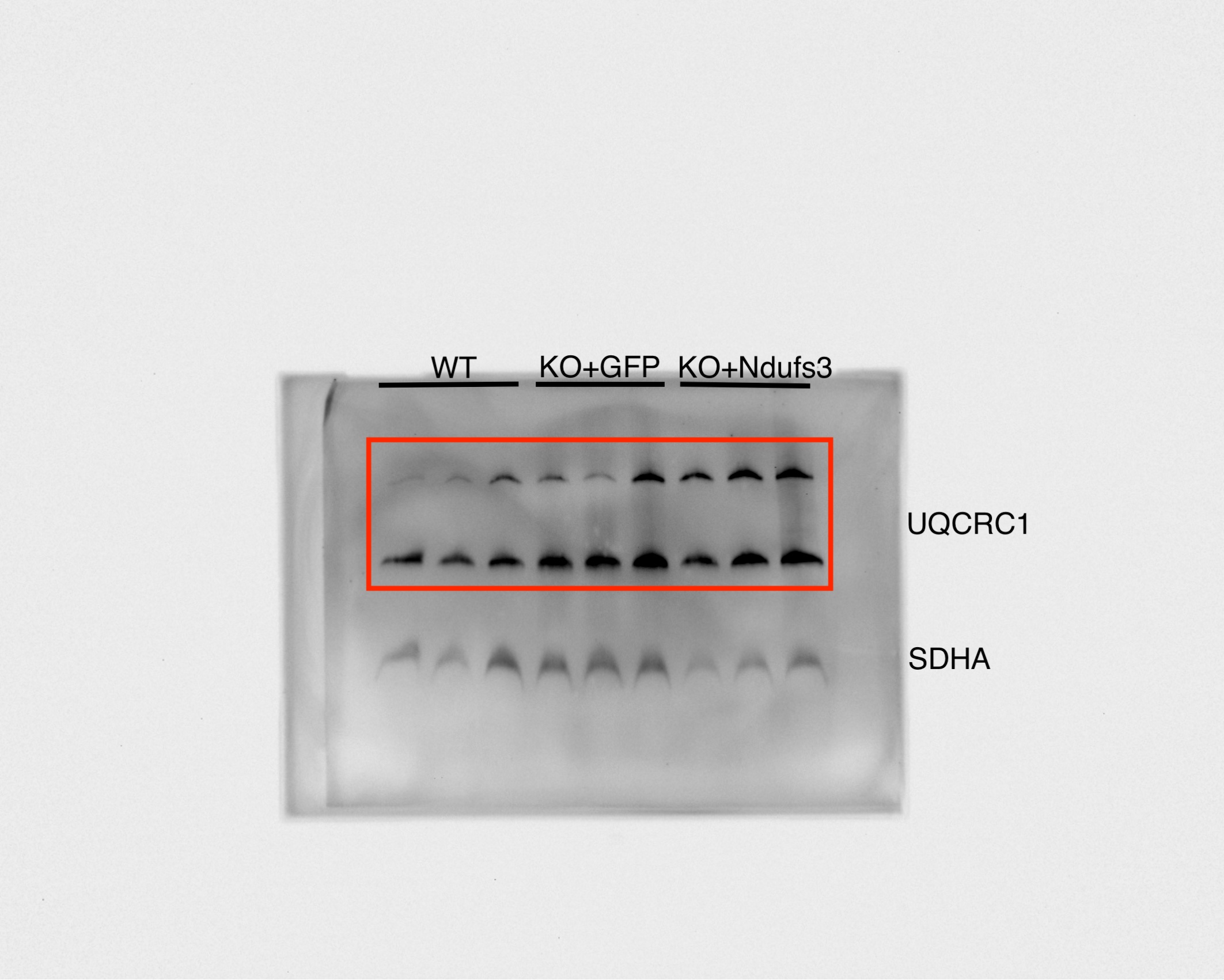

Supplement: Supplementary file 10 — EV and Appendix Figure Source Data [file 44321_2024_111_MOESM10_ESM.zip › Source Data for Expanded View and Appendix/EMM-2024-19843_SourceData-FigureEV1/EV1I/western - UQCRC1.tiff]

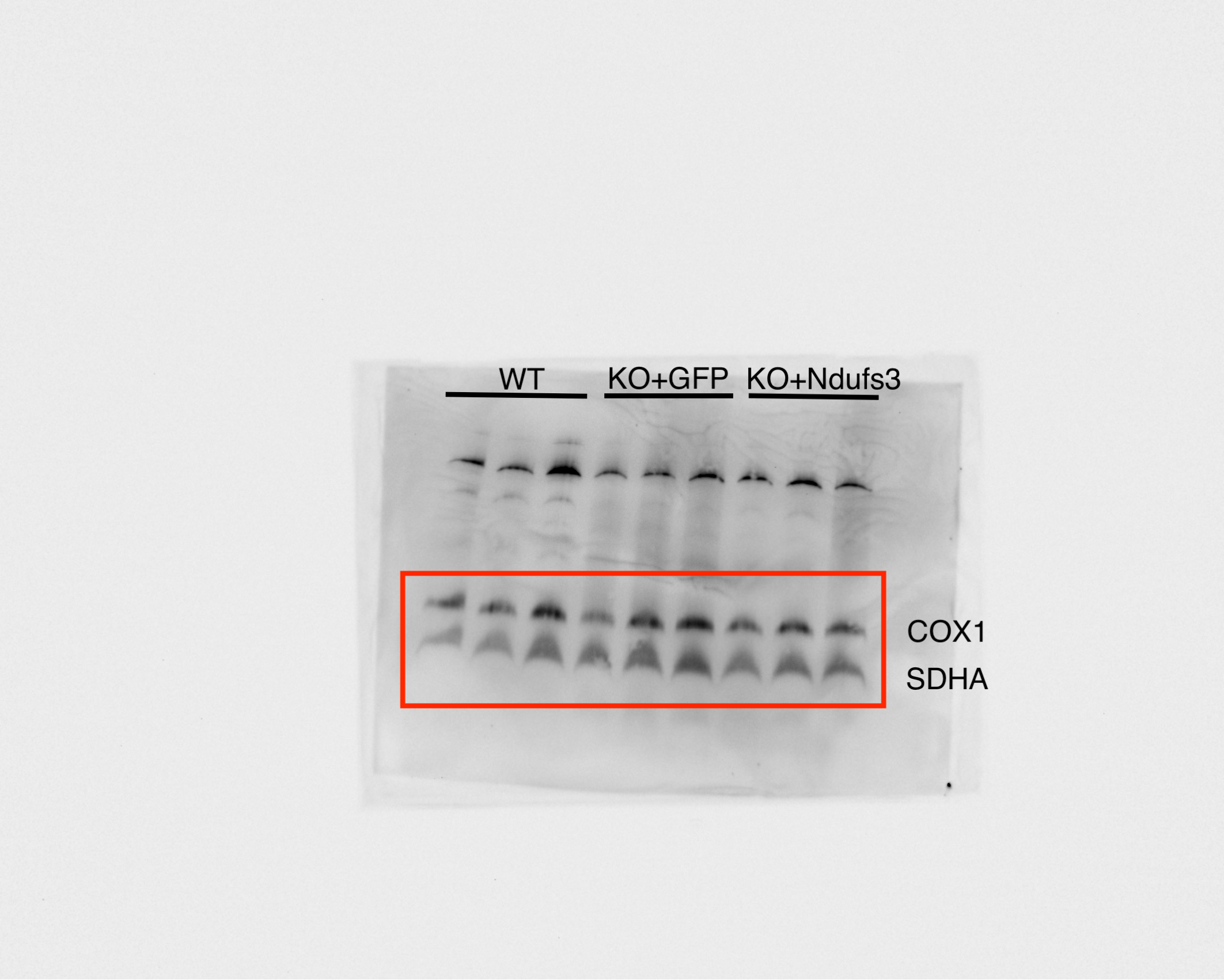

Supplement: Supplementary file 10 — EV and Appendix Figure Source Data [file 44321_2024_111_MOESM10_ESM.zip › Source Data for Expanded View and Appendix/EMM-2024-19843_SourceData-FigureEV1/EV1I/western - COX1_SDHA.tiff]

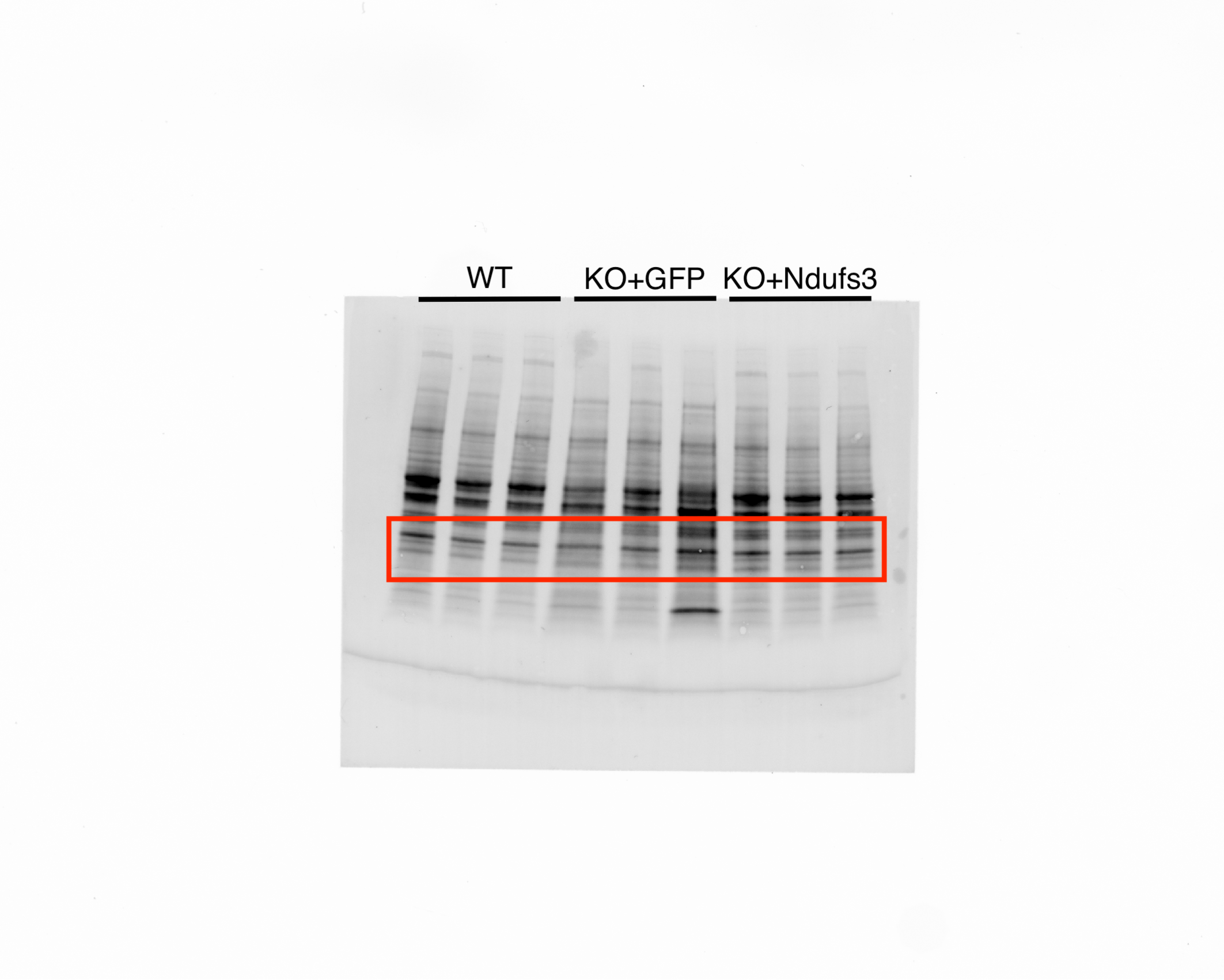

Supplement: Supplementary file 10 — EV and Appendix Figure Source Data [file 44321_2024_111_MOESM10_ESM.zip › Source Data for Expanded View and Appendix/EMM-2024-19843_SourceData-FigureEV1/EV1A/western - Total Protein.tiff]

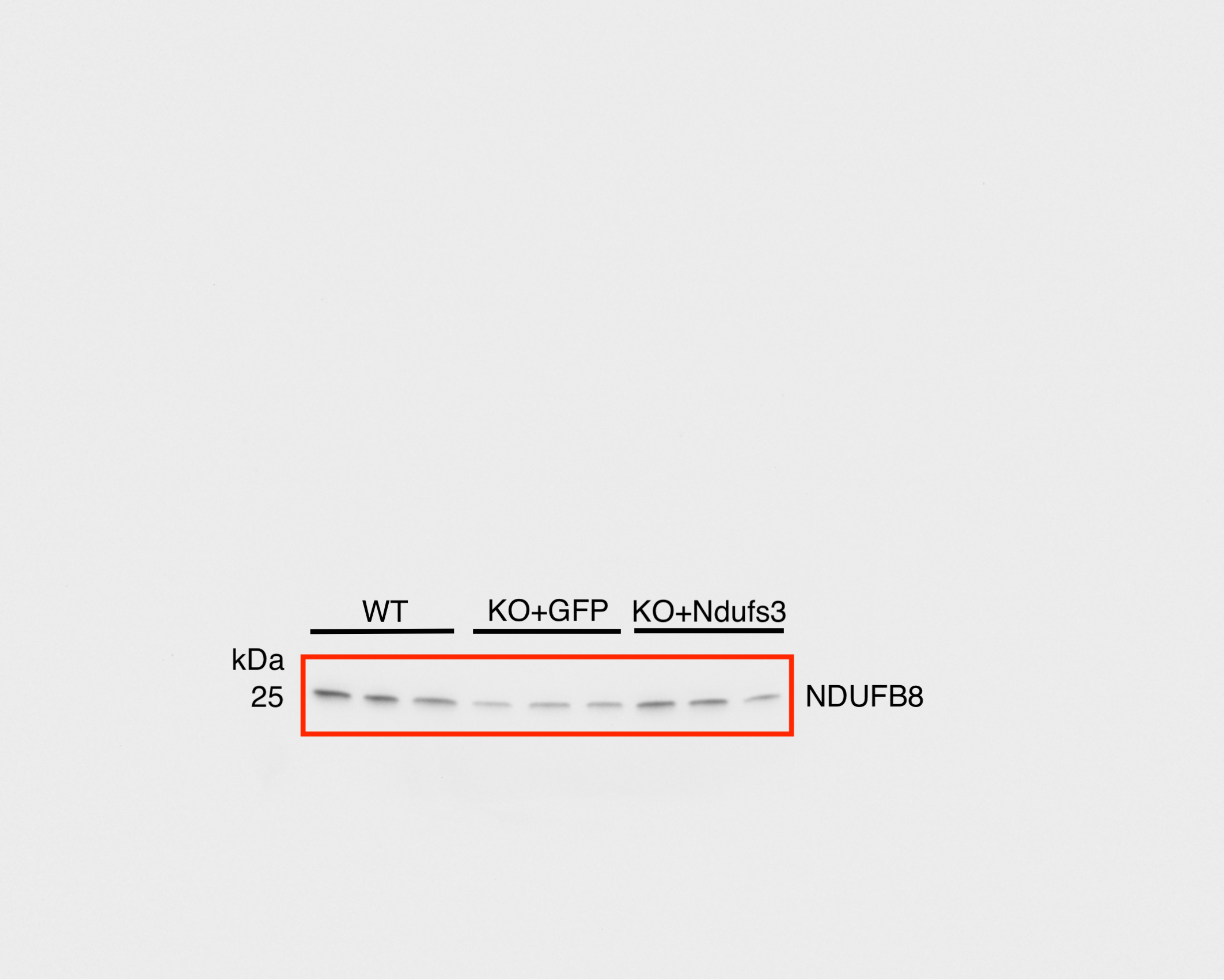

Supplement: Supplementary file 10 — EV and Appendix Figure Source Data [file 44321_2024_111_MOESM10_ESM.zip › Source Data for Expanded View and Appendix/EMM-2024-19843_SourceData-FigureEV1/EV1A/western - NDUFB8.tiff]

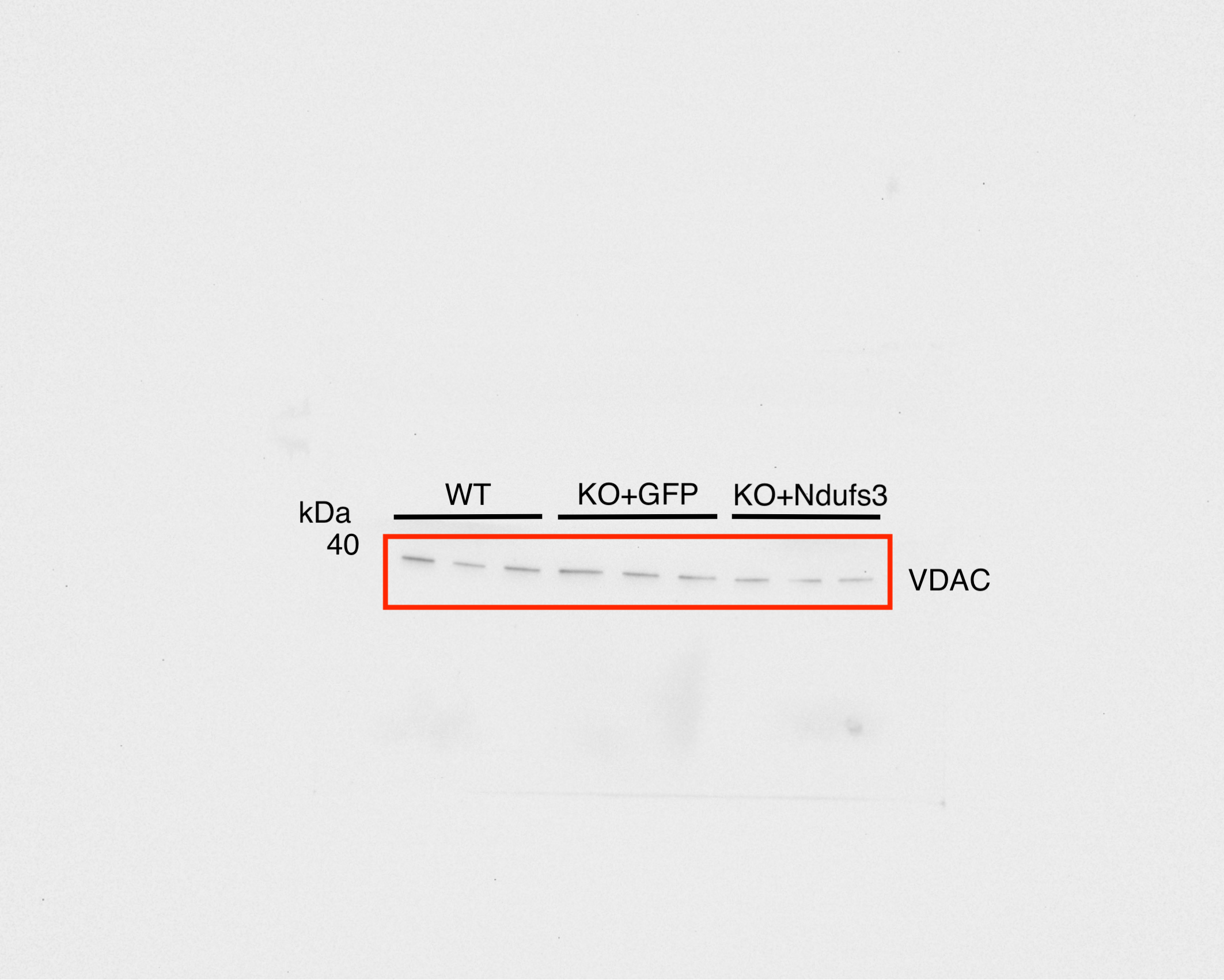

Supplement: Supplementary file 10 — EV and Appendix Figure Source Data [file 44321_2024_111_MOESM10_ESM.zip › Source Data for Expanded View and Appendix/EMM-2024-19843_SourceData-FigureEV1/EV1A/western - VDAC.tiff]

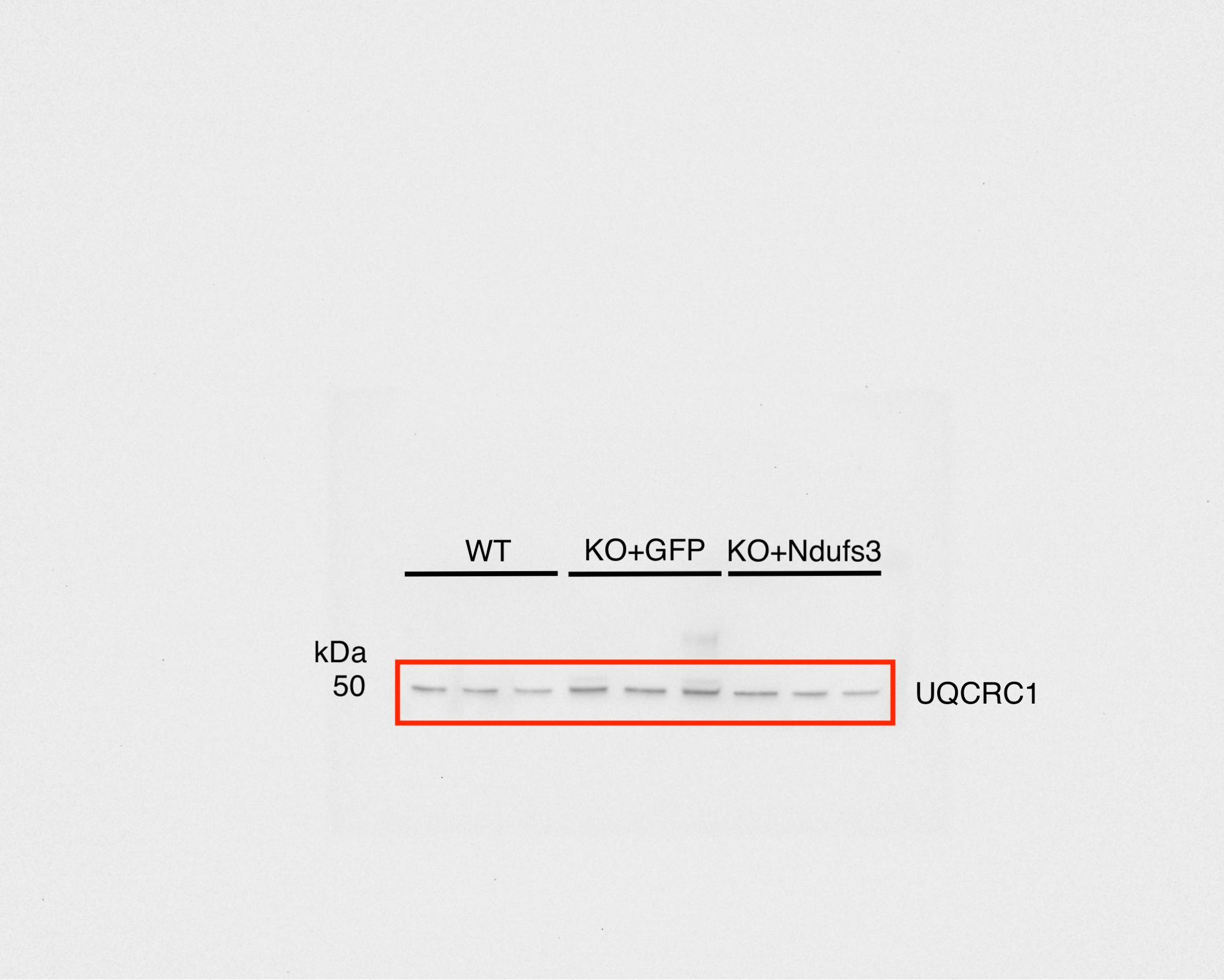

Supplement: Supplementary file 10 — EV and Appendix Figure Source Data [file 44321_2024_111_MOESM10_ESM.zip › Source Data for Expanded View and Appendix/EMM-2024-19843_SourceData-FigureEV1/EV1A/western - UQCRC1.tiff]

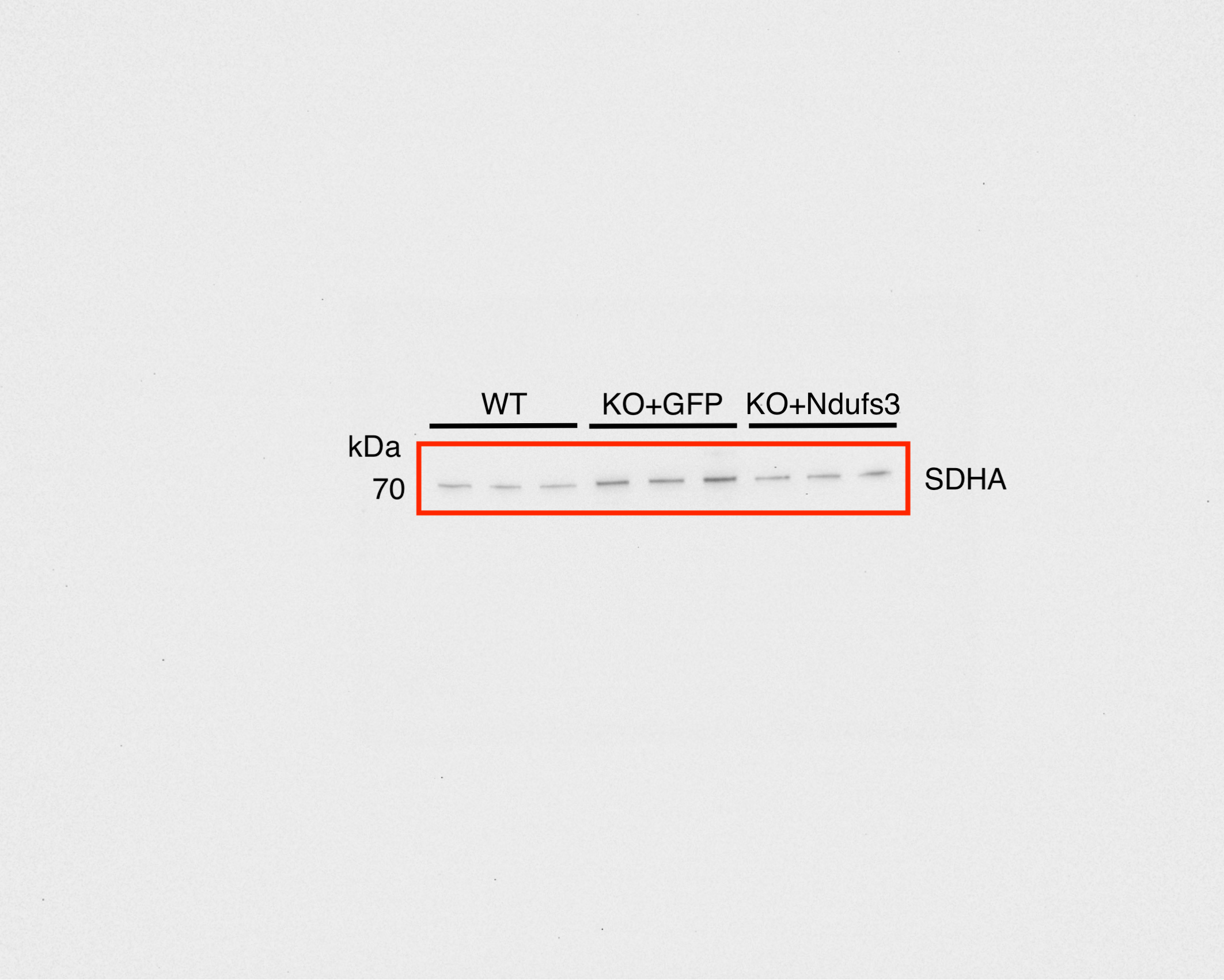

Supplement: Supplementary file 10 — EV and Appendix Figure Source Data [file 44321_2024_111_MOESM10_ESM.zip › Source Data for Expanded View and Appendix/EMM-2024-19843_SourceData-FigureEV1/EV1A/western - SDHA.tiff]

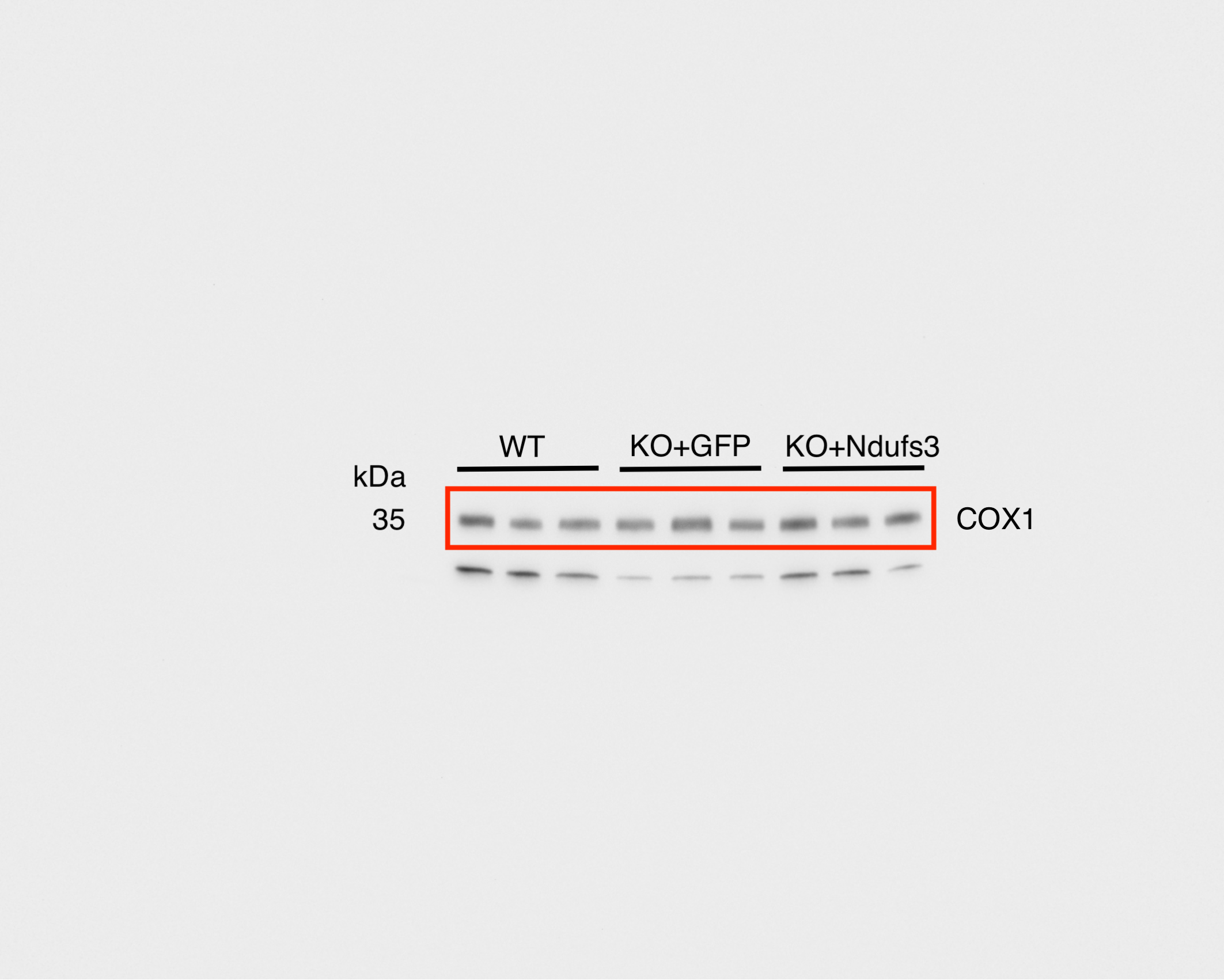

Supplement: Supplementary file 10 — EV and Appendix Figure Source Data [file 44321_2024_111_MOESM10_ESM.zip › Source Data for Expanded View and Appendix/EMM-2024-19843_SourceData-FigureEV1/EV1A/western - COX1.tiff]

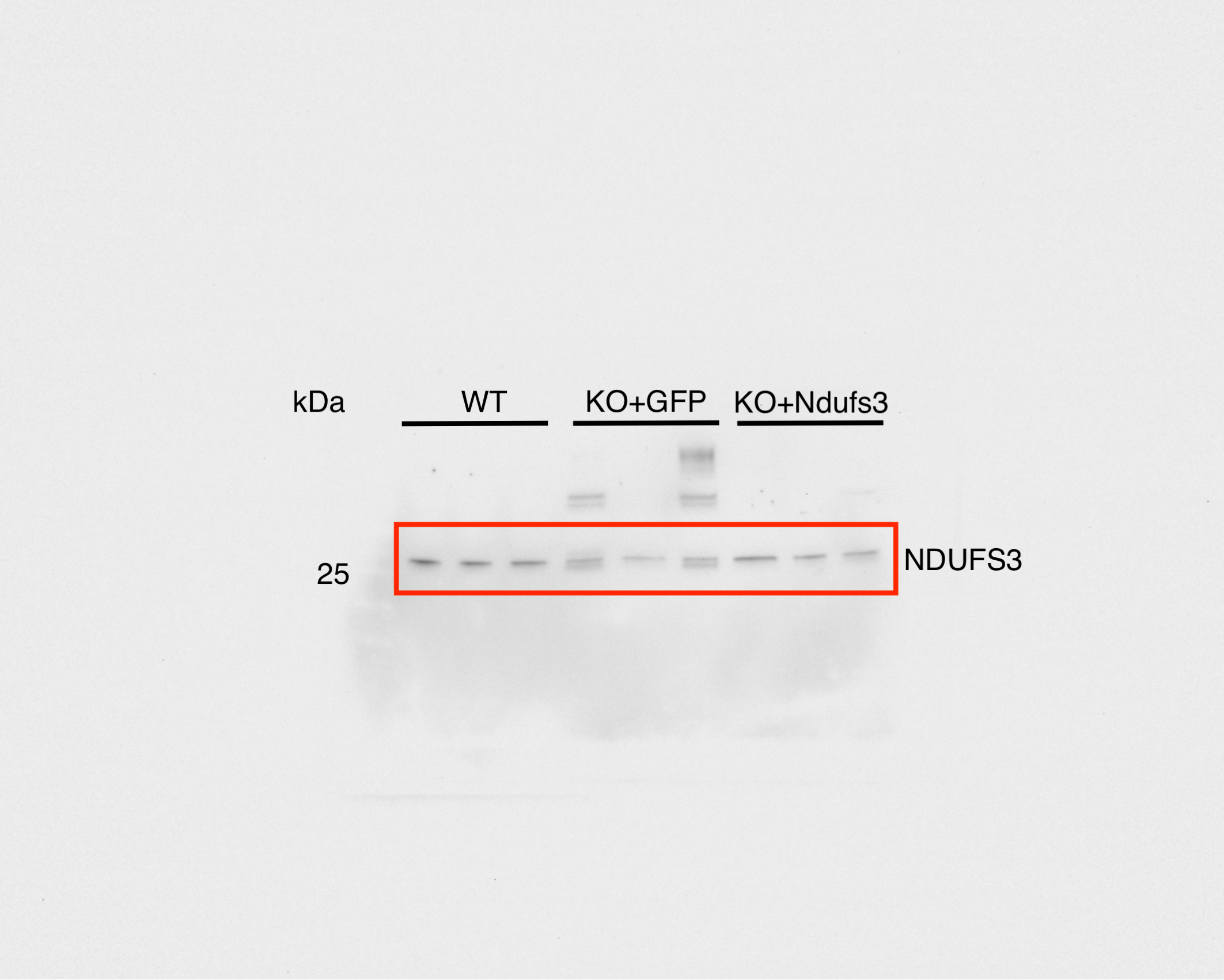

Supplement: Supplementary file 10 — EV and Appendix Figure Source Data [file 44321_2024_111_MOESM10_ESM.zip › Source Data for Expanded View and Appendix/EMM-2024-19843_SourceData-FigureEV1/EV1A/western - NDUFS3.tiff]

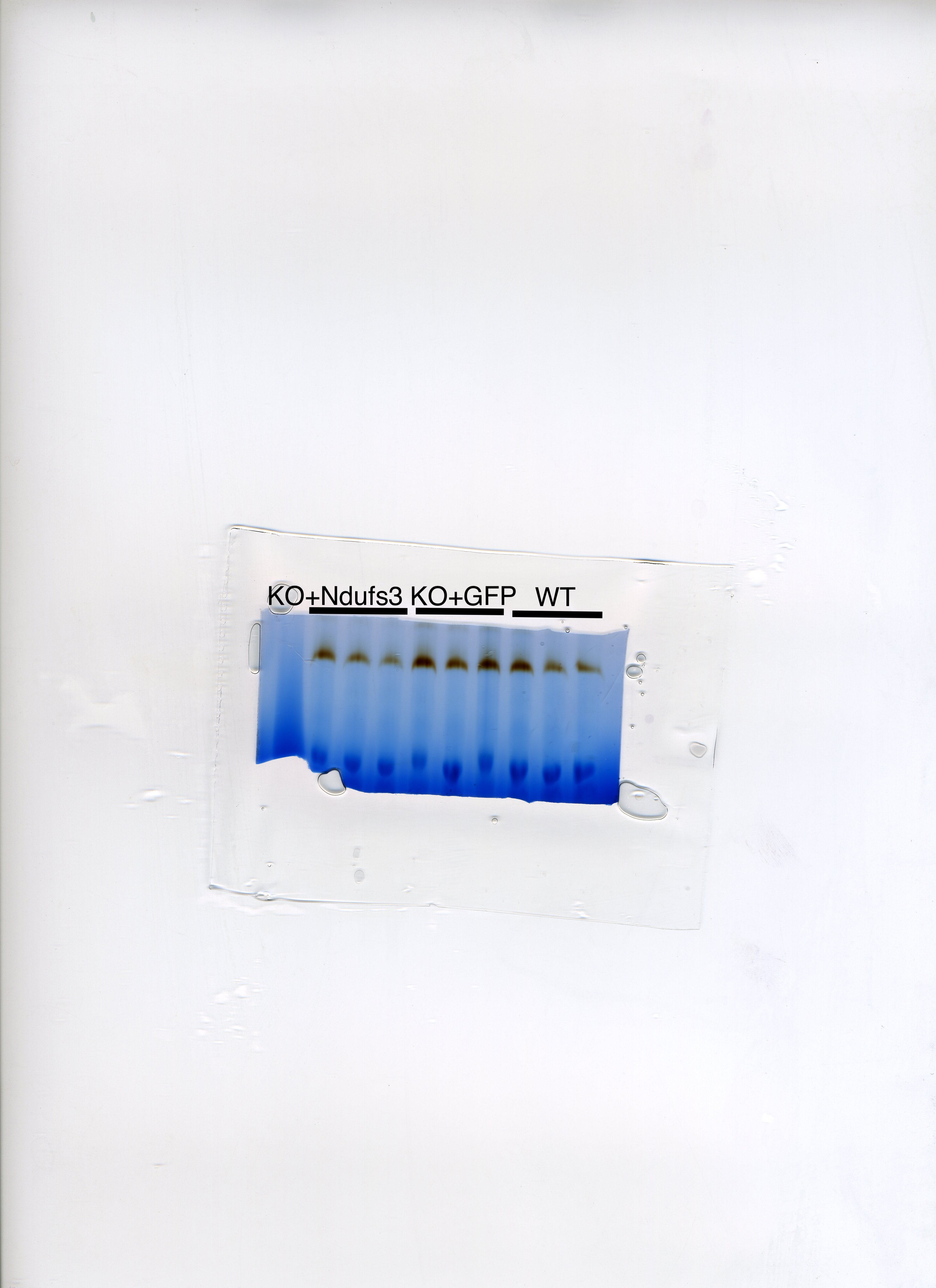

Supplement: Supplementary file 10 — EV and Appendix Figure Source Data [file 44321_2024_111_MOESM10_ESM.zip › Source Data for Expanded View and Appendix/EMM-2024-19843_SourceData-FigureEV1/EV1K/IGA-Complex IV.tiff]

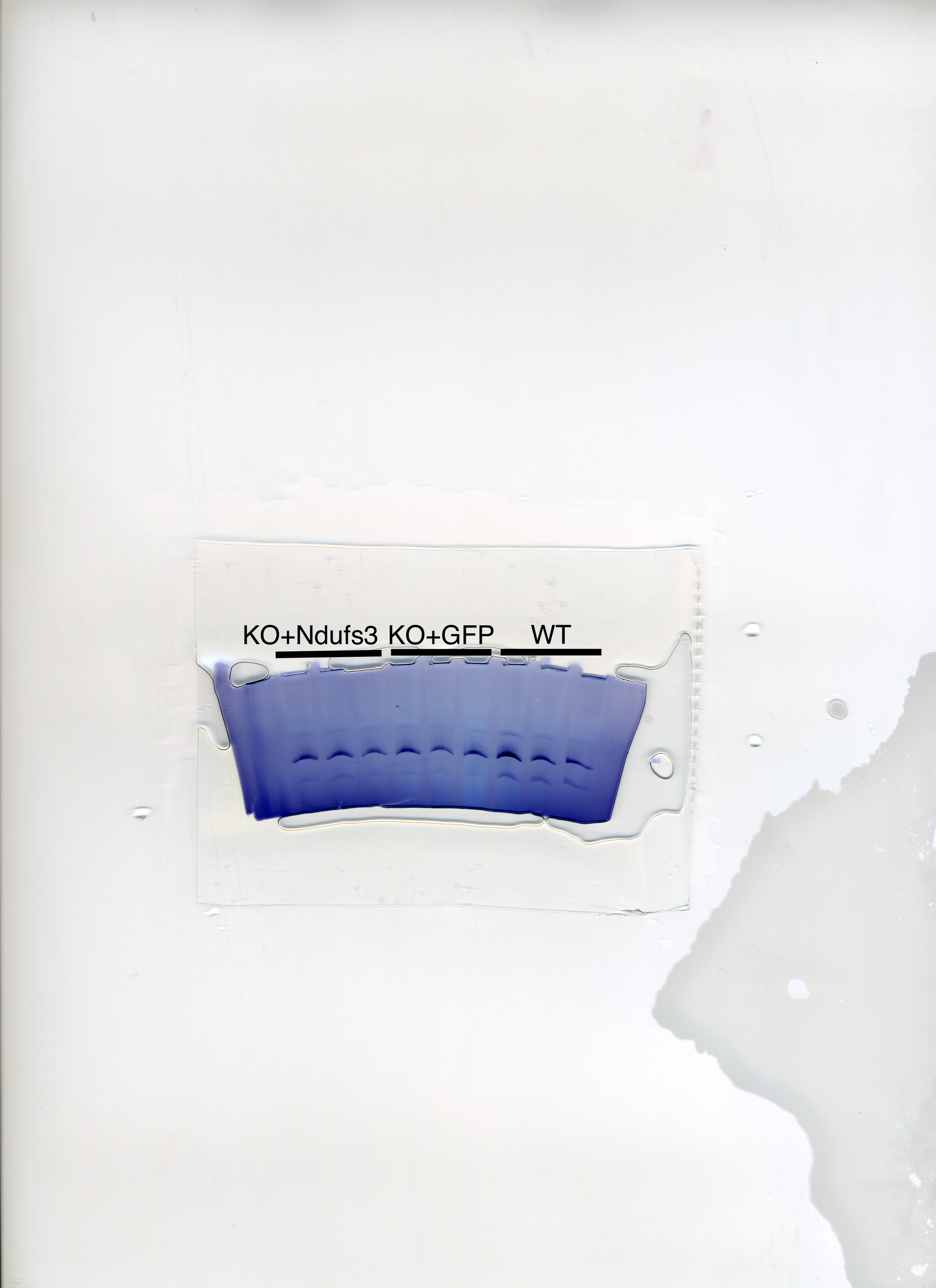

Supplement: Supplementary file 10 — EV and Appendix Figure Source Data [file 44321_2024_111_MOESM10_ESM.zip › Source Data for Expanded View and Appendix/EMM-2024-19843_SourceData-FigureEV1/EV1K/IGA-Complex I.tiff]

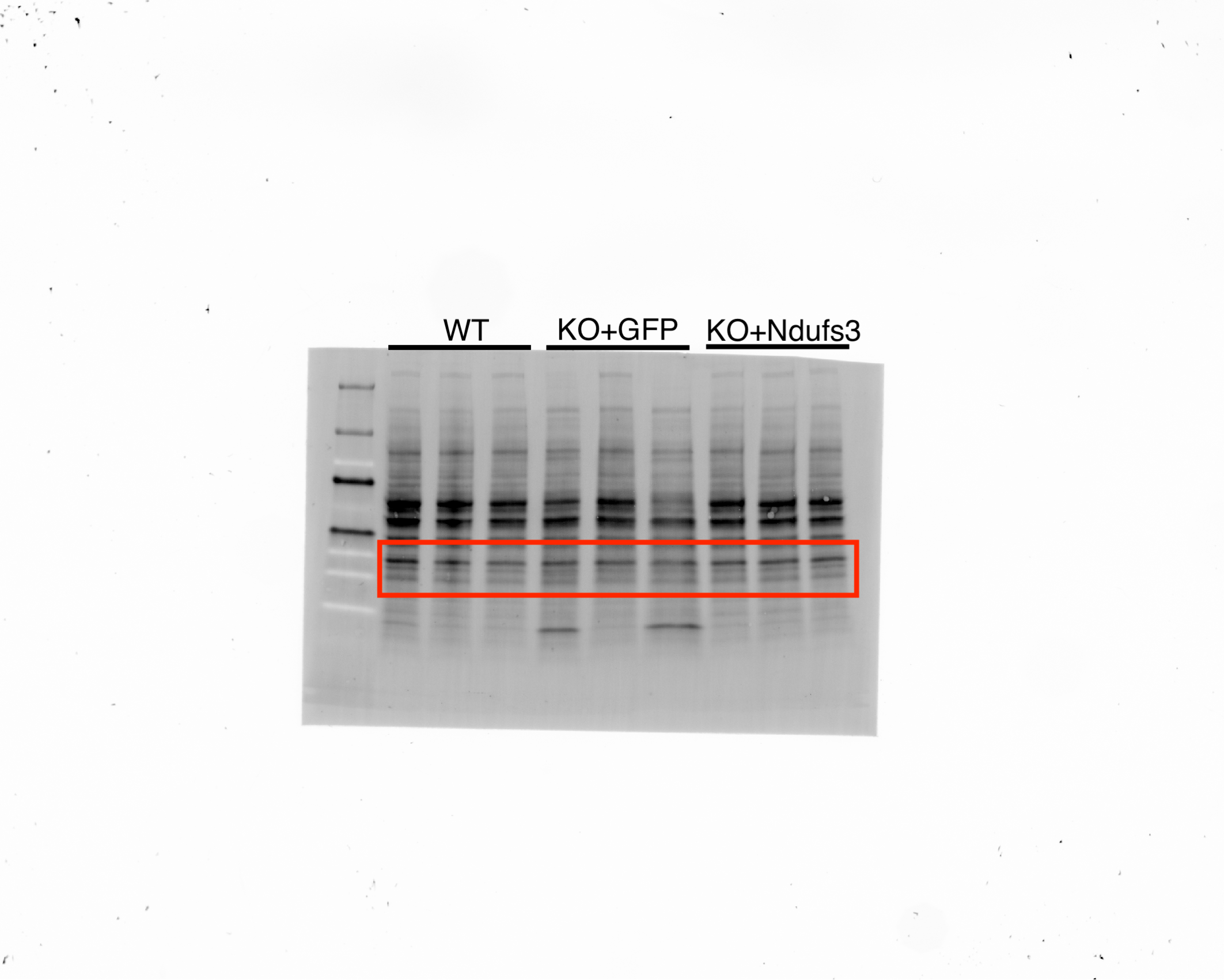

Supplement: Supplementary file 10 — EV and Appendix Figure Source Data [file 44321_2024_111_MOESM10_ESM.zip › Source Data for Expanded View and Appendix/EMM-2024-19843_SourceData-FigureEV1/EV1E/western - Total Protein.tiff]

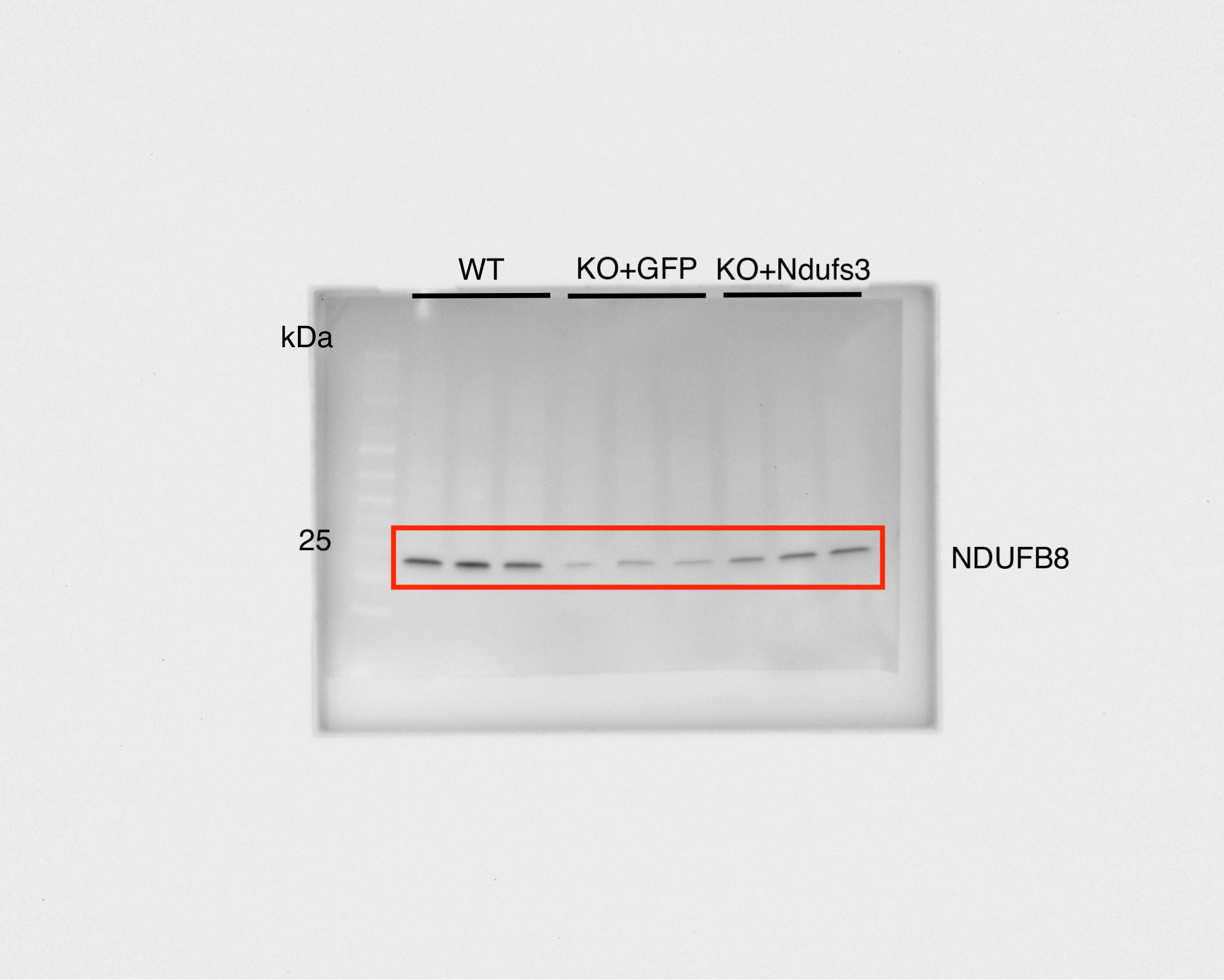

Supplement: Supplementary file 10 — EV and Appendix Figure Source Data [file 44321_2024_111_MOESM10_ESM.zip › Source Data for Expanded View and Appendix/EMM-2024-19843_SourceData-FigureEV1/EV1E/western - NDUFB8.tiff]

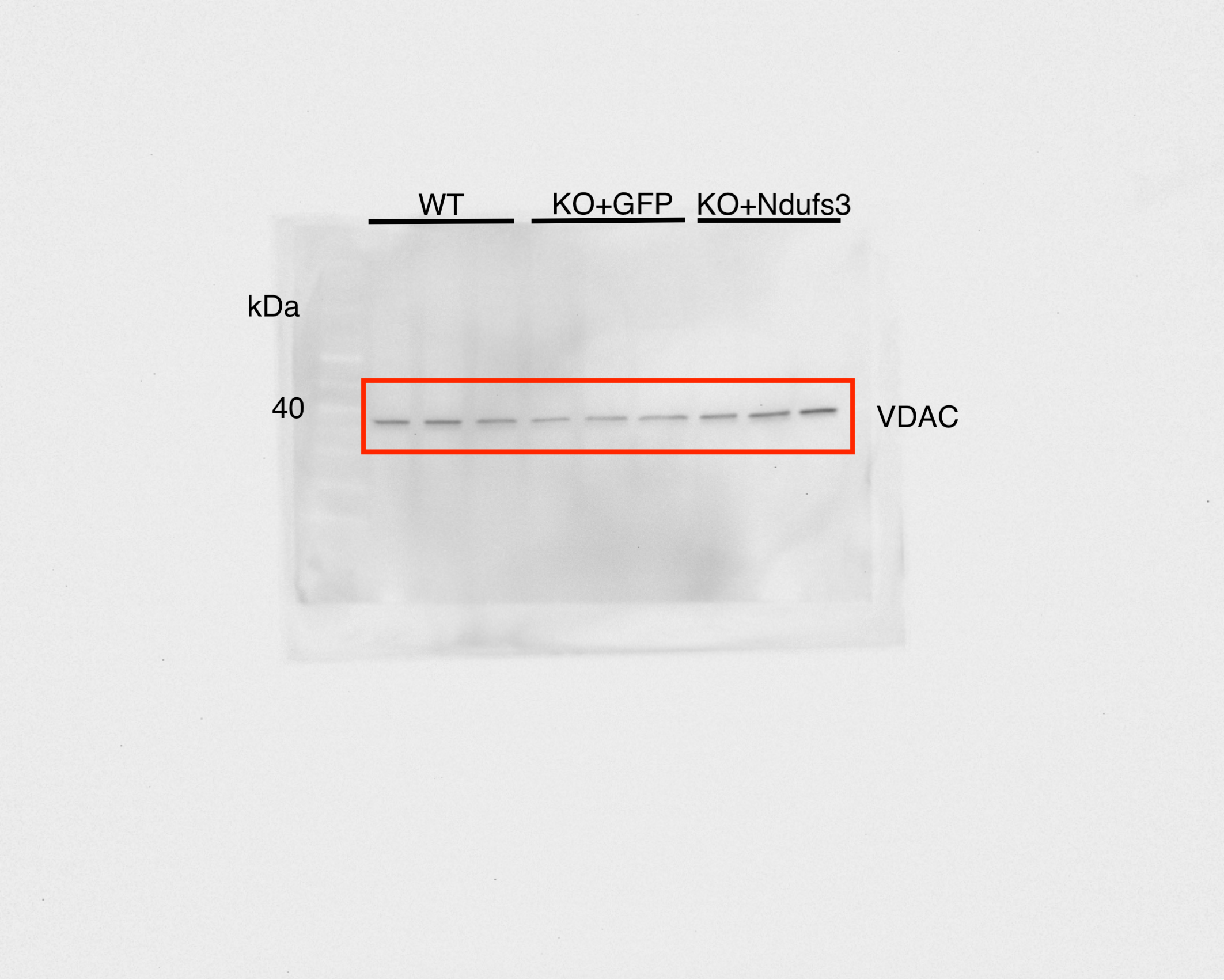

Supplement: Supplementary file 10 — EV and Appendix Figure Source Data [file 44321_2024_111_MOESM10_ESM.zip › Source Data for Expanded View and Appendix/EMM-2024-19843_SourceData-FigureEV1/EV1E/western - VDAC.tiff]

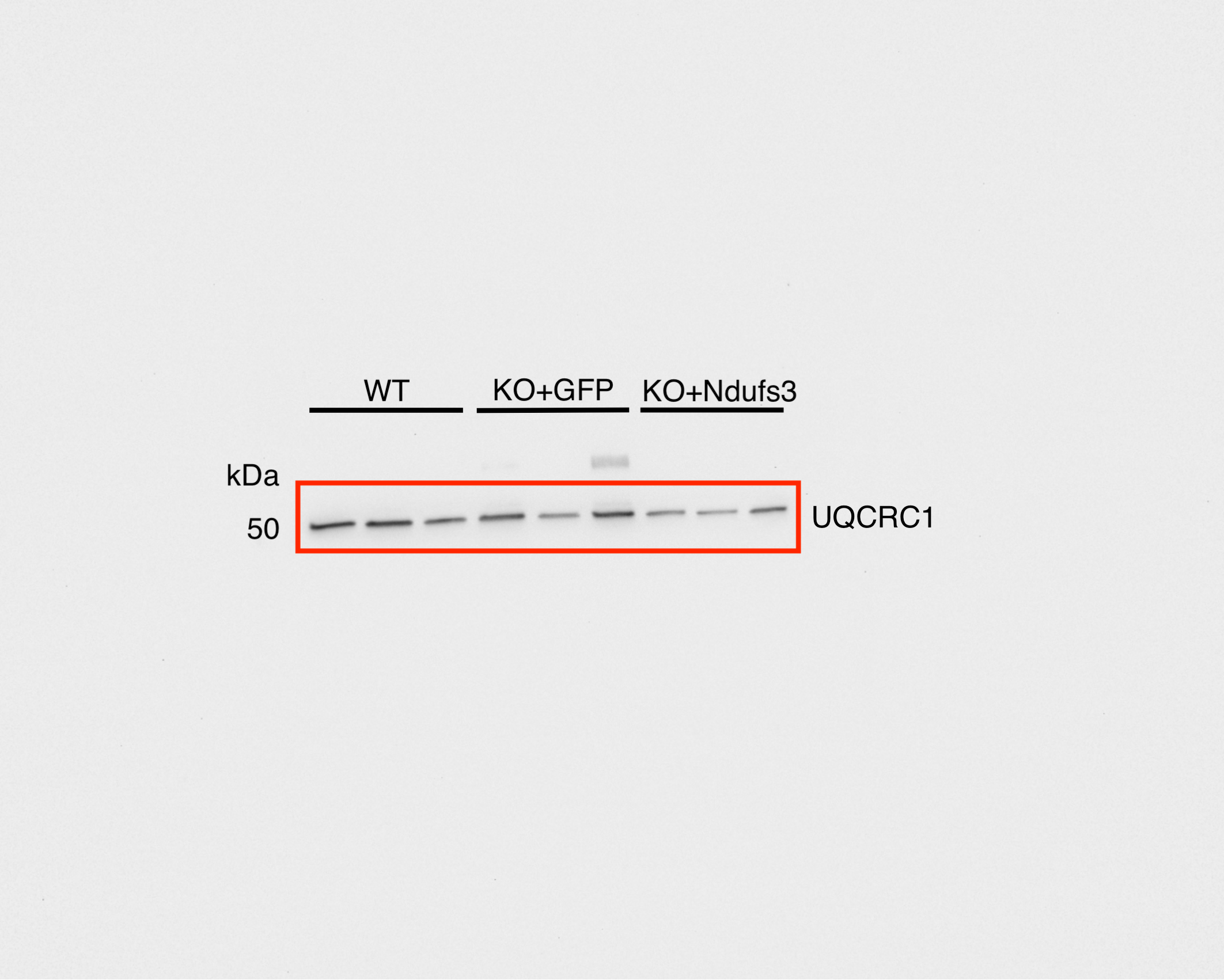

Supplement: Supplementary file 10 — EV and Appendix Figure Source Data [file 44321_2024_111_MOESM10_ESM.zip › Source Data for Expanded View and Appendix/EMM-2024-19843_SourceData-FigureEV1/EV1E/western - UQCRC1.tiff]

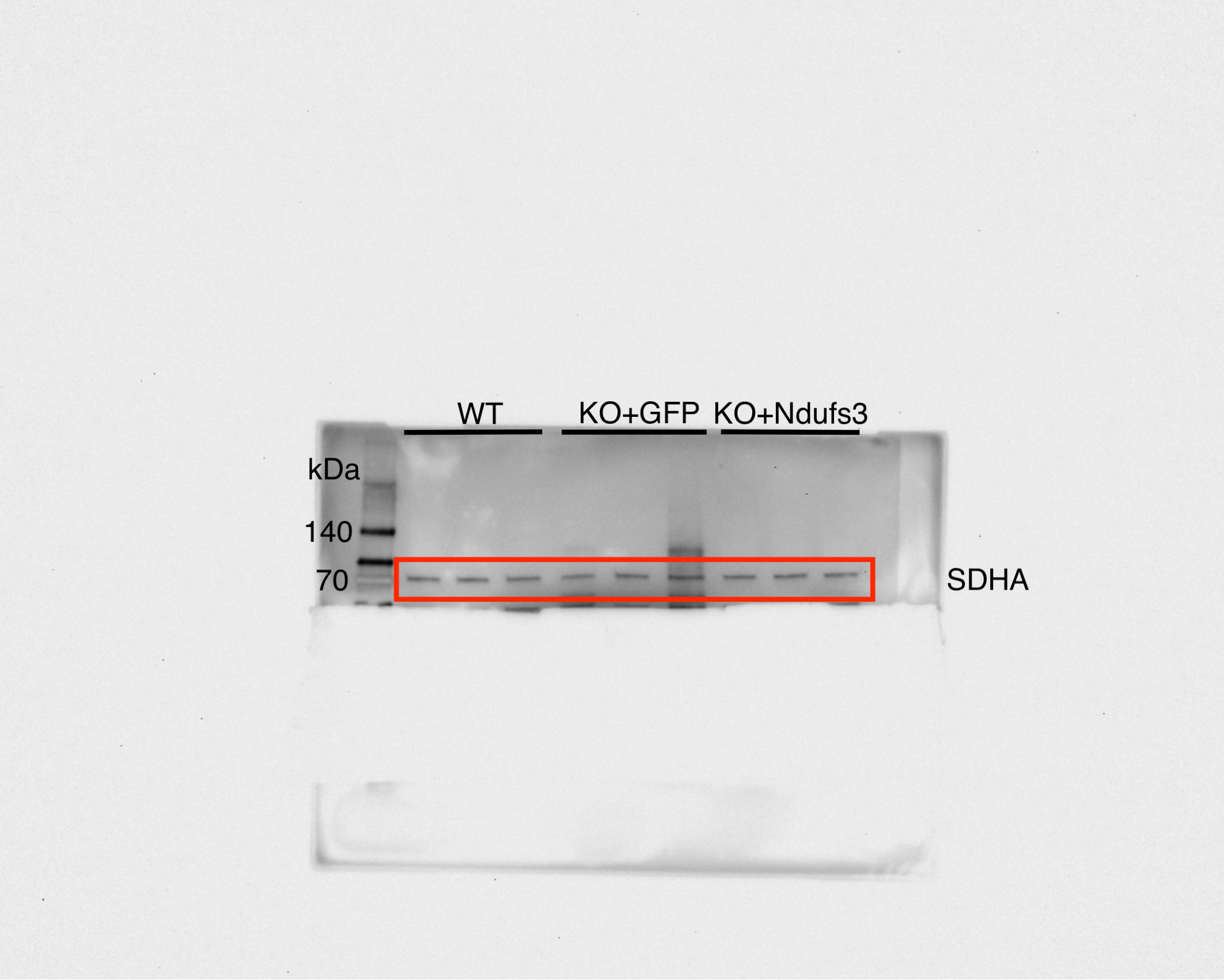

Supplement: Supplementary file 10 — EV and Appendix Figure Source Data [file 44321_2024_111_MOESM10_ESM.zip › Source Data for Expanded View and Appendix/EMM-2024-19843_SourceData-FigureEV1/EV1E/western - SDHA.tiff]

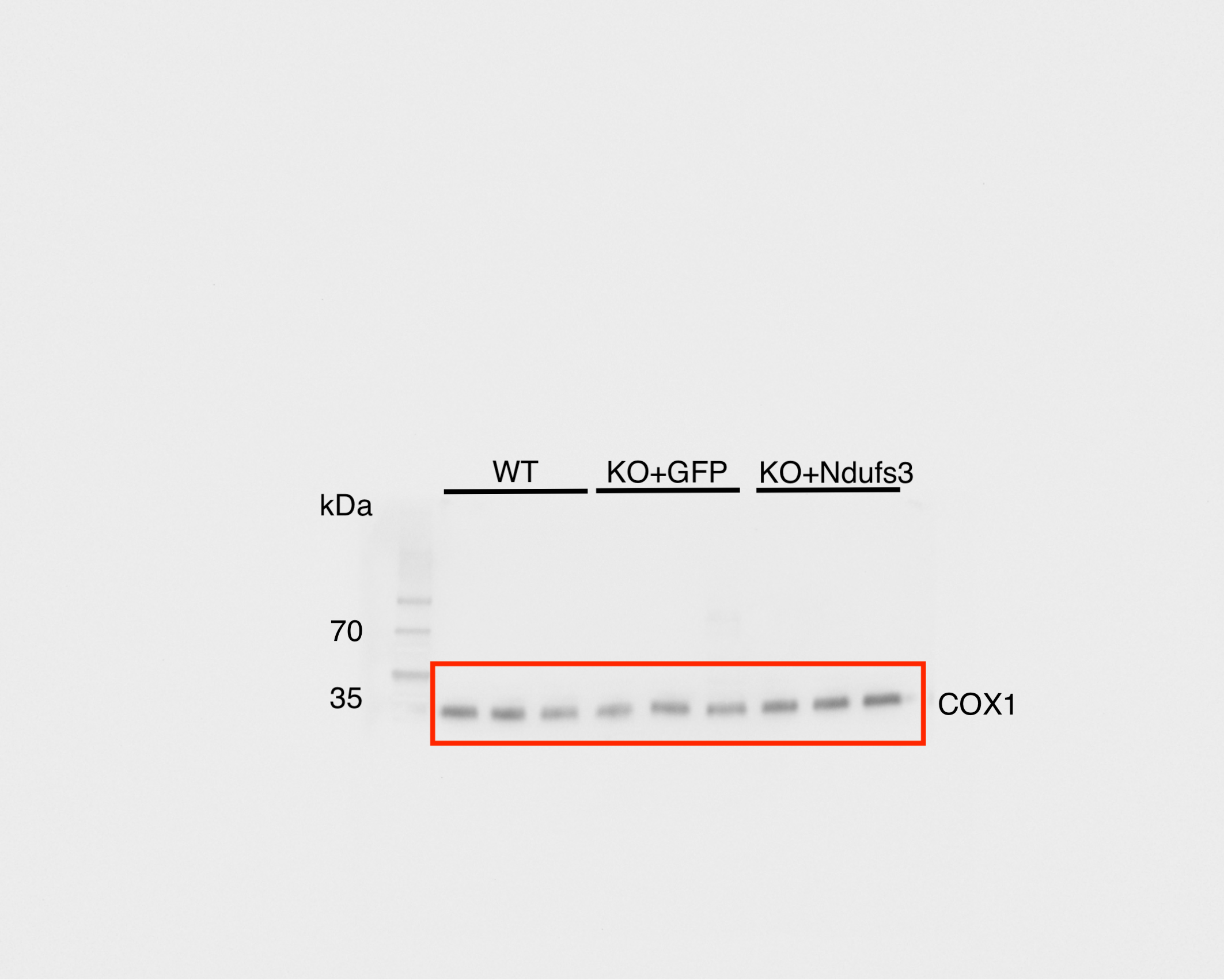

Supplement: Supplementary file 10 — EV and Appendix Figure Source Data [file 44321_2024_111_MOESM10_ESM.zip › Source Data for Expanded View and Appendix/EMM-2024-19843_SourceData-FigureEV1/EV1E/western - COX1.tiff]

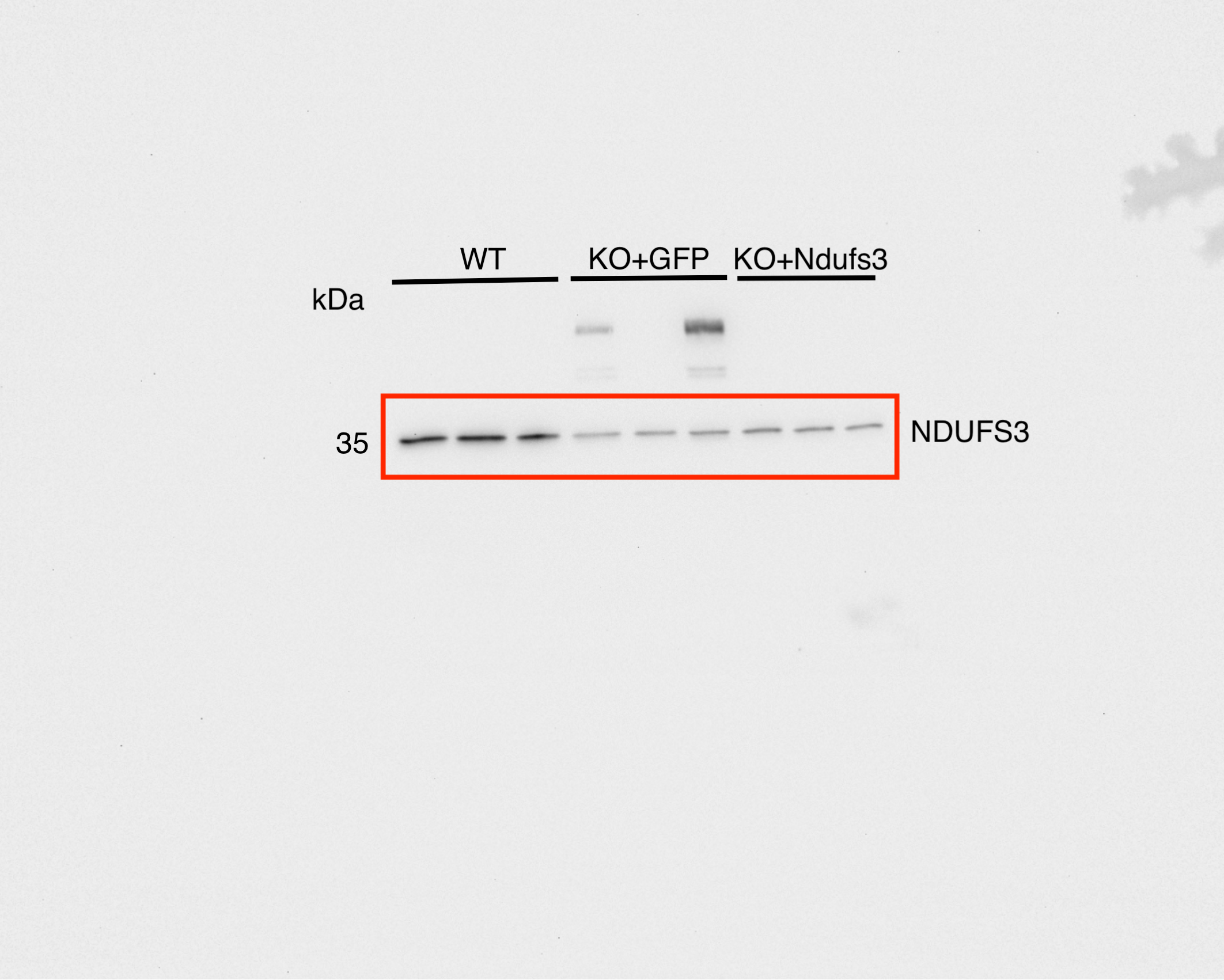

Supplement: Supplementary file 10 — EV and Appendix Figure Source Data [file 44321_2024_111_MOESM10_ESM.zip › Source Data for Expanded View and Appendix/EMM-2024-19843_SourceData-FigureEV1/EV1E/western - NDUFS3.tiff]

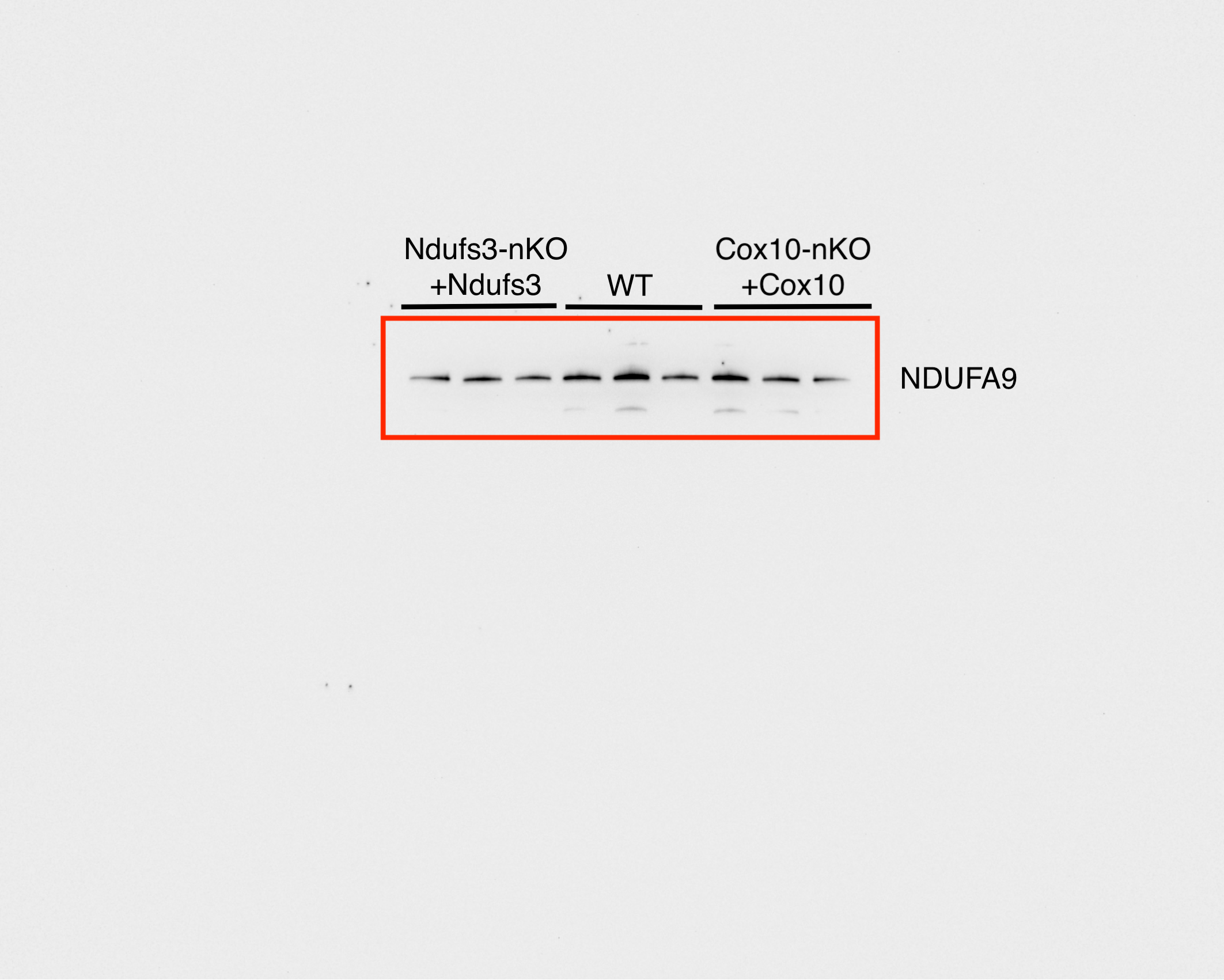

Supplement: Supplementary file 10 — EV and Appendix Figure Source Data [file 44321_2024_111_MOESM10_ESM.zip › Source Data for Expanded View and Appendix/EMM-2024-19843_SourceData-FigureEV3/EV3F/western - NDUFA9.tiff]

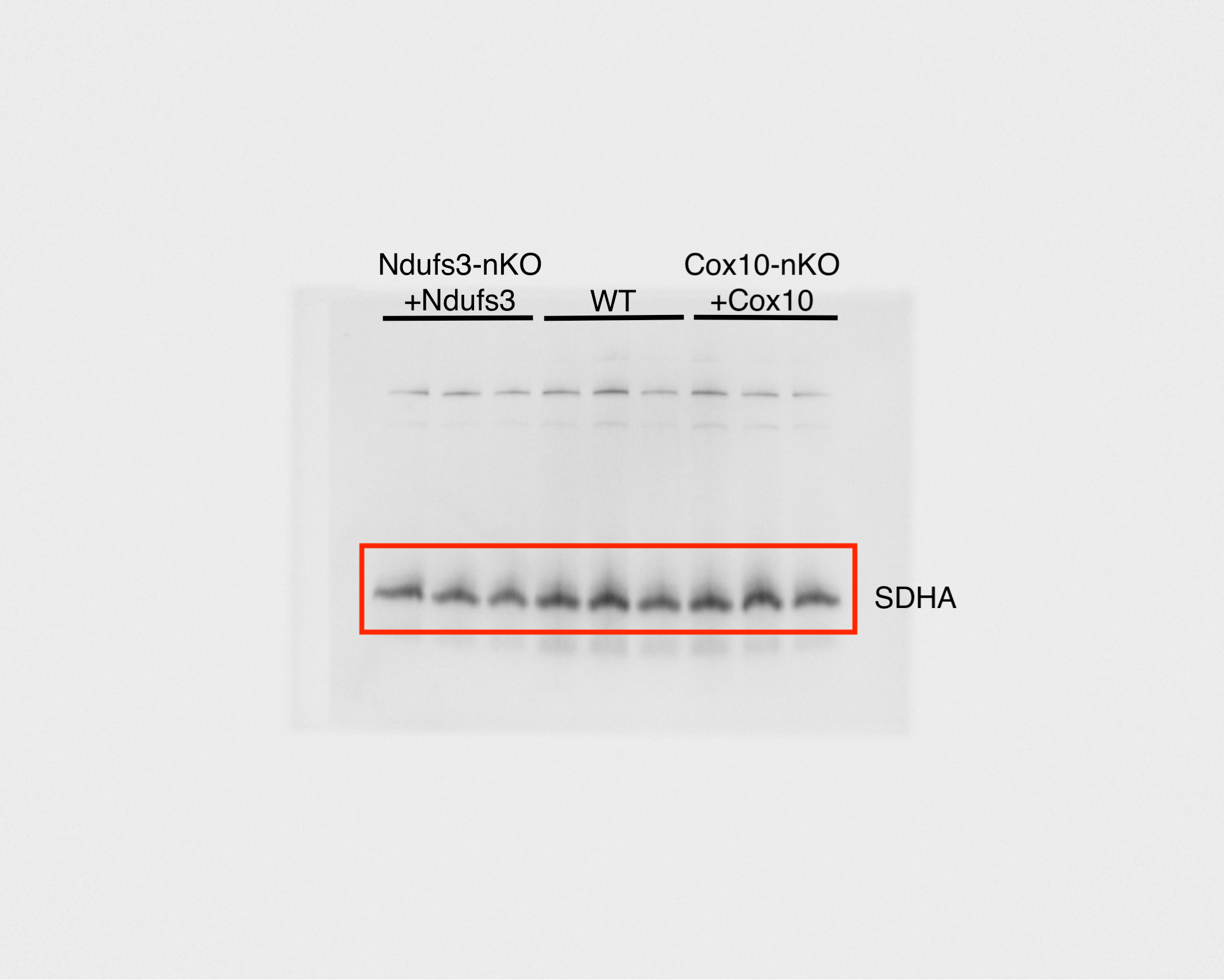

Supplement: Supplementary file 10 — EV and Appendix Figure Source Data [file 44321_2024_111_MOESM10_ESM.zip › Source Data for Expanded View and Appendix/EMM-2024-19843_SourceData-FigureEV3/EV3F/western - SDHA.tiff]

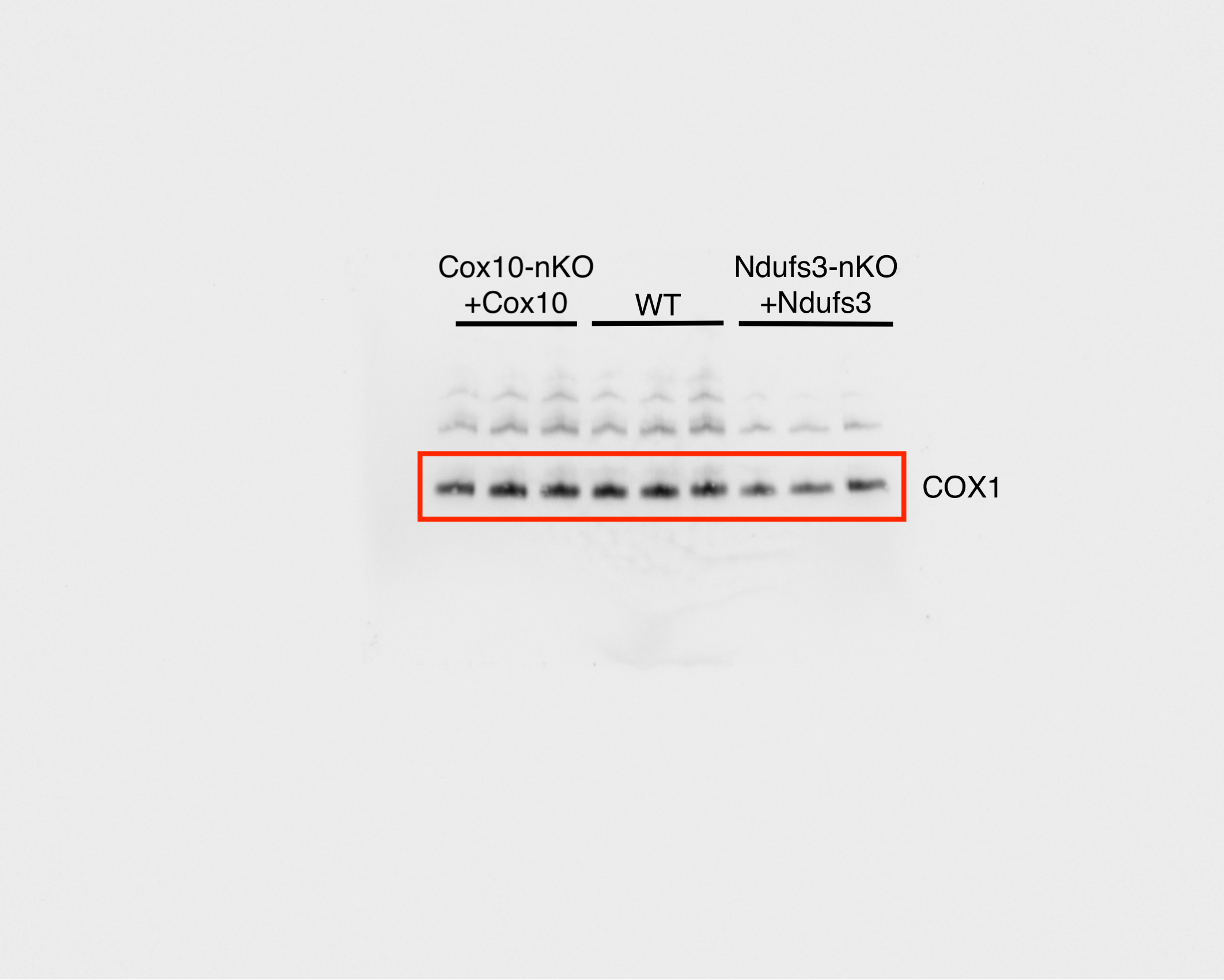

Supplement: Supplementary file 10 — EV and Appendix Figure Source Data [file 44321_2024_111_MOESM10_ESM.zip › Source Data for Expanded View and Appendix/EMM-2024-19843_SourceData-FigureEV3/EV3F/western - COX1.tiff]

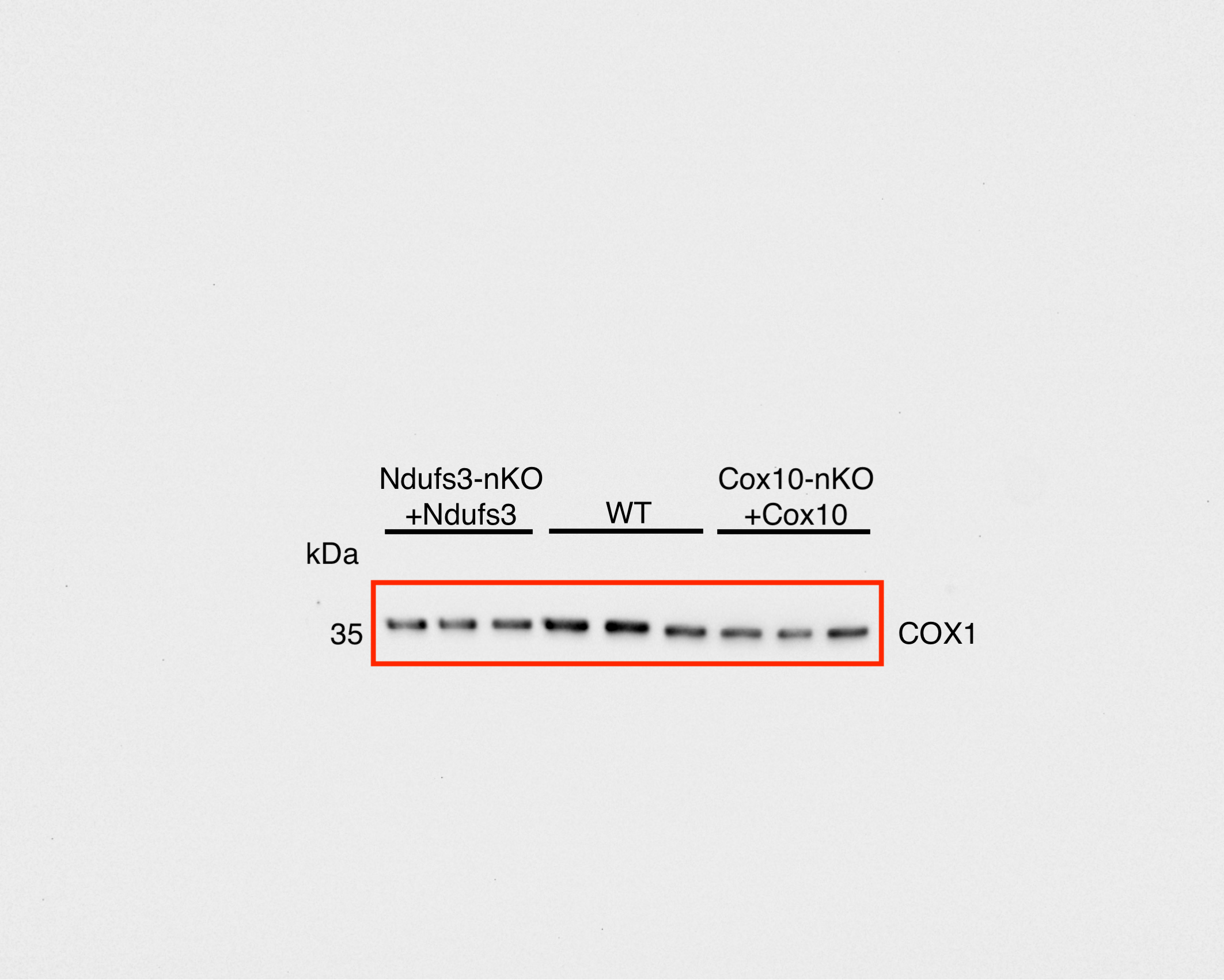

Supplement: Supplementary file 10 — EV and Appendix Figure Source Data [file 44321_2024_111_MOESM10_ESM.zip › Source Data for Expanded View and Appendix/EMM-2024-19843_SourceData-FigureEV3/EV3D/western - COX1 CTX.tiff]

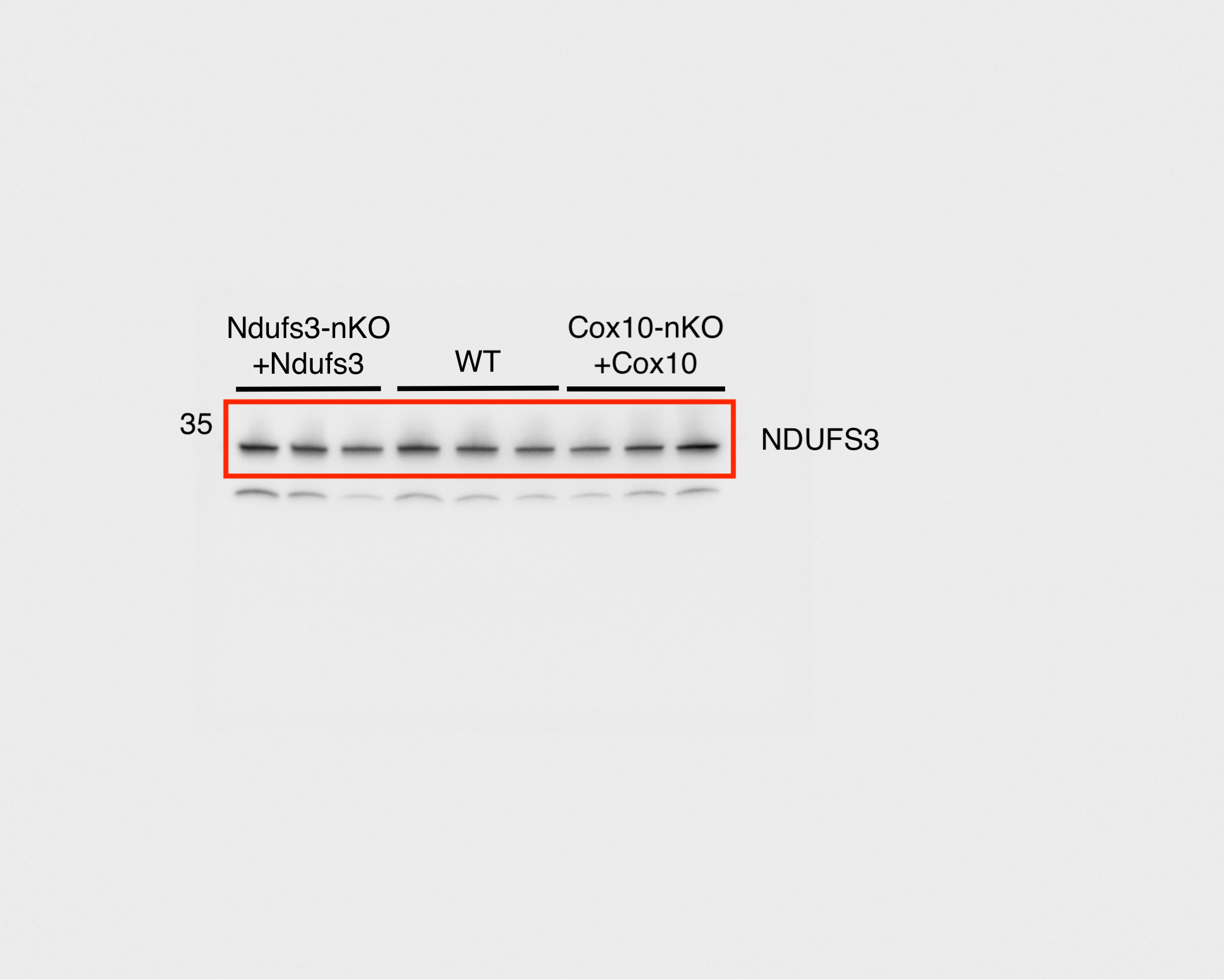

Supplement: Supplementary file 10 — EV and Appendix Figure Source Data [file 44321_2024_111_MOESM10_ESM.zip › Source Data for Expanded View and Appendix/EMM-2024-19843_SourceData-FigureEV3/EV3D/western - NDUFS3 CTX.tiff]

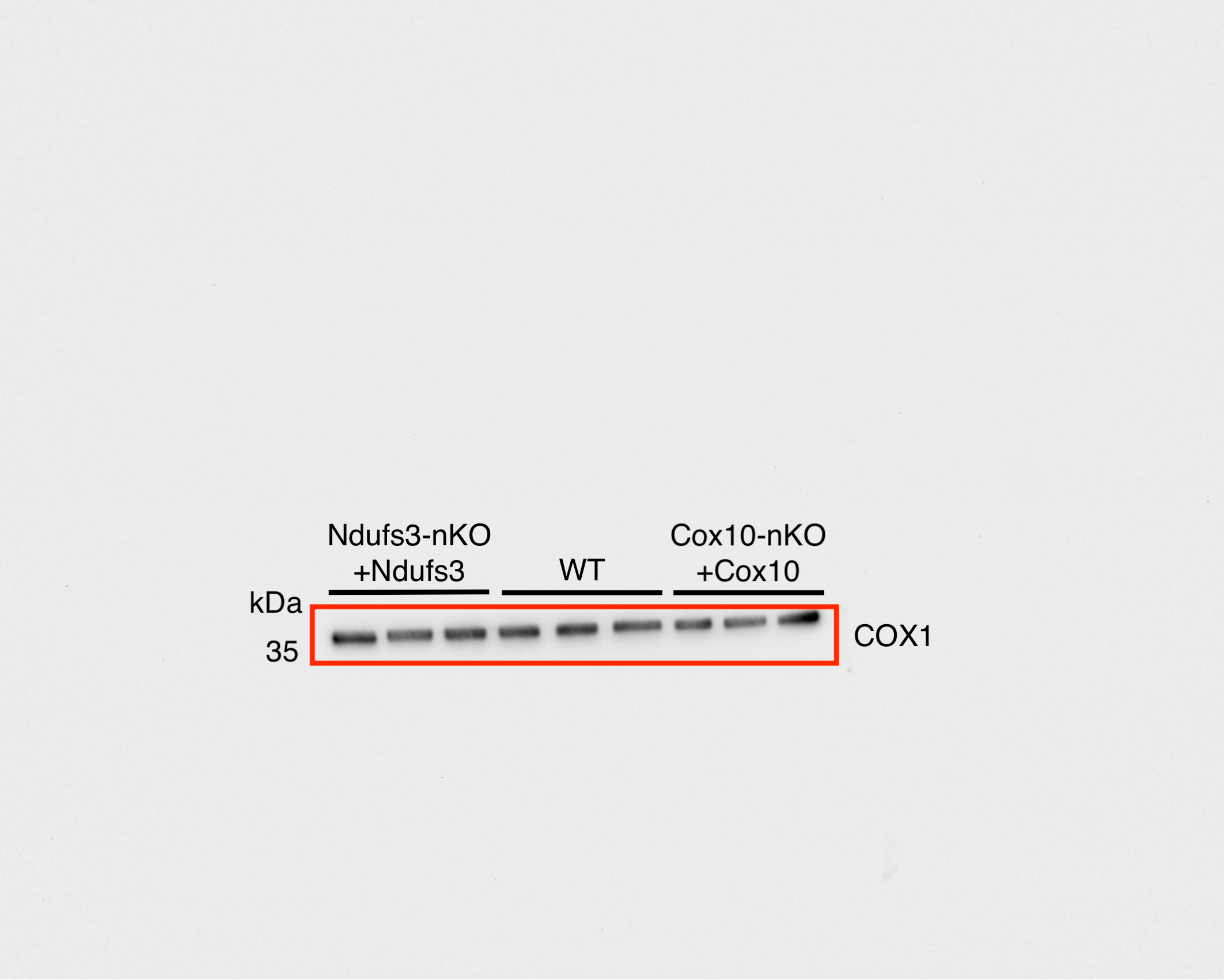

Supplement: Supplementary file 10 — EV and Appendix Figure Source Data [file 44321_2024_111_MOESM10_ESM.zip › Source Data for Expanded View and Appendix/EMM-2024-19843_SourceData-FigureEV3/EV3D/western - COX1 HIPP.tiff]

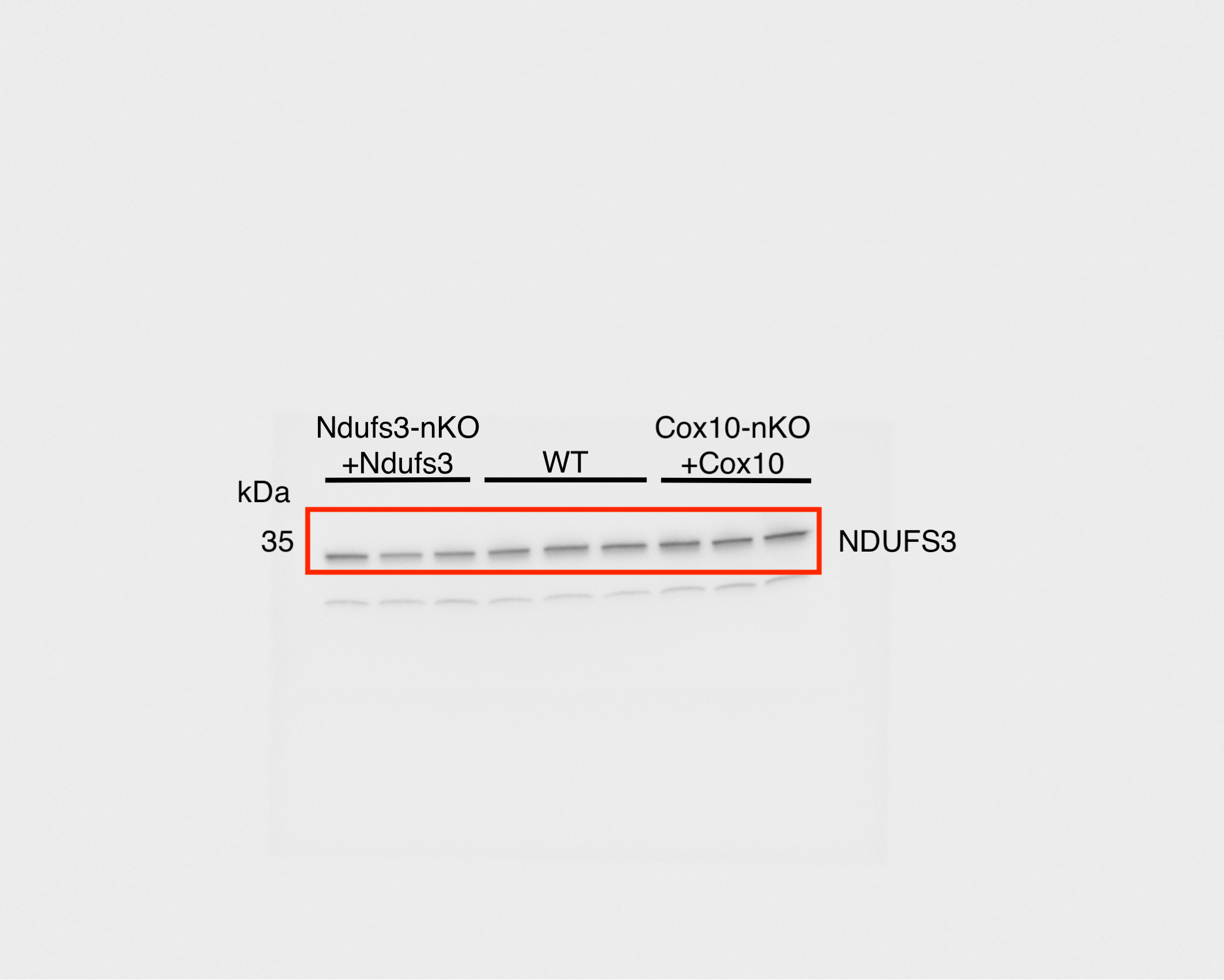

Supplement: Supplementary file 10 — EV and Appendix Figure Source Data [file 44321_2024_111_MOESM10_ESM.zip › Source Data for Expanded View and Appendix/EMM-2024-19843_SourceData-FigureEV3/EV3D/western - NDUFS3 HPP.tiff]

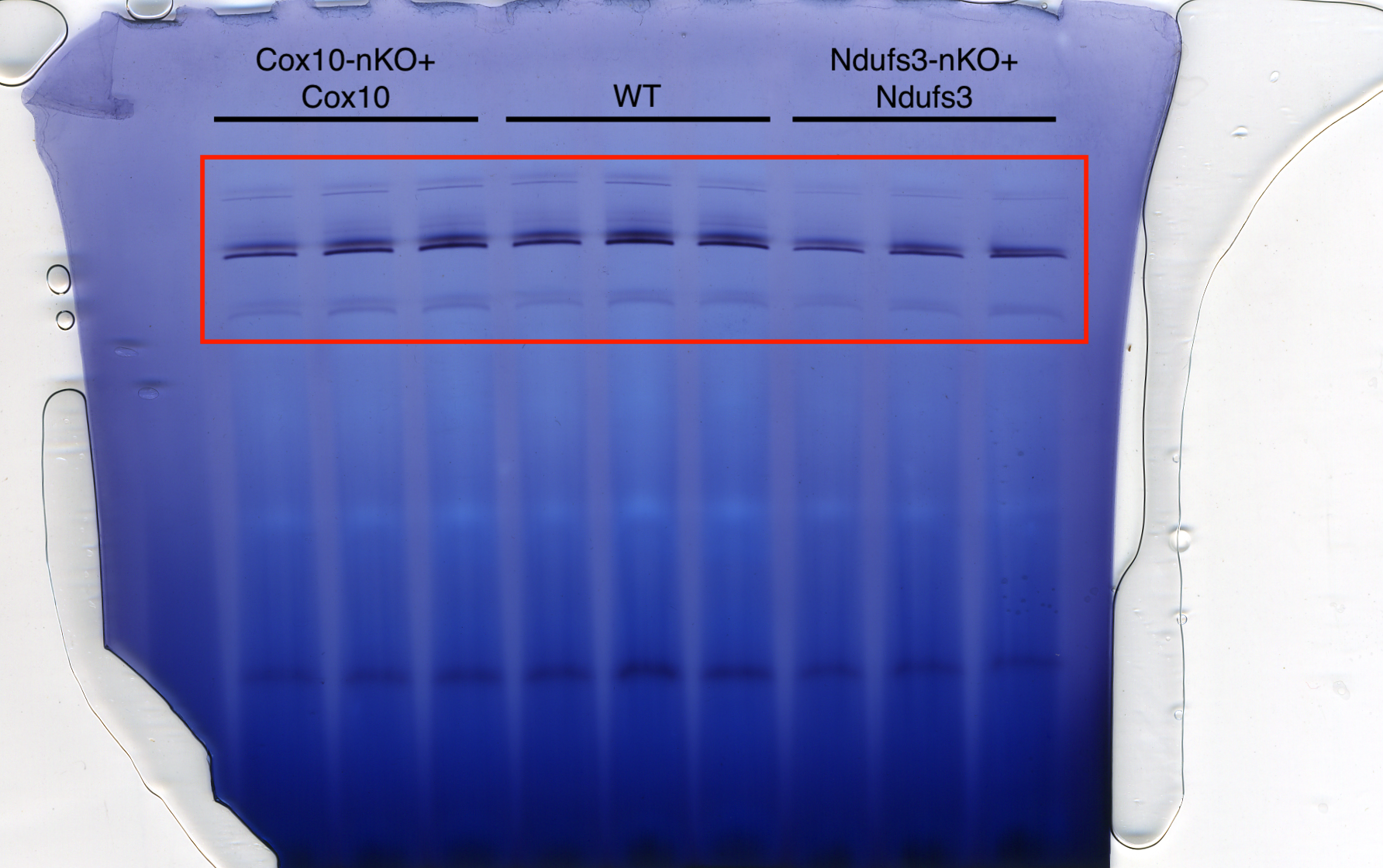

Supplement: Supplementary file 10 — EV and Appendix Figure Source Data [file 44321_2024_111_MOESM10_ESM.zip › Source Data for Expanded View and Appendix/EMM-2024-19843_SourceData-FigureEV3/EV3H/IGA - Complex I.tiff]

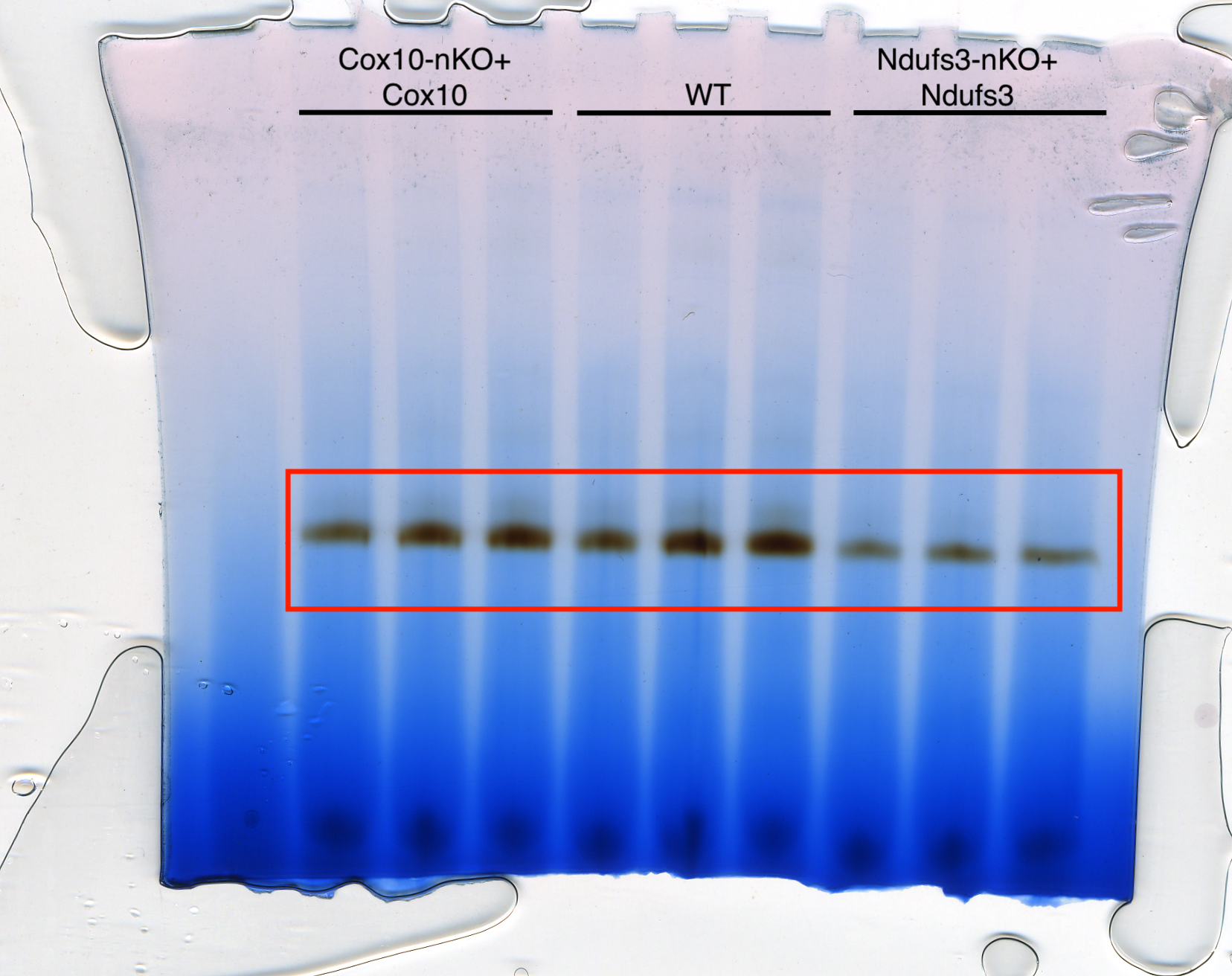

Supplement: Supplementary file 10 — EV and Appendix Figure Source Data [file 44321_2024_111_MOESM10_ESM.zip › Source Data for Expanded View and Appendix/EMM-2024-19843_SourceData-FigureEV3/EV3H/IGA - Complex IV.tiff]

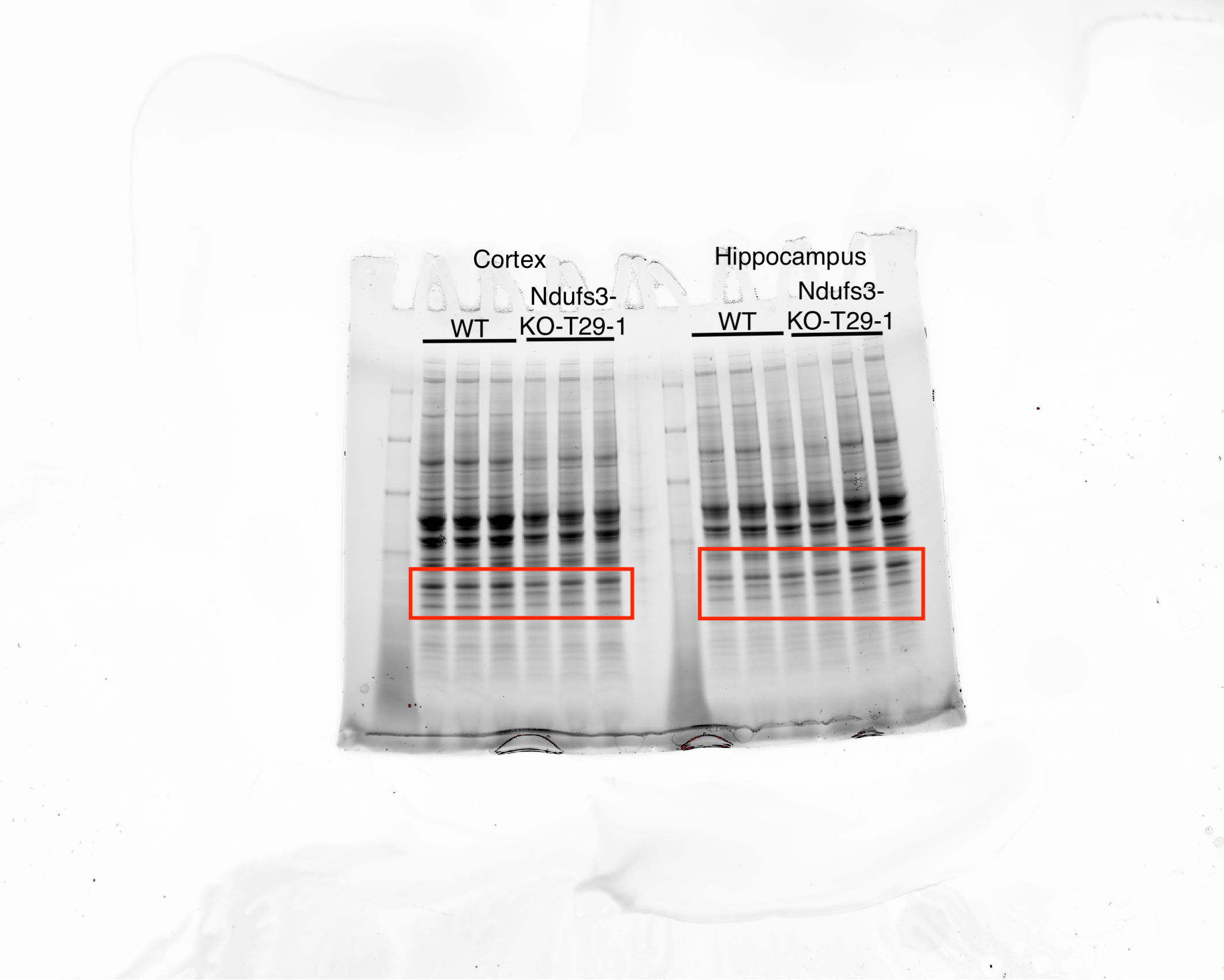

Supplement: Supplementary file 10 — EV and Appendix Figure Source Data [file 44321_2024_111_MOESM10_ESM.zip › Source Data for Expanded View and Appendix/EMM-2024-19843_SourceData-FigureEV5/EV5F,J/western - Total Protein.tiff]

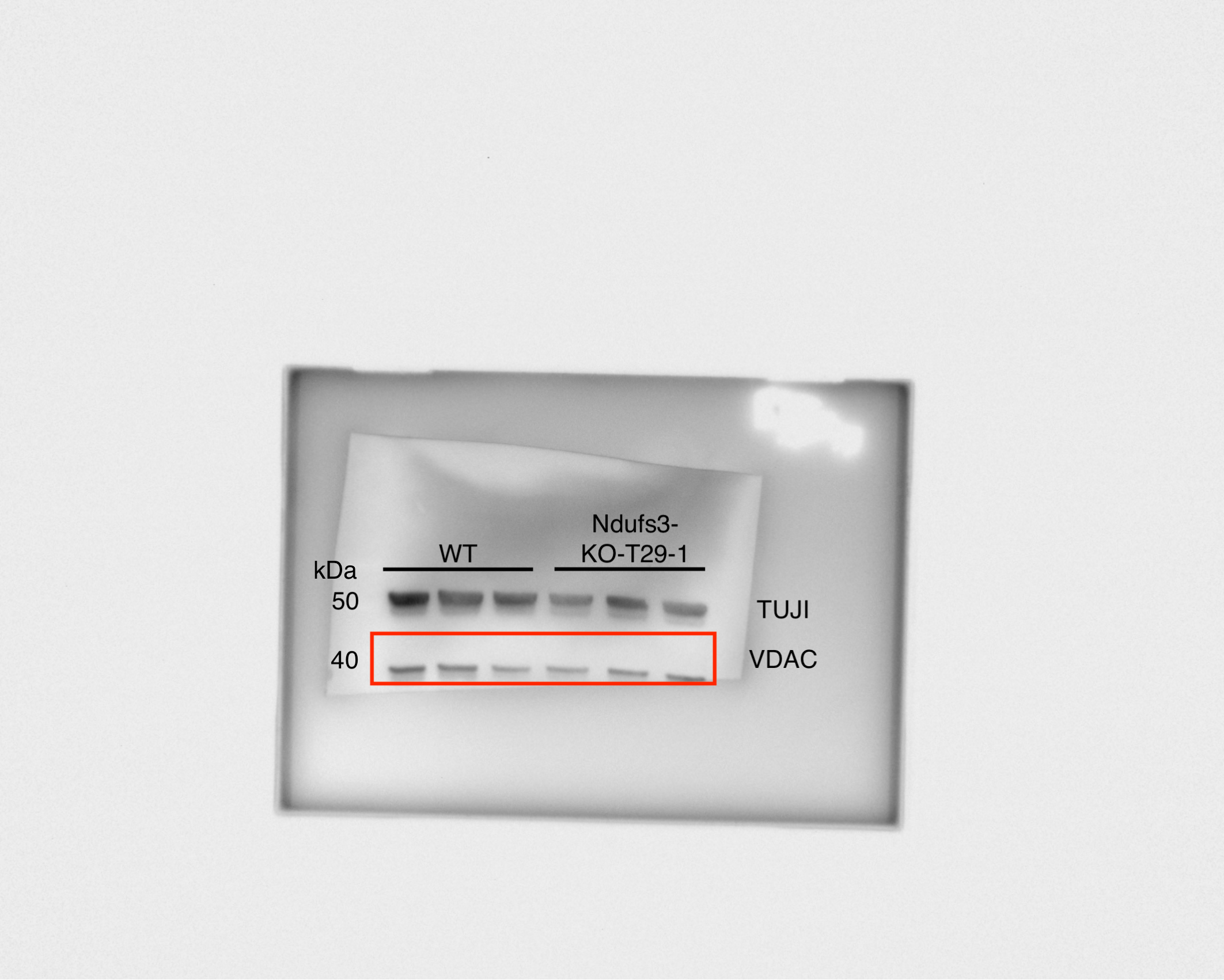

Supplement: Supplementary file 10 — EV and Appendix Figure Source Data [file 44321_2024_111_MOESM10_ESM.zip › Source Data for Expanded View and Appendix/EMM-2024-19843_SourceData-FigureEV5/EV5F,J/western - VDAC HPP.tiff]

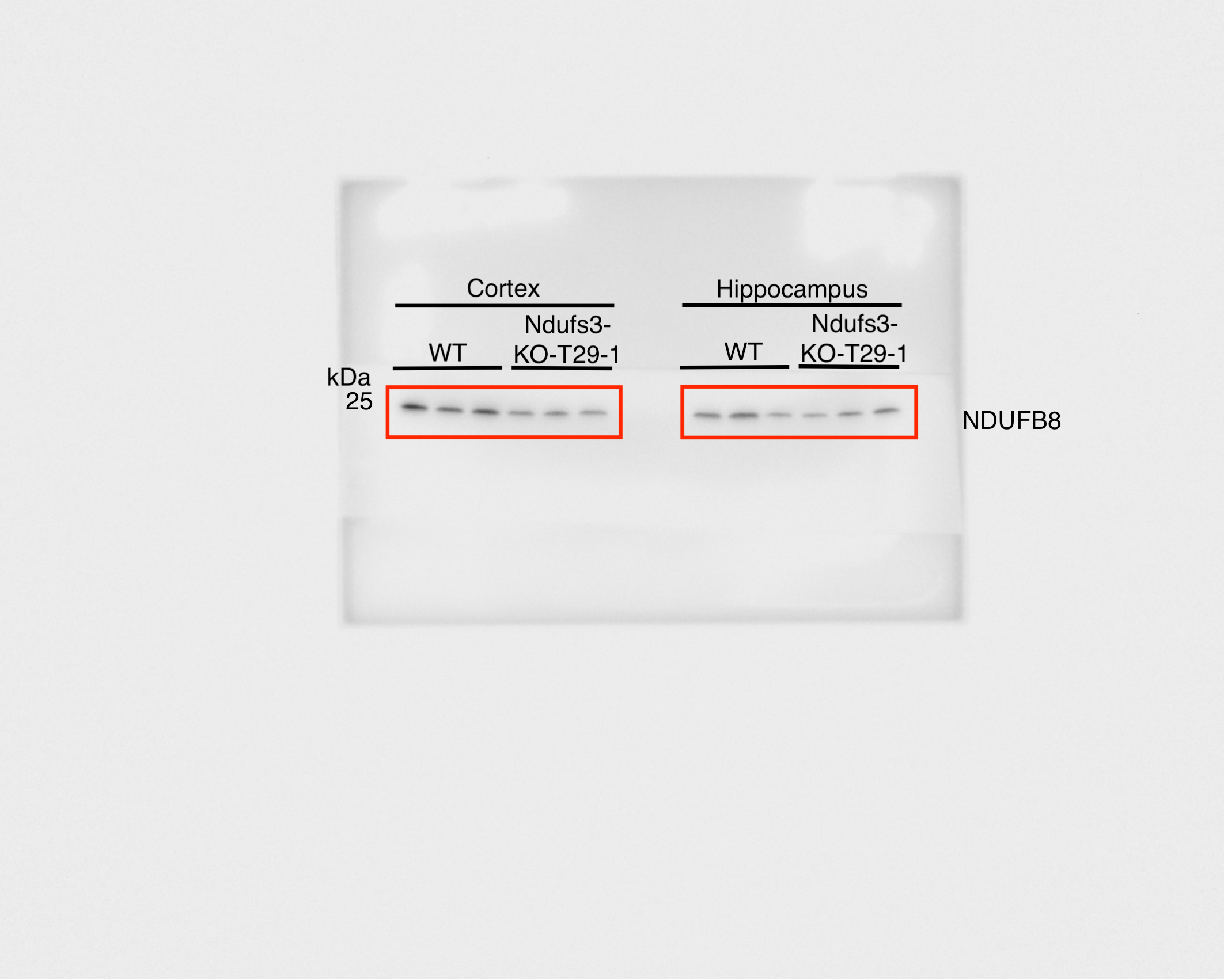

Supplement: Supplementary file 10 — EV and Appendix Figure Source Data [file 44321_2024_111_MOESM10_ESM.zip › Source Data for Expanded View and Appendix/EMM-2024-19843_SourceData-FigureEV5/EV5F,J/western - NDUFB8.tiff]

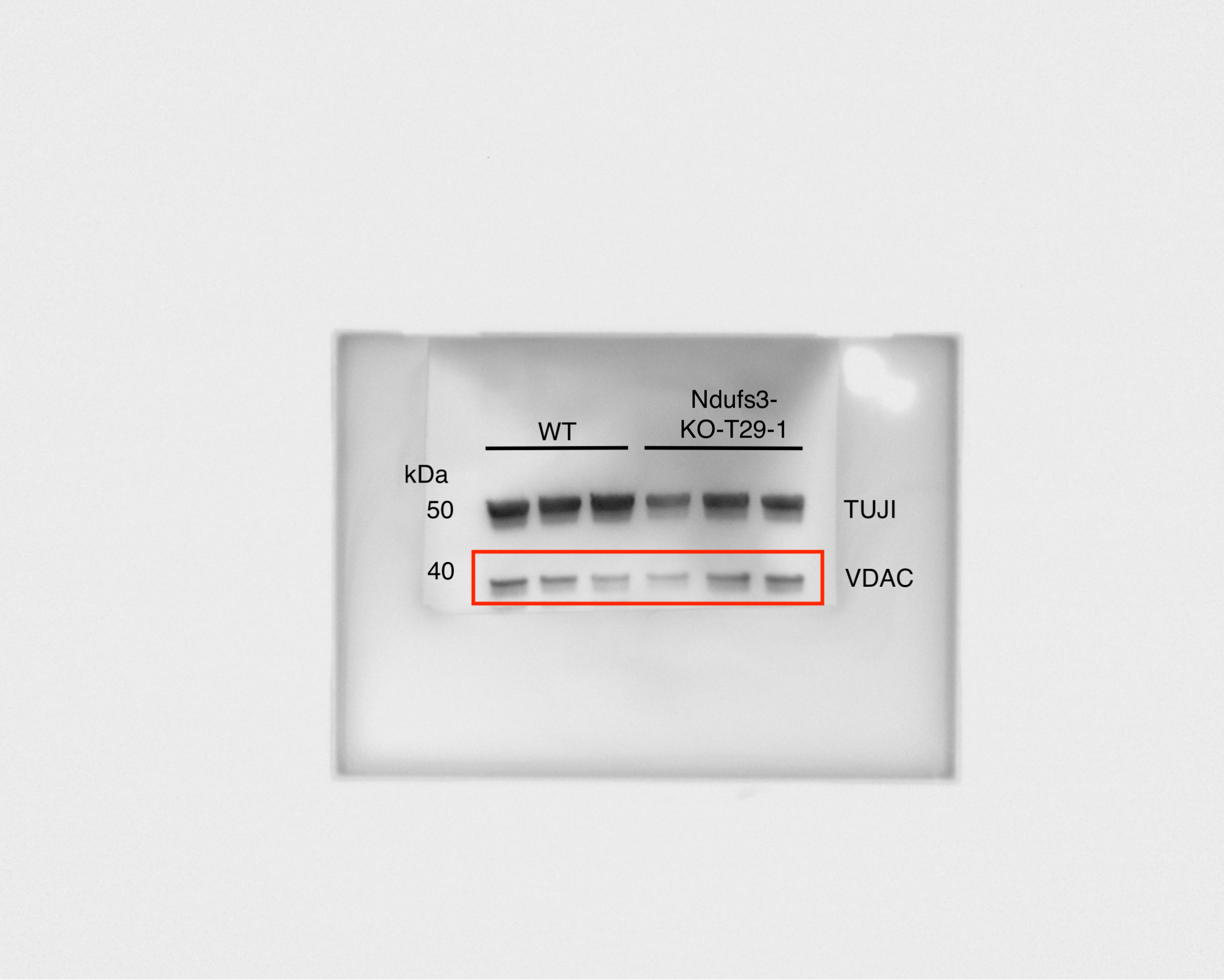

Supplement: Supplementary file 10 — EV and Appendix Figure Source Data [file 44321_2024_111_MOESM10_ESM.zip › Source Data for Expanded View and Appendix/EMM-2024-19843_SourceData-FigureEV5/EV5F,J/western - VDAC CTX.tiff]

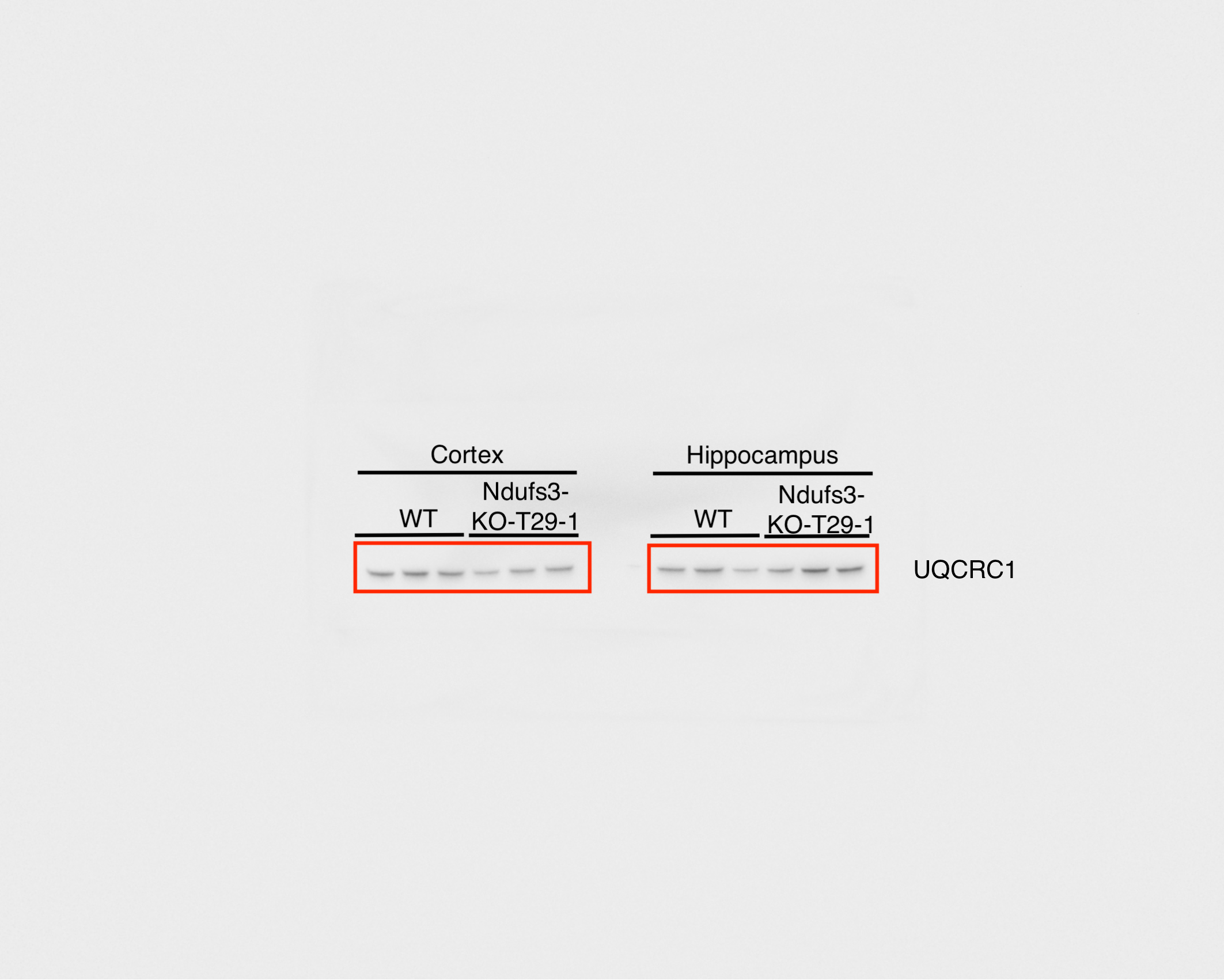

Supplement: Supplementary file 10 — EV and Appendix Figure Source Data [file 44321_2024_111_MOESM10_ESM.zip › Source Data for Expanded View and Appendix/EMM-2024-19843_SourceData-FigureEV5/EV5F,J/western - UQCRC1.tiff]

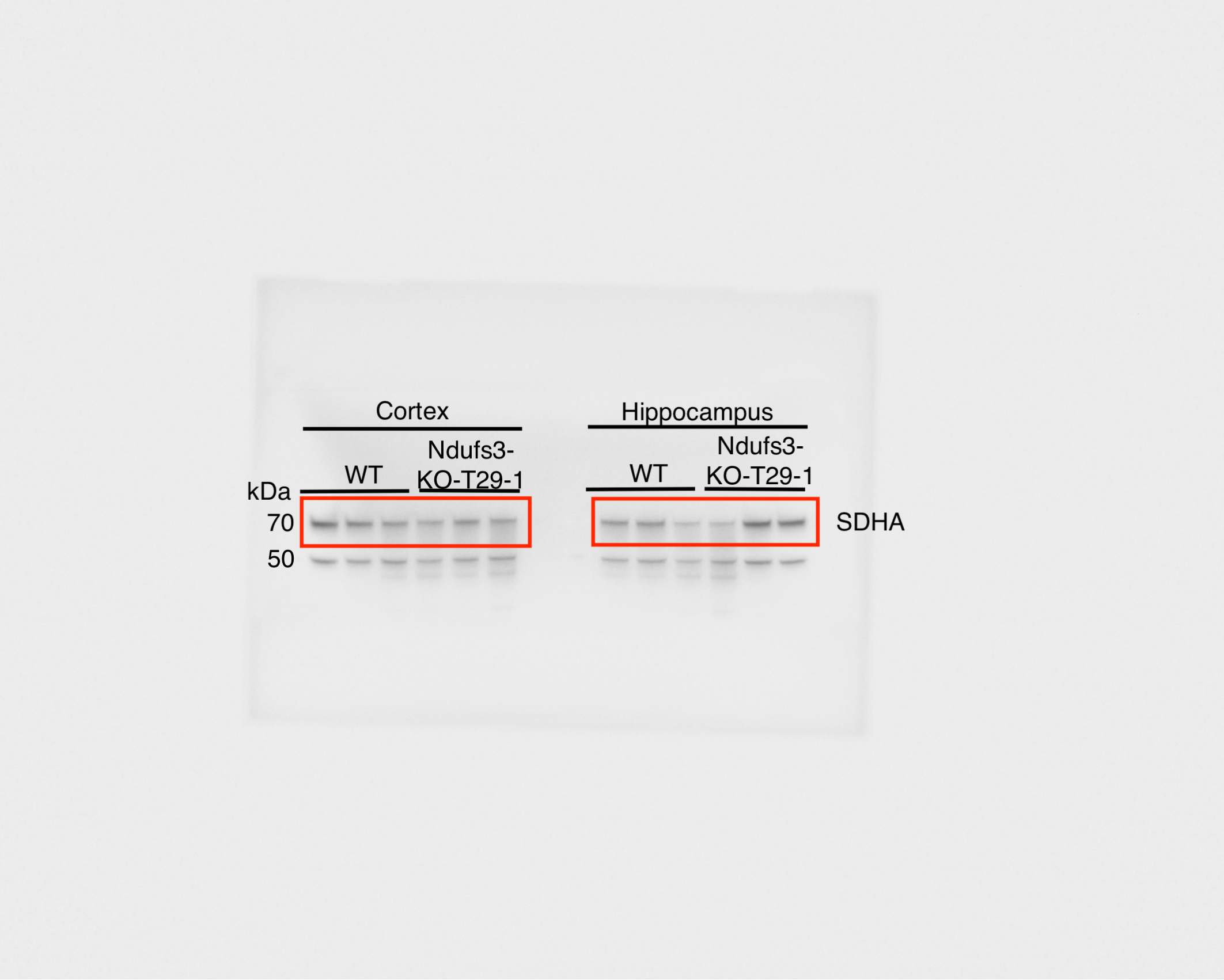

Supplement: Supplementary file 10 — EV and Appendix Figure Source Data [file 44321_2024_111_MOESM10_ESM.zip › Source Data for Expanded View and Appendix/EMM-2024-19843_SourceData-FigureEV5/EV5F,J/western - SDHA.tiff]

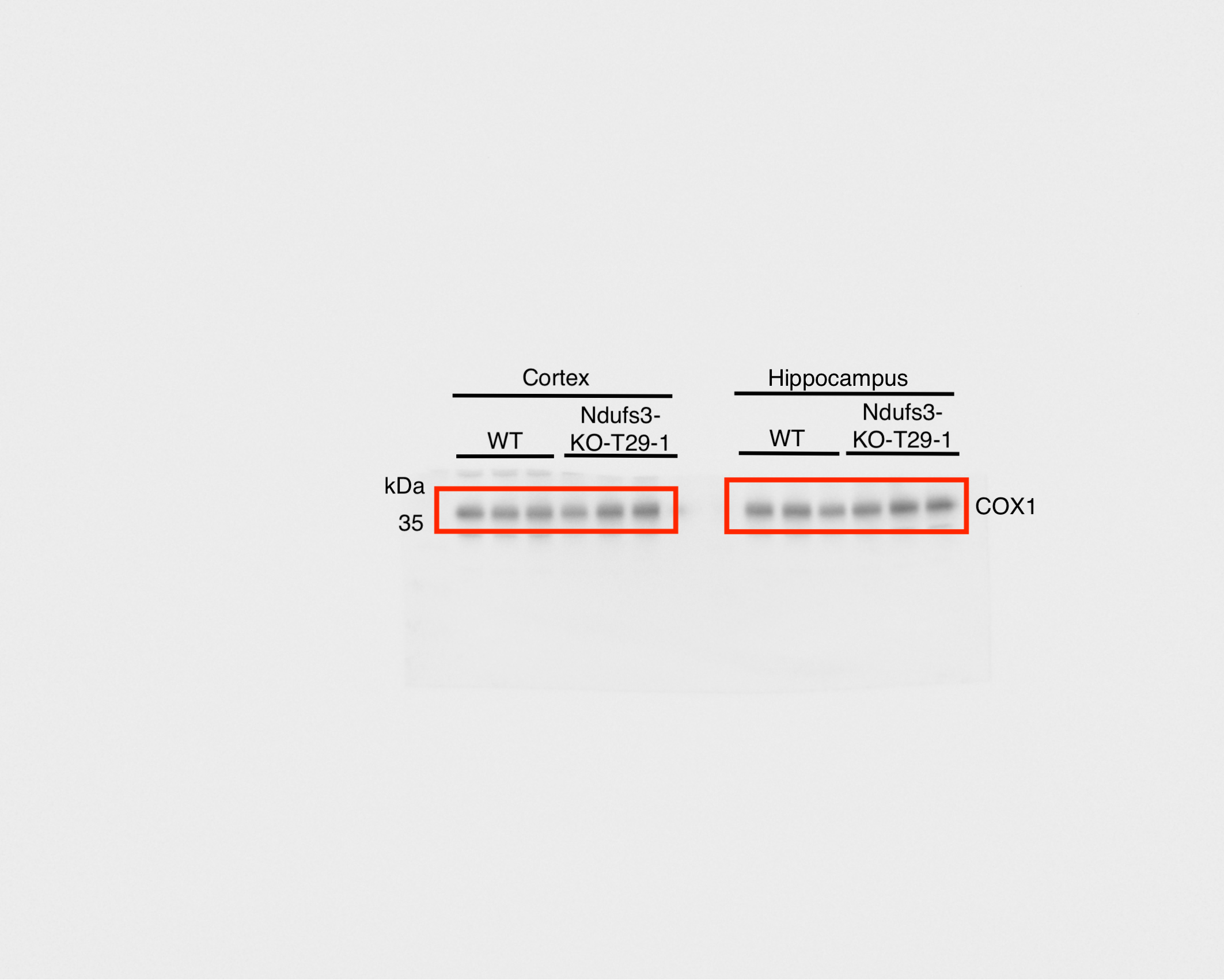

Supplement: Supplementary file 10 — EV and Appendix Figure Source Data [file 44321_2024_111_MOESM10_ESM.zip › Source Data for Expanded View and Appendix/EMM-2024-19843_SourceData-FigureEV5/EV5F,J/western - COX1.tiff]

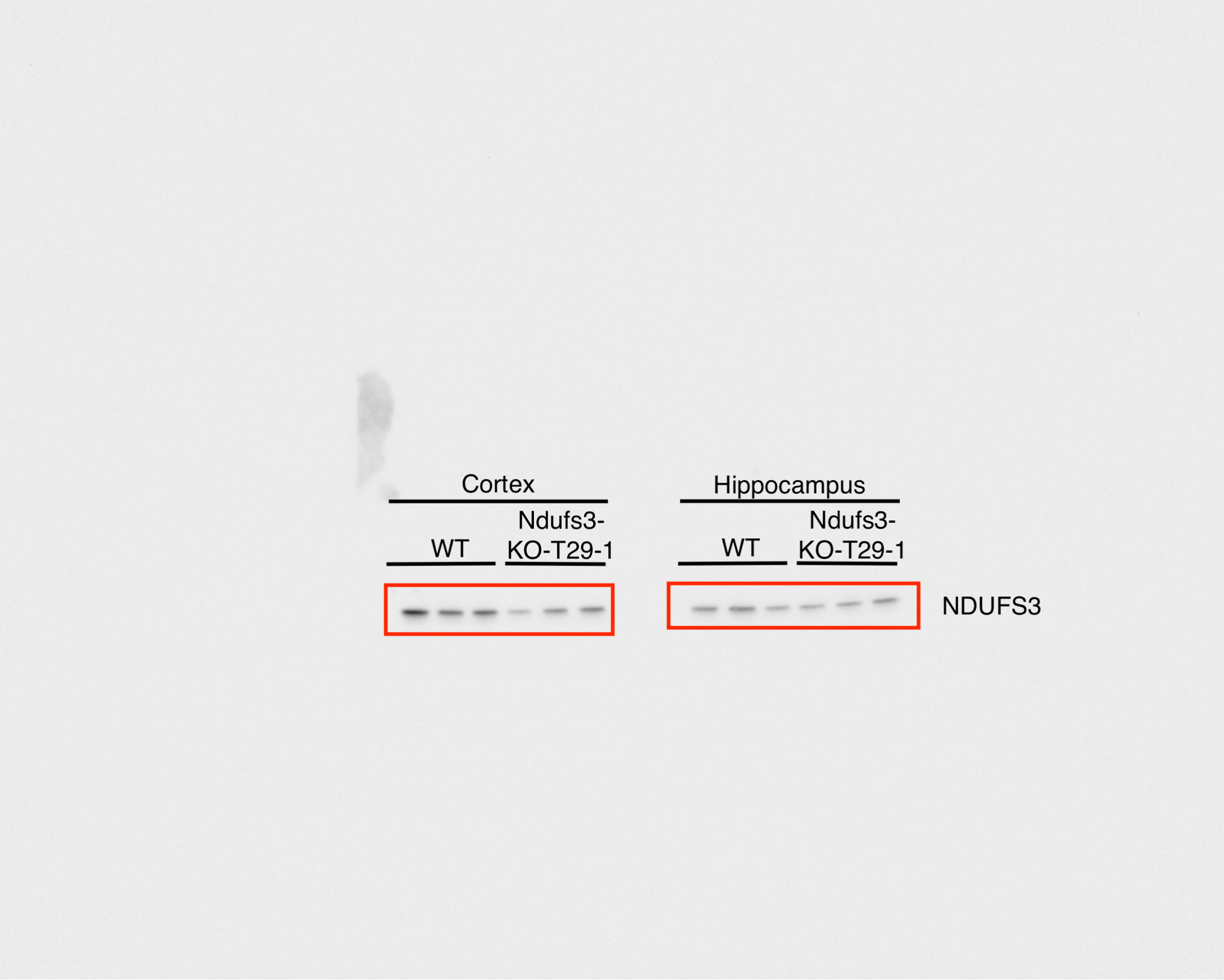

Supplement: Supplementary file 10 — EV and Appendix Figure Source Data [file 44321_2024_111_MOESM10_ESM.zip › Source Data for Expanded View and Appendix/EMM-2024-19843_SourceData-FigureEV5/EV5F,J/western - NDUFS3.tiff]

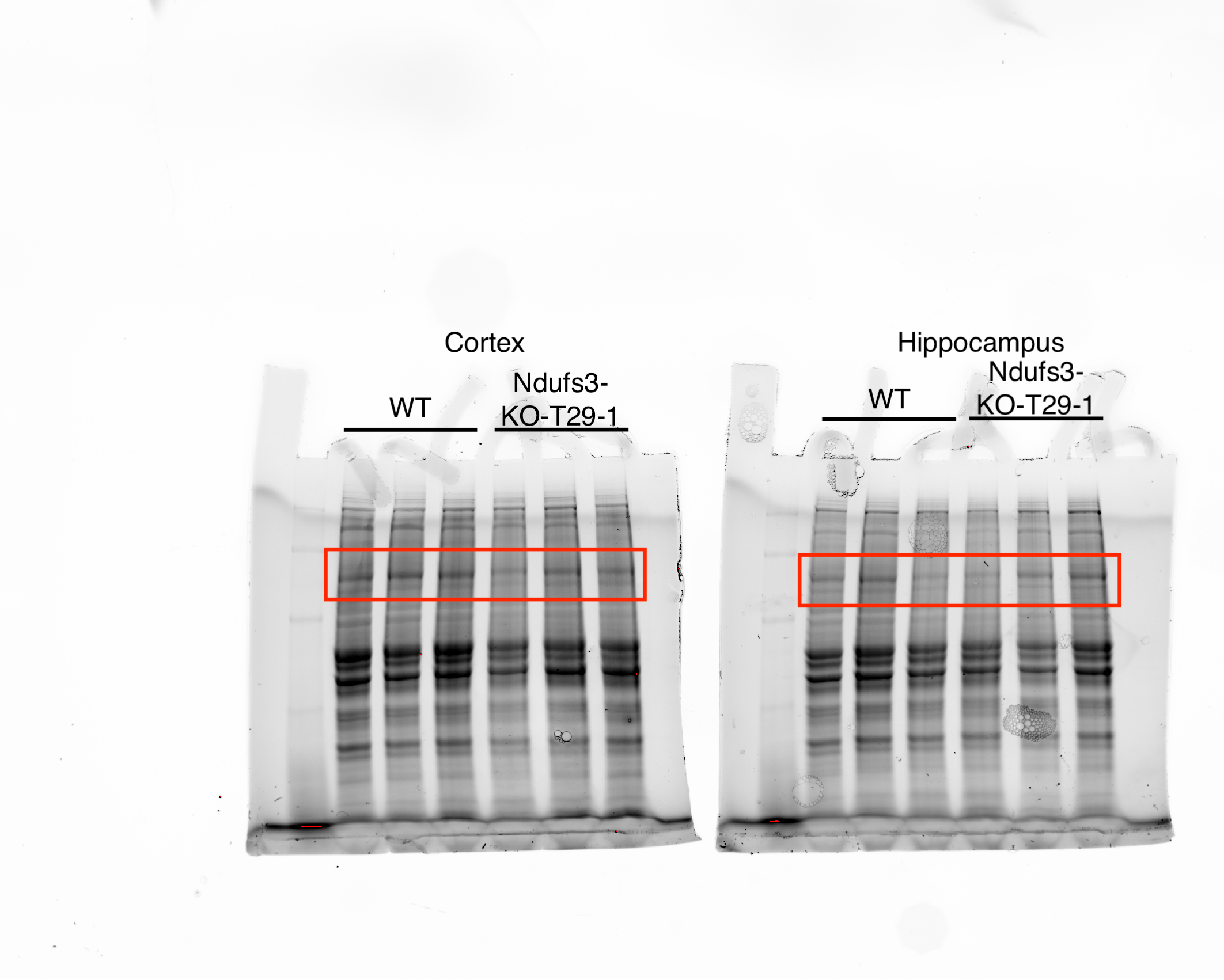

Supplement: Supplementary file 10 — EV and Appendix Figure Source Data [file 44321_2024_111_MOESM10_ESM.zip › Source Data for Expanded View and Appendix/EMM-2024-19843_SourceData-FigureEV5/EV5N/western - Total Protein.tiff]

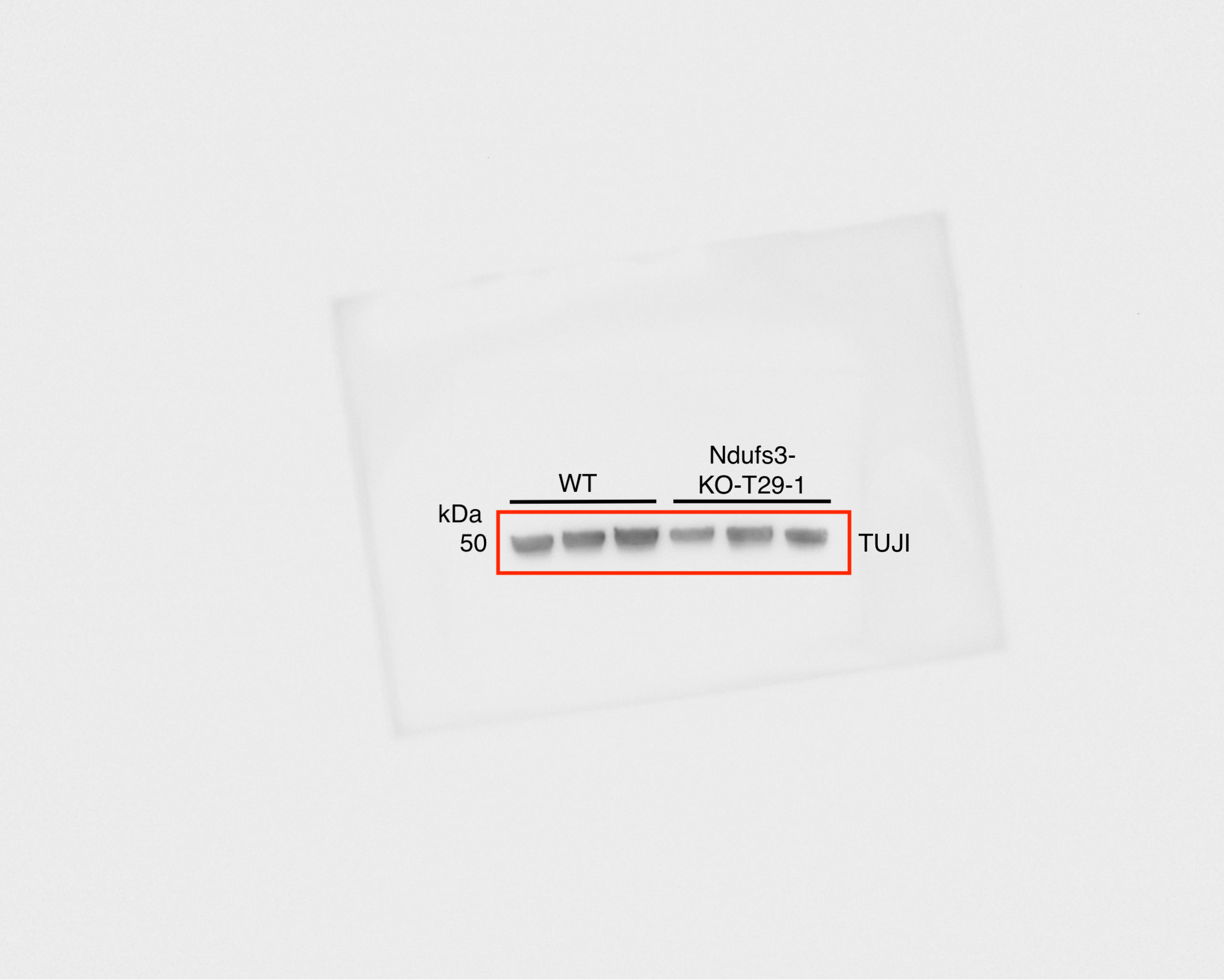

Supplement: Supplementary file 10 — EV and Appendix Figure Source Data [file 44321_2024_111_MOESM10_ESM.zip › Source Data for Expanded View and Appendix/EMM-2024-19843_SourceData-FigureEV5/EV5N/western - TUJI CTX.tiff]

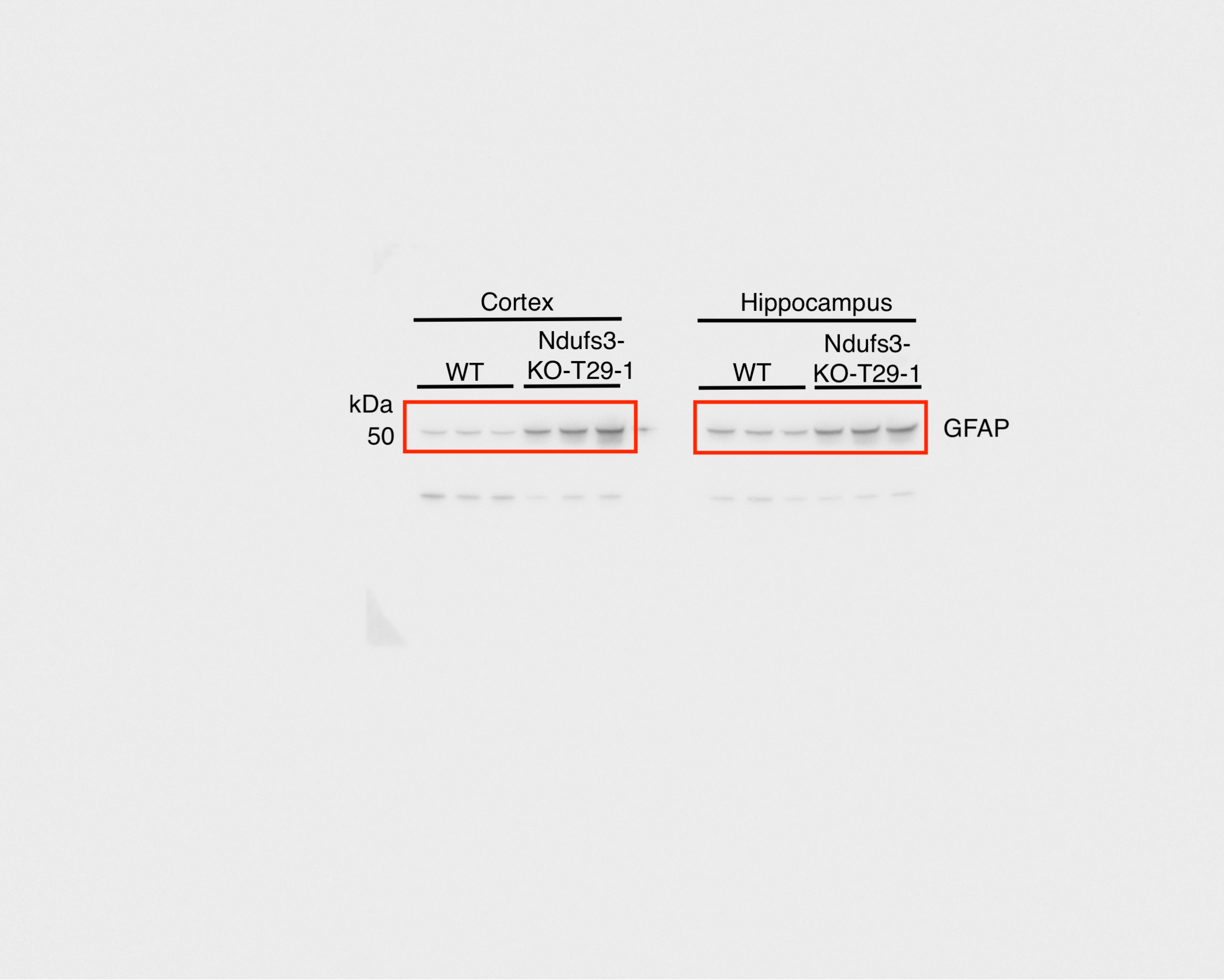

Supplement: Supplementary file 10 — EV and Appendix Figure Source Data [file 44321_2024_111_MOESM10_ESM.zip › Source Data for Expanded View and Appendix/EMM-2024-19843_SourceData-FigureEV5/EV5N/western GFAP.tiff]

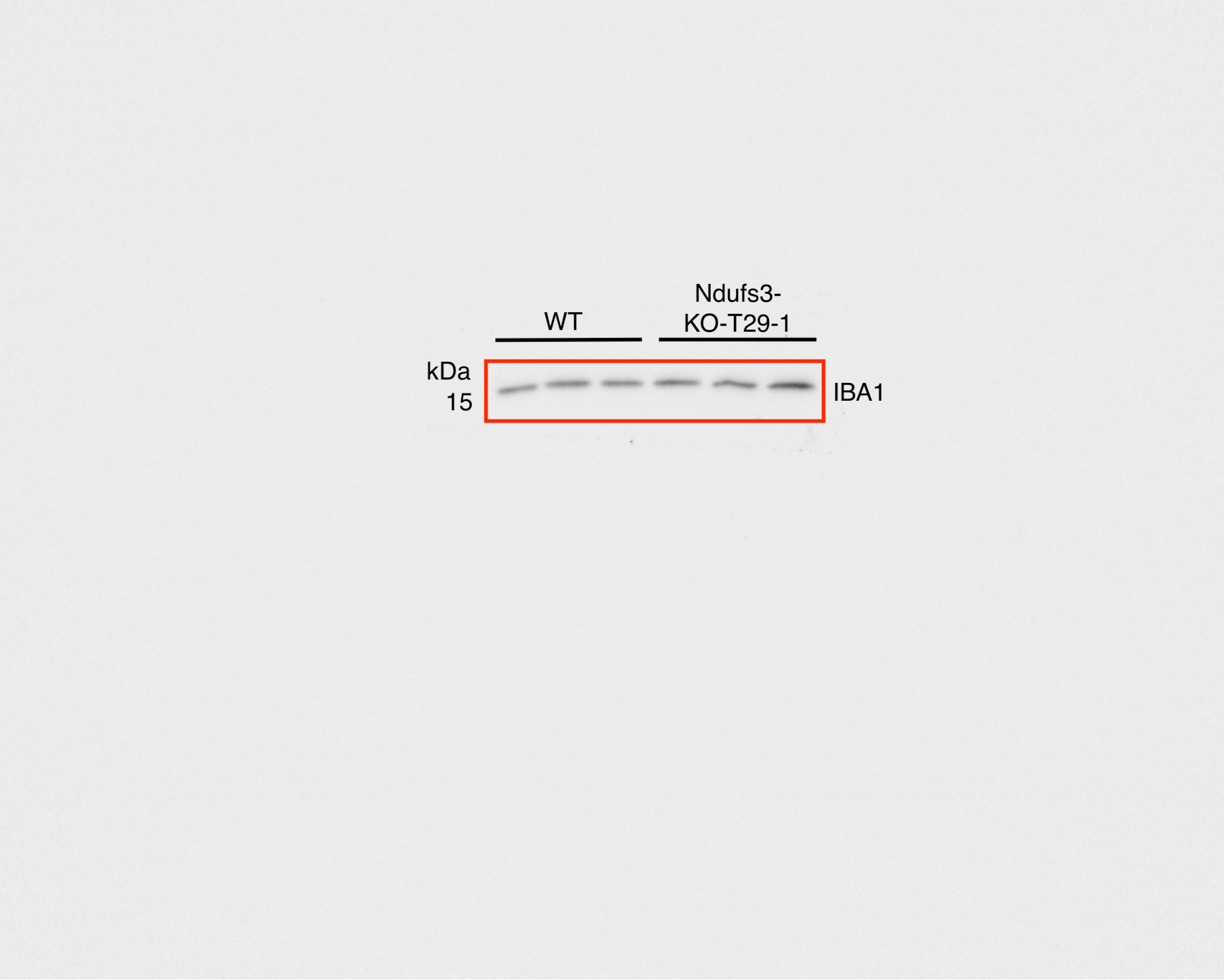

Supplement: Supplementary file 10 — EV and Appendix Figure Source Data [file 44321_2024_111_MOESM10_ESM.zip › Source Data for Expanded View and Appendix/EMM-2024-19843_SourceData-FigureEV5/EV5N/western - IBA1 HPP.tiff]

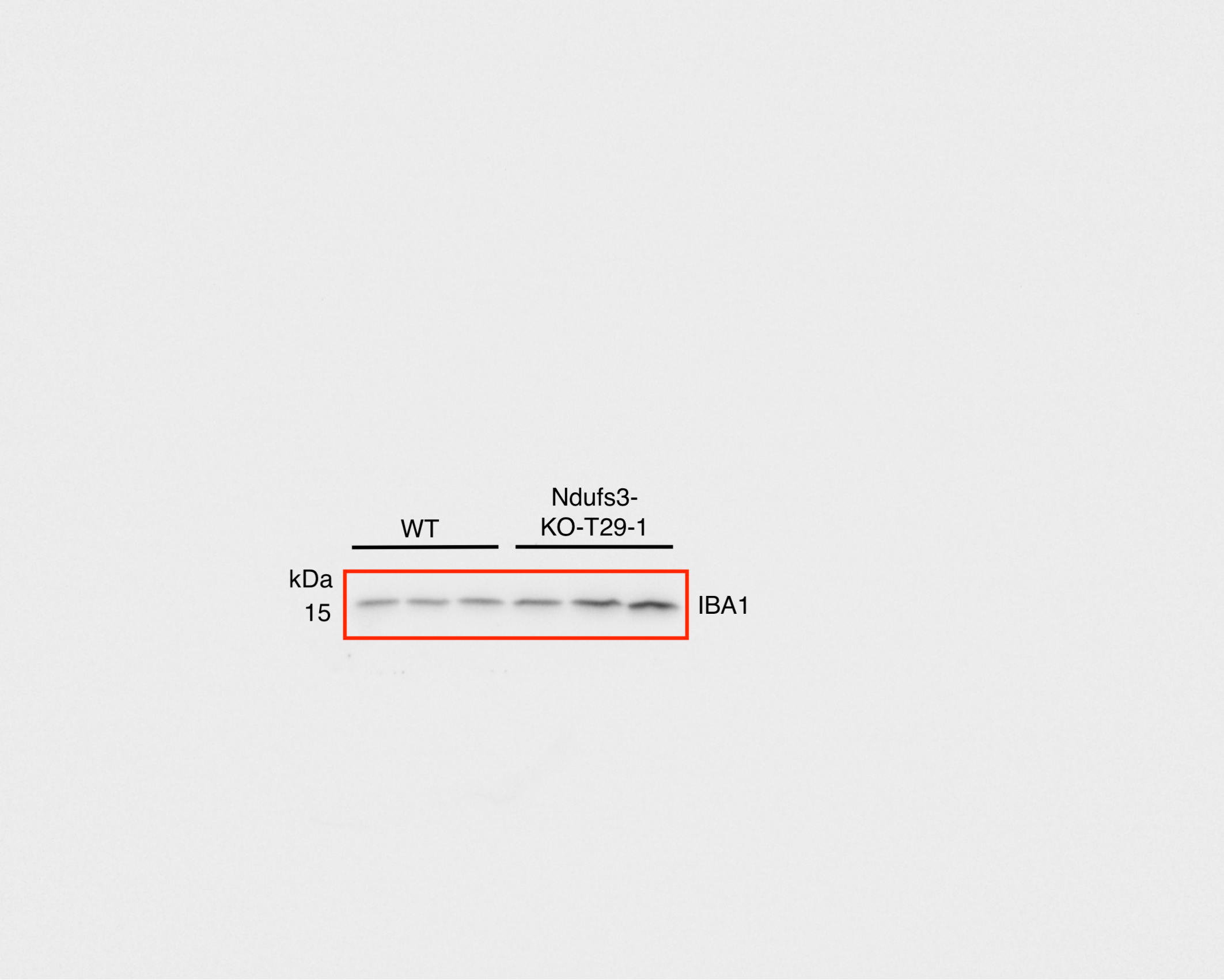

Supplement: Supplementary file 10 — EV and Appendix Figure Source Data [file 44321_2024_111_MOESM10_ESM.zip › Source Data for Expanded View and Appendix/EMM-2024-19843_SourceData-FigureEV5/EV5N/western - IBA1 CTX.tiff]

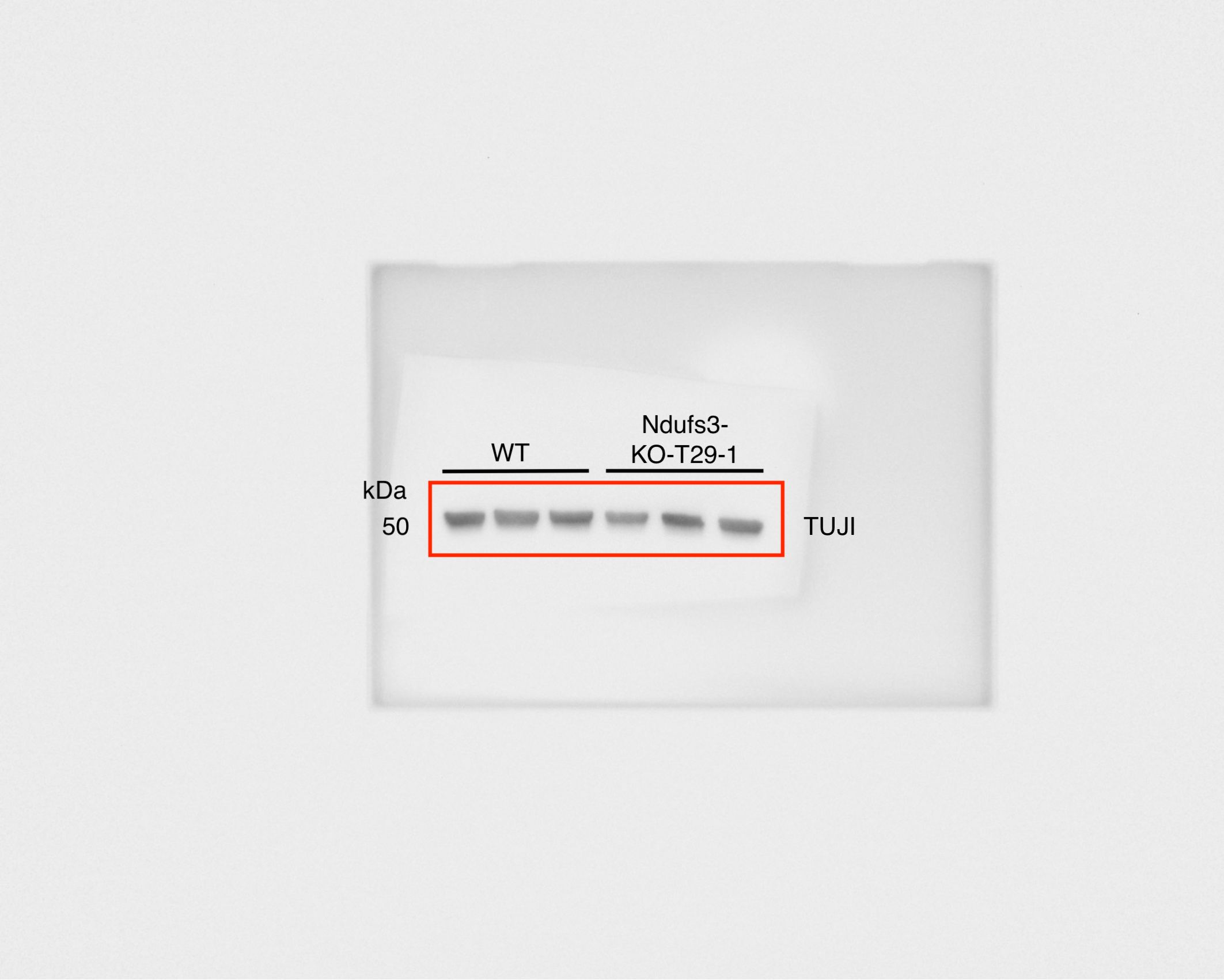

Supplement: Supplementary file 10 — EV and Appendix Figure Source Data [file 44321_2024_111_MOESM10_ESM.zip › Source Data for Expanded View and Appendix/EMM-2024-19843_SourceData-FigureEV5/EV5N/western - TUJI HPP.tiff]

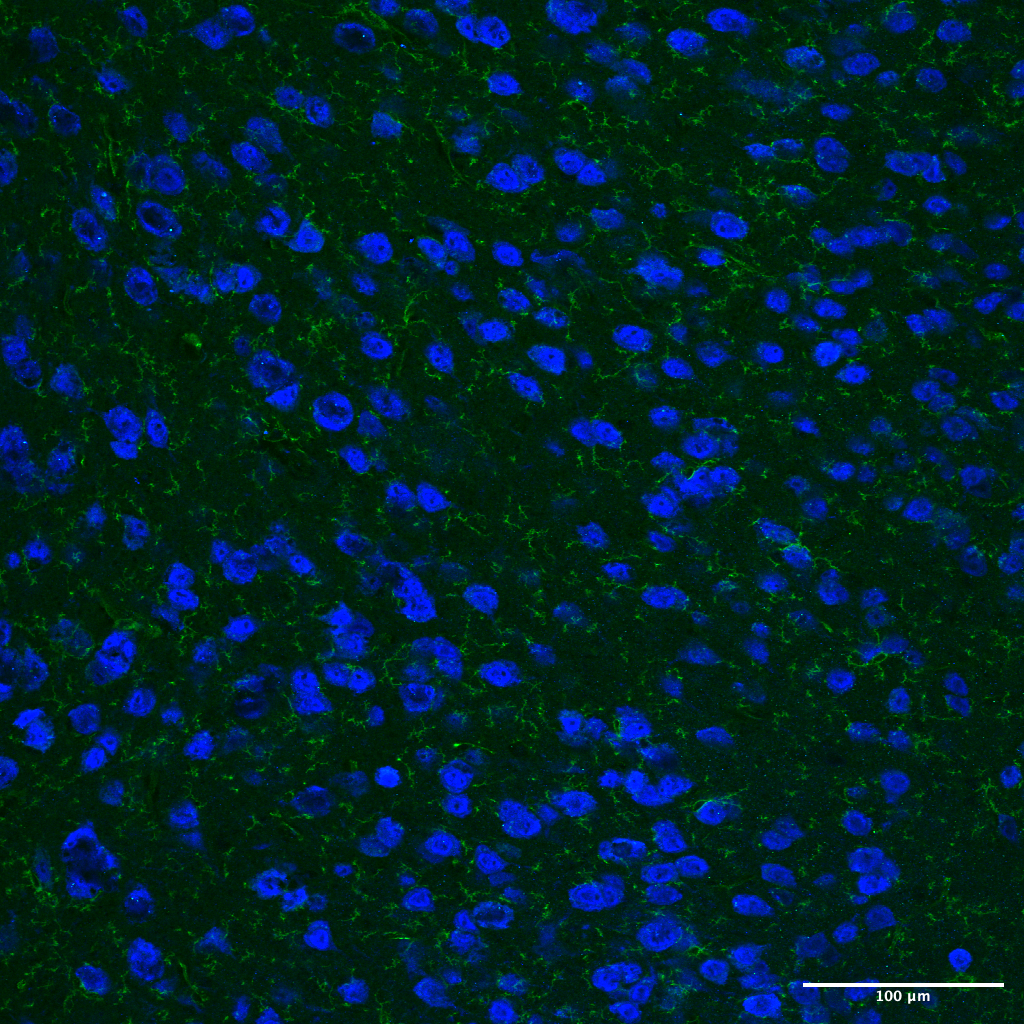

Supplement: Supplementary file 10 — EV and Appendix Figure Source Data [file 44321_2024_111_MOESM10_ESM.zip › Source Data for Expanded View and Appendix/Appendix/EMM-2024-19843_SourceData-FigureS4/S4A/MERGE IHC - saline.tiff]

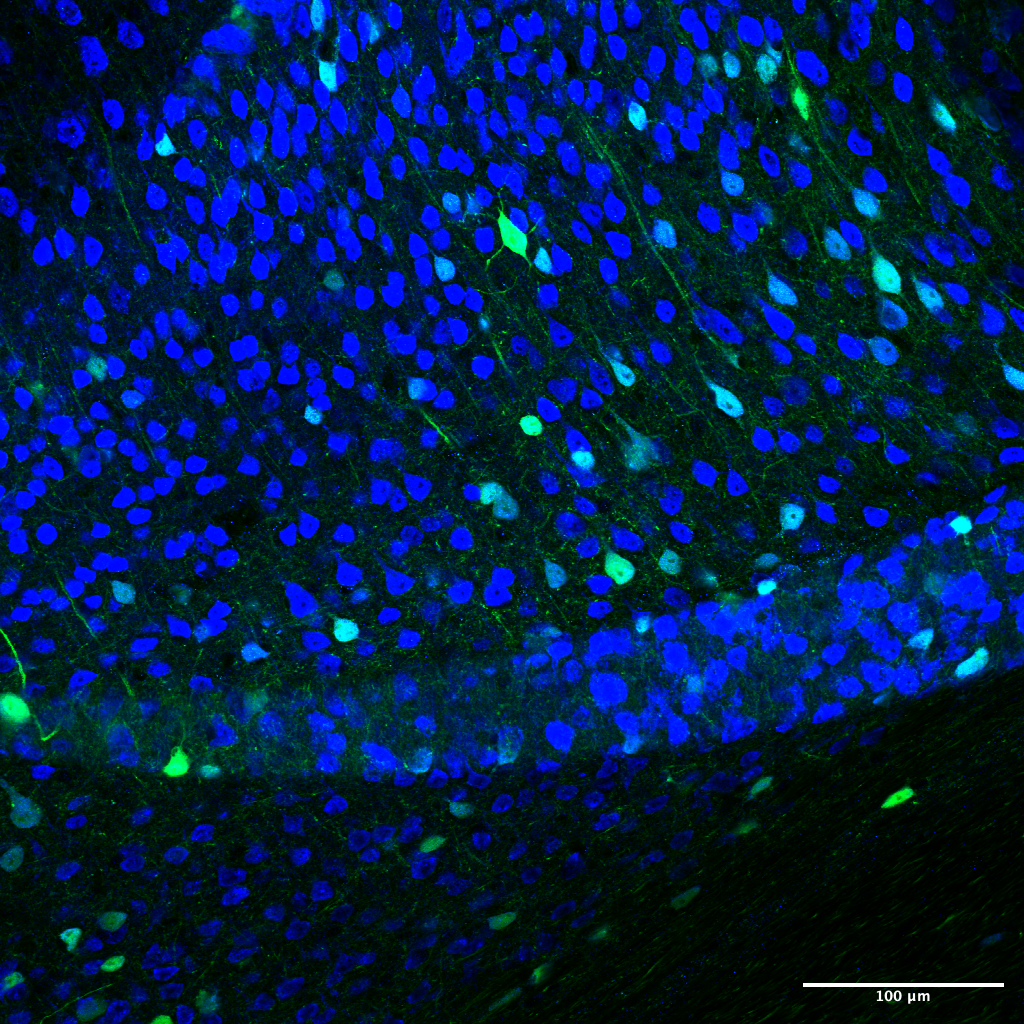

Supplement: Supplementary file 10 — EV and Appendix Figure Source Data [file 44321_2024_111_MOESM10_ESM.zip › Source Data for Expanded View and Appendix/Appendix/EMM-2024-19843_SourceData-FigureS4/S4A/MERGE IHC - 1.5x1011.tiff]

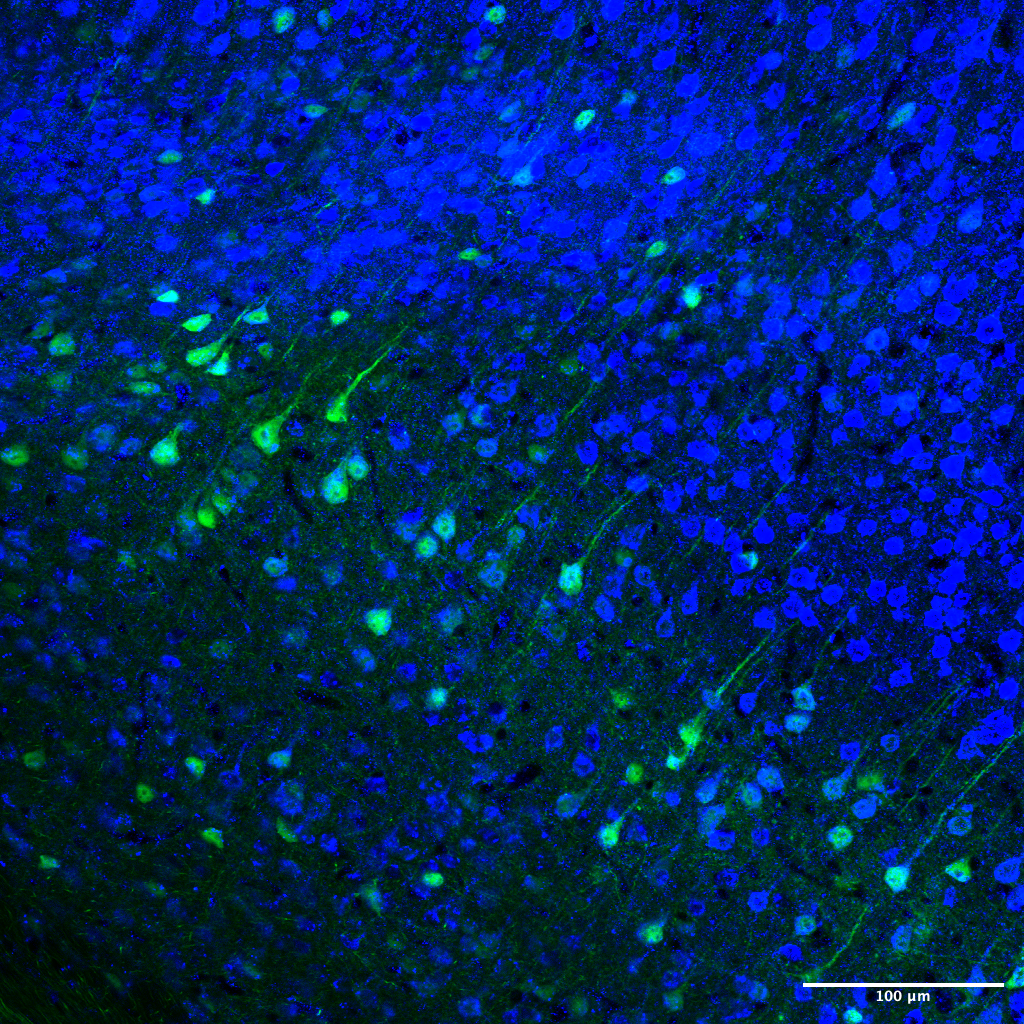

Supplement: Supplementary file 10 — EV and Appendix Figure Source Data [file 44321_2024_111_MOESM10_ESM.zip › Source Data for Expanded View and Appendix/Appendix/EMM-2024-19843_SourceData-FigureS4/S4A/MERGE IHC - 7.5x1012.tiff]

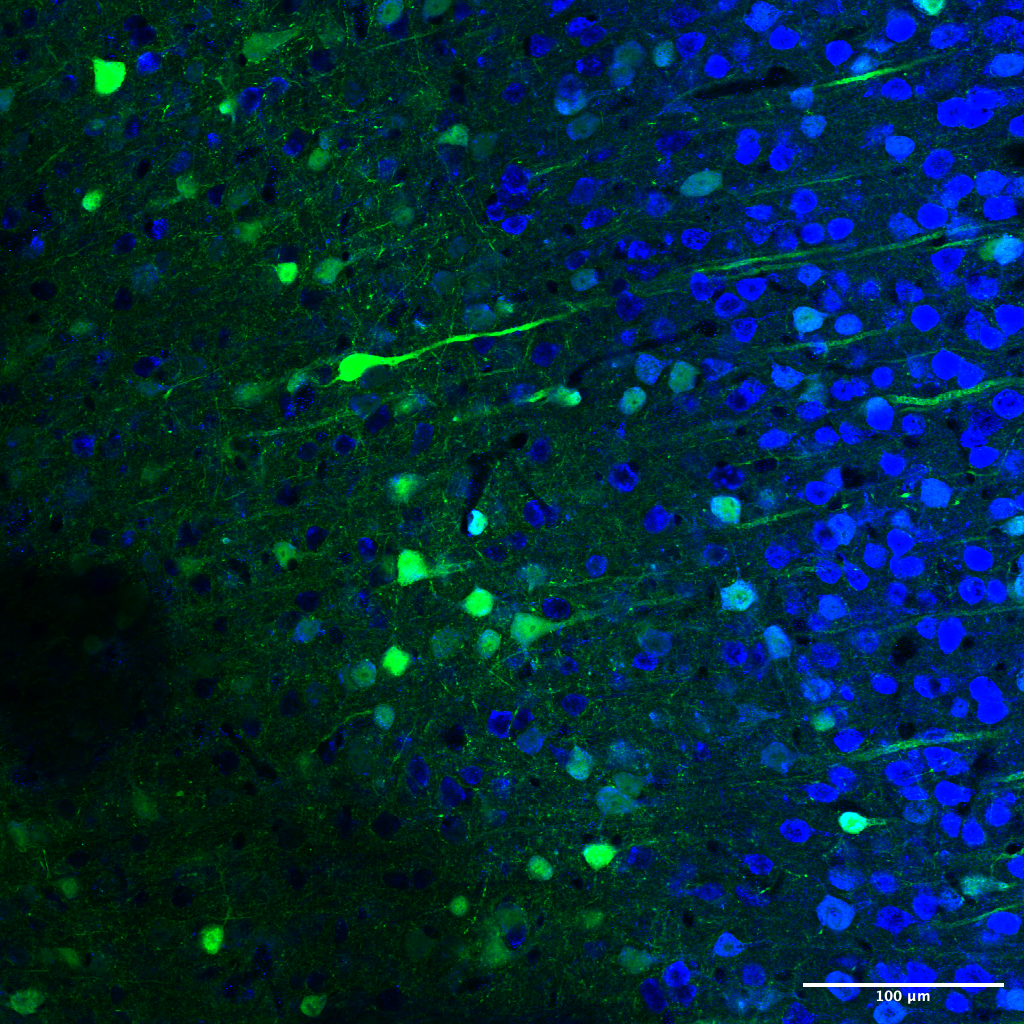

Supplement: Supplementary file 10 — EV and Appendix Figure Source Data [file 44321_2024_111_MOESM10_ESM.zip › Source Data for Expanded View and Appendix/Appendix/EMM-2024-19843_SourceData-FigureS4/S4A/MERGE IHC - 1.5x1012.tiff]

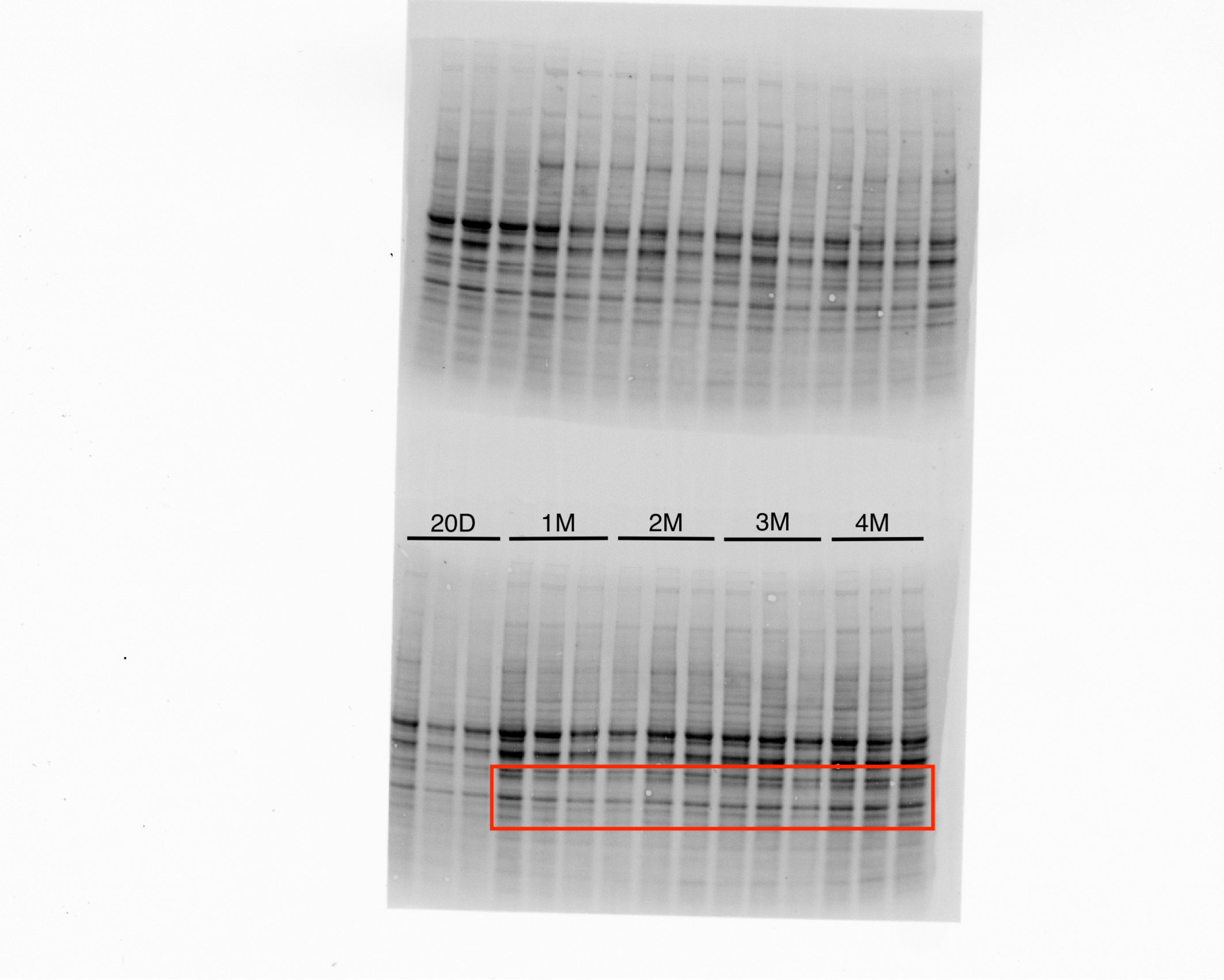

Supplement: Supplementary file 10 — EV and Appendix Figure Source Data [file 44321_2024_111_MOESM10_ESM.zip › Source Data for Expanded View and Appendix/Appendix/EMM-2024-19843_SourceData-FigureS3/S3C/western - Total Protein HPP.tiff]

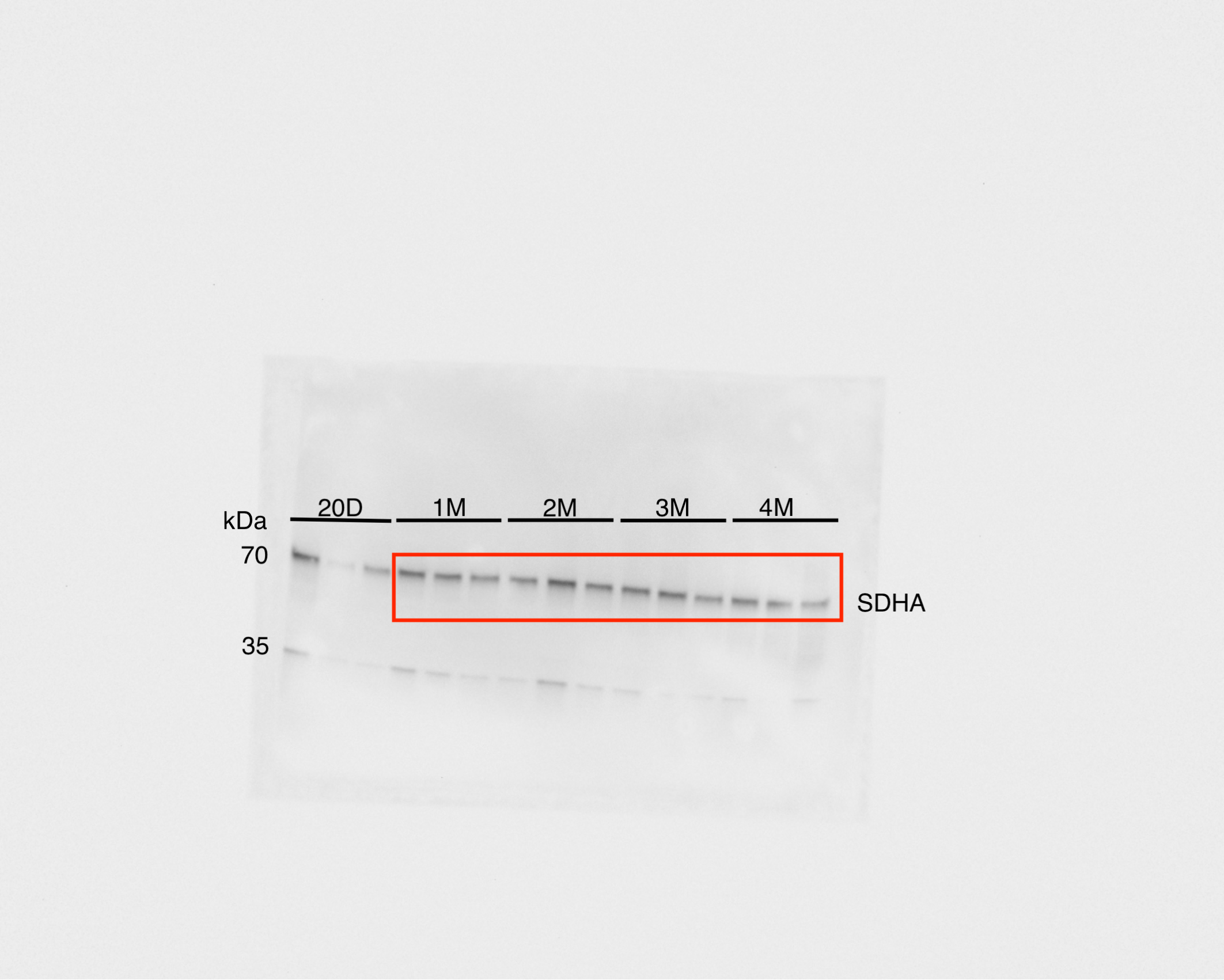

Supplement: Supplementary file 10 — EV and Appendix Figure Source Data [file 44321_2024_111_MOESM10_ESM.zip › Source Data for Expanded View and Appendix/Appendix/EMM-2024-19843_SourceData-FigureS3/S3C/western - SDHA HPP.tiff]

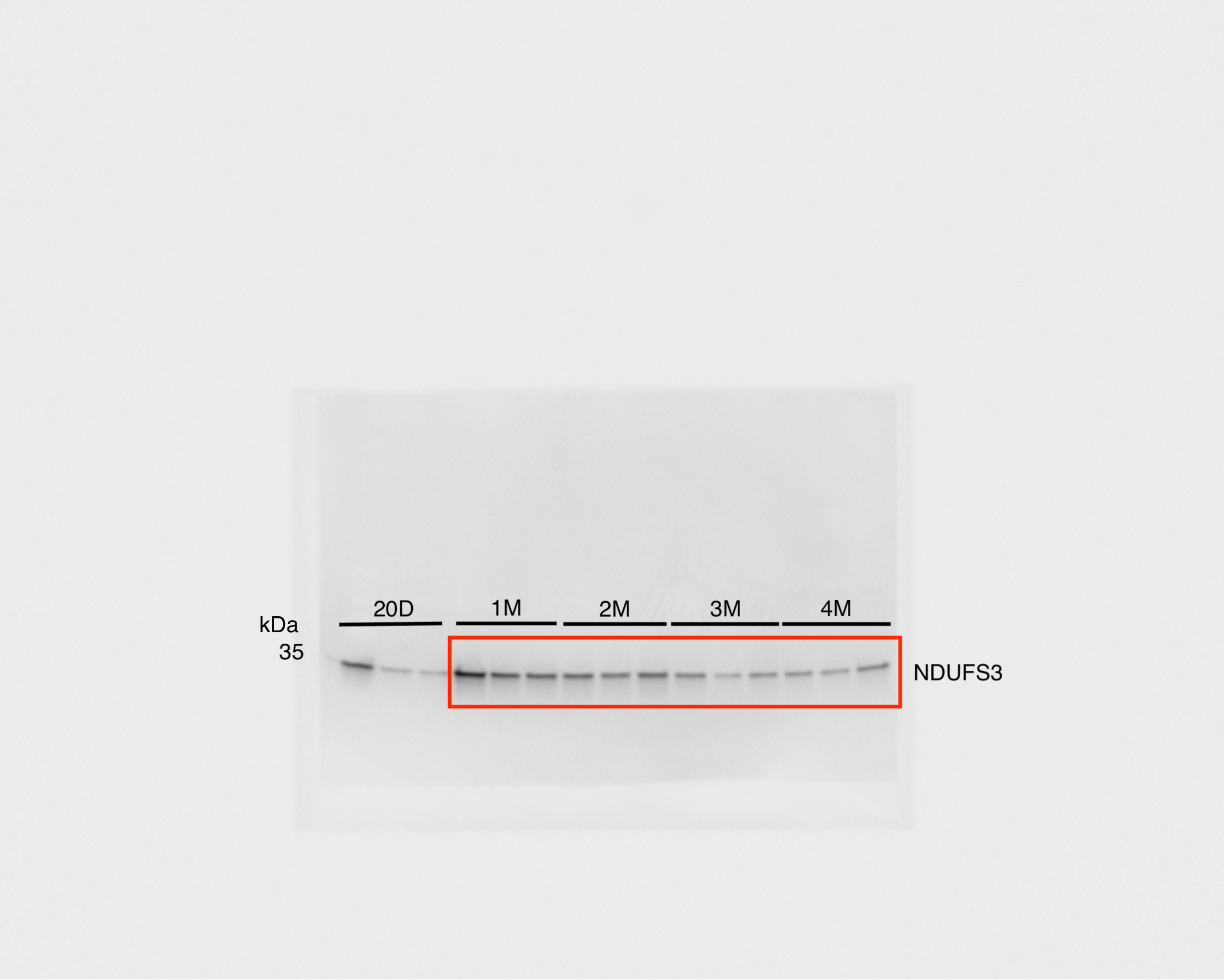

Supplement: Supplementary file 10 — EV and Appendix Figure Source Data [file 44321_2024_111_MOESM10_ESM.zip › Source Data for Expanded View and Appendix/Appendix/EMM-2024-19843_SourceData-FigureS3/S3C/western - NDUFS3 CTX.tiff]

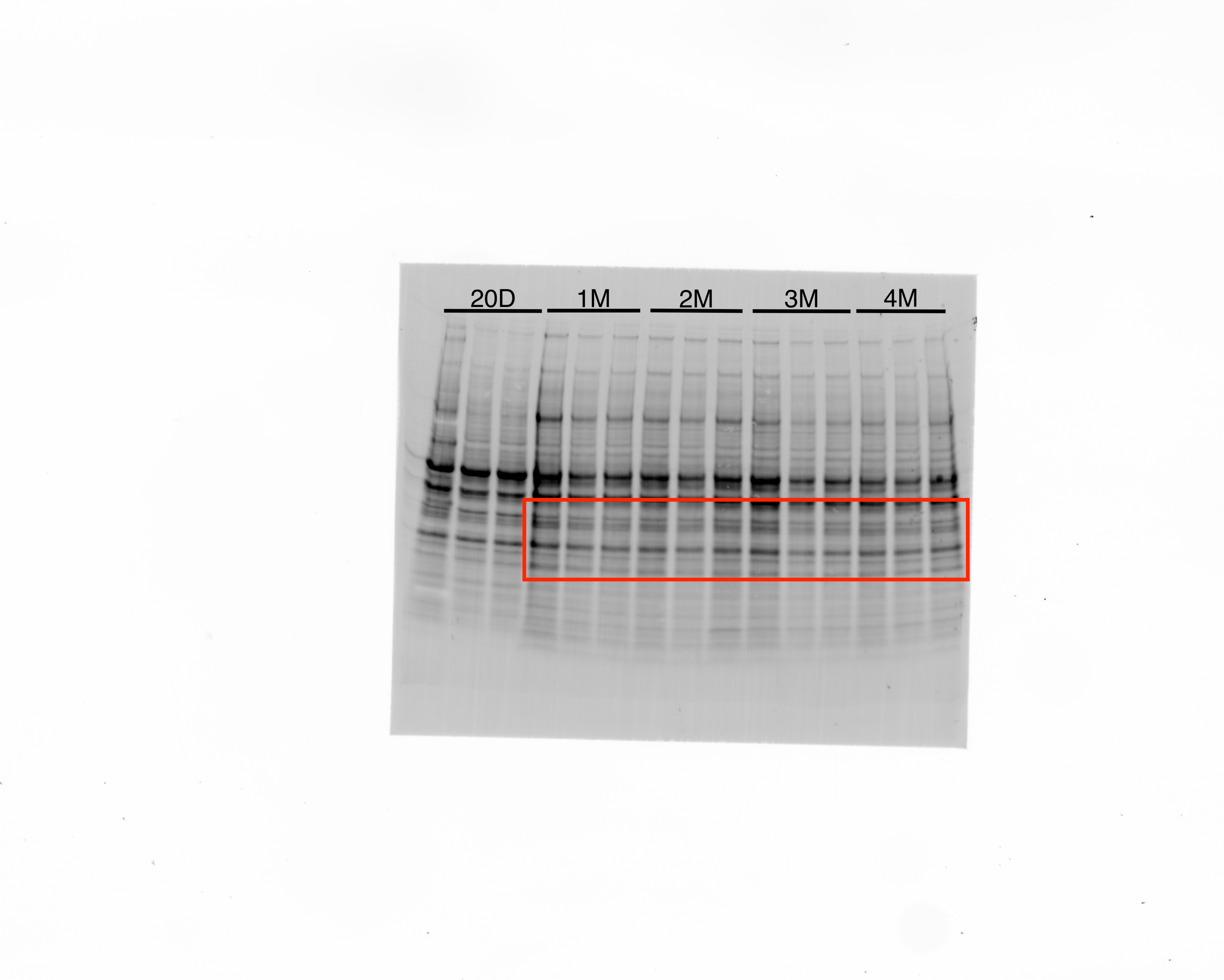

Supplement: Supplementary file 10 — EV and Appendix Figure Source Data [file 44321_2024_111_MOESM10_ESM.zip › Source Data for Expanded View and Appendix/Appendix/EMM-2024-19843_SourceData-FigureS3/S3C/western - Total Protein CTX.tiff]

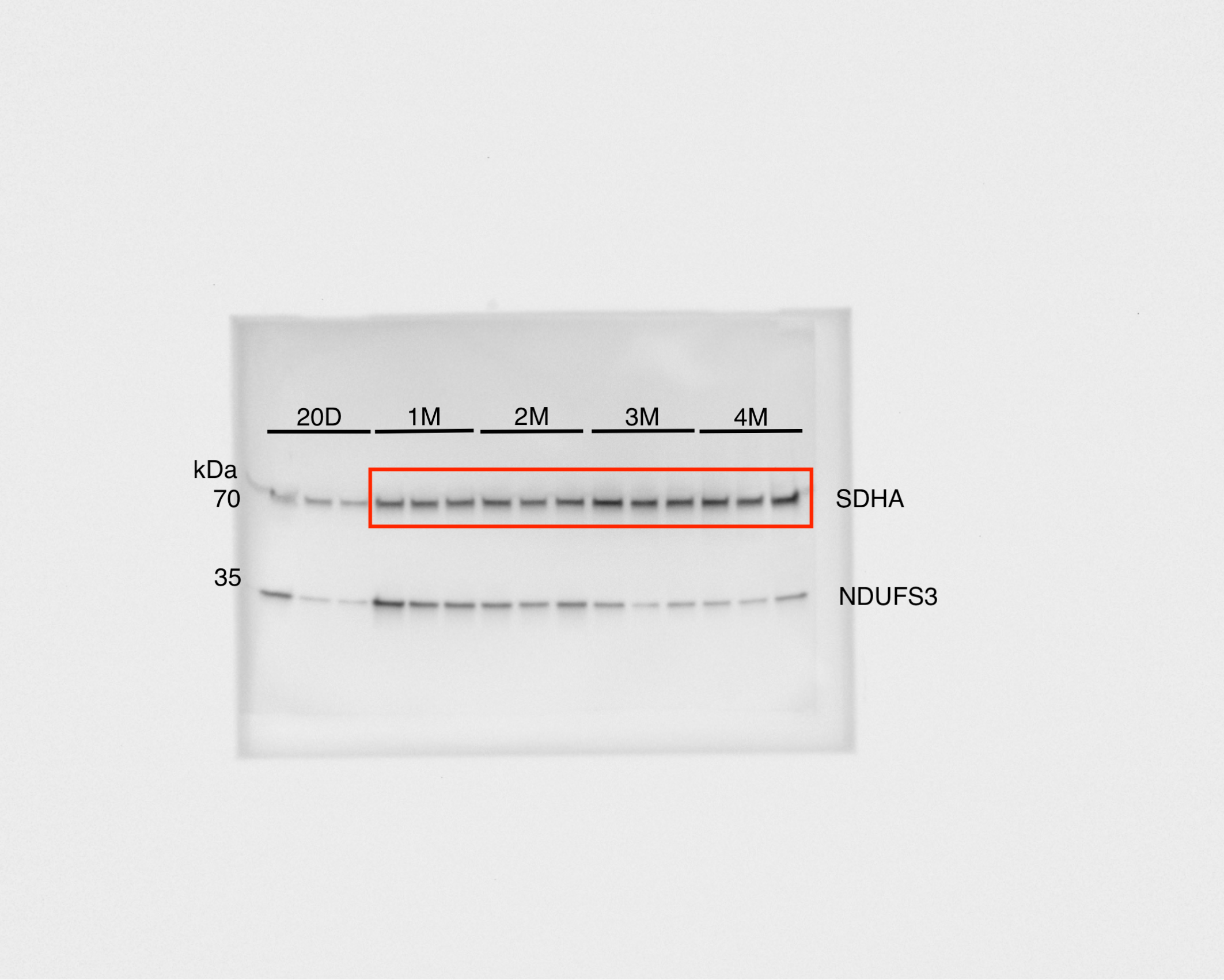

Supplement: Supplementary file 10 — EV and Appendix Figure Source Data [file 44321_2024_111_MOESM10_ESM.zip › Source Data for Expanded View and Appendix/Appendix/EMM-2024-19843_SourceData-FigureS3/S3C/western - SDHA CTX.tiff]

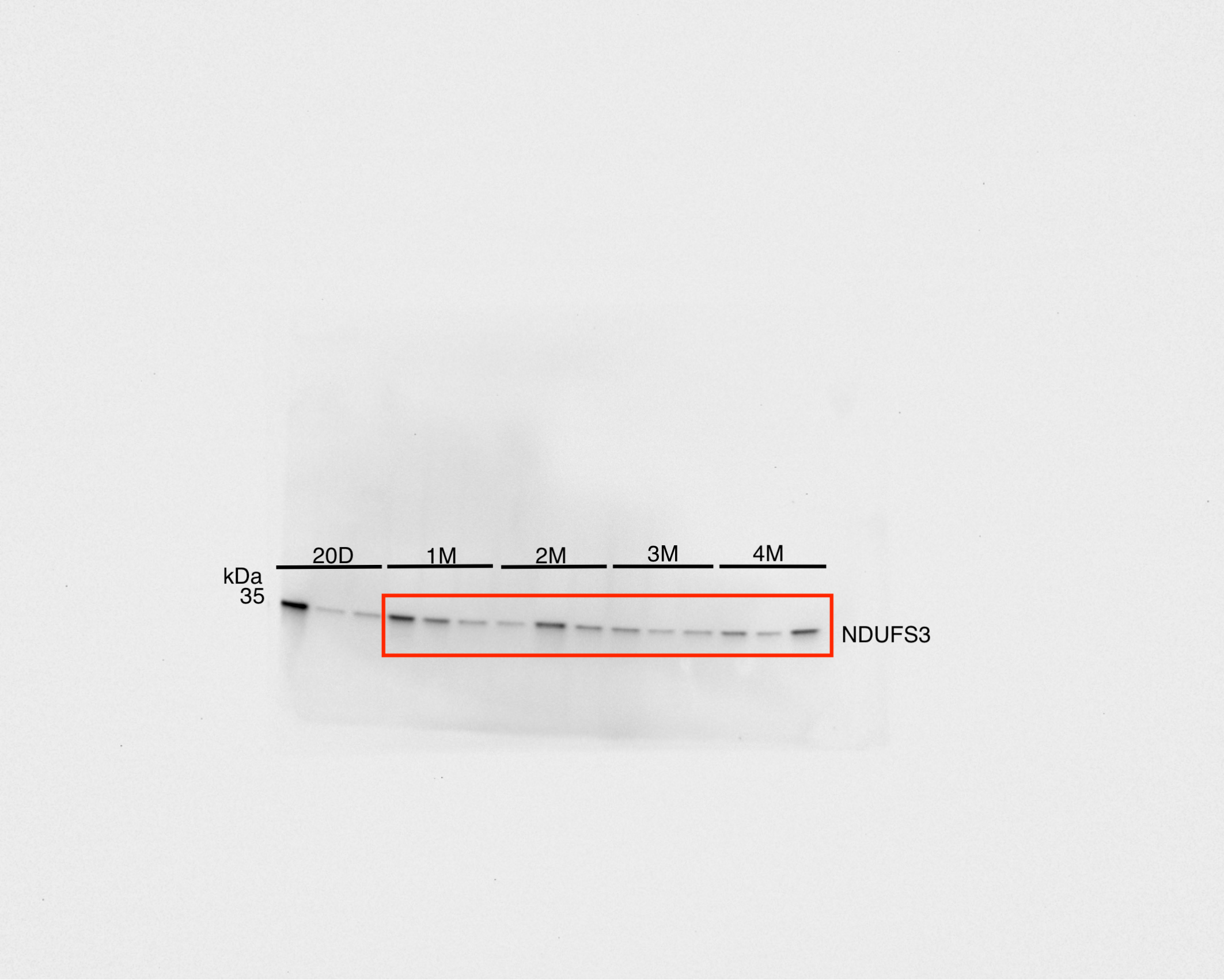

Supplement: Supplementary file 10 — EV and Appendix Figure Source Data [file 44321_2024_111_MOESM10_ESM.zip › Source Data for Expanded View and Appendix/Appendix/EMM-2024-19843_SourceData-FigureS3/S3C/western - NDUFS3 HPP.tiff]

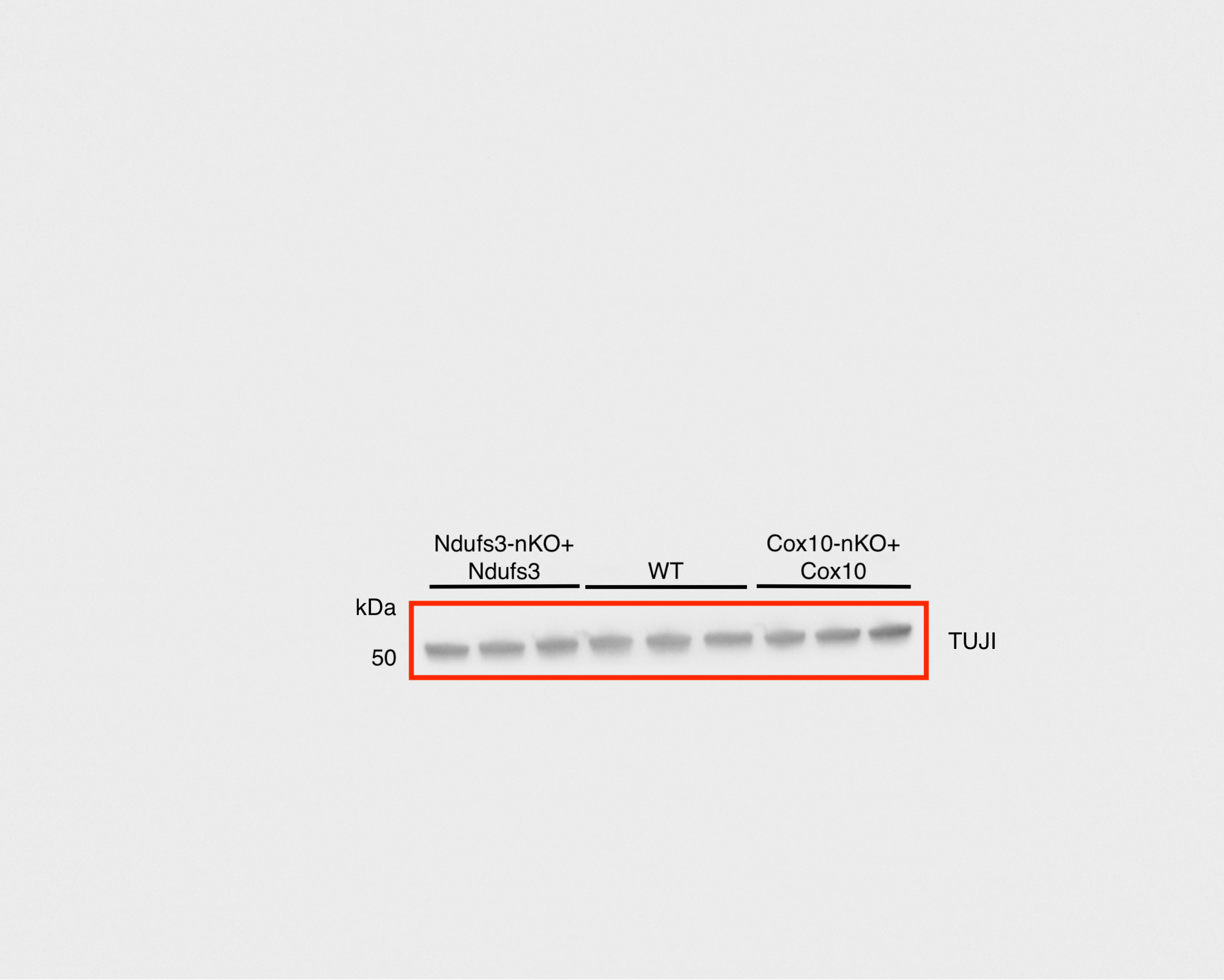

Supplement: Supplementary file 10 — EV and Appendix Figure Source Data [file 44321_2024_111_MOESM10_ESM.zip › Source Data for Expanded View and Appendix/EMM-2024-19843_SourceData-FigureEV4/EV4C/HPP/western - TUJI.tiff]

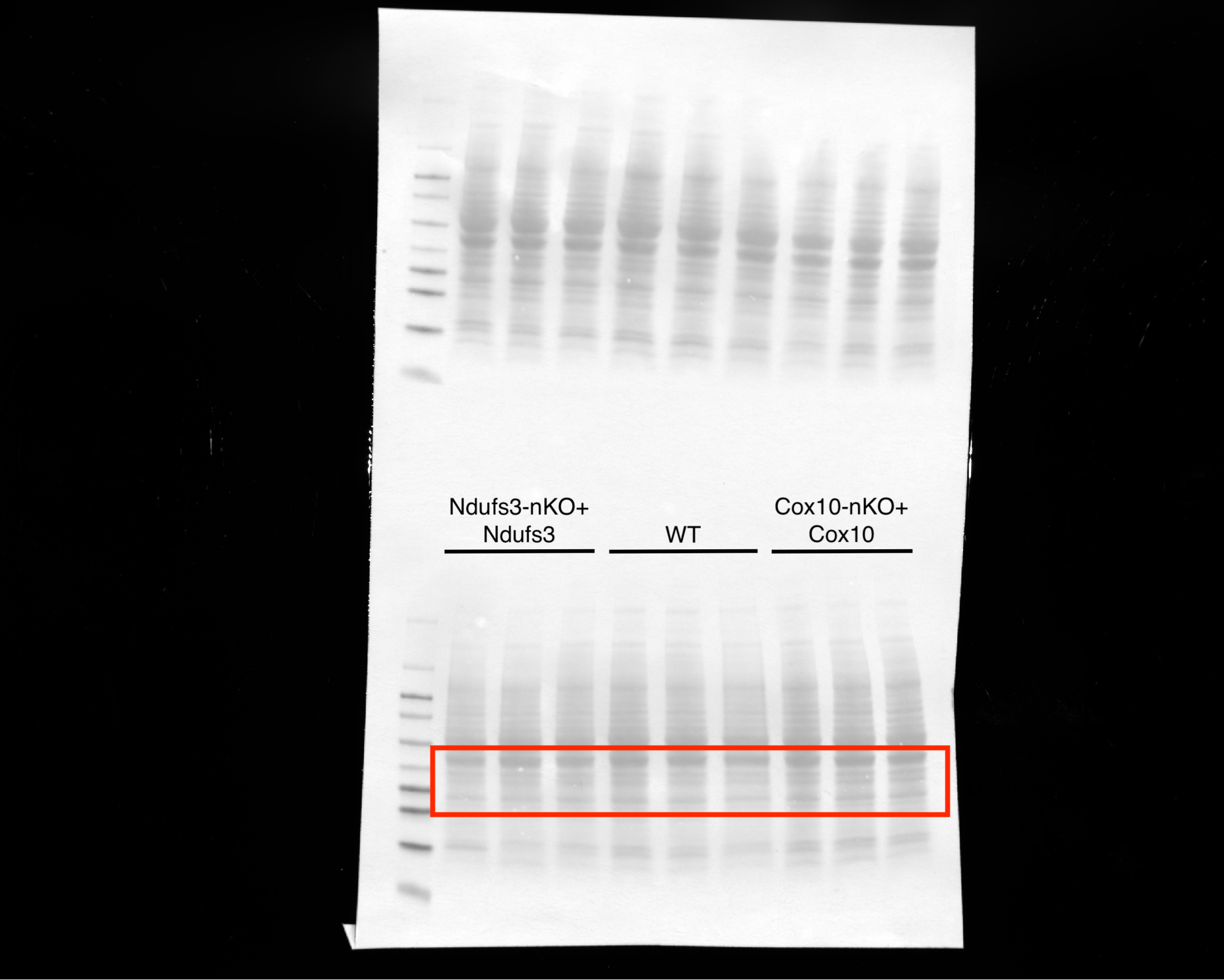

Supplement: Supplementary file 10 — EV and Appendix Figure Source Data [file 44321_2024_111_MOESM10_ESM.zip › Source Data for Expanded View and Appendix/EMM-2024-19843_SourceData-FigureEV4/EV4C/HPP/western - Total Protein.tiff]

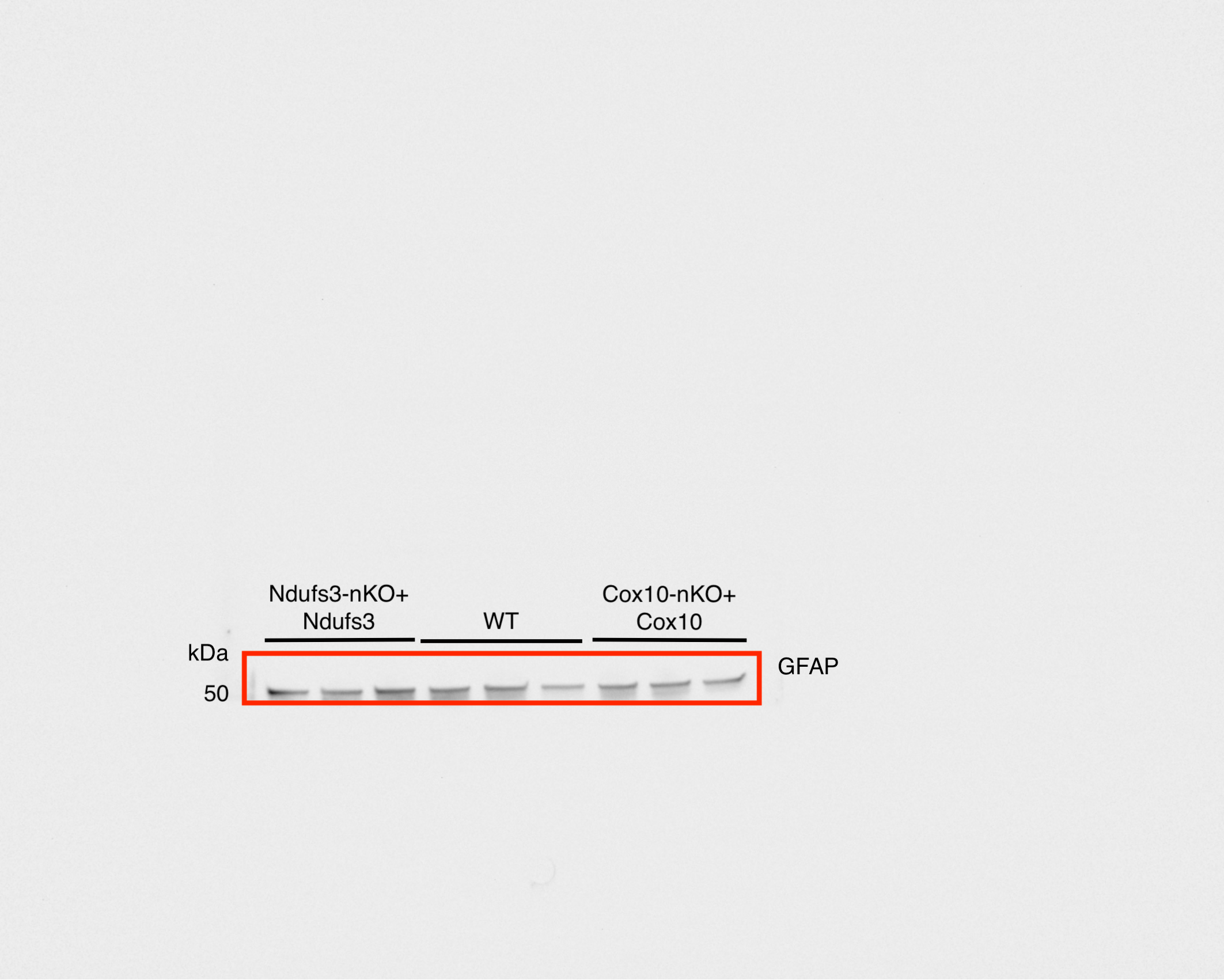

Supplement: Supplementary file 10 — EV and Appendix Figure Source Data [file 44321_2024_111_MOESM10_ESM.zip › Source Data for Expanded View and Appendix/EMM-2024-19843_SourceData-FigureEV4/EV4C/HPP/western - GFAP.tiff]

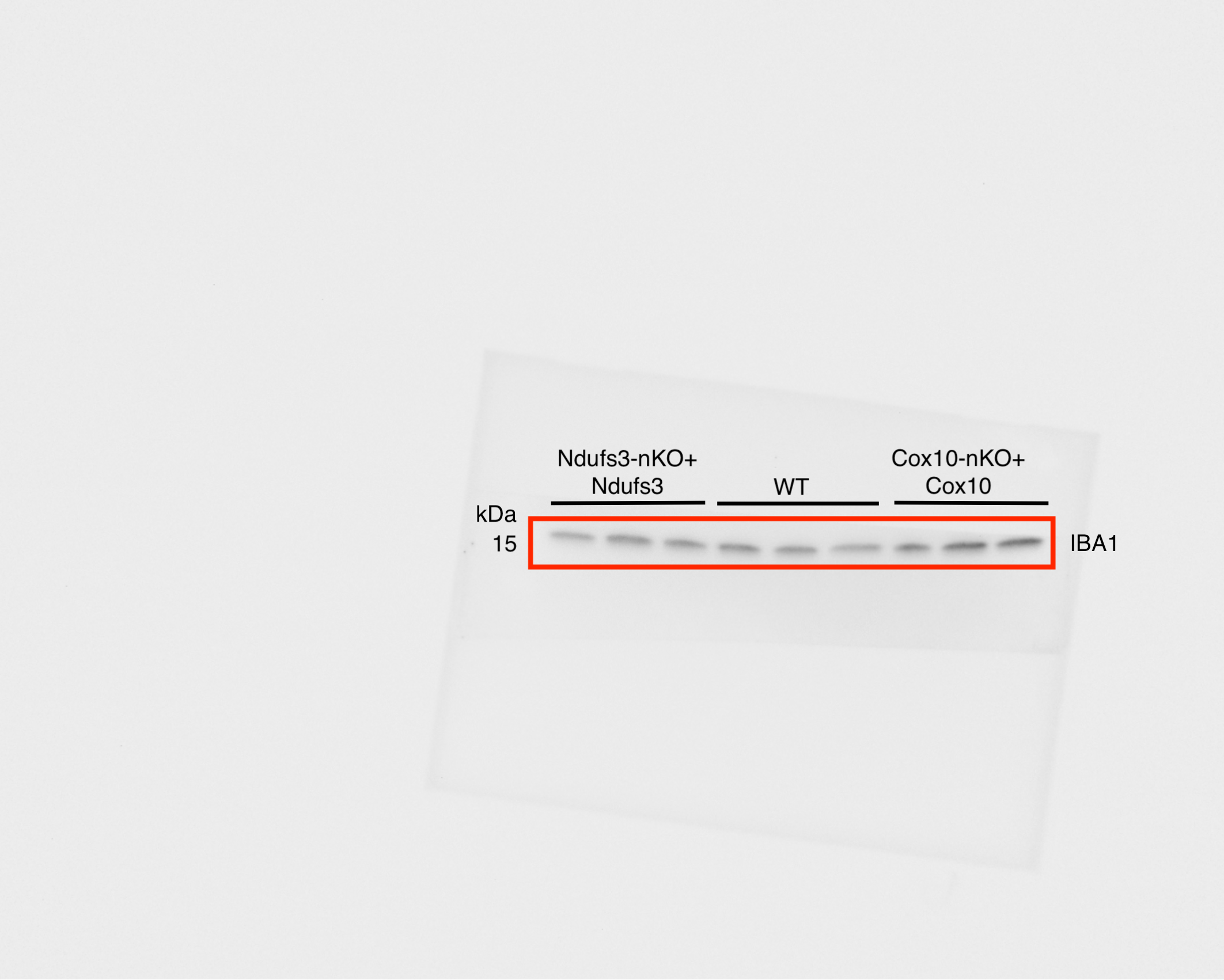

Supplement: Supplementary file 10 — EV and Appendix Figure Source Data [file 44321_2024_111_MOESM10_ESM.zip › Source Data for Expanded View and Appendix/EMM-2024-19843_SourceData-FigureEV4/EV4C/HPP/western - IBA1.tiff]

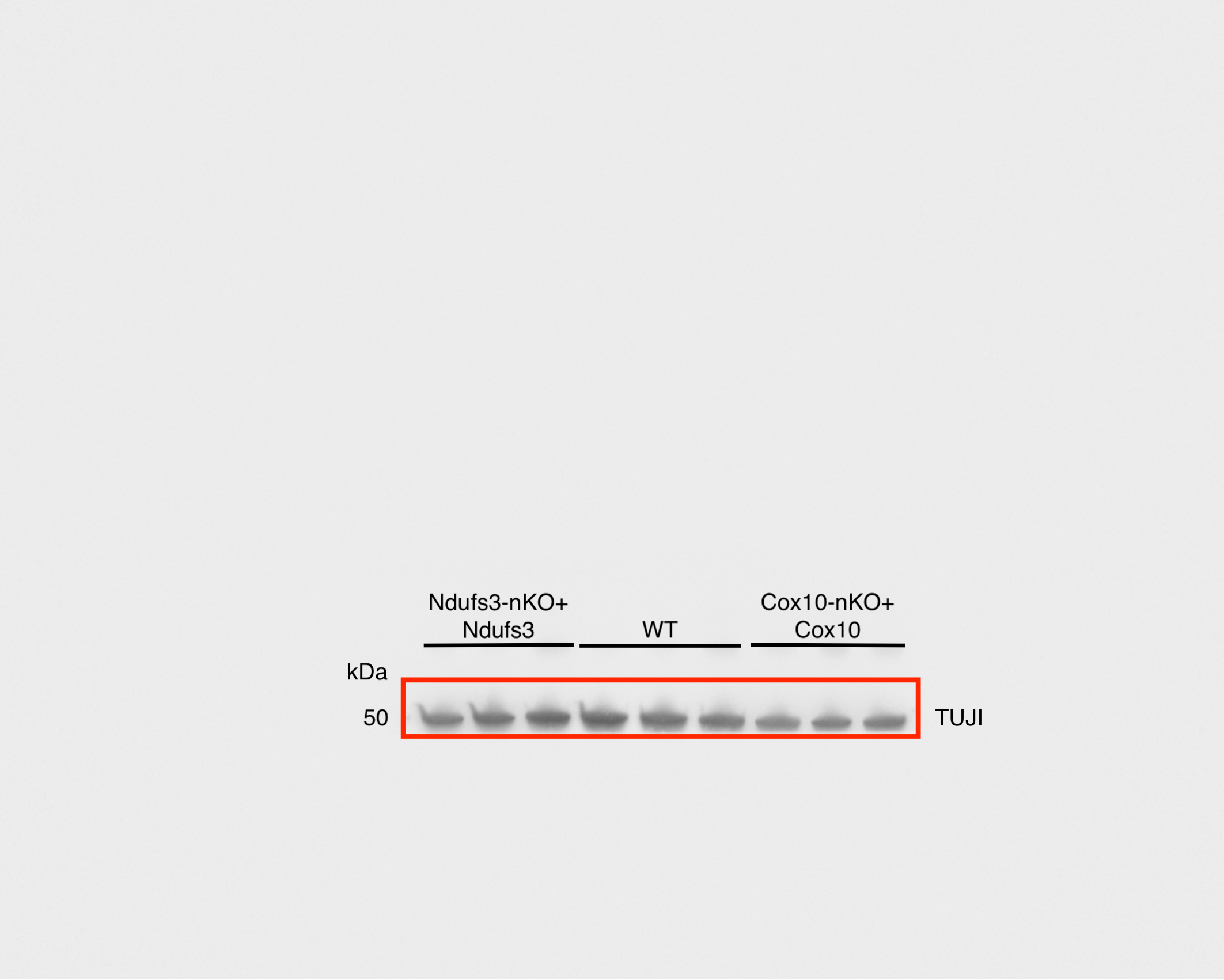

Supplement: Supplementary file 10 — EV and Appendix Figure Source Data [file 44321_2024_111_MOESM10_ESM.zip › Source Data for Expanded View and Appendix/EMM-2024-19843_SourceData-FigureEV4/EV4C/CTX/western - TUJI.tiff]

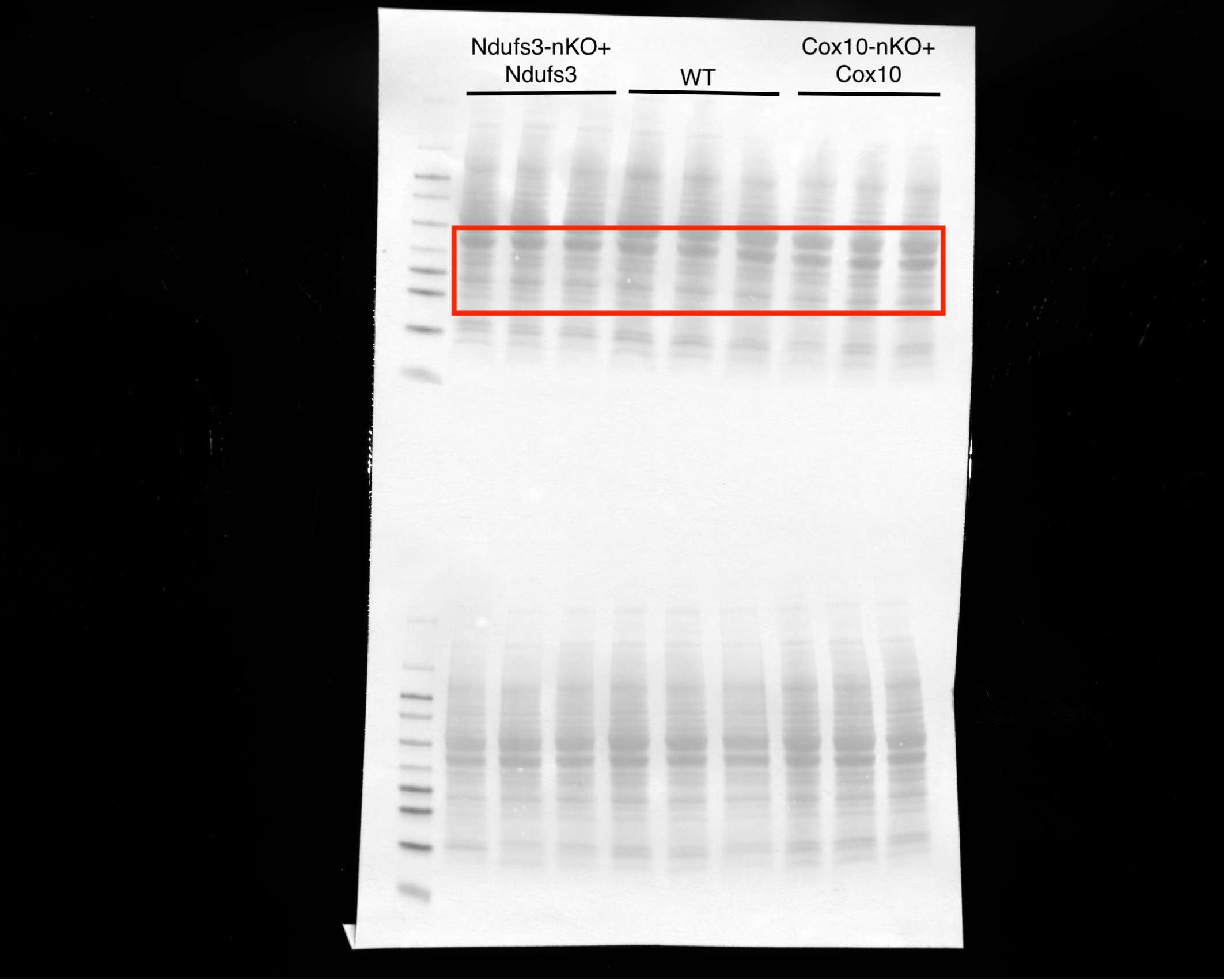

Supplement: Supplementary file 10 — EV and Appendix Figure Source Data [file 44321_2024_111_MOESM10_ESM.zip › Source Data for Expanded View and Appendix/EMM-2024-19843_SourceData-FigureEV4/EV4C/CTX/western - Total Protein.tiff]

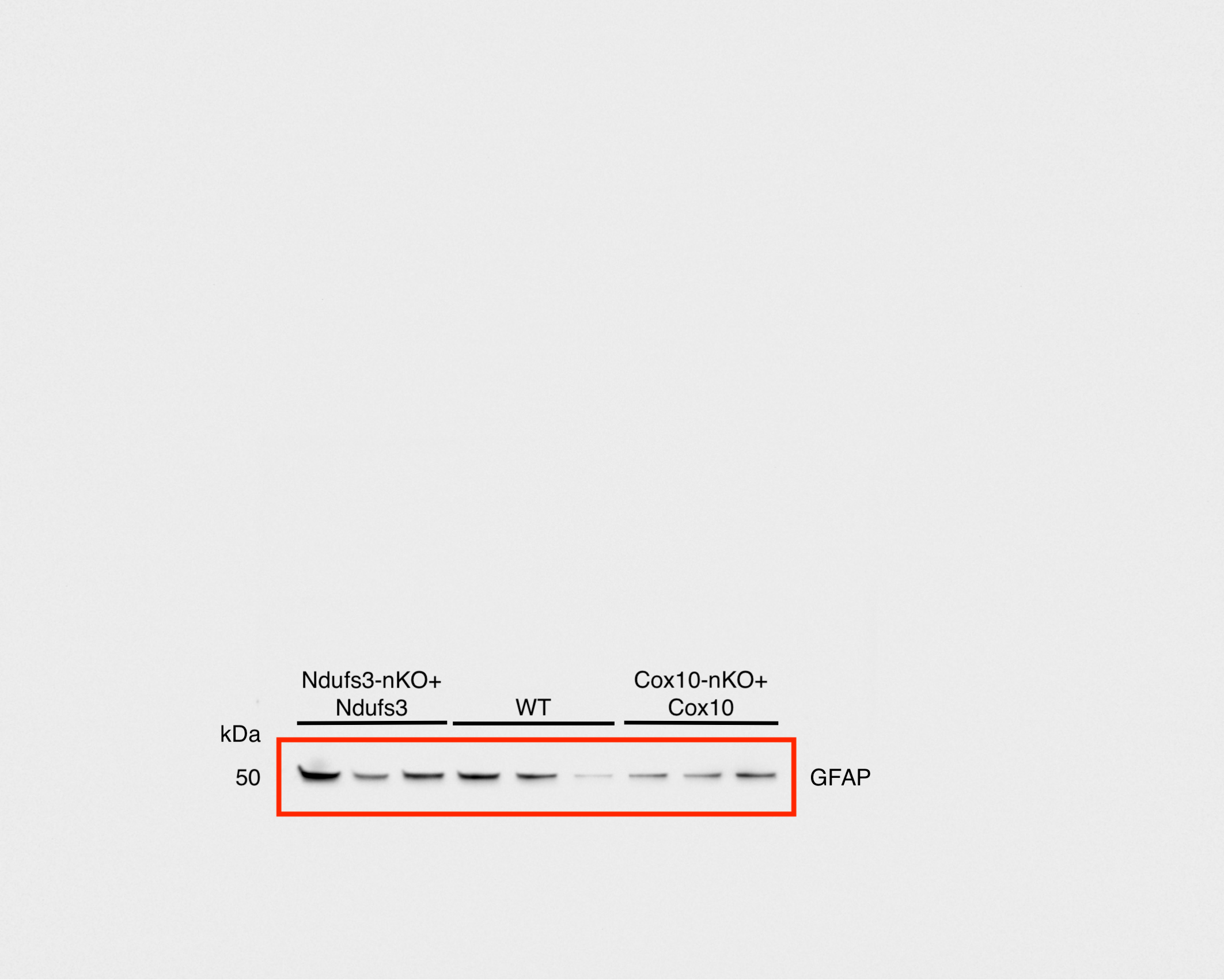

Supplement: Supplementary file 10 — EV and Appendix Figure Source Data [file 44321_2024_111_MOESM10_ESM.zip › Source Data for Expanded View and Appendix/EMM-2024-19843_SourceData-FigureEV4/EV4C/CTX/western - GFAP.tiff]

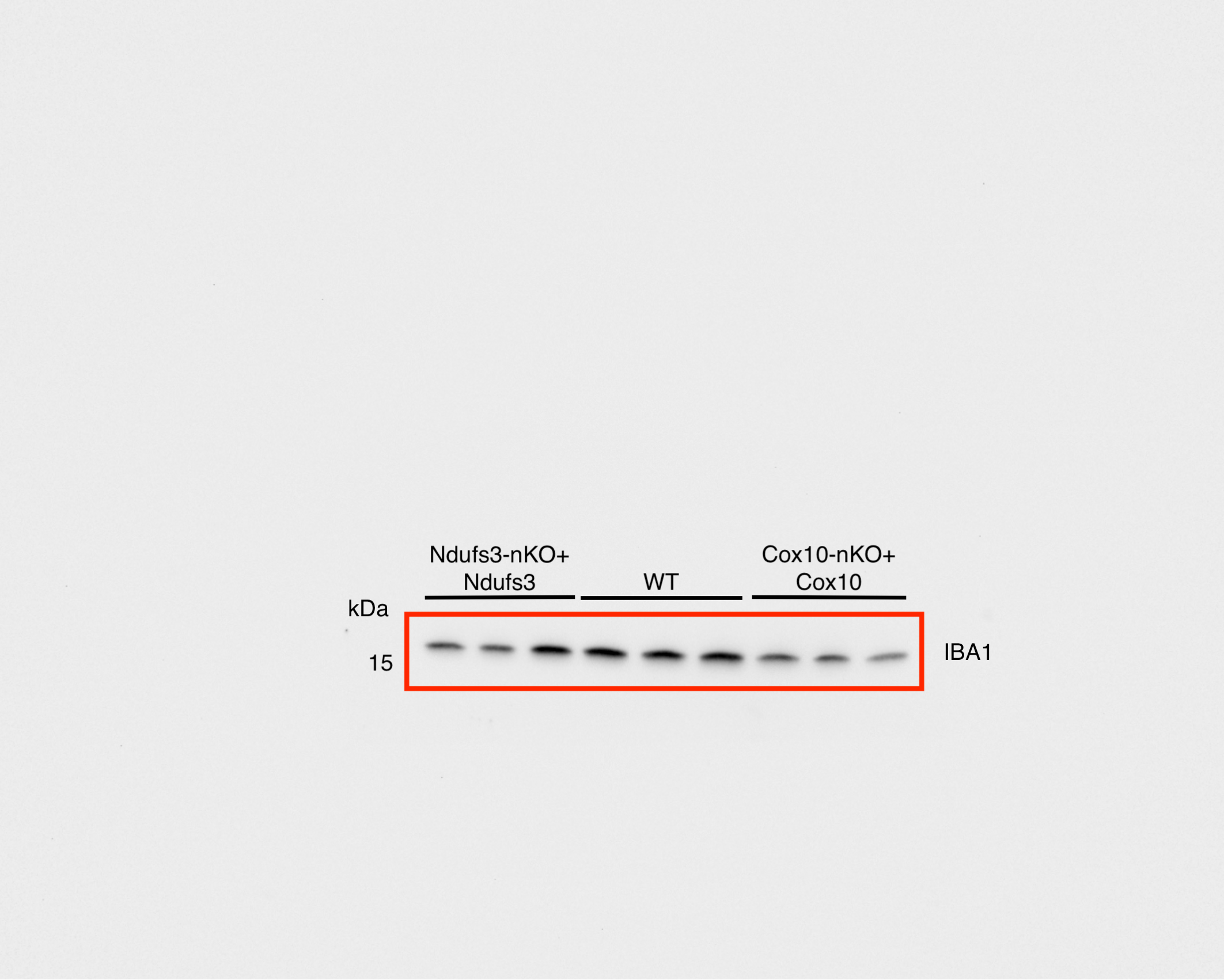

Supplement: Supplementary file 10 — EV and Appendix Figure Source Data [file 44321_2024_111_MOESM10_ESM.zip › Source Data for Expanded View and Appendix/EMM-2024-19843_SourceData-FigureEV4/EV4C/CTX/western - IBA1.tiff]

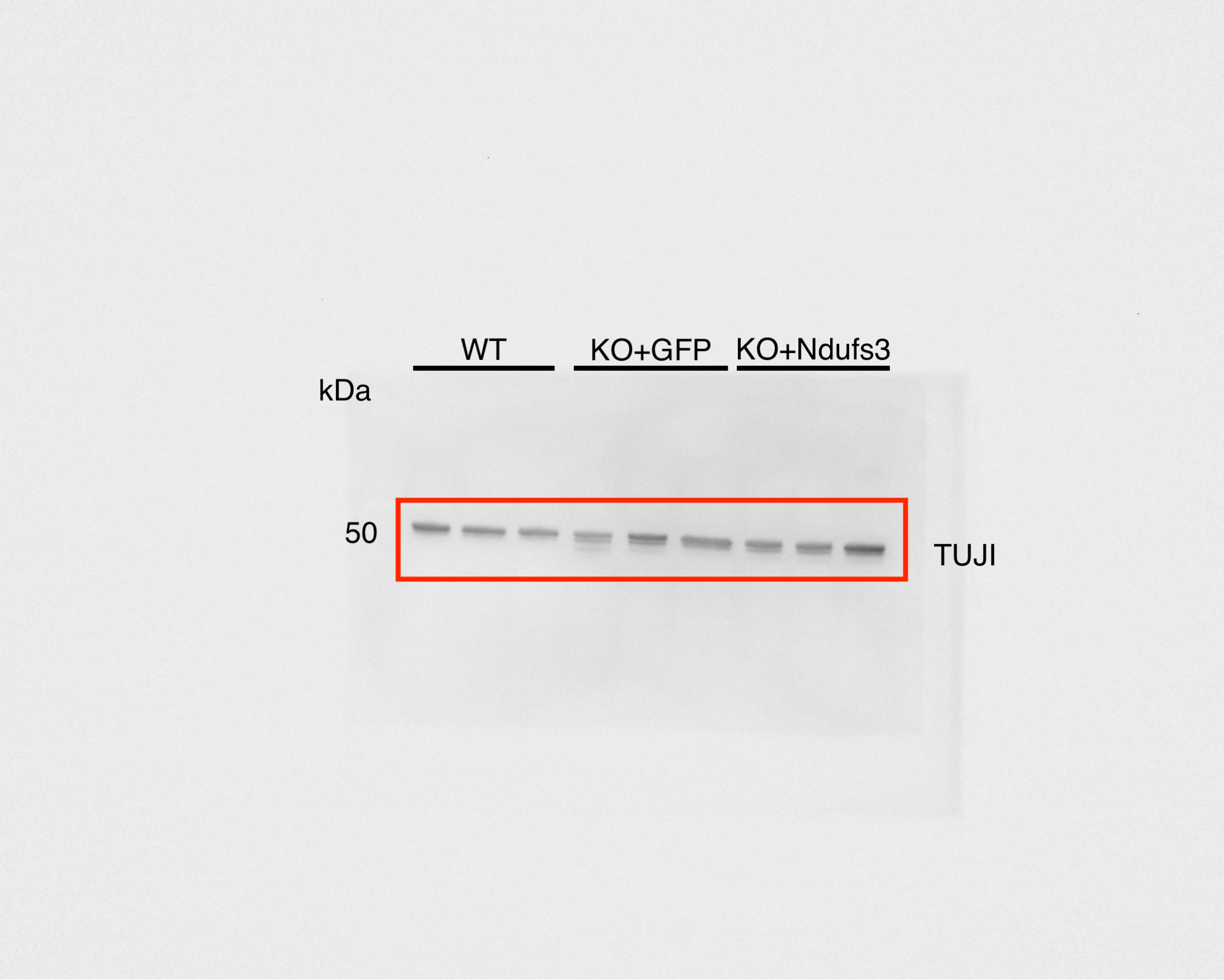

Supplement: Supplementary file 10 — EV and Appendix Figure Source Data [file 44321_2024_111_MOESM10_ESM.zip › Source Data for Expanded View and Appendix/EMM-2024-19843_SourceData-FigureEV2/EV2A/HIPP/western - TUJI.tiff]

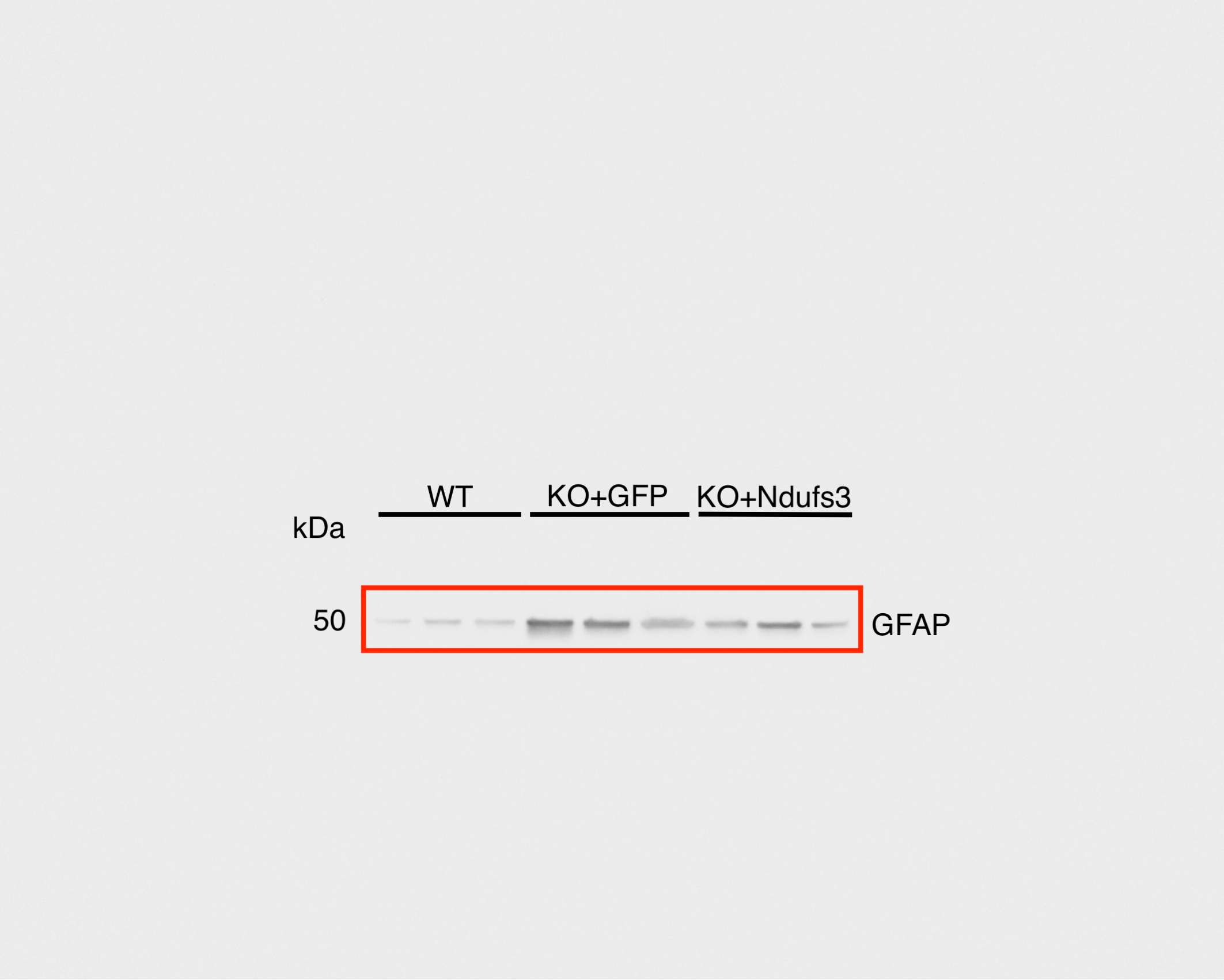

Supplement: Supplementary file 10 — EV and Appendix Figure Source Data [file 44321_2024_111_MOESM10_ESM.zip › Source Data for Expanded View and Appendix/EMM-2024-19843_SourceData-FigureEV2/EV2A/HIPP/western - GFAP.tiff]

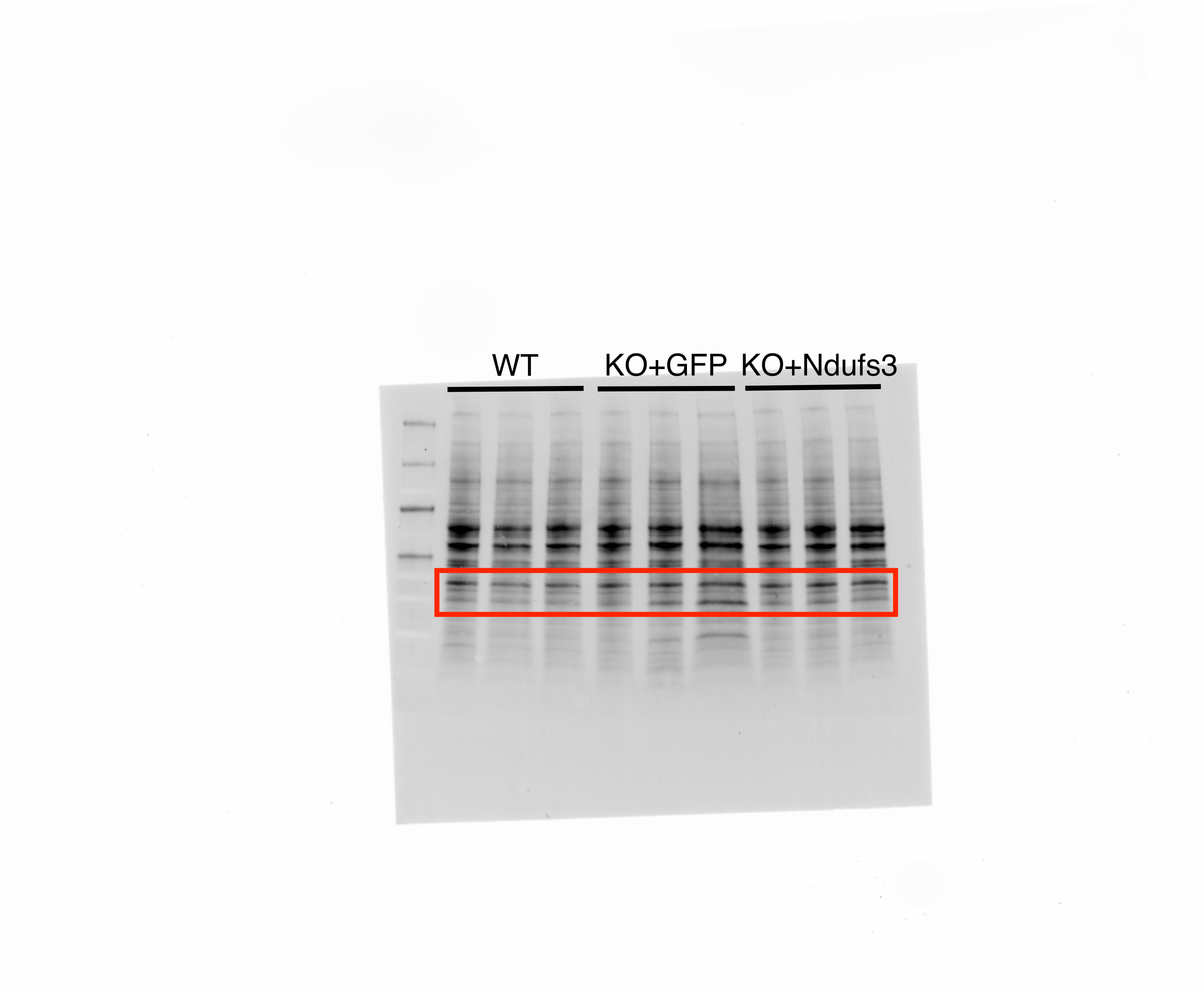

Supplement: Supplementary file 10 — EV and Appendix Figure Source Data [file 44321_2024_111_MOESM10_ESM.zip › Source Data for Expanded View and Appendix/EMM-2024-19843_SourceData-FigureEV2/EV2A/HIPP/western - Total Protein.tif]

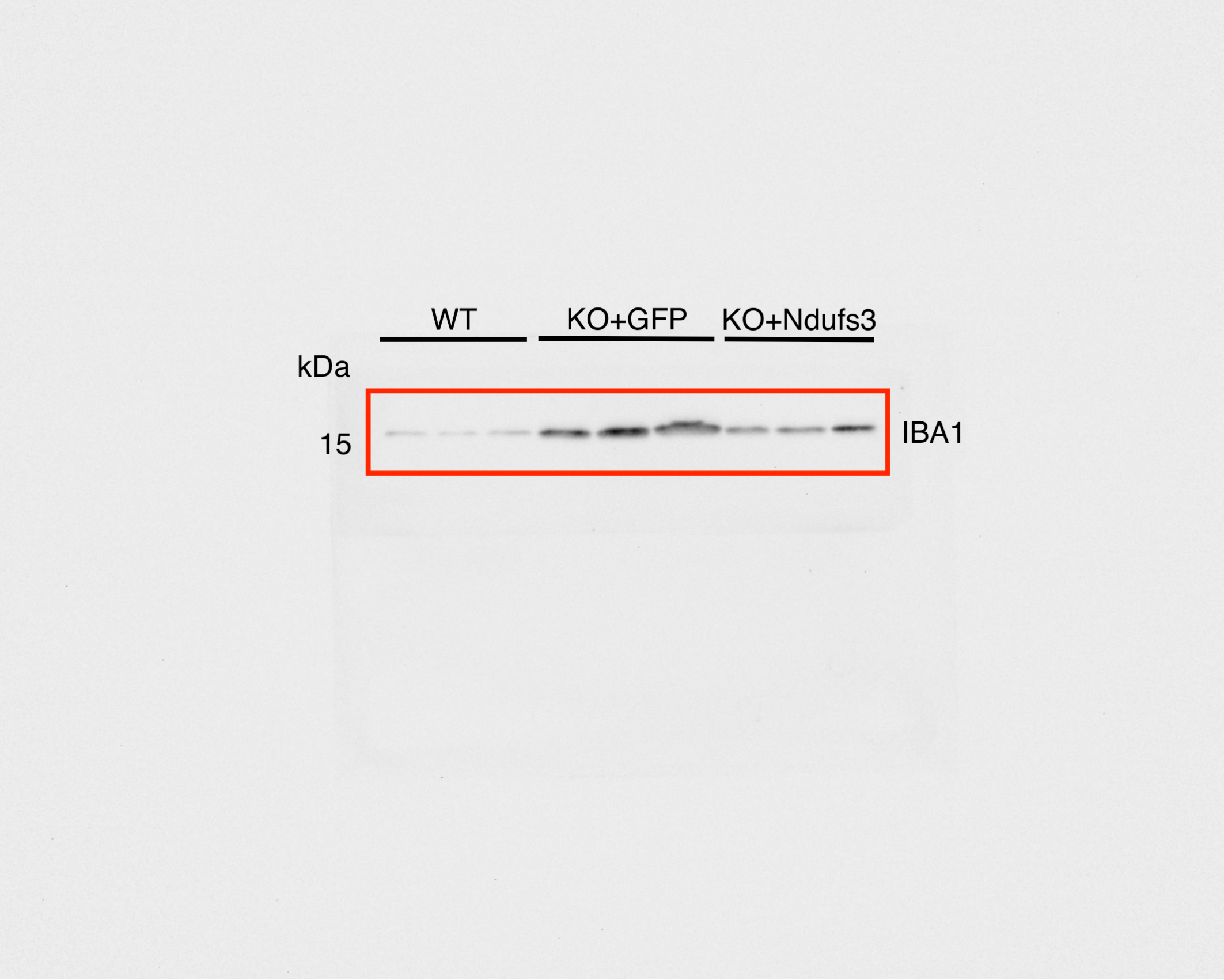

Supplement: Supplementary file 10 — EV and Appendix Figure Source Data [file 44321_2024_111_MOESM10_ESM.zip › Source Data for Expanded View and Appendix/EMM-2024-19843_SourceData-FigureEV2/EV2A/HIPP/western - IBA1.tiff]

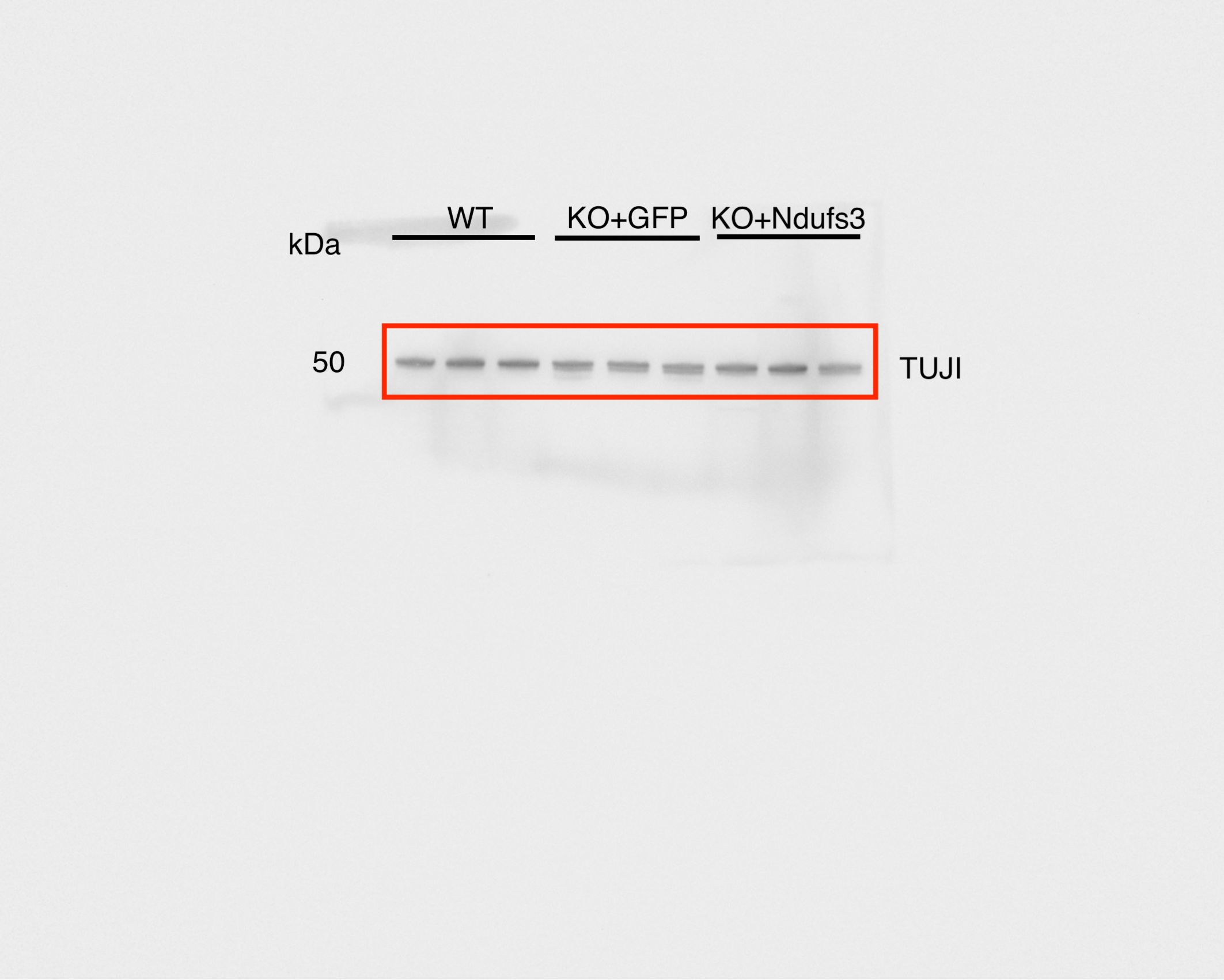

Supplement: Supplementary file 10 — EV and Appendix Figure Source Data [file 44321_2024_111_MOESM10_ESM.zip › Source Data for Expanded View and Appendix/EMM-2024-19843_SourceData-FigureEV2/EV2A/CTX/western - TUJI.tiff]

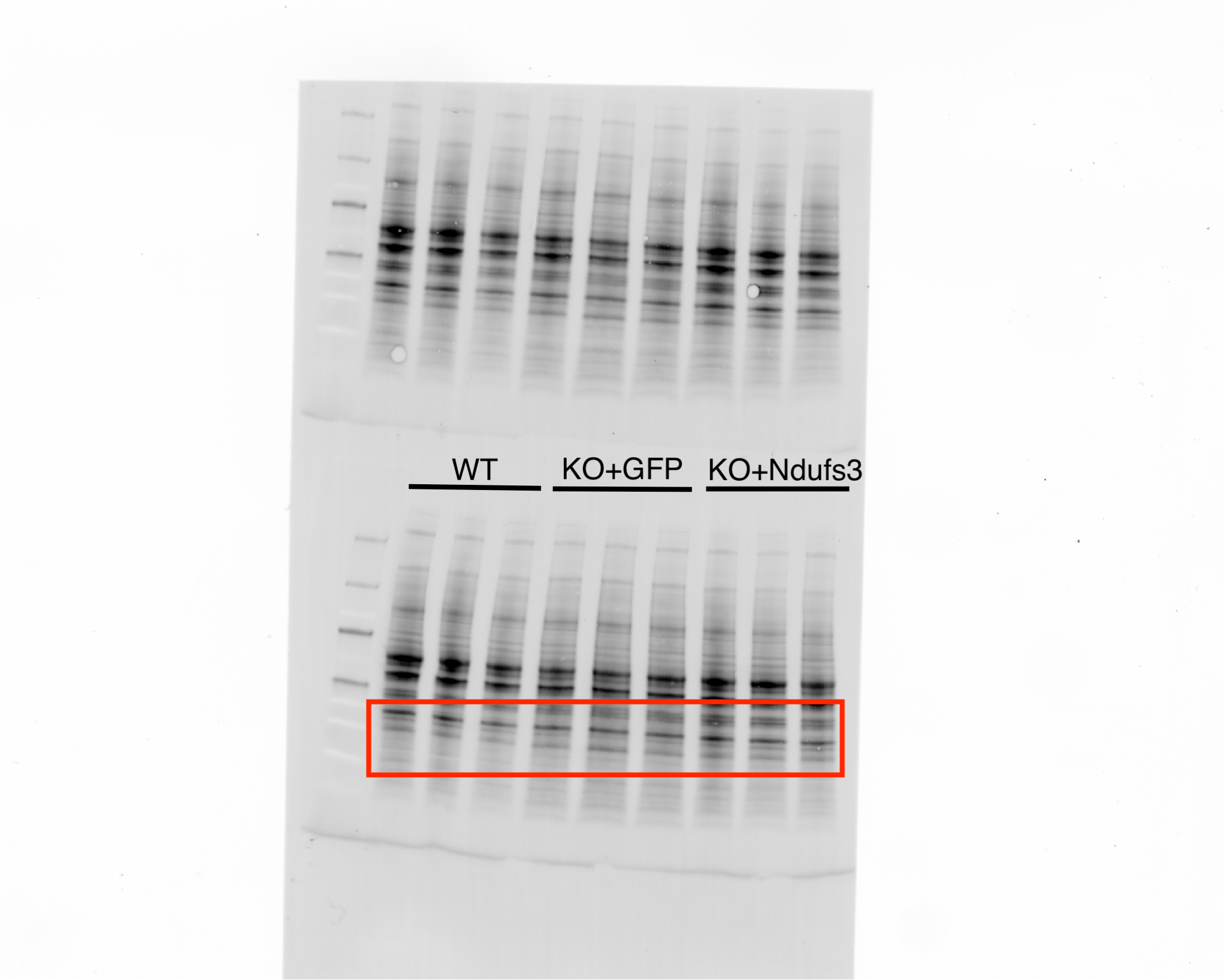

Supplement: Supplementary file 10 — EV and Appendix Figure Source Data [file 44321_2024_111_MOESM10_ESM.zip › Source Data for Expanded View and Appendix/EMM-2024-19843_SourceData-FigureEV2/EV2A/CTX/western - Total Protein.tiff]

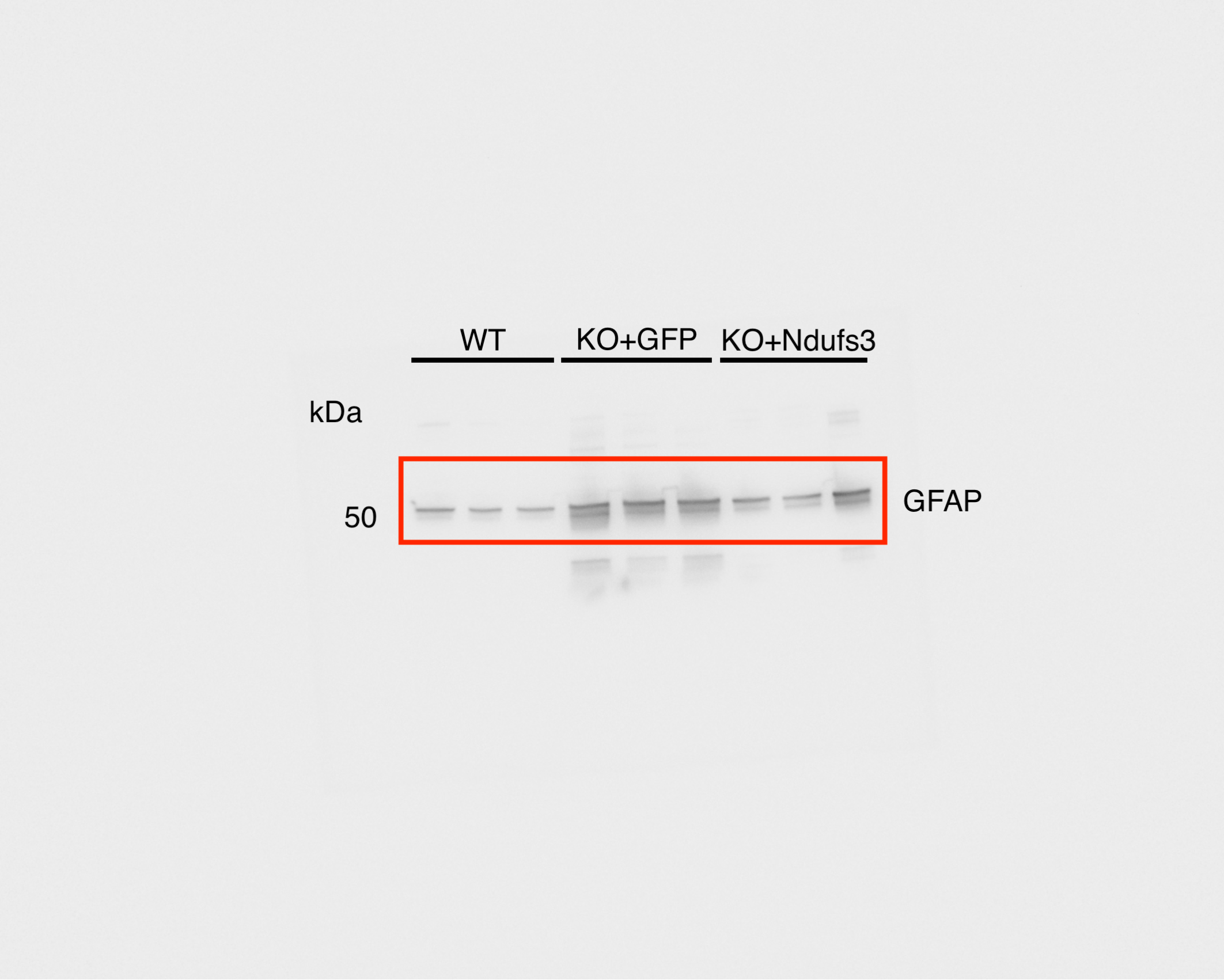

Supplement: Supplementary file 10 — EV and Appendix Figure Source Data [file 44321_2024_111_MOESM10_ESM.zip › Source Data for Expanded View and Appendix/EMM-2024-19843_SourceData-FigureEV2/EV2A/CTX/western - GFAP.tiff]

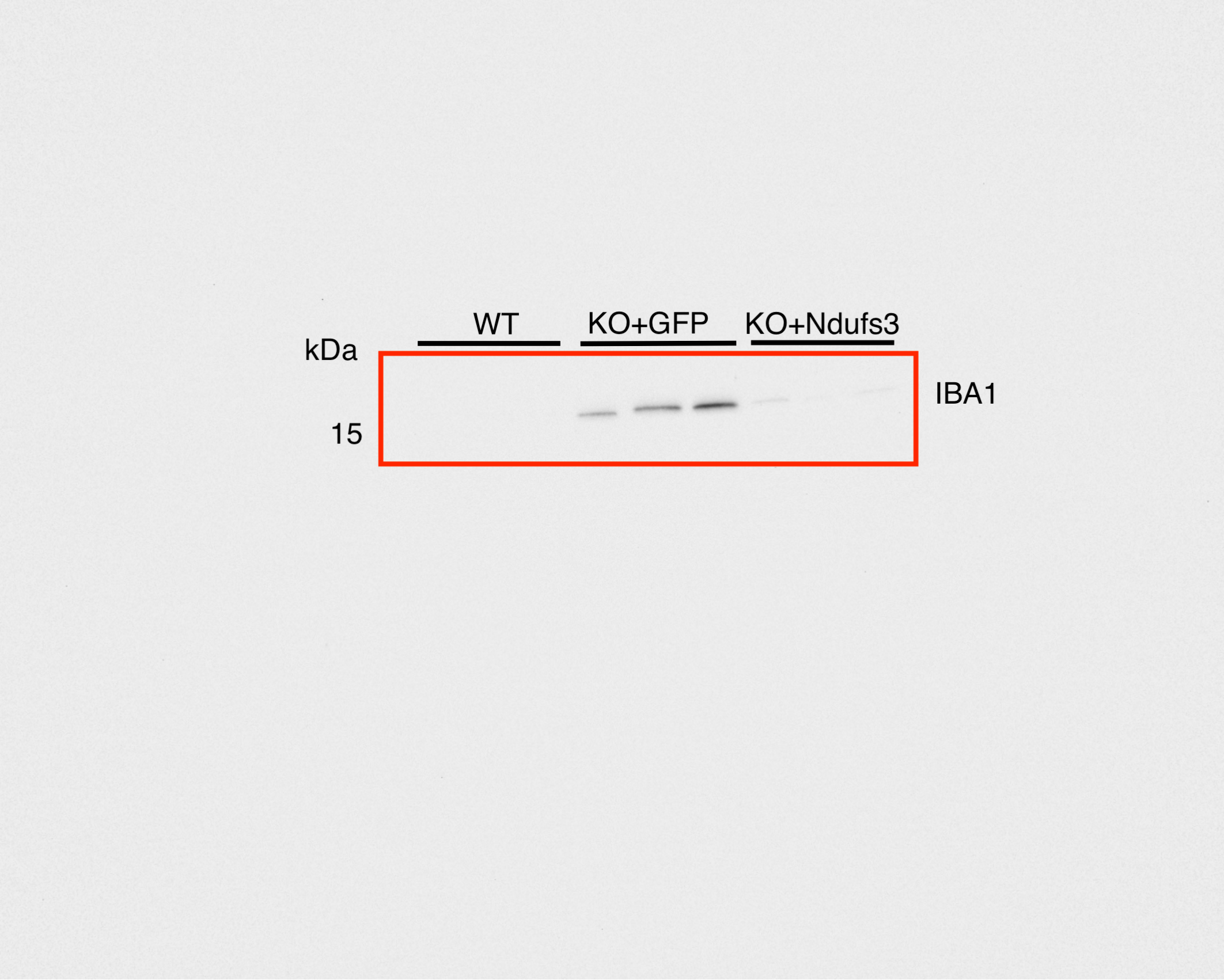

Supplement: Supplementary file 10 — EV and Appendix Figure Source Data [file 44321_2024_111_MOESM10_ESM.zip › Source Data for Expanded View and Appendix/EMM-2024-19843_SourceData-FigureEV2/EV2A/CTX/western - IBA1.tiff]
