# Supplementary material for: Restoration of defective oxidative phosphorylation to a subset of neurons prevents mitochondrial encephalopathy
Source: EMBO Mol Med. 2024 Aug 21;16(9):13. doi: 10.1038/s44321-024-00111-4 (PMC11392956; doi:10.1038/s44321-024-00111-4)
Supplement: Supplementary file 11 — Expanded View Figures [file 44321_2024_111_MOESM11_ESM.pdf]

Expanded View Figures

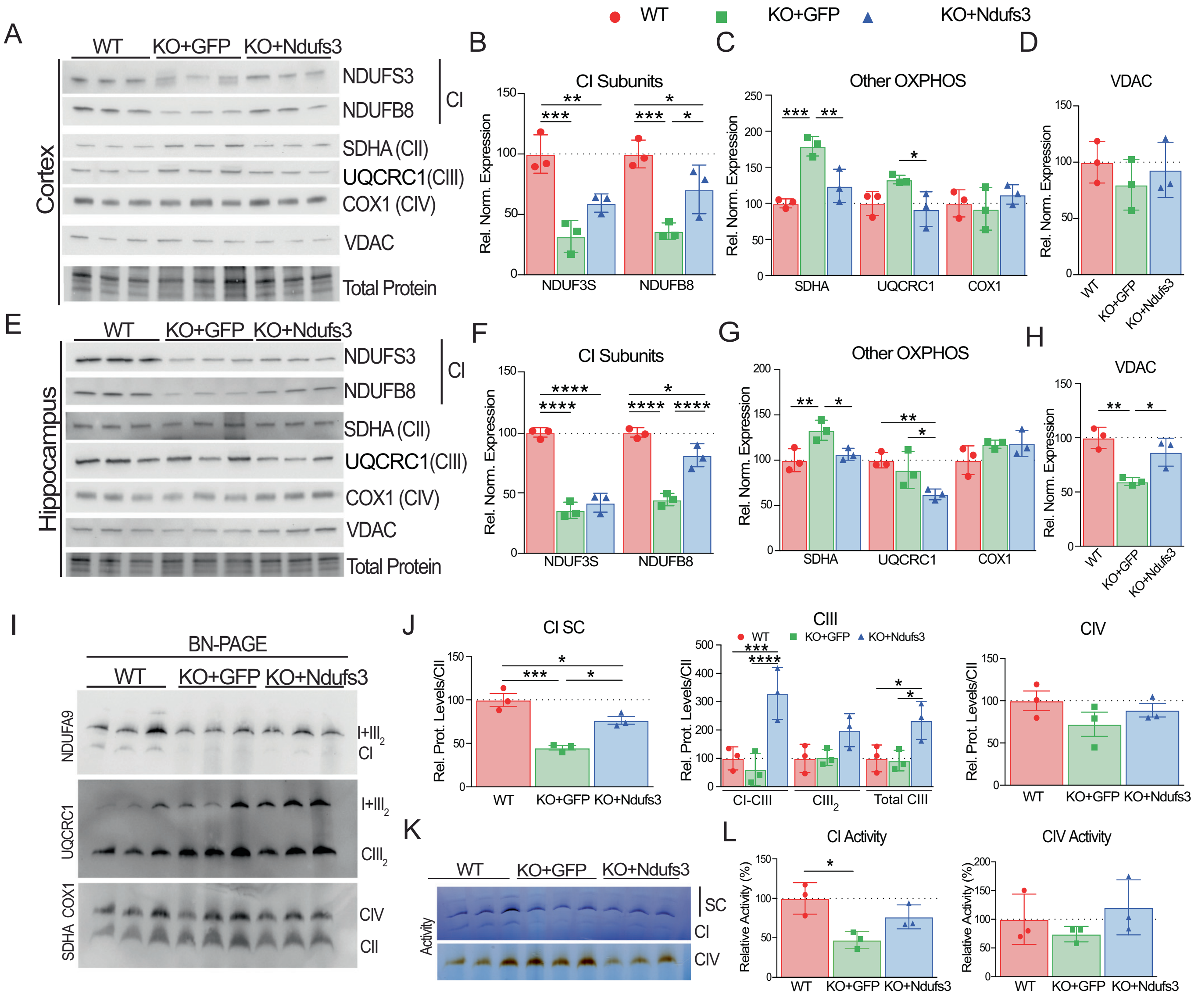

Figure EV1. Restoration of NDUFS3 in females *Ndufs3*-nKO mice.

(**A–H**) Western blots and relative quantifications of cortical and hippocampal homogenates of 5-month-old wild-type (WT), *Ndufs3*-nKO+GFP (KO + GFP), and *Ndufs3*-nKO+*NdufsS3* (KO+Ndufs3) female mice. Probed for NDUFS3 and NDUFB8 (Complex I subunits), SDHA (Complex II subunit), CORE1 (Complex II subunit, COX1 (Complex IV subunit), and VDAC (mitochondrial membrane protein). Total protein loading was used as loading control. All protein loading staining and their respective blots are shown in Appendix Fig. S5. (**I, J**) BN-PAGE and relative quantifications of steady-state levels of respiratory complexes normalized to CII levels. (**K, L**) BN-PAGE in gel activity and relative quantifications of enzymatic activity. Data information: In (**B, C, F, G, J** (CIII)), data are represented as mean ± SD ($n = 3$/group). *P* values were calculated using two-way ANOVA, with Tukey's multiple comparisons test. In (**D, H, J, L**), data are represented as mean ± SD ($n = 3$/group). *P* values were determined by one-way ANOVA, with Tukey's multiple comparisons test. $P(*) = 0.0332$, $P(**) = 0.0021$, $P(***) = 0.0002$, $P(****) < 0.0001$. Exact *P* values are listed in Appendix Table S1. Source data are available online for this figure.

 © The Author(s)

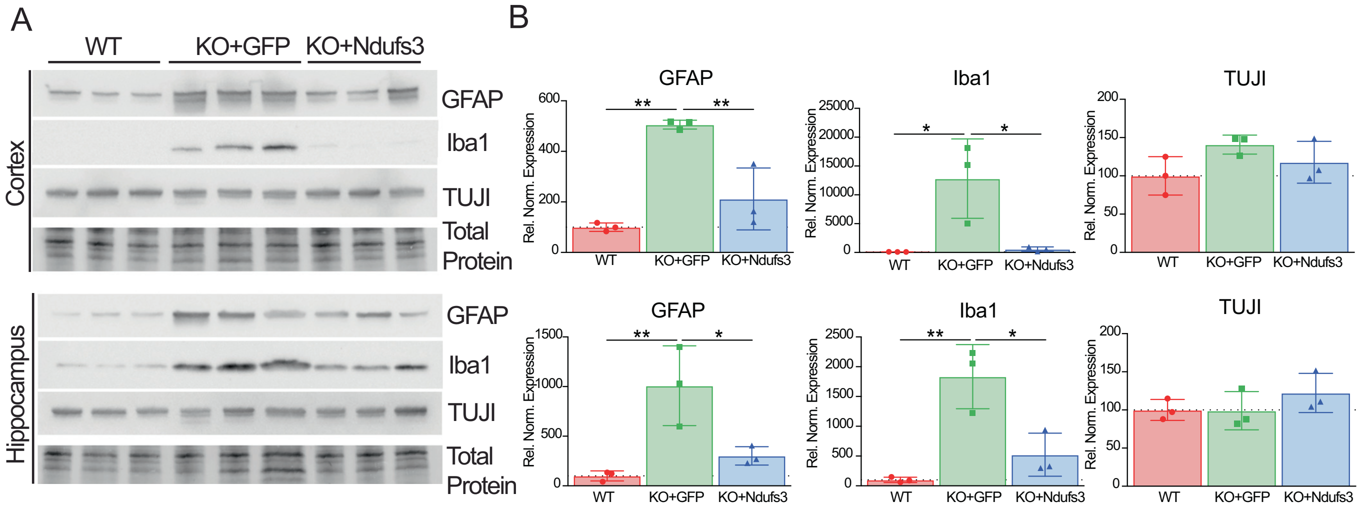

Figure EV2. Figure 6. Neuroinflammation in *Ndufs3*-nKO female mice is improved by CNS gene therapy.

(**A**, **B**) Western blots and relative quantification of protein homogenates from cortex and hippocampus of female WT, KO+*eGFP*, and KO+*Ndufs3* mice at 5 months of age, probing for astrocyte activation (GFAP), microglial marker IBA1, and neuronal marker TUJ1. Total protein loading was used as loading control. All protein loading staining and their respective blots are shown in Appendix Fig. S5. Data Information: In (**B**), data are represented as mean ± SD ($n = 3$/group). *P* values were calculated using one-way ANOVA, with Tukey's multiple comparisons test. $P(*) = 0.0332$, $P(**) = 0.0021$. Exact *P* values are listed in Appendix Table S1. Source data are available online for this figure.

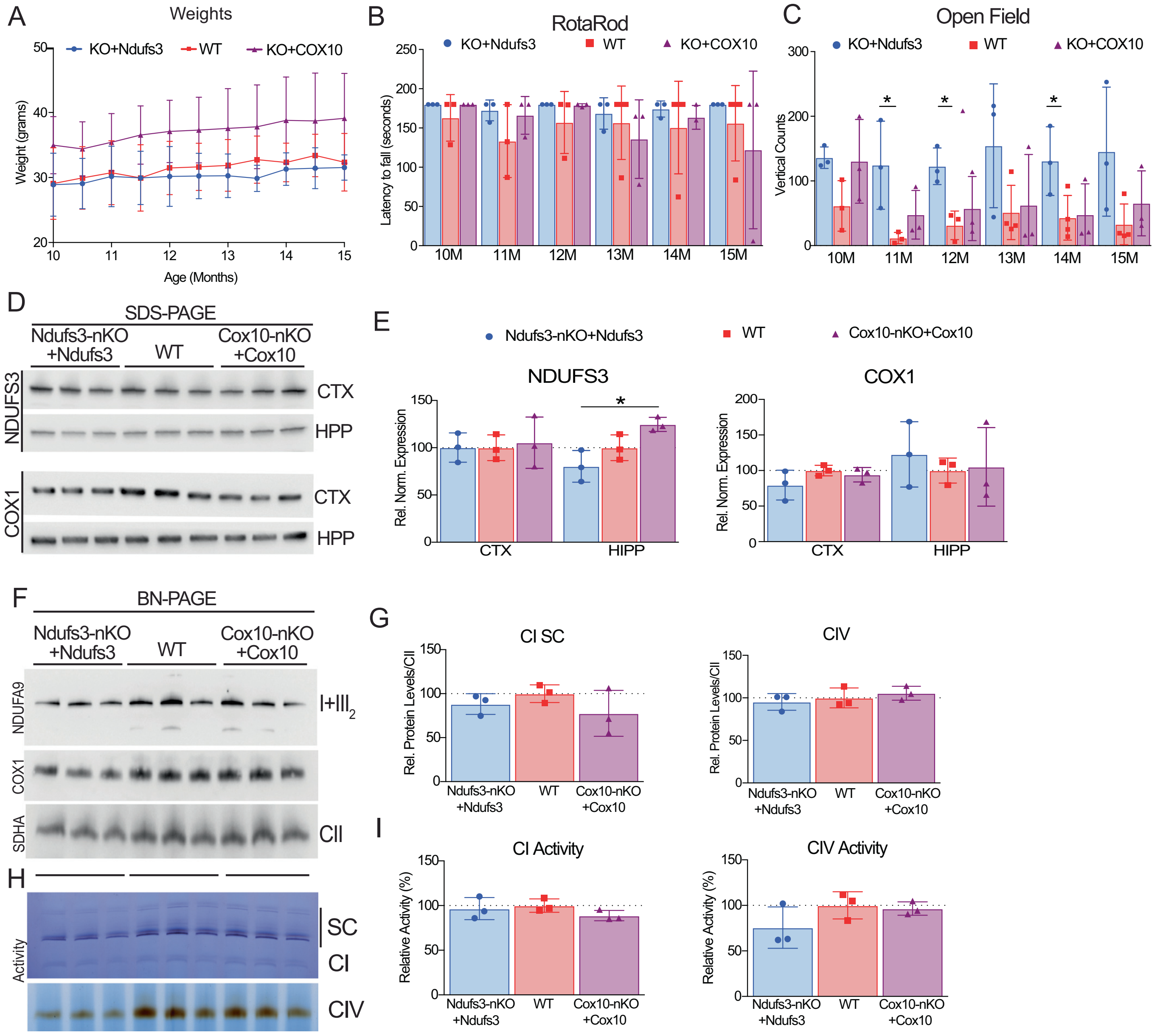

Figure EV3. Extended survival study.

(**A**) Weekly weights of *Ndufs3*-nKO+*NdufsS3* (blue), *Cox10*-nKO+*Cox10* (purple), and WT (red) mice between 10 and 15 months of age. (**B**) Rotarod performed by *Ndufs3*-nKO+*Ndufs3* (blue), WT (red), and *Cox10*-nKO+*Cox10* (purple) mice at 10, 11, 12, 13, 14, and 15. (**C**) Vertical counts recorded during open field analysis of *Ndufs3*-nKO+*Ndufs3* (blue), WT (red), and *Cox10*-nKO+*Cox10* (purple) mice at 10, 11, 12, 13, 14, and 15. (**D, E**) Western blot and relative quantifications of NDUFS3 and COX1 in cortex and hippocampal homogenates of 15-month-old animals. Total protein loading was used as loading control. All protein loading staining and their respective blots are shown in Appendix Fig. S5. (**F, G**) BN-PAGE and relative quantifications of steady-state levels of respiratory complexes, normalized to CII levels. (**H, I**) BN-PAGE in gel activity and relative quantifications of enzymatic activity. Data information: In (**A, B, C, E**), data are represented as mean ± SD ($n = 3$–4/group). *P* values were calculated using two-way ANOVA, with Tukey's multiple comparisons test, compared to WT. In (**G, I**), data are represented as mean ± SD ($n = 3$/group). *P* values were determined by one-way ANOVA, with Tukey's multiple comparisons test, compared to WT. Exact *P* values are listed in Appendix Table S1. Source data are available online for this figure.

 © The Author(s)

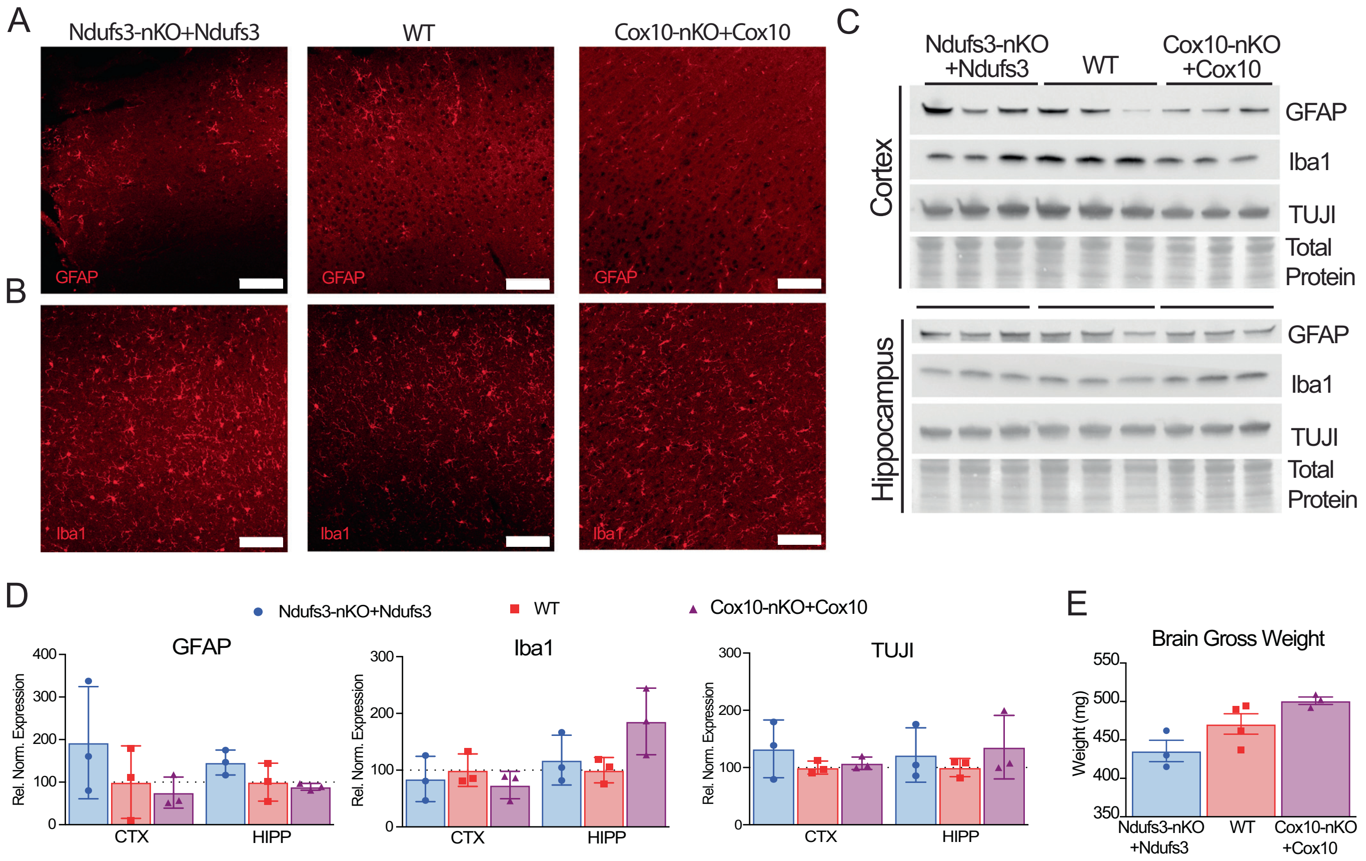

Figure EV4. Neuropathology of 15-month-old OXPHOS-nKO mice after gene therapy.

(**A**) Immunohistochemical images of GFAP in motor cortex of 15-month-old male mice. Scale bar is 100 μm. (**B**) Immunohistochemical images of IBA1 in motor cortex of 15-month-old male mice. Scale bar is 100 μm. (**C, D**) Western blots and relative quantification of protein homogenates from cortex and hippocampi of *Ndufs3*-nKO-*Ndufs3*, WT, and *Cox10*-nKO+*Cox10* mice at 15 months of age, probing for GFAP, IBA1, and TUJ1. Total protein loading was used as loading control. All protein loading staining and their respective blots are shown in Appendix Fig. S5. (**E**) Brain weight of 15-month-old *Ndufs3*-nKO+*Ndufs3* (blue), WT (red), and *Cox10*-nKO+*Cox10* (purple), mixed sex. Data information: In (**D**), data are represented as mean ± SD ($n = 3$/group). *P* values were calculated using two-way ANOVA with Tukey's multiple comparisons test, compared to WT. In (**E**), data are represented as mean ± SD ($n = 3$–4/group). *P* values were determined by one-way ANOVA, with Tukey's multiple comparisons test, compared to WT. $P(*) = 0.0332$. Exact *P* values are listed in Appendix Table S1. Source data are available online for this figure.

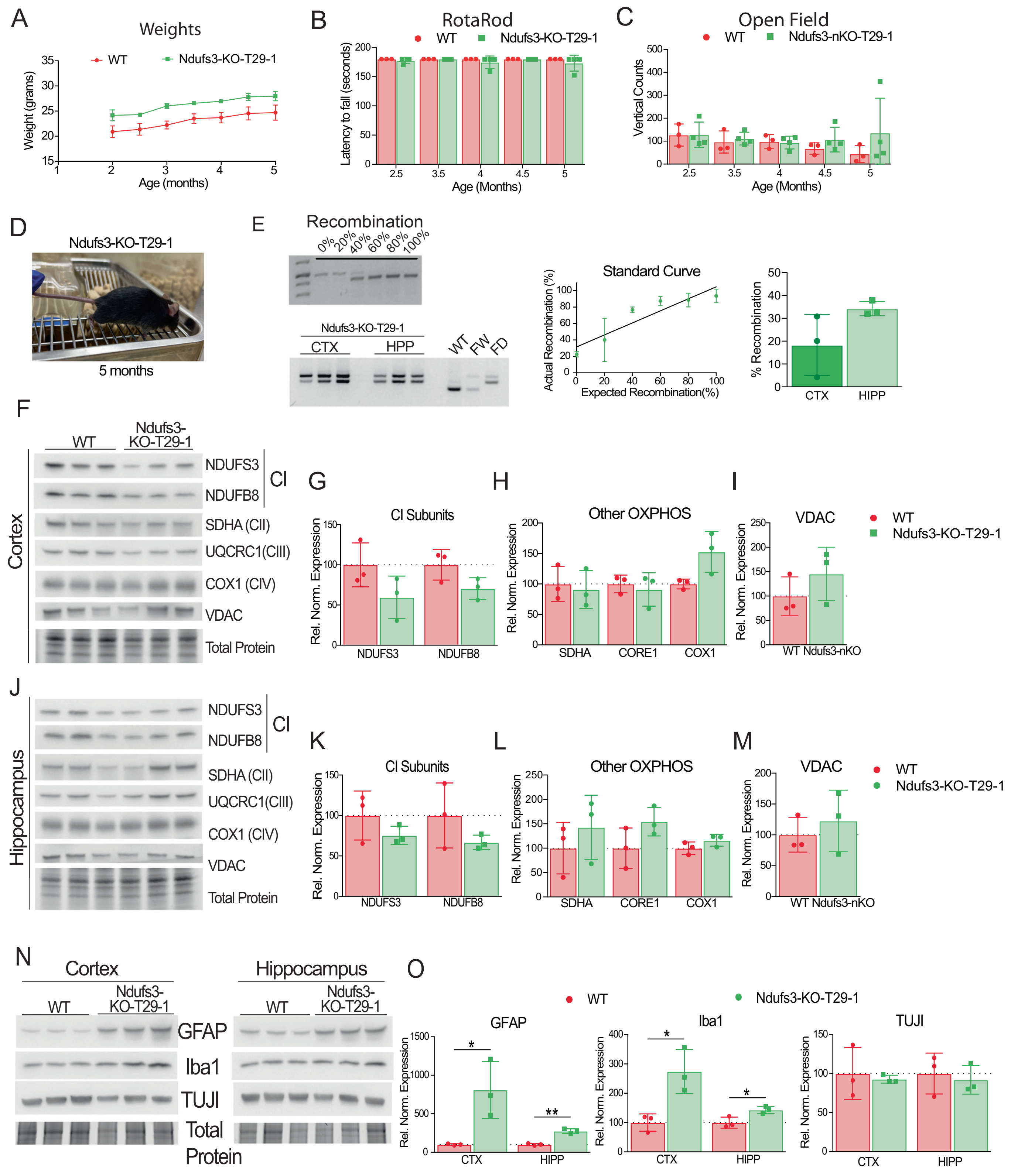

A
Weights
WT
Ndufs3-KO-T29-1
Weight (grams)
Age (months)
B
RotaRod
WT
Ndufs3-KO-T29-1
Latency to fall (seconds)
Age (Months)
C
Open Field
WT
Ndufs3-nKO-T29-1
Vertical Counts
Age (Months)
D
Ndufs3-KO-T29-1
5 months
E
Recombination
0% 20% 40% 60% 80% 100%
Ndufs3-KO-T29-1
CTX
HPP
WT FW FD
Standard Curve
Actual Recombination (%)
Expected Recombination(%)
% Recombination
CTX
HIPP
F
WT
Ndufs3-KO-T29-1
Cortex
NDUFS3
NDUFB8
CI
SDHA (CII)
UQCRC1(CIII)
COX1 (CIV)
VDAC
Total Protein
G
CI Subunits
Rel. Norm. Expression
NDUFS3
NDUFB8
H
Other OXPHOS
SDHA
CORE1
COX1
I
VDAC
WT Ndufs3-nKO
WT
Ndufs3-KO-T29-1
J
Hippocampus
NDUFS3
NDUFB8
CI
SDHA (CII)
UQCRC1(CIII)
COX1 (CIV)
VDAC
Total Protein
K
CI Subunits
NDUFS3
NDUFB8
L
Other OXPHOS
SDHA
CORE1
COX1
M
VDAC
WT Ndufs3-nKO
WT
Ndufs3-KO-T29-1
N
Cortex
Hippocampus
WT
Ndufs3-KO-T29-1
GFAP
Iba1
TUJI
Total Protein
O
WT
Ndufs3-KO-T29-1
GFAP
Iba1
TUJI
CTX
HIPP

© The Author(s)

Figure EV5. T29-1-cre driven CNS deletion of *Ndufs3* does not cause overt CNS phenotypes.

(**A**) Weekly weights of *Ndufs3*-nKO-T29-1 mice over the course of the age-matched study. (**B**) Rotarod performed by WT (red) and *Ndufs3*-nKO-T29-1 (green) mice at 2.5, 3.5, 4, 4.5, and 5 months of age. (**C**) Vertical counts recorded during open field analysis of *Ndufs3*-nKO-T29-1 mice at 2.5, 3.5, 4, 4.5, and 5 months of age. (**D**) Representative image of 5-month-old *Ndufs3*-nKO-T29-1 mice. (**E**) Representative gel of three primer PCR, showing recombination of *Ndufs3*-nKO-T29-1 mice at 8 months of age. Quantification of the three primer PCR for cortex and hippocampus. (**F–M**) Western blots and relative quantifications of protein homogenates from cortex and hippocampus of 8-month-old wild-type (WT) and *Ndufs3*-nKO-T29-1 male mice probed for NDUFS3 and NDUFB8 (Complex I subunits), SDHA (Complex II subunit), UQCRC1 (Complex III subunit), COX1 (Complex IV subunit), and VDAC (mitochondrial membrane protein). Total protein loading was used as loading control. All protein loading staining and their respective blots are shown in Appendix Fig. S5. (**N**, **O**) Western blots and relative quantification of protein homogenates from cortex and hippocampus of males WT and Ndufs3-nKO-T29-1 mice at 8 months of age, probing for astrocyte activation (GFAP), microglial marker IBA1, and neuronal marker TUJ1. Total protein loading was used as loading control. All protein loading staining and their respective blots are shown in Appendix Fig. S5. Data information: Data are represented as mean ± SD ($n = 3$/group). *P* values were calculated using Welch's t-test, or multiple t-tests with Holm Sidak's multiple comparisons test. $P(*) = 0.0332$. Exact *P* values are listed in Appendix Table S1. Source data are available online for this figure.

© The Author(s)
